# Thermodynamic Preference Between Deprotonation Pathways in Boronic Acid-Based Proteasome Inhibitors: Insights from a DFT Study

**DOI:** 10.3390/ph19081251

**Published:** 2026-08-08

**Authors:** Nikolay Toshev, Iliyan Dimitrov, Ovanes Muradyan, Vassil Delchev, Todor Dudev

**Affiliations:** 1 Department of Bioorganic Chemistry, Faculty of Pharmacy, Medical University of Plovdiv, 15A Vassil Aprilov Blvd., 4002 Plovdiv, Bulgaria; 2 Department of Medical Biochemistry, Faculty of Pharmacy, Medical University of Plovdiv, 15A Vassil Aprilov Blvd., 4002 Plovdiv, Bulgaria; iliyan.dimitrov@mu-plovdiv.bg; 3 Medical Faculty, Medical University of Plovdiv, 15A Vassil Aprilov Blvd., 4002 Plovdiv, Bulgaria; ovanes.m301204@gmail.com; 4 Department of Physical Chemistry, Faculty of Chemistry, Plovdiv University “Paisii Hilendarski”, 24 Tsar Asen Str., 4000 Plovdiv, Bulgaria; vdelchev@uni-plovdiv.net; 5 Department of Pharmaceutical and Applied Organic Chemistry, Faculty of Chemistry and Pharmacy, Sofia University “St. Kliment Ohridski”, 1 James Bourchier Blvd., 1164 Sofia, Bulgaria; ohtttd@chem.uni-sofia.bg

**Keywords:** cancer, anticancer drugs, proteasome inhibitors, boronic acid-based proteasome inhibitors, Bortezomib, Ixazomib, Delanzomib, warhead model, deprotonation mechanism

## Abstract

**Background/Objectives**: Boronic acid-based proteasome inhibitors (BABPIs) including Bortezomib, Ixazomib, and Delanzomib are clinically relevant anticancer agents whose mechanism of action depends on direct interaction between the boronic acid warhead and the threonine residue at the first position (Thr1), leading to the formation of covalent tetrahedral complex. Although the formation of this complex is well studied, the subsequent behavior of the boronic acid warhead, particularly the possible formation of monoanionic boronate species through deprotonation of one of the two boronic hydroxyl groups, remains unexplored. Therefore, the present study addresses whether the formation of Thr1-OH and a monoanionic boronate species is thermodynamically favorable and which of the two hydroxyl groups is more favorable for deprotonation. **Methods**: Density Functional Theory (DFT) calculations at the B3LYP/6-311+G(d,p) level, combined with the Polarizable Continuum Model (PCM), were used to study two competing deprotonation pathways for the inhibitors and for a simplified warhead model. To mimic the proteasome environment, reactions were modeled in different polar media—diethyl ether (ε = 4), methanol (ε = 33), and water (ε = 78). **Results**: Our DFT calculations confirmed that the covalent tetrahedral complex could convert into Thr1-OH and a monoanionic boronate species, representing the deprotonation of one of the boronic hydroxyl groups. Deprotonation via pathway 1 is more favorable than deprotonation via pathway 2 for all inhibitors, especially in polar solvents. Bortezomib demonstrated a strong preference for -OH_1_ deprotonation with ∆∆G ≈ −7 kcal·mol^−1^. In contrast, Ixazomib, Delanzomib, and the simplified warhead model showed smaller ∆∆G values (≈−2 kcal·mol^−1^), within the method’s uncertainty (±2 kcal·mol^−1^), suggesting both deprotonation modes under physiological conditions. **Conclusions**: These results provide comparative thermodynamic insight into the deprotonation behavior of BABPIs, suggesting that the two hydroxyl groups are not equivalent during deprotonation. This finding offers a physicochemical framework that may support the rational design of next-generation BABPIs.

## 1. Introduction

### 1.1. Proteasome Structure and Function

The proteasome is a high-molecular-weight enzyme complex involved in intracellular, ubiquitin-mediated proteolysis. It consists of two types of protein components—a 19S regulatory subunit that recognizes ubiquitin-bound proteins targeted for proteolysis and a 20S proteolytic subunit [1,2]. The central 20S area possesses a unique cylindrical structure, organized in four heteroheptameric, tightly packed rings—two centrally placed, called β, and two peripheral, α. Each of the β-rings consists of β_1_–β_7_ subunits and has three different active sites. Each active site has its own specific substrate binding pocket (S), whose features are determined by the surrounding amino acid residues. The active sites are formed after proteolytic cleavage of the β1/PRE3, β2/PUP1, and β5/PRE2 subunits, where the threonine residue on the first position (Thr1) appears to be of key importance for the nucleophilic attack on the target peptide bond. All three subunits also have contact sites where they interact with proteasome inhibitors (PIs) [2,3,4].

### 1.2. Mechanism of Proteasome Action

The proteasome belongs to the family of N-terminal nucleophile hydrolases, in which the N-terminal Thr1 residue acts as the catalytic nucleophile. In the normal proteolytic mechanism, proteasomes follow a common pathway that is typical for N-terminal nucleophile hydrolases, as described by Myung et al. [5].

In general, Thr1 activation requires proton transfer from its hydroxyl group (Thr1-OH) to its free NH_2_-group (Thr1-NH_2_) yielding Thr1-O^−^ and Thr1-NH_3_^+^. It is proposed that the proton transfer occurs more likely through an intermediate water molecule. Later, the activated Thr1-O^−^ performs a nucleophilic attack on the carbonyl carbon of the substrate peptide bond, leading to peptide bond cleavage and substrate degradation. Finally, the end products leave the active site after a proton transfer from Thr1-NH_3_^+^, while Thr1-O^−^ accepts a proton from a water molecule to produce Thr1-OH, allowing the enzyme to participate in subsequent hydrolytic peptide-bond cleavage cycles [5].

### 1.3. Proteasome Inhibition as an Anticancer Strategy

Proteasome inhibition has emerged as a promising therapeutic approach in cancer therapy due to the proteasome’s central role in regulating cellular protein turnover, and, consequently, cell metabolism, cell cycle progression, and apoptosis. The application of PIs causes cytotoxicity due to turning off cell survival pathways, turning on apoptotic pathways, and increasing endoplasmic reticulum stress [6,7,8,9,10,11]. Moreover, proteasome activity is frequently affected in various cancers, including multiple myeloma and lymphomas [12]. These findings serve as a basis of interest in understanding the mechanisms of proteasome inhibition, with emphasis on the structural factors that govern inhibitor binding, reversibility, and release. Thus, the development of new PIs may help circumvent binding-site mutations, as well as broaden the range of inhibition, including other catalytically active subunits. Furthermore, developing drug resistance might be overcome by combining conventional and novel inhibitors, allowing synergistic targeting [13]. However, the design of such proteasome-specific inhibitors must be considered with the fact that among enzymes with protease activity, proteasomes specifically utilize the hydroxyl group of their Thr1 [14].

### 1.4. BABPIs: Mechanism of Action and Clinically Relevant Inhibitors

Boron-containing compounds have attracted scientific attention in medicinal chemistry due to the specific electronic properties of boron, which has even been described as a “magic element” in biomedicine [15]. The importance of boron arises from its electron-deficient nature and Lewis-acidic character, as well as its ability to form specific interactions with biological targets, including proteins and enzymes [16]. The clinical success of Bortezomib, followed by subsequent approvals of other boron-containing drugs such as Ixazomib, has underscored the value of boron chemistry in modern drug design [17]. Furthermore, natural products continue to provide structurally diverse proteasome-inhibitory scaffolds and remain an important source for the development of next-generation proteasome inhibitors [18].

BABPIs are considered one of the most important classes of PIs, since they are highly selective due to the nature of their boronic acid warhead. The boronic acid warhead possesses high affinity for hard oxygen nucleophiles, with the boron atom interacting with the nucleophilic oxygen of Thr1. That renders boronates as less prone to interactions with the soft nucleophiles of cysteine proteases [14,19].

**Figure 1:**
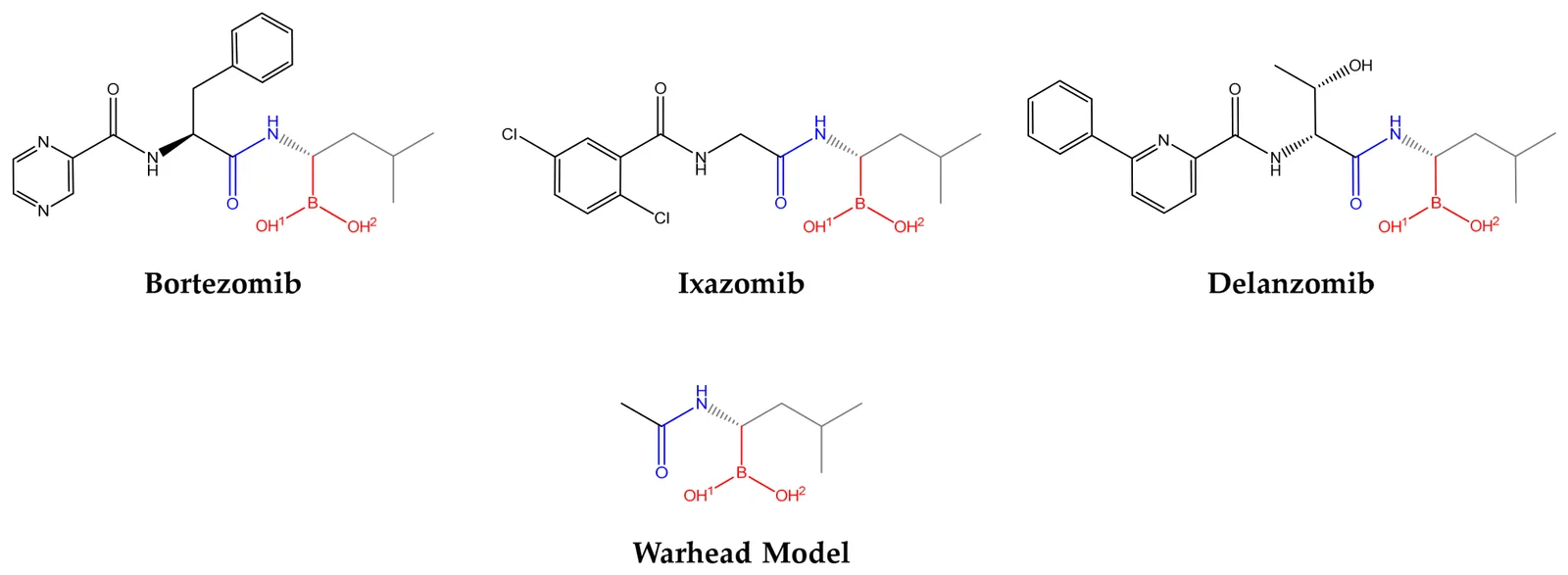
Chemical structures of the studied BABPIs and the simplified warhead model. The boronic acid warhead is highlighted in red, the peptide bond in blue, and the isobutyl chain in gray. The two hydroxyl groups of the boronic acid warhead are denoted as OH_1_ and OH_2_, to indicate their chemical non-equivalence and potentially different deprotonation behavior.

Bortezomib, Ixazomib, and Delanzomib are BABPIs with similar dipeptidyl-based structures—a boronic acid warhead, an isobutyl side chain, and the same chiral center, where the boronic acid warhead is attached. Their molecules resemble the shape of the proteasome’s natural substrates, allowing them to interact with key amino acid residues in the active site. They show a high rate of selectivity to proteasomes and some of them tend to indicate low resistance in vivo [20,21,22,23,24,25,26,27]. The structures of the abovementioned molecules are shown in Figure 1, and their subunit selectivity, therapeutic relevance, and clinical status of the studied molecules aresummarized in Table 1.

Despite the peptide-like structure of the BABPIs, the key for the enzyme activity Thr1-O^−^ launches nucleophilic attack on the boronic acid warhead, instead of on the carbonyl atom of the peptide bond, thus forming a covalent tetrahedral boronate complex without peptide bond cleavage [14]. This contrasts with the general proteolytic process, separating the boronic acid-based inhibition from the peptide-bond directed mechanism and underlines the specific reactivity of boronic acid-based proteasome inhibitors.

### 1.5. Pharmacophore Model of BABPIs

BABPIs can be described by a pharmacophore model composed of five structural elements: a hydrogen bond acceptor (HBA), a hydrogen bond donor (HBD), two hydrophobic groups (HY), and a boronic acid warhead. This model was previously proposed and validated to study the binding and inhibitor properties of dipeptidyl BABPIs [29]. The HBA and HBD units are represented by the amide carbonyl (C=O) and the adjacent N-H group, respectively. Aromatic and aliphatic units of the inhibitors facilitate hydrophobic interactions, supporting molecular recognition within the active site. The boronic acid group is involved in the formation of the tetrahedral boronate complex in the enzyme active site [14]. Notably, this unique architecture underpins the inhibitors’ activity across the aforementioned inhibitors.

### 1.6. Research Gap and Study Objectives

The present work focuses on Bortezomib, Ixazomib, and Delanzomib as representative BABPIs, along with a simplified warhead model, to examine the thermodynamic feasibility of a potential transformation of the tetrahedral boronate complex formed through the interaction between BABPIs and Thr1. Although the formation of this covalent complex plays a key role in proteasome inhibition, less attention has been focused on its potential conversion to products, which may have implications for reversibility and inhibitor release. Specifically, we propose that the tetrahedral intermediate can rearrange into a Thr1-OH, while the inhibitor is converted into a monoanionic boronate species. This process would arise from the proton transfer from one of the boronic hydroxyl groups to the deprotonated threonine residue, without focusing on the exact steps or transition states involved. One of the central questions of this study is whether such a transformation is thermodynamically feasible under conditions mimicking the physiological environment.

**Scheme 1:**
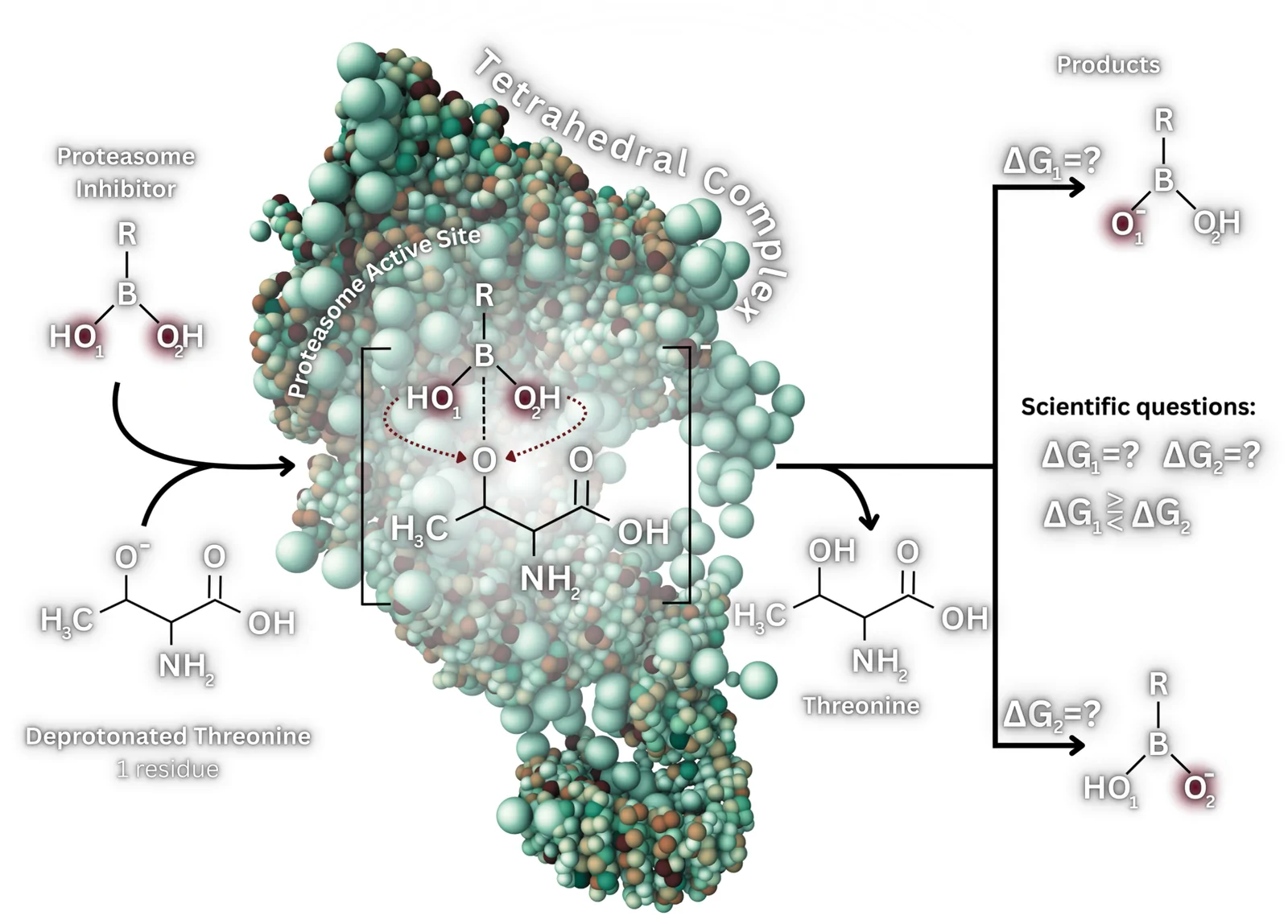
Schematic illustration of the two competing proton-transfer pathways examined in this study for BABPIs (created in https://www.canva.cn/en/?display-com-option=true, accessed on 31 July 2026).

A schematic representation of the two competing proton-transfer pathways is shown in Scheme 1.

In this context, computational approaches have emerged as valuable tools for studying proteasome inhibitors, including the modeling of 20S proteasome active site mechanisms and boronic acid-containing warheads [30,31].

Despite extensive research on BABPIs, the thermodynamic preference for deprotonation of the boronic hydroxyl groups remains insufficiently understood. To the best of our knowledge, no systematic DFT-based study has addressed the question about the thermodynamic preference for deprotonation of one hydroxyl group over the other in BABPIs. Moreover, the influence of solvent polarity, employed to mimic different regions of the proteasome active site, on proton transfer energetics remains unexplored.

To fill this knowledge gap, the present study addresses the following questions: (1) Is the proton transfer from the inhibitor to the catalytic Thr1 thermodynamically favorable? (2) Which of the two hydroxyl groups (OH_1_ or OH_2_) on the boronic acid warhead of the proteasome inhibitor is more favorable to undergo deprotonation? (3) Does the preferred deprotonation site remain consistent across different solvents that mimic regions within the proteasome active site? (4) How accurately does the proposed simplified warhead model reproduce the deprotonation behavior of the studied molecules?

To address these questions, we employed DFT calculations at the B3LYP/6-311+G(d,p) level of theory, combined with the PCM, to study the thermodynamics of proton transfer from BABPIs to the catalytic threonine residue in different media. This computational approach aims to evaluate whether the proposed transformation of the tetrahedral boronate complex is thermodynamically feasible and to clarify the factors governing the deprotonation behavior of the studied molecules.

## 2. Results and Discussion

### 2.1. Optimized Geometries of the Studied Inhibitors

The investigated BABPIs share a common scaffold that includes a boronic acid warhead, HBD and HBA groups, and hydrophobic/aromatic moieties. These pharmacophoric elements are consistent with the ligand-based peptide-boronate pharmacophore proposed for dipeptidyl BABPIs [29]. In this study, Bortezomib, Ixazomib, Delanzomib, and the simplified warhead model were optimized at the B3LYP/6-311+G(d,p) level of theory using the PCM solvation model. This DFT-based study provides a structural analysis of the optimized geometries of these clinically relevant inhibitors and the simplified warhead model as a basis for the subsequent thermodynamic analysis.

**Figure 2:**
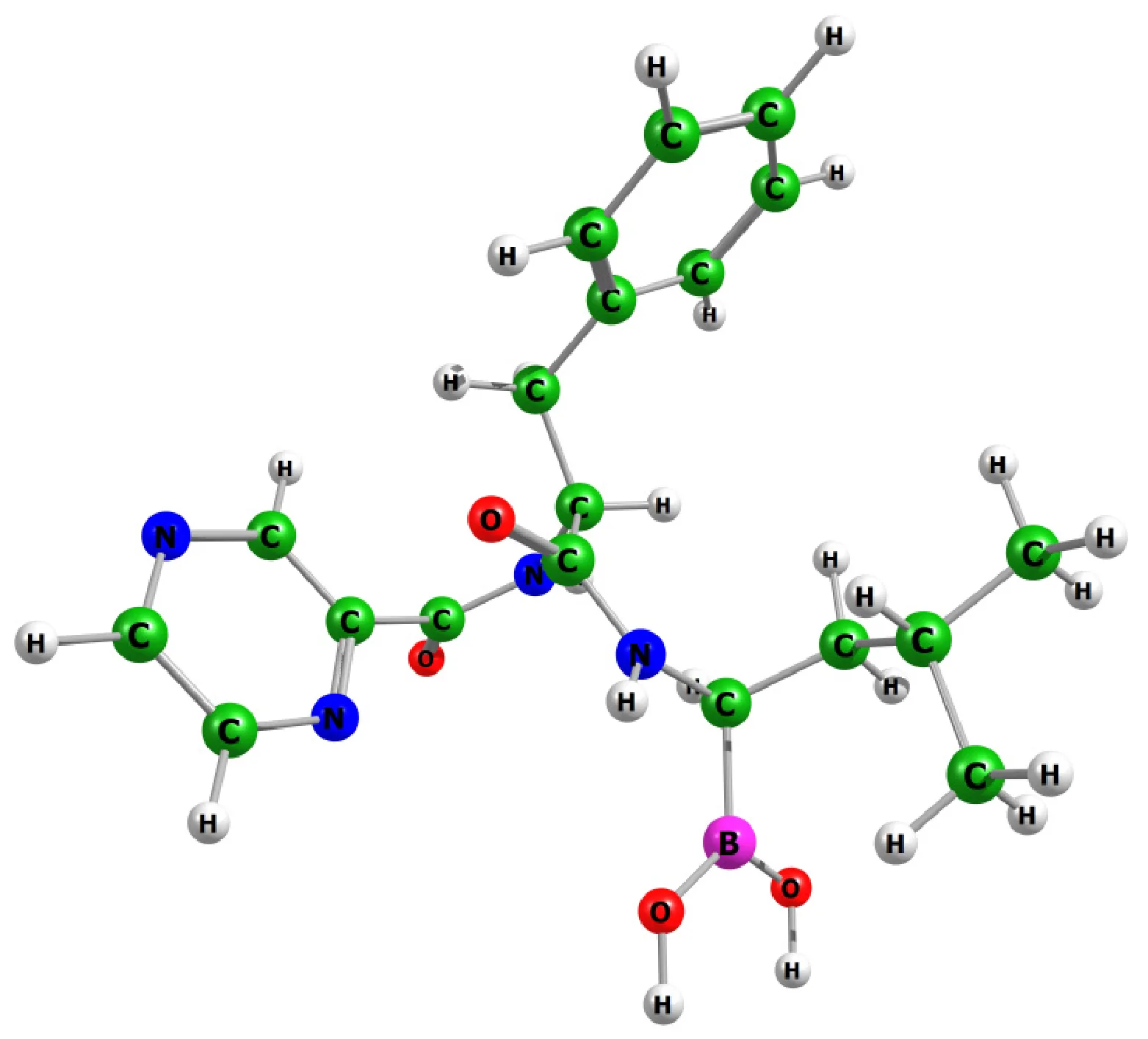
B3LYP/6-311+G(d,p) optimized geometry of Bortezomib in water using the PCM solvation model.

Bortezomib has a peptide structure characterized by a pyrazinoyl-phenylalanine-leucine backbone, in which the boronic acid group substitutes the terminal carboxyl group, acting as the reactive warhead. The amide group acts as an HBA, while the N-H bonds act as hydrogen bond donors. In this molecule, the aromatic phenyl ring and the isobutyl side chain of leucine form two different hydrophobic regions. The optimized geometry of Bortezomib is presented in Figure 2.

**Figure 3:**
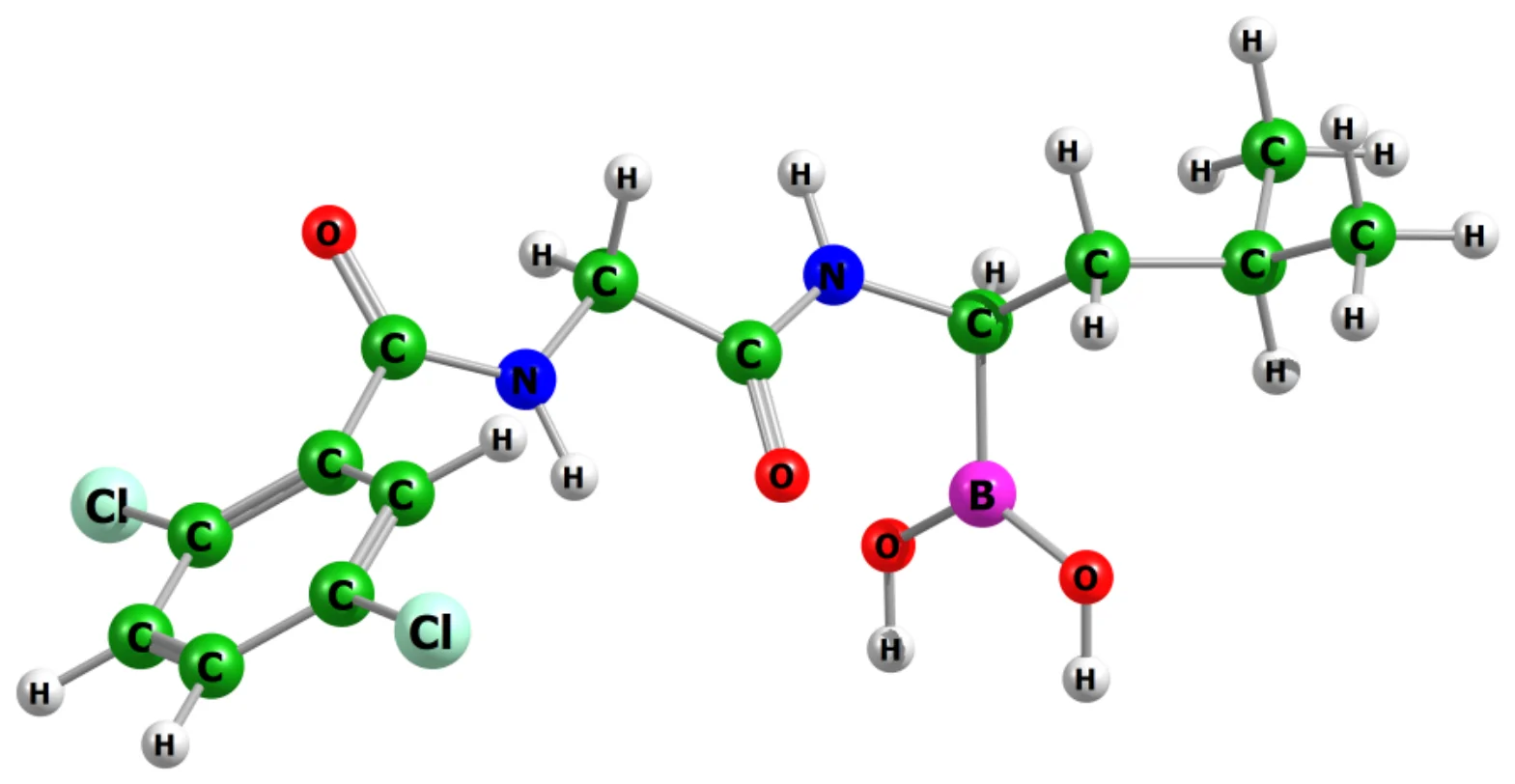
B3LYP/6-311+G(d,p) optimized geometry of Ixazomib in water using the PCM solvation model.

Ixazomib comprises a dichlorobenzoyl-glycine amide unit linked to a dihydroxyborane-3-methylbutyl fragment. Similar to Bortezomib, it contains an amide carbonyl group, acting as an HBA, and an N-H group (HBD), and both a dichlorophenyl ring and an alkyl group, acting as hydrophobic/aromatic substituents. The optimized geometry of Ixazomib is presented in Figure 3.

**Figure 4:**
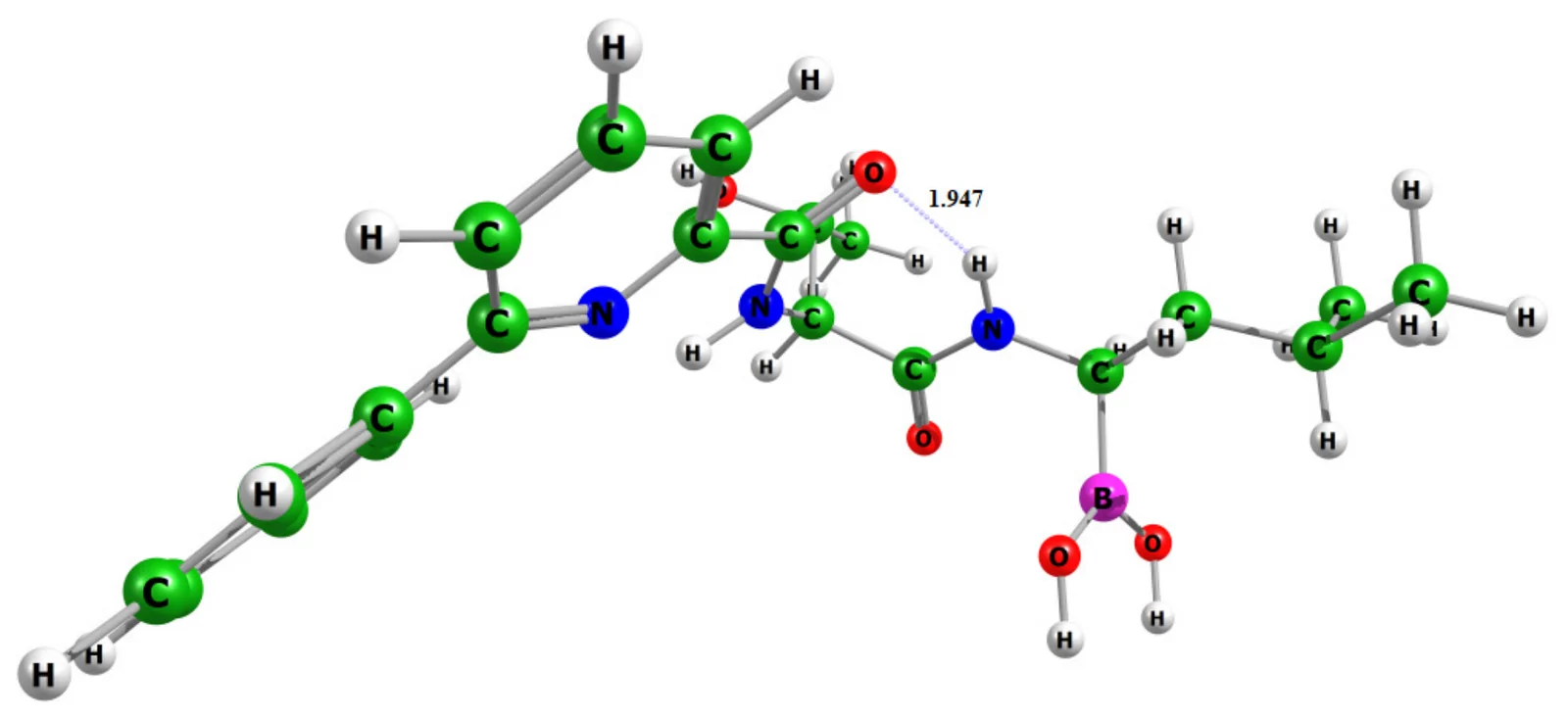
B3LYP/6-311+G(d,p) optimized geometry of Delanzomib in water using the PCM solvation model.

Delanzomib consists of a 6-phenylpyridine-2-carboxamide moiety connected through an amide nitrogen to a 3-hydroxy-2-aminobutyryl unit, a boronic acid warhead, and an isobutyl side chain. In the optimized geometry, an additional intramolecular stabilizing interaction of approximately 1.95 Å was observed between the carbonyl group oxygen and the hydrogen from the amide group connected to the isobutyl moiety. The optimized geometry of Delanzomib is presented in Figure 4.

**Figure 5:**
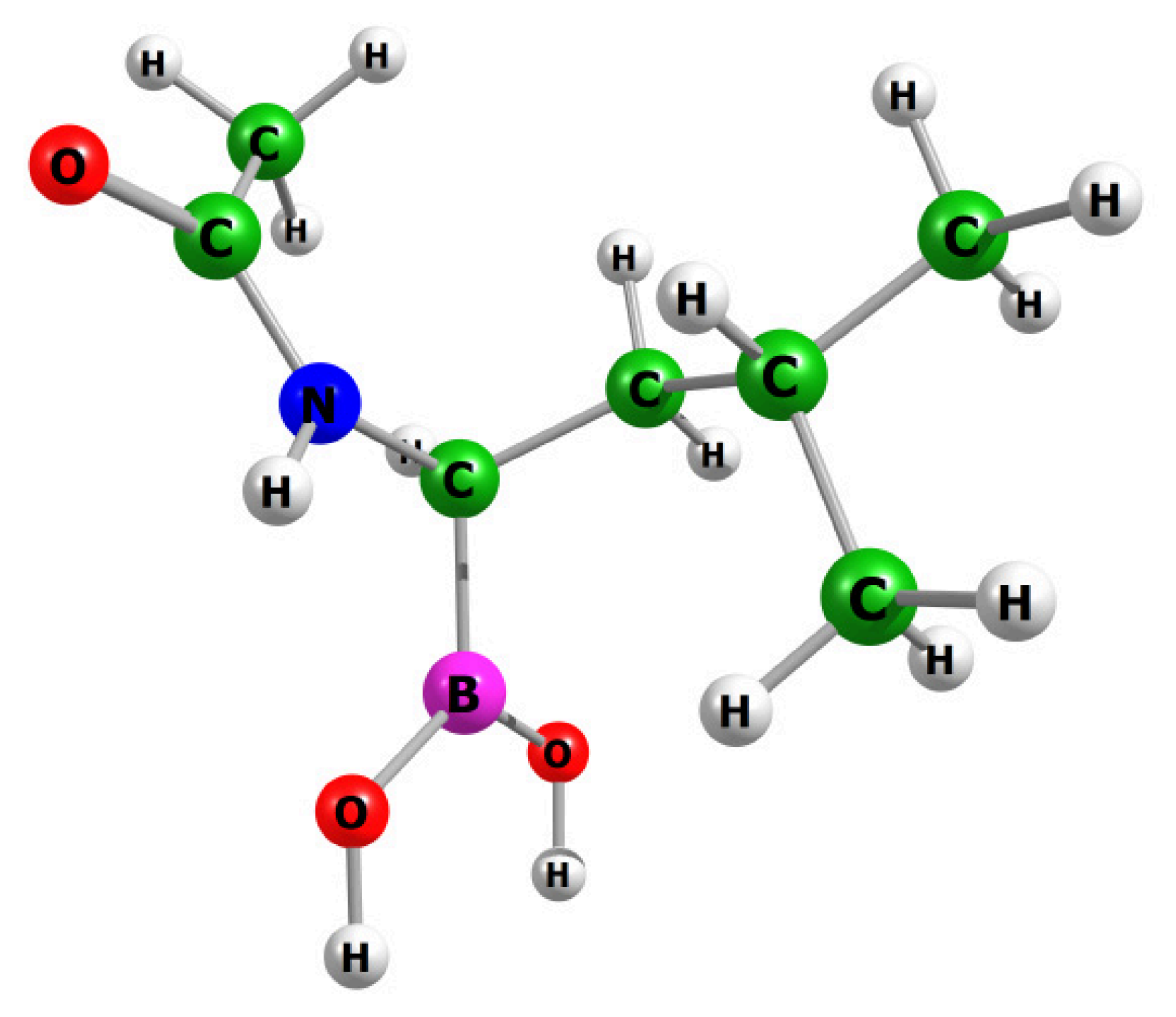
B3LYP/6-311+G(d,p) optimized geometry of the simplified warhead model in water using the PCM solvation model.

The simplified warhead model was optimized to evaluate the intrinsic structural and electronic behavior of the boronic acid warhead independently of the larger inhibitor scaffold. The optimized geometry of the simplified warhead model is presented in Figure 5.

### 2.2. Structural Validation of the Computational Protocol

Before analyzing the thermodynamics of proton transfer, the reliability of the chosen computational protocol was evaluated by comparing representative DFT-calculated bond lengths for Bortezomib and Ixazomib using the B3LYP/6-311+G(d,p) level of theory in polar media (PCM), which are summarized and compared with the corresponding crystallographic data [14,32]. The analysis was focused on key structural parameters within the boronic acid warhead and peptide backbone, namely B-O^1^, B-O^2^, B-C, C=O, and N-C bond distances. These results are shown in Table 2.

In all studied molecules, the B-O^1^ and B-O^2^ bond lengths were approximately 1.37 Å, in good agreement with experimental values obtained from crystallographic data (approximately 1.43 Å). The B-C bond lengths were calculated between 1.59 and 1.60 Å, aligning well with experimental values for Bortezomib and Ixazomib (1.56–1.57 Å). The peptide-backbone bond distances, specifically C=O and N-C, are closely aligned with crystallographic data (C=O ≈ 1.23 Å and N-C ≈ 1.34 Å). The bond lengths obtained from the optimized geometries of the molecules studied show good agreement with previously reported experimental data for Bortezomib and Ixazomib, with differences typically within 0.005–0.065 Å. This close match supports the reliability of the chosen computational protocol, and the results obtained can be considered as suitable for thermodynamic analysis of proton-transfer reactions. It should be noted that this comparison mainly validates the optimized geometries, while the sensitivity of the calculated Gibbs free energies to the basis set and density functional was additionally assessed through benchmark calculations presented in Tables S1 and S2 of the Supplementary Materials.

### 2.3. Solvation Analysis of the Studied Proteasome Inhibitors

The computed relative solvation Gibbs free energies of the BABPIs and the simplified warhead model are summarized in Table 3.

All studied inhibitors demonstrated stabilization in polar solvents, with water providing the strongest effect. Bortezomib showed the highest stabilization in water (∆G^78^ = −18.01 kcal·mol^−1^), followed by Delanzomib (∆G^78^ = −15.96 kcal·mol^−1^), and Ixazomib (∆G^78^ = −15.67 kcal·mol^−1^). The simplified warhead model, which was designed in this study to mimic the pharmacophore with minimized and simplified size, exhibited the lowest stabilization across the series (∆G^78^ = −12.07 kcal·mol^−1^), which can be attributed to its reduced molecular size, surface area, and heteroatom content.

Ixazomib and Delanzomib exhibited comparable stabilization values across all studied solvents. On the other hand, the simplified warhead model applied in this study effectively reproduced the general trend observed across the series. The differences between stabilization in methanol and water were typically low (±1 kcal·mol^−1^), indicating similar stabilization in these two polar environments. However, in diethyl ether, molecules have significantly decreased stabilization by 4.5–5.5 kcal·mol^−1^, thus demonstrating the sensitivity of the calculated solvation contribution to medium polarity.

### 2.4. Thermodynamic Feasibility of Proton Transfer from Boronic Hydroxyl Groups

Proteasome inhibition by BABPIs proceeds through the formation of a tetrahedral boronate complex with the N-terminal Thr1 residue. While this interaction is well described, the thermodynamic feasibility of a potential rearrangement of the complex—dissociating into Thr1-OH and a monoanionic boronate species—remains unstudied. Therefore, after establishing the optimized geometries and solvent-dependent stabilization, we evaluated whether this transformation may be thermodynamically feasible under conditions mimicking the proteasome active site, offering new insights into inhibitor reversibility and release mechanisms. This approach should be interpreted as a comparative thermodynamic analysis, rather than as a full description of inhibitor release or proteasome mechanism.

The analysis was performed for Bortezomib, Ixazomib, Delanzomib, and the simplified warhead model. All studied molecules have a boronic acid warhead with two hydroxyl groups attached to the boron atom that are chemically distinct and can serve as proton donors, leading to two rival deprotonation pathways. As a result, two deprotonation modes were considered: pathway 1, involving OH_1_ deprotonation; and pathway 2, involving OH_2_ deprotonation.

The following analysis examines the thermodynamic feasibility of a proton transfer, the preferred deprotonation site, and the influence of media on the reaction outcome.

#### 2.4.1. Deprotonation Pathway 1: Proton Transfer from OH_1_

The data in Table 4 indicate that proton transfer from hydroxyl group 1 (OH_1_) of the studied BABPIs and a warhead model is thermodynamically favorable for all studied molecules across all environments, with all ∆G values being negative and ranging from (−35.27 kcal·mol^−1^ to −15.53 kcal·mol^−1^).

In the gas phase (∆G^1^), the reactions are strongly exergonic, and the trend is as follows: warhead Model < Bortezomib < Delanzomib < Ixazomib, with Ixazomib having the highest affinity for deprotonation (∆G^1^ = −35.27 kcal·mol^−1^).

In diethyl ether (∆G^4^), a low-polarity media, the deprotonation exhibits a significant decrease in the absolute values of ∆G compared to the gas phase. This reduction of almost 10–15 kcal·mol^−1^ can be explained by partial stabilization of both reactants and products by the solvent. In diethyl ether, Ixazomib (∆G^4^ = −22.22 kcal·mol^−1^) and the warhead model (∆G^4^ = −19.16 kcal·mol^−1^) showed stronger affinities for deprotonation compared to Delanzomib (∆G^4^ = −17.14 kcal·mol^−1^) and Bortezomib (∆G^4^ = −16.66 kcal·mol^−1^), where the warhead model had a similar energy difference to Bortezomib (∆G^4^ = −16.01 kcal·mol^−1^). These results highlight the role of diethyl ether as a solvent that bridges gas-phase and high-polarity solvation behavior. In polar solvents such as methanol (∆G^33^) and water (∆G^78^), the reaction remains exergonic but slightly decreases compared to diethyl ether.

In methanol and water, Bortezomib is the most thermodynamically favorable deprotonated proteasome inhibitor (∆G^33^ = −22.48 kcal·mol^−1^ and ∆G^78^ = −22.61 kcal·mol^−1^), followed by Ixazomib (∆G^33^ = −17.80 kcal·mol^−1^ and ∆G^78^ = −17.38 kcal·mol^−1^), and with almost equal values, Delanzomib (∆G^33^ = −16.16 kcal·mol^−1^ and ∆G^78^ = −15.66 kcal·mol^−1^), and the warhead model (∆G^33^ = −16.01 kcal·mol^−1^ and ∆G^78^ = −15.53 kcal·mol^−1^). Furthermore, in both methanol and water, only minor differences within ±1 kcal·mol^−1^ were observed between the environments studied. Moreover, the simplified warhead model reproduces the overall deprotonation trend observed here with accuracy, differing from the inhibitors by less than 1 kcal·mol^−1^ in highly polar media and approximately 3–4 kcal·mol^−1^ in ether. These results should be interpreted as comparative thermodynamic trends rather than a complete description of proton transfer in the proteasome active site.

#### 2.4.2. Deprotonation Pathway 2: Proton Transfer from OH_2_

Similarly to pathway 1, all calculated Gibbs free energy values for pathway 2 are negative, indicating that the proton transfer from the second hydroxyl group 2 (OH_2_) of the studied BABPIs and a simplified warhead model to the Thr1-O^−^ is thermodynamically favorable under all studied conditions. The results are presented in Table 5.

In the gas phase (∆G^1^), the exergonicity follows the trend: Warhead model < Bortezomib < Delanzomib < Ixazomib, with Ixazomib having the highest affinity (∆G^1^ = −31.88 kcal·mol^−1^). This trend is almost identical to the one in pathway 1. Upon transition to diethyl ether (∆G^4^), an environment with moderate polarity, the Gibbs free energy values decrease by approximately 10–12 kcal·mol^−1^. Within this environment, Ixazomib and Bortezomib display the most favorable values (∆G^4^ = −19.29 kcal·mol^−1^ and ∆G^4^ = −17.19 kcal·mol^−1^), followed by the warhead model (∆G^4^ = −16.66 kcal·mol^−1^) and Delanzomib (∆G^4^ = −15.84 kcal·mol^−1^).

In methanol (ε = 32) and water (ε = 78), all ∆G values become less negative. Ixazomib again displays the most favorable deprotonation (∆G^33^ = −16.31 kcal·mol^−1^ and ∆G^78^ = −16.08 kcal·mol^−1^), followed by Bortezomib (∆G^33^ = −15.28 kcal·mol^−1^ and ∆G^78^ = −14.98 kcal·mol^−1^), and Delanzomib (∆G^33^ = −14.10 kcal·mol^−1^ and ∆G^78^ = −13.93 kcal·mol^−1^), and the warhead model (∆G^33^ = −14.45 kcal·mol^−1^ and ∆G^78^ = −14.01 kcal·mol^−1^), suggesting that there is a minimal influence of side chains on OH_2_ reactivity. In addition, as in pathway 1, the warhead model accurately represents the overall reactivity trends, varying with approximately ±2 kcal·mol^−1^ in water and methanol and ±3 kcal·mol^−1^ in ether.

Overall, the results involving deprotonation pathways 1 and 2 indicate that both hydroxyl groups (denoted as OH_1_ and OH_2_) on the boronic acid warhead can serve as potential proton donors under physiological-like conditions. This raises the question: Which of the two sites is thermodynamically preferred for deprotonation, and how does this preference vary across different BABPIs and solvent environments?

#### 2.4.3. Comparison of the Two Deprotonation Pathways

To understand the thermodynamic preference between the two potential reaction pathways of the studied inhibitors, we calculated the Gibbs free energy differences (∆∆G^x^ = ∆G^x^ (OH_1_) − ∆G^x^ (OH_2_), in kcal·mol^−1^) between pathway 1 and pathway 2 in different environments, thus mimicking different regions of the proteasome cavity. The results are summarized in Table 6.

The comparison of the calculated ∆∆G values indicates that deprotonation via pathway 1 (involving OH_1_) is thermodynamically more favorable for all studied molecules, particularly in polar solvents such as methanol and water.

Nevertheless, several important considerations must be considered to accurately explain these findings. Bortezomib exhibits the highest preference for pathway 1, with ∆∆G values of −7.20 kcal·mol^−1^ and −7.63 kcal·mol^−1^, respectively, suggesting the hydroxyl group denoted as “1” is the dominant deprotonation site under biologically relevant conditions. In diethyl ether, however, Bortezomib showed a small positive ∆∆G value of 0.53 kcal·mol^−1^, indicating a weak preference for pathway 2. Because this value is small and lies within the expected uncertainty of the computational approach, it should not be overinterpreted.

Conversely, Ixazomib and Delanzomib and the warhead model demonstrated significantly smaller ∆∆G values (ranging from −2.06 to −1.30 kcal·mol^−1^). The observed differences are within the expected uncertainty limits of the chosen DFT protocol (±2 kcal·mol^−1^), suggesting that both deprotonation modes are energetically comparable for the studied inhibitors and may coexist under physiologically relevant conditions. This differs for Bortezomib, which exhibits a clear thermodynamic preference for OH_1_ deprotonation. Moreover, the similarity of the observed patterns across polar environments supports the validity of the comparative analysis and the conclusion that warhead models capture the trends of deprotonation behavior in the studied molecules.

The comparison between the two possible proton-transfer pathways underscores an important chemical feature of the BABPIs: the two hydroxyl groups of the boronic acid warhead should not be considered entirely interchangeable during deprotonation. Previous studies of BABPIs have mainly focused on the formation of tetrahedral boronate adducts. Conversely, the present study directly compares the relative thermodynamic stability of the two possible monoanionic boronate products formed after OH_1_ or OH_2_ deprotonation across the studied BABPIs and the simplified warhead model. The pronounced OH_1_ preference observed for Bortezomib, compared with the weaker preferences calculated for Ixazomib, Delanzomib, and the simplified warhead model, suggests that the molecular scaffold can influence the stabilization of the deprotonated boronate form. This finding provides a more detailed thermodynamic view of the boronic acid warhead and may be useful for future drug-design studies. From a pharmaceutical perspective, understanding deprotonation preferences may support the rational design of new inhibitors with improved warhead reactivity and selectivity. Experimental confirmation of this preference would be challenging; therefore, future spectroscopic studies, isotope-labeling experiments, and theoretical approaches such as QM/MM calculations/simulations of enzyme-inhibitor complexes would be needed to evaluate the studied trends.

### 2.5. Study Limitations

The present study has several limitations. First, this DFT study was performed using a simplified model in which the catalytic residue was represented by an isolated deprotonated Thr1-O^−^ species. Therefore, the full proteasome active-site environment, including neighboring amino acid residues, hydrogen-bonding networks, and water molecules, was not included. These factors may influence the stabilization of charged species, the orientation of the boronic acid warhead, and the relative preference between OH_1_ and OH_2_ deprotonation in the real enzyme environment.

Second, solvent effects were described using PCM continuum models with three dielectric constants, corresponding to low-, intermediate-, and high-polarity environments. Although this technique allows comparison of the effect of the medium on the calculated thermodynamic trends, it cannot fully reproduce the heterogeneous environment of the proteasome active site and does not account for explicit hydrogen-bond networks or water-mediated proton transfer.

Third, the present study was focused on thermodynamic feasibility and product stability. Transition states, activation barriers, and full reaction coordinates were not calculated. Additionally, molecular docking and molecular dynamics calculations of the enzyme-inhibitor complexes were not included and remain important directions for future research.

Finally, the reported ∆G and ∆∆G values should be interpreted as comparative thermodynamic trends under simplified conditions, while a more complete study will require explicit active-site models, kinetic calculations, and QM/MM, and MD calculations/simulations.

## 3. Materials and Methods

### 3.1. Computational Methodology

The Gaussian 16 software package was used for all calculations in this study [33]. Visualization and analysis of molecular structures were carried out with ChemCraft version 1.8.3 [34]. The B3LYP functional combined with the 6-311+G(d,p) basis set was employed [35,36,37]. First, each molecule was optimized in the gas phase and, subsequently, vibrational frequency analysis was performed to verify the absence of imaginary frequencies, thus confirming that the studied molecules correspond to a true local minimum of the potential energy surface. Second, Gibbs free energies were obtained by combining electronic energies and thermochemical corrections generated from the calculations of Gibbs free energies.

To validate the accuracy of the chosen computational protocol, two complementary validation approaches were applied. First, representative bond lengths obtained from the DFT-optimized geometries of Bortezomib and Ixazomib were compared with available experimental data [14,32]. The geometries were optimized in polar media using the PCM approach to assess the reliability of the B3LYP/6-311+G(d,p) level of theory for studying structural characteristics of the BABPIs and for subsequent thermodynamic analysis. The detailed comparison is summarized in Table 2. The deviations between calculated and experimental bond lengths were small, typically within 0.01–0.03 Å, supporting the accuracy of the chosen computational protocol. Second, additional benchmark calculations were performed for both Bortezomib deprotonation pathways using the 3-21G*, 6-31+G(d,p), 6-311+G(d,p), and B3LYP/6-311++G(d,p) basis sets, as well as the B3LYP and M06-2X functionals. The corresponding results are presented in Tables S1 and S2 of the Supplementary Materials. Data obtained demonstrated that the free energies of modeled reactions converge with increasing the complexity of the basis set. At the same time, B3LYP and M06-2X functionals produced similar results. Thus, the B3LYP method in combination with 6-311+G(d,p) basis set was used with confidence throughout the paper.

To systematically evaluate the influence of solvation on deprotonation preferences, all reactions were modeled both in the gas phase and in media of different polarity. Solvent effects were considered using PCM calculations in diethyl ether (ε ≈ 4; low polarity, mimicking buried active sites), methanol (ε = 33; intermediate polarity/representing solvent-accessible binding pockets), and water (ε = 78; high polarity/imitating fully solvent-exposed active centers). These media were selected not as exact depictions of specific proteasome active site regions, but as simplified continuum dielectric environments combining low, intermediate, and high polarity regions. For this reason, the PCM results should be interpreted as comparative thermodynamic trends, since this approach does not explicitly reproduce the heterogeneous protein site.

### 3.2. Modeling of the Deprotonation Reactions

To assess the thermodynamic feasibility of a potential rearrangement of the tetrahedral boronate complex, we modeled a simplified proton-transfer reaction between a BABPI and a deprotonated threonine residue, represented as Thr1-O^−^. In this model, a proton is transferred from one of the boronic hydroxyl groups of the boronic acid warhead to the deprotonated threonine, resulting in a neutral Thr-OH and a monoanionic boronate species. Because the two hydroxyl groups attached to the boron atom are chemically non-equivalent in the optimized geometries, the proton-transfer reaction was evaluated for both deprotonation pathways, as shown in Equations (1) and (2).

R-B(OH_1_)(OH_2_) + Thr1-O^−^ → R-B(O_1_^−^)(OH_2_) + Thr1-OH (1)

R-B(OH_1_)(OH_2_) + Thr1-O^−^ → R-B(OH_1_)(O_2_^−^) + Thr1-OH (2)

Note that Equation (1) corresponds to pathway 1, where the OH_1_ group is deprotonated, resulting in the formation of monoanionic boronate species—R-B(O_1_^−^)(OH_2_). Equation (2) corresponds to pathway 2, where the OH_2_ group is deprotonated, resulting in the formation of monoanionic boronate species R-B(OH_1_)(O_2_^−^).These two pathways were calculated for Bortezomib, Ixazomib, Delanzomib, and the simplified warhead model.

Free energies in solution were determined by including solvation contributions to the gas-phase energies. The solvation energy (∆E^ε^_solv_) was found as the difference between electronic energies computed in polar media using the PCM model for the studied molecules and the corresponding values, as per the following Equation (3):

∆E^ε^_solv_ ≈ E_el_^2^ − E_el_^1^ (3)

For each reaction, the Gibbs free energy change (∆G^x^) was calculated as follows:

∆G^x^ = [G^x^[R-B(OH)(O_1,2_)]^−^ + G^x^[Thr-OH] − G^x^[R-B(OH_1_)(OH_2_)] − G^x^[Thr1-O^−^], (4)

where x is the dielectric constant of the studied environment.

More negative ∆G^x^ suggests a more thermodynamically favorable proton-transfer process, thus indicating a favored deprotonation site on the boronic acid warhead. The preference between two rival pathways was further evaluated using the energy difference ∆∆G^x^ = ∆G^x^(OH_1_) − ∆G^x^(OH_2_), where negative values indicate preference for OH_1_ deprotonation over OH_2_. These results allow comparative analysis of the behavior of BABPIs and clarify the preferences governing the boronic hydroxyl group deprotonation. Note that this study examines the thermodynamic feasibility of the proposed transformation, excluding specific transition states or kinetic barriers, aiming to recognize the general trends rather than reproduce absolute values. Supplementary Materials contain the Cartesian coordinates of all optimized molecular structures.

## 4. Conclusions

Proteasome inhibition is now a validated therapeutic approach in cancer treatment, with several proteasome inhibitors approved by the FDA and EMA for clinical use. The importance of BABPIs within this class arises from the ability of their boronic acid warhead to form a tetrahedral boronate complex with the proteasome’s catalytic Thr1 residue. Although the formation of this complex is a crucial step in proteasome inhibition, the possible deprotonation behavior of the boronic acid warhead remains unexplored.

Understanding whether such a transformation, resulting in Thr1-OH and a monoanionic boronate species, is thermodynamically possible, is important, as it may provide insight into the chemical behavior of boronic acid-based proteasome inhibitors.

In this DFT-based study, we showed that proton transfer from BABPIs to deprotonated Thr1-O^−^ is thermodynamically favorable across all studied polar environments—diethyl ether, methanol, and water. These media were selected as environments representing different polarity conditions, rather than as exact models of specific proteasome active-site regions. Therefore, the calculated values should be interpreted as comparative thermodynamic trends, rather than as exact models of specific proteasome active-site regions.

This thermodynamic analysis suggests that boronic hydroxyl groups are capable of deprotonation under physiologically relevant conditions, with a reactivity trend favoring the deprotonation pathway involving hydroxyl group 1 (OH_1_), which remains preferred across all studied inhibitors regardless of solvent polarity.

However, the comparison of energy differences between the two deprotonation pathways revealed that Bortezomib exhibited a clear thermodynamic preference for reaction pathway 1, favoring OH_1_ deprotonation, with ∆∆G values exceeding 7 kcal·mol^−1^ in both methanol and water. Conversly, Ixazomib, Delanzomib, and the simplified warhead model displayed minor energy differences between the formation of monoanionic boronate species, with ∆∆G values ranging from 1.3 to 2.06 kcal·mol^−1^, which align with the expected uncertainty of the DFT approach (±2 kcal·mol^−1^). Moreover, these results suggest that both deprotonation pathways may be energetically possible and potentially coexist depending on the solvent environment.

The observed preference for OH_1_ deprotonation pathway is mostly governed by the structural factors, including the local geometry of the boronic acid warhead, solvent stabilization, hydrogen-bonding, and stabilization of the deprotonated species. However, the present study does not provide a quantitative decomposition of these effects.

Overall, this comparative DFT analysis sheds more light on the chemical non-equivalence of the two hydroxyl groups of the boronic acid warhead and suggests that the molecular scaffold can influence the deprotonation pathway. These findings offer a thermodynamic framework for the design of the next generation of BABPIs.

## Figures and Tables

**Table 1:** Overview of the studied BABPIs, with emphasis on subunit selectivity, therapeutic implications, and current clinical status.

Drug (Trademark)	Development Code	Structure	Subunit Selectivity	Therapeutic Implications	Clinical Status
**Bortezomib**	PS-341	Pyrazin-2-carboxyl-Phe-*boroLeu*	β5, β1	Treatment of multiple myeloma (MM) and mantle-cell lymphoma (MCL)	FDA/EMA-approved [20,21]
**Ixazomib**	MLN9708	2,5-dichlorobenzoyl-Gly-*boroLeu*	β5, β1, β2	Treatment of MM	FDA/EMA-approved [22,23,24,25]
**Delanzomib**	CEP-18770	6-phenylpyridine-2-carbonyl-Thr-*boroLeu*	β5, β1	Treatment of MM	Not approved by drug regulators [26,27,28]

**Table 2:** Comparison of experimental and DFT-calculated mean bond lengths (in Å) for Bortezomib and Ixazomib in polar media using PCM at the B3LYP/6-311+g(d,p) level of theory.

Bond Type	Calculated Average Bond Lengths			Experimental Average Bond Lengths			Difference in Average Values
	Bortezomib	Ixazomib	Average	Bortezomib	Ixazomib	Average	
B-O^1^	1.370	1.370	1.370	1.430	1.440	1.435	0.065
B-O^2^	1.370	1.370	1.370	1.420	1.450	1.435	0.065
B-C	1.590	1.600	1.595	1.580	1.590	1.585	0.010
C=O	1.230	1.230	1.230	1.230	1.240	1.235	0.005
N-C	1.350	1.340	1.340	1.340	1.340	1.340	0.000

**Table 3:** Relative solvation Gibbs free energies (∆G, kcal·mol^−1^) of the studied BABPIs and the simplified warhead model at the B3LYP/6-311+G(d,p) level of theory in the gas phase (∆G^1^), diethyl ether (∆G^4^), methanol (∆G^33^), and water (∆G^78^), referenced to the gas-phase free energy (∆G^1^).

Proteasome Inhibitor	ΔG^1^	ΔG^4^	ΔG^33^	ΔG^78^
Bortezomib	0.0	−11.68	−17.21	−18.01
Ixazomib	0.0	−9.18	−14.81	−15.67
Delanzomib	0.0	−10.03	−15.35	−15.96
Warhead model	0.0	−7.77	−11.58	−12.07

**Table 4:** Calculated Gibbs free energy changes (∆G, kcal·mol^−1^) for a reaction of proton transfer from boronic hydroxyl group 1 [OH_1_] of the studied BABPIs and the simplified warhead model to the deprotonated threonine residue (Thr1-O^−^), resulting in the formation of the monoanionic boronate species and Thr1-OH. Energies were obtained using DFT calculations at the B3LYP/6-311+G(d,p) level of theory in different media: gas phase (∆G^1^), diethyl ether (∆G^4^), methanol (∆G^33^), and water (∆G^78^).

Modeled OH_1_ Proton-Transfer Reaction	ΔG^1^	ΔG^4^	ΔG^33^	ΔG^78^
Bortezomib-OH_1_ + Thr1-O^−^ → Bortezomib-O_1_^−^ + Thr1-OH	−32.66	−16.66	−22.48	−22.61
Ixazomib-OH_1_ + Thr1-O^−^ → Ixazomib-O_1_^−^ + Thr1-OH	−35.27	−22.22	−17.80	−17.38
Delanzomib-OH_1_ + Thr1-O^−^ → Delanzomib-O_1_^−^ + Thr1-OH	−34.38	−17.14	−16.16	−15.66
Warhead model-OH_1_ + Thr1-O^−^ → Warhead model-O_1_^−^ + Thr1-OH	−27.61	−19.16	−16.01	−15.53

**Table 5:** Calculated Gibbs free energy changes (∆G, kcal·mol^−1^) for a reaction of proton transfer from boronic hydroxyl group 2 [OH_2_] of the studied BABPIs and the simplified warhead model to the deprotonated threonine residue (Thr1-O^−^), resulting in the formation of the monoanionic boronate species and Thr1-OH. Energies were obtained using DFT calculations at the B3LYP/6-311+G(d,p) level of theory in different media: gas phase (∆G^1^), diethyl ether (∆G^4^), methanol (∆G^33^), and water (∆G^78^).

Modeled OH_2_ Proton-Transfer Reaction	ΔG^1^	ΔG^4^	ΔG^33^	ΔG^78^
Bortezomib-OH_2_ + Thr1-O^−^ → Bortezomib-O_2_^−^ + Thr1-OH	−28.86	−17.19	−15.28	−14.98
Ixazomib-OH_2_ + Thr1-O^−^ → Ixazomib-O_2_^−^ + Thr1-OH	−31.88	−19.29	−16.31	−16.08
Delanzomib-OH_2_ + Thr1-O^−^ →Delanzomib-O_2_^−^ + Thr1-OH	−30.52	−15.84	−14.10	−13.93
Warhead model-OH_2_ + Thr1-O^−^ → Warhead model-O_2_^−^ + Thr1-OH	−25.45	−16.66	−14.45	−14.01

**Table 6:** Gibbs free energy differences ∆∆G^x^ = ∆G^x^ (OH_1_) − ∆G^x^ (OH_2_), in kcal·mol^−1^ between the two deprotonation pathways of the studied BABPIs and a warhead model, calculated using DFT at the B3LYP/6-311+G(d,p) level of theory. Negative values indicate a preference for pathway 1 (OH_1_), while positive values indicate a preference for pathway 2 (OH_2_). The ∆∆G range summarizes the minimum and maximum energy differences observed across the media studied.

Studied System	∆∆G^4^	∆∆G^33^	∆∆G^78^	∆∆G_range_
Bortezomib	0.53	−7.20	−7.63	−7.63 to 0.53
Ixazomib	−2.93	−1.49	−1.30	−2.93 to −1.30
Delanzomib	−1.30	−2.06	−1.73	−2.06 to −1.30
Warhead model	−2.50	−1.56	−1.52	−2.50 to −1.52

## Data Availability

The data presented in this study are available on request from the corresponding authors.

## Supplementary Materials

The following supporting information can be downloaded at: https://www.mdpi.com/article/10.3390/ph19081251/s1.

## Abbreviations

The following abbreviations are used in this manuscript:

BABPIs	Boronicacid-based proteasome inhibitors
B-Leu	Boron-leucine complex (P1 site warhead moiety)
B3LYP	Becke, 3-parameter, Lee-Yang-Parr (Density Functional Theory exchange-correlation functional)
Bid, Bim, Bax	BH3-only domain pro-apoptotic proteins
∆G^1^	Change in Gibbs Free Energy in gas phase (ε = 1)
∆G^4^	Change in Gibbs Free Energy in diethyl ether (ε = 4)
∆G^33^	Change in Gibbs Free Energy in Methanol (ε = 33)
∆G^78^	Change in Gibbs Free Energy in Water (ε = 78)
DFT	Density Functional Theory
EMA	European Medicines Agency
ER	Endoplasmic Reticulum
FDA	Food and Drug Administration
Gly	Glycine
HBA	Hydrogen Bond Acceptor
HBD	Hydrogen Bond Donor
HY	Hydrophobic group/region
JNK	c-Jun NH2-terminal kinase
NF-κB	nuclear factor kappa-light-chain-enhancer of activated B cells
MCL	Mantle-Cell Lymphoma
MM	Multiple Myeloma
PCM	Polarizable Continuum Model
Phe	Phenylalanine
PIs	Proteasome Inhibitors
Pyz	Pyrazin-2-carboxyl (N-terminal capping group)
P2	P2 Area of the Inhibitor Molecule
P3	P3 Area of the Inhibitor Molecule
Thr1	N-terminal catalytic threonine residue at position 1
Thr1-O^−^	Deprotonated catalytic threonine residue
Thr1-OH	Neutral/protonated catalytic threonine residue
UPR	Unfolded Protein Response
