# Supplementary material for: Thermodynamic Preference Between Deprotonation Pathways in Boronic Acid-Based Proteasome Inhibitors: Insights from a DFT Study
Source: Pharmaceuticals (Basel). 2026 Aug 8;19(8):1251. doi: 10.3390/ph19081251 (PMC13516238; doi:10.3390/ph19081251)
Supplement: Supplementary file 1 [file pharmaceuticals-19-01251-s001.zip › pharmaceuticals-4452122-supplementary.pdf]

Supplementary Information

Thermodynamic Preference Between Deprotonation Pathways in Boronic Acid-Based Proteasome Inhibitors: Insights from a DFT Study

Nikolay Toshev [1,*], Iliyan Dimitrov [2], Ovanes Muradyan [3], Vassil Delchev [4] and Todor Dudev [5]

1 Department of Bioorganic Chemistry, Faculty of Pharmacy, Medical University of Plovdiv, 15A Vassil Aprilov Blvd., 4002 Plovdiv, Bulgaria; nikolay.toshev@mu-plovdiv.bg
2 Department of Medical Biochemistry, Faculty of Pharmacy, Medical University of Plovdiv, 15A Vassil Aprilov Blvd., 4002 Plovdiv, Bulgaria; iliyan.dimitrov@mu-plovdiv.bg
3 Medical Faculty, Medical University of Plovdiv, 15A Vassil Aprilov Blvd., 4002 Plovdiv, Bulgaria; ovanes.m301204@gmail.com
4 Department of Physical Chemistry, Faculty of Chemistry, Plovdiv University "Paisii Hilendarski", 24 Tsar Asen Str., 4000 Plovdiv, Bulgaria; vdelchev@uni-plovdiv.net
5 Department of Pharmaceutical and Applied Organic Chemistry, Faculty of Chemistry and Pharmacy, Sofia University "St. Kliment Ohridski", 1 James Bourchier Blvd., 1164 Sofia, Bulgaria, ohtttd@chem.uni-sofia.bg
* Correspondence: nikolay.toshev@mu-plovdiv.bg

Table of Contents

1. Supplementary benchmark calculations: Tables S1- S2
2. Cartesian coordinates of the optimized geometries.
3. Frequency & IR Intensity

1. Supplementary benchmark calculations: Tables S1- S2

Table S1. Dependence of the calculated relative Gibbs free energy changes (ΔG, kcal.mol⁻¹) for the two deprotonation pathways of Bortezomib on the basis set in the gas phase (ΔG^1), diethyl ether (ΔG^4), methanol (ΔG^{33}), and water (ΔG^{78}).

Bortezomib-OH_1 +Thr1-O^-→Bortezomib-O_1^-+Thr-OH

B3LYP	ΔG^1	ΔG^4	ΔG^{33}	ΔG^{78}
**3-21G***	-38.60	-38.97	-43.46	-41.63
**6-31+G(d,p)**	-33.59	-17.41	-22.75	-23.11
**6-311+G(d,p) (selected)**	-32.66	-16.66	-22.48	-22.61
**6-311++G(d,p)**	-32.33	-16.71	-22.65	-21.91

Bortezomib-OH_2 +Thr1-O^-→Bortezomib-O_2^-+Thr1-OH

B3LYP	ΔG^1	ΔG^4	ΔG^{33}	ΔG^{78}
**3-21G***	-34.44	-38.47	-32.00	-30.38
**6-31+G(d,p)**	-29.77	-18.26	-15.48	-15.13
**6-311+G(d,p) (selected)**	-28.86	-17.19	-15.28	-14.98
**6-311++G(d,p)**	-28.57	-17.35	-15.21	-14.86

Table S2. Dependence of the calculated relative Gibbs free energy changes (ΔG, kcal.mol⁻¹) for the two deprotonation pathways of Bortezomib on the functional in the gas phase (ΔG^1), diethyl ether (ΔG^4), methanol (ΔG^{33}), and water (ΔG^{78}).

Bortezomib-OH_1 +Thr1-O^-→Bortezomib-O_1^-+Thr1-OH

Functional/basis set	ΔG^1	ΔG^4	ΔG^{33}	ΔG^{78}
**B3LYP/6-311+G(d,p) (selected)**	-32.66	-16.66	-22.48	-22.61
**M062X/6-311+G(d,p)**	-37.40	-25.61	-23.03	-22.24

Bortezomib-OH_2 +Thr1-O^-→Bortezomib-O_2^-+Thr1-OH

**Functional/basis set**	**ΔG[1]**	**ΔG[4]**	**ΔG[33]**	**ΔG[78]**
**B3LYP/6-311+G(d,p) (selected)**	-28.86	-17.19	-15.28	-14.98
**M062X/6-311+G(d,p)**	-36.23	-20.08	-15.61	-15.69

2. Cartesian coordinates of the optimized geometries

Level of theory B3LYP/6-311+G(d,p)

Bortezomib in gas phase

O	0.55081000	0.57077300	-1.64217300
O	-1.01230100	-4.43194400	1.11379200
O	-0.23696900	-3.89429400	-1.11880200
O	3.09078000	-0.00833600	2.79946400
N	-0.51657300	-1.16797300	-0.68210600
N	1.10818200	0.08285500	1.71270000
N	2.87873100	-0.94874500	-0.46202200
N	5.15264800	0.65078100	-0.81447900
C	-2.47316100	-1.69366200	0.83011500
C	-3.53680100	-1.70091300	-0.28438900
C	-1.00773200	-2.01035700	0.41393900
C	0.20538000	0.74465900	0.74325000
C	0.11407200	0.03305600	-0.63282200
C	0.51207800	2.25345400	0.57785600
C	-3.77401400	-3.09245500	-0.88874300
C	-4.85432700	-1.12079000	0.25145300
C	-0.68957600	3.07209800	0.14995300
C	-0.97335500	3.31075100	-1.19976000
C	-1.55160700	3.60539000	1.11683800
C	2.48261000	0.03177300	1.73919900
C	-2.08865200	4.06128700	-1.56885700

C	-2.66900000	4.35315400	0.75013400
C	-2.94114500	4.58330100	-0.59735900
C	3.25352700	-0.05015900	0.44839200
C	4.39826000	0.73816000	0.27918700
C	3.64613000	-1.04892000	-1.54783000
C	4.77170900	-0.24819200	-1.72675100
B	-0.74011700	-3.54523800	0.10732100
H	-2.75865100	-2.40466600	1.61444500
H	-2.49173200	-0.70271000	1.29803100
H	-3.18348700	-1.03916700	-1.08421100
H	-0.38471700	-1.81399800	1.29315500
H	-0.78030800	0.65517800	1.20399300
H	-0.45364300	-1.59968600	-1.59504700
H	0.86278700	2.62032900	1.54656700
H	1.32575800	2.36860600	-0.14024800
H	-4.08324000	-3.80766600	-0.11866800
H	-4.56579900	-3.05407200	-1.64260500
H	-2.88369000	-3.48872900	-1.38388300
H	-4.71650500	-0.10600800	0.63545400
H	-5.61431200	-1.07936600	-0.53410500
H	-5.25210800	-1.73622700	1.06621400
H	0.76330900	0.12061700	2.66506800
H	-0.32129000	2.89217100	-1.95656800
H	-1.33850100	3.44677300	2.17024000
H	-0.83710300	-5.35986900	0.93222600
H	-0.04745300	-4.82603800	-1.26305500
H	-2.29075500	4.23900600	-2.61951200
H	-3.31971000	4.76238500	1.51522100
H	-3.80664700	5.16881000	-0.88690300
H	4.69894300	1.44557100	1.04442900
H	3.35283200	-1.77927600	-2.29517600
H	5.38076400	-0.33360500	-2.62140200

Bortezomib in diethyl ether

O	0.73888800	0.55855400	-1.48833200
O	-1.22626100	-4.38131800	1.18759400
O	-0.14422800	-3.91138400	-0.93043300

O	3.22363200	-0.43315200	2.71827100
N	-0.39465400	-1.17466700	-0.59159700
N	1.21818000	0.07026100	1.81071600
N	3.21643400	-1.12801300	-0.38625300
N	4.70576300	1.13794700	-1.09020500
C	-2.53099300	-1.59127400	0.68699100
C	-3.46295200	-1.61680700	-0.53978900
C	-1.04194800	-1.97486300	0.45724700
C	0.26870800	0.71337300	0.87946900
C	0.22822000	0.02234000	-0.50757700
C	0.49522900	2.23853300	0.75843400
C	-3.68974500	-3.02649400	-1.10459700
C	-4.80711500	-0.96540500	-0.18051700
C	-0.73489200	2.99506600	0.29841600
C	-0.94967400	3.29201400	-1.05252700
C	-1.69159400	3.41282700	1.23301100
C	2.56057100	-0.13531800	1.72876300
C	-2.09054200	3.98492000	-1.45726500
C	-2.83433800	4.10311000	0.83159200
C	-3.03777300	4.39093300	-0.51788000
C	3.25708600	-0.04288700	0.38943000
C	4.01019000	1.08266900	0.04591100
C	3.91770700	-1.07174200	-1.51986000
C	4.65302400	0.05777400	-1.87430100
B	-0.79293600	-3.52812100	0.21071600
H	-2.92945100	-2.25620800	1.46211500
H	-2.56380100	-0.57973600	1.10715000
H	-2.99927800	-1.00690000	-1.32460900
H	-0.52043700	-1.77763600	1.39987700
H	-0.70362800	0.55883200	1.34793700
H	-0.30751900	-1.60630700	-1.50308000
H	0.78599100	2.59796900	1.74914400
H	1.32764200	2.41977600	0.07732200
H	-4.11963400	-3.68844000	-0.34514800
H	-4.38472100	-2.99326700	-1.94875700
H	-2.76690200	-3.48392000	-1.47043100
H	-4.67124900	0.06111000	0.17179300
H	-5.47346000	-0.93689600	-1.04747200
H	-5.31490700	-1.52771000	0.61121600
H	0.88739100	-0.01014300	2.76531000
H	-0.22110800	2.96877300	-1.78627600
H	-1.53500300	3.20738800	2.28778300
H	-1.06729000	-5.31908300	1.03559700
H	0.03328400	-4.85264500	-1.03217300
H	-2.23806500	4.20860100	-2.50818000
H	-3.55982100	4.42213500	1.57189700
H	-3.92346600	4.93112700	-0.83314200
H	4.05230400	1.94826200	0.69912600
H	3.89430100	-1.94592900	-2.16209100
H	5.21219200	0.08853100	-2.80361700

Bortezomib in methanol

O	0.72847900	0.60103500	-1.45702300
O	-1.22395400	-4.38634400	1.16744900
O	-0.04111400	-3.89857400	-0.89226200
O	3.19894500	-0.67501000	2.64461500
N	-0.36551500	-1.16631100	-0.57876900
N	1.22853700	0.06348900	1.82866400
N	3.34341600	-1.11323300	-0.40984600
N	4.57970100	1.31063400	-1.07103000
C	-2.53773200	-1.61066100	0.62319900
C	-3.42539900	-1.65484000	-0.63576800
C	-1.03719400	-1.97674300	0.44856900
C	0.26529900	0.71222200	0.91609600
C	0.22925200	0.04193900	-0.48026700
C	0.46589800	2.24177400	0.82474600
C	-3.62069600	-3.07079300	-1.19641700
C	-4.78700900	-1.01227900	-0.32968600
C	-0.77318600	2.98395800	0.36540700
C	-0.91570100	3.41799300	-0.95737000
C	-1.80736100	3.25562500	1.27146600
C	2.54519400	-0.23027100	1.70159100
C	-2.06018600	4.10423000	-1.36496700
C	-2.95393700	3.93806600	0.86731300
C	-3.08413600	4.36497700	-0.45475600
C	3.24391400	-0.03591100	0.37320800
C	3.86983300	1.17032100	0.04998900
C	4.04937000	-0.96807000	-1.53293600
C	4.66134600	0.23970600	-1.86515600
B	-0.75509200	-3.52736900	0.21308500
H	-2.95515300	-2.27665000	1.38714500
H	-2.59628300	-0.59812800	1.03765400
H	-2.94060900	-1.04712400	-1.40972600
H	-0.55362200	-1.76861400	1.40840700
H	-0.70123200	0.53451600	1.38747000
H	-0.28826700	-1.57723300	-1.50088900
H	0.74129600	2.58592300	1.82509600
H	1.29986400	2.45463200	0.15578000
H	-4.07886400	-3.72784300	-0.44947900
H	-4.27955900	-3.04874400	-2.06944500
H	-2.68131000	-3.52765500	-1.51815300
H	-4.67230300	0.01795200	0.01953000
H	-5.42222300	-0.99694200	-1.22008700
H	-5.31717400	-1.57300400	0.44828900
H	0.89476300	-0.07448000	2.77571700
H	-0.12651100	3.21017700	-1.67038600
H	-1.71071800	2.93992500	2.30588200
H	-1.04032600	-5.32144100	1.02318900
H	0.13214500	-4.84137600	-0.99519400
H	-2.15063800	4.43558600	-2.39366800
H	-3.74080900	4.14222600	1.58506700
H	-3.97314000	4.89928100	-0.77062400
H	3.80275200	2.03062900	0.70764500
H	4.13492600	-1.83240300	-2.18283100

H	5.23121600	0.33926800	-2.78275000

Bortezomib in water

O	0.72553000	0.60645700	-1.45467900
O	-1.21921400	-4.38512300	1.17016700
O	-0.02144000	-3.89573000	-0.88054800
O	3.19901100	-0.68303200	2.63879900
N	-0.36284300	-1.16441600	-0.57662300
N	1.22963900	0.06620400	1.83006700
N	3.34884000	-1.10919200	-0.41221900
N	4.57061800	1.32194100	-1.07300100
C	-2.53832200	-1.61467100	0.61620800
C	-3.42002200	-1.66548100	-0.64673500
C	-1.03578300	-1.97580600	0.44933400
C	0.26429000	0.71423400	0.91927600
C	0.22841100	0.04534500	-0.47766100
C	0.46125700	2.24425500	0.83000700
C	-3.60764900	-3.08356700	-1.20461300
C	-4.78534500	-1.02708300	-0.34852200
C	-0.77947300	2.98393400	0.37100800
C	-0.91516000	3.43366600	-0.94721500
C	-1.82183200	3.23797200	1.27285600
C	2.54457800	-0.23150900	1.69912000
C	-2.06080600	4.11815400	-1.35472200
C	-2.96961300	3.91844100	0.86878200
C	-3.09294800	4.36112500	-0.44882500
C	3.24222300	-0.03238400	0.37081800
C	3.86096200	1.17759100	0.04775400
C	4.05421500	-0.95989400	-1.53517700
C	4.65913400	0.25155200	-1.86715100
B	-0.74620300	-3.52580600	0.21825100
H	-2.95684900	-2.28011600	1.37997900
H	-2.60209400	-0.60130000	1.02779800
H	-2.93403700	-1.05799900	-1.42017500
H	-0.55752900	-1.76364100	1.41088000
H	-0.70135600	0.53380400	1.39131800
H	-0.28708500	-1.57350600	-1.49969700
H	0.73539400	2.58754500	1.83098900
H	1.29492500	2.46051400	0.16182600
H	-4.06748800	-3.74012800	-0.45830000
H	-4.26204000	-3.06600800	-2.08110500
H	-2.66515100	-3.53832400	-1.52021700
H	-4.67595600	0.00434500	-0.00109700
H	-5.41646800	-1.01619000	-1.24190300
H	-5.31701200	-1.58789200	0.42834700
H	0.89667800	-0.07391100	2.77712700
H	-0.11947500	3.24012700	-1.65708300
H	-1.73094900	2.90978000	2.30386900
H	-1.03074500	-5.31972900	1.02858100
H	0.15350200	-4.83850400	-0.98185100
H	-2.14579200	4.46189400	-2.37982200

H	-3.76297500	4.10861000	1.58321100
H	-3.98296400	4.89387200	-0.76445700
H	3.78857900	2.03753000	0.70525900
H	4.14541100	-1.82364400	-2.18504100
H	5.22889600	0.35432600	-2.78442300

Deprotonated Bortezomib (de1) in gas phase

O	0.79207400	1.08507300	-1.53056800
O	0.44313600	-3.96819100	1.47028600
O	1.16807700	-3.28708300	-0.74470000
O	3.39840300	2.84026700	1.58120700
N	0.02011200	-0.77485400	-0.47229600
N	1.36225500	1.90448000	1.38238800
N	2.89257000	-0.34347000	0.27969200
N	5.09278900	0.36256400	-1.30074400
C	-1.70770000	-1.75824100	1.07867000
C	-2.69276000	-2.19598800	-0.02536100
C	-0.21406700	-1.65059600	0.69482100
C	0.13300200	1.37227400	0.74999100
C	0.36817000	0.52043700	-0.51633600
C	-0.73604300	2.60564000	0.37426600
C	-2.45306000	-3.62497200	-0.53364900
C	-4.13796200	-2.04634500	0.47583300
C	-2.15262100	2.31299900	-0.06765900
C	-2.43926300	1.97344700	-1.39629900
C	-3.21530200	2.40477900	0.83858600
C	2.69829000	1.95532800	1.08705900
C	-3.75076300	1.73599700	-1.80202800
C	-4.52876300	2.16912600	0.43510900
C	-4.80092300	1.83480000	-0.88989000
C	3.34662400	0.90640900	0.23127600
C	4.45330400	1.25856500	-0.55005000
C	3.51515400	-1.24747400	-0.48428000
C	4.61151400	-0.88574000	-1.26654800
B	0.54277100	-3.06399400	0.36866200
H	-1.77121800	-2.46474300	1.91544000
H	-2.06337100	-0.78966600	1.46098300
H	-2.56264400	-1.51021600	-0.87171900
H	0.31680700	-1.22964300	1.55558600
H	-0.38846000	0.77844400	1.50143300
H	0.34645500	-1.31850100	-1.27354600
H	-0.77663300	3.26325900	1.25016700
H	-0.19794100	3.13581600	-0.41471900
H	-2.54188300	-4.34837900	0.28450500
H	-3.19460300	-3.88695900	-1.29784500
H	-1.46051900	-3.74347300	-0.97254300
H	-4.35010800	-1.01866500	0.78526000
H	-4.85797300	-2.31703200	-0.30453900
H	-4.31989300	-2.70265600	1.33588400
H	1.15473400	2.66205600	2.02260600
H	-1.62280000	1.88678700	-2.10452400
H	-3.01231200	2.67063100	1.87195100

H	0.92083300	-4.76395100	1.21196700
H	-3.95193300	1.46928500	-2.83413400
H	-5.33687900	2.24547800	1.15507000
H	-5.82095800	1.64735800	-1.20764400
H	4.81309700	2.28105100	-0.55970100
H	3.08256000	-2.24677500	-0.50183500
H	5.11320300	-1.62378500	-1.88555800

Deprotonated Bortezomib (de1) in diethyl ether

O	0.84721000	-1.10124400	1.53686900
O	0.53805000	3.93229000	-1.43411900
O	1.05918200	3.34241000	0.85960400
O	3.41521300	-2.81505200	-1.61544300
N	0.04375600	0.75223600	0.50156200
N	1.35751300	-1.94035800	-1.36611600
N	2.96897600	0.36434400	-0.38325700
N	5.01951300	-0.38992700	1.36935100
C	-1.68300600	1.73908700	-1.05730300
C	-2.69707800	2.19889200	0.01093200
C	-0.19239400	1.64723200	-0.65098800
C	0.13844700	-1.38643300	-0.73446600
C	0.39591300	-0.54260800	0.52866700
C	-0.75565900	-2.60560600	-0.36949100
C	-2.51632900	3.66123700	0.44225300
C	-4.12871400	1.97811400	-0.50224200
C	-2.17000500	-2.29201700	0.06676000
C	-2.46683000	-1.99752800	1.40362800
C	-3.22278000	-2.33475000	-0.85538400
C	2.69105000	-1.95767400	-1.09959000
C	-3.77933800	-1.75329900	1.80518800
C	-4.53711000	-2.09392100	-0.45657200
C	-4.81959100	-1.80300400	0.87739800
C	3.33461000	-0.90848200	-0.23815100
C	4.36544200	-1.28496100	0.63099100
C	3.62164700	1.26464500	0.35839000
C	4.64119200	0.88621300	1.22901600
B	0.53302700	3.06575500	-0.29618600
H	-1.73907000	2.42066200	-1.91506000
H	-2.01895500	0.75907700	-1.42264100
H	-2.55927800	1.56411600	0.89520600
H	0.35019400	1.24093700	-1.51097500
H	-0.36962300	-0.78155200	-1.48539200
H	0.33436700	1.27627700	1.32618600
H	-0.80259300	-3.25070000	-1.25269700
H	-0.23397000	-3.15931400	0.41411100
H	-2.63994300	4.33502400	-0.41337800
H	-3.26627100	3.93419800	1.19259800
H	-1.53017800	3.84357000	0.87210000
H	-4.30625400	0.93018400	-0.75834800
H	-4.86735300	2.26902700	0.25187800
H	-4.31586200	2.58159500	-1.39866300
H	1.13817200	-2.68354200	-2.01996600

H	-1.66240500	-1.96199300	2.12985400
H	-3.01368400	-2.57202000	-1.89409700
H	0.97721100	4.75054300	-1.17610500
H	-3.98942900	-1.52826400	2.84511600
H	-5.33866500	-2.13760600	-1.18597900
H	-5.84079300	-1.61739600	1.19134400
H	4.65223200	-2.32603100	0.72645100
H	3.29299400	2.29515500	0.28208400
H	5.16168300	1.62714800	1.82701300

Deprotonated Bortezomib (de1) in methanol

O	-0.81616300	-0.15266500	3.13095800
O	0.71650100	4.22414800	-0.48000000
O	-0.47944100	4.09062100	1.62578500
O	3.94873200	-0.65387800	2.06329200
N	-0.70711300	1.33111200	1.42318600
N	1.71375800	-0.88875300	1.84322300
N	2.43201300	-1.56853800	-0.93821700
N	4.47595700	0.02379200	-1.99829000
C	-0.99880300	1.59728200	-1.06293500
C	-2.49448400	1.97833200	-1.06848000
C	-0.17270000	1.95667600	0.19687700
C	0.39345200	-0.88710300	1.19537100
C	-0.44890600	0.14099300	1.98241000
C	-0.22157600	-2.30418700	1.25117300
C	-2.73980700	3.48591700	-1.22713900
C	-3.22733600	1.20926600	-2.17870500
C	-1.52559500	-2.45964200	0.49856200
C	-2.75953400	-2.30597900	1.14280600
C	-1.52264600	-2.78341000	-0.86479300
C	2.95972700	-0.76302400	1.33250000
C	-3.95658700	-2.47198700	0.44555900
C	-2.71674900	-2.95160900	-1.56469700
C	-3.93934900	-2.79644800	-0.91075100
C	3.16749800	-0.76666000	-0.16254200
C	4.19228900	0.02482500	-0.69546800
C	2.73001900	-1.58398000	-2.23919000
C	3.74555900	-0.78922300	-2.76804200
B	-0.01655600	3.54411200	0.53781900
H	-0.50800300	2.06429800	-1.92563600
H	-0.93355700	0.51399900	-1.22722100
H	-2.92315600	1.66350600	-0.10851200
H	0.84070600	1.57527000	0.02743500
H	0.51741800	-0.58496200	0.16159400
H	-1.11426900	2.02072000	2.05321300
H	0.52457400	-2.98131700	0.83067000
H	-0.36150700	-2.56881300	2.30160700
H	-2.32945200	3.84616800	-2.17741500
H	-3.81308900	3.70228100	-1.22545400
H	-2.28336700	4.06407000	-0.42199400
H	-3.13148700	0.12799700	-2.04722000
H	-4.29409600	1.45433000	-2.18975700

H	-2.81858400	1.46558300	-3.16326600
H	1.69436200	-0.91488000	2.85714800
H	-2.78050600	-2.06023200	2.19889300
H	-0.57580900	-2.91587100	-1.37846300
H	0.79006100	5.15433900	-0.23688100
H	-4.90193900	-2.35324500	0.96374800
H	-2.69248800	-3.20837500	-2.61815300
H	-4.86911800	-2.93023900	-1.45231300
H	4.78095500	0.66657300	-0.05067100
H	2.14798300	-2.24126000	-2.87623300
H	3.97096900	-0.80983100	-3.82891000

Deprotonated Bortezomib (de1) in water

O	-0.81238400	-0.15478900	3.12979800
O	0.71644300	4.22176600	-0.48038900
O	-0.46680600	4.08567100	1.63200800
O	3.94958800	-0.65051400	2.06291200
N	-0.70810500	1.32701800	1.42054300
N	1.71550700	-0.89210000	1.84168300
N	2.43624200	-1.57157300	-0.93803500
N	4.47254200	0.02869000	-2.00061200
C	-1.00239500	1.59907400	-1.06487900
C	-2.49887000	1.97672200	-1.06624200
C	-0.17440400	1.95464600	0.19482500
C	0.39550500	-0.89010500	1.19326300
C	-0.44685900	0.13771000	1.98021500
C	-0.22037400	-2.30679800	1.24804500
C	-2.74820400	3.48508800	-1.21064600
C	-3.23010800	1.21632500	-2.18350700
C	-1.52564800	-2.45964900	0.49705800
C	-2.75841300	-2.31074900	1.14467800
C	-1.52508700	-2.77601700	-0.86805300
C	2.96124700	-0.76273100	1.33141000
C	-3.95684000	-2.47423100	0.44909800
C	-2.72052500	-2.94158800	-1.56633000
C	-3.94198100	-2.79124000	-0.90903700
C	3.16906100	-0.76612200	-0.16343600
C	4.18994800	0.02933900	-0.69744900
C	2.73328200	-1.58679100	-2.23913900
C	3.74517400	-0.78800500	-2.76929900
B	-0.01266200	3.54060900	0.53911000
H	-0.51425000	2.07064400	-1.92658200
H	-0.93493000	0.51672000	-1.23396100
H	-2.92656100	1.65189300	-0.10911600
H	0.83793500	1.57150400	0.02321200
H	0.51989600	-0.58755200	0.15962900
H	-1.11582500	2.01503800	2.05167200
H	0.52472000	-2.98380100	0.82552300
H	-0.35909500	-2.57303200	2.29821800
H	-2.33942800	3.85520200	-2.15777800
H	-3.82199300	3.69867500	-1.20642200
H	-2.29275500	4.05711200	-0.40046400

H	-3.13022900	0.13410000	-2.06325600
H	-4.29770200	1.45771500	-2.19108500
H	-2.82313800	1.48403100	-3.16571800
H	1.69570900	-0.92037500	2.85554100
H	-2.77747700	-2.07116300	2.20223300
H	-0.57928800	-2.90475000	-1.38454600
H	0.79495900	5.15120500	-0.23584900
H	-4.90125400	-2.35970300	0.96993600
H	-2.69815900	-3.19265500	-2.62117400
H	-4.87268300	-2.92326100	-1.44940000
H	4.77636400	0.67421400	-0.05368800
H	2.15360500	-2.24690500	-2.87531900
H	3.96981900	-0.80850400	-3.83029500

Deprotonated Bortezomib (de2) in gas phase

O	0.84562800	0.29917900	-1.47974500
O	-1.75126200	-4.52998000	0.96689400
O	-0.75411000	-3.89892900	-1.16243700
O	3.32990500	-0.24547100	2.78956900
N	-0.47181300	-1.29742200	-0.54511200
N	1.29898100	-0.03718800	1.81162000
N	3.19658100	-1.22351700	-0.32909700
N	4.92601200	0.85581500	-1.06705400
C	-2.54890200	-1.45367800	0.90017300
C	-3.52546100	-1.14750900	-0.25376700
C	-1.16755700	-2.04821400	0.52706200
C	0.33561400	0.56363200	0.86340800
C	0.25860600	-0.17629600	-0.50021400
C	0.60032500	2.07719300	0.67012700
C	-3.97692700	-2.39795200	-1.02190000
C	-4.74627400	-0.38400700	0.28452900
C	-0.60092500	2.85772500	0.17852000
C	-0.91195100	2.94026200	-1.18500000
C	-1.43453200	3.51659300	1.09085100
C	2.65761700	-0.12699800	1.77002400
C	-2.02296900	3.66216800	-1.61747600
C	-2.54692500	4.23822600	0.66063100
C	-2.84517000	4.31338800	-0.69862200
C	3.36016500	-0.14297600	0.43214900
C	4.23734100	0.88538200	0.07301100
C	3.89144200	-1.25772900	-1.46509000
C	4.74242500	-0.22052300	-1.83775200
B	-1.26707800	-3.64912600	0.16877700
H	-3.00675600	-2.17891400	1.58272300
H	-2.43010300	-0.52042100	1.47256400
H	-3.00936900	-0.48589400	-0.96159000
H	-0.54453100	-2.00984500	1.42688500
H	-0.62792600	0.44128600	1.35592400
H	-0.38926400	-1.81345500	-1.41538800
H	0.91278800	2.48144400	1.63742300
H	1.42996200	2.18919700	-0.03079900
H	-4.45727700	-3.11587900	-0.34954500

H	-4.69471400	-2.12583300	-1.80530400
H	-3.13833700	-2.91112600	-1.49469700
H	-4.45111400	0.54400000	0.78506100
H	-5.43839300	-0.12263000	-0.52361800
H	-5.29744500	-0.99507400	1.00921300
H	0.96047300	-0.11928800	2.76210000
H	-0.28347000	2.41671000	-1.89580800
H	-1.20622700	3.46831500	2.15171500
H	-0.83053600	-4.84827700	-1.30390800
H	-2.24957200	3.71108400	-2.67737600
H	-3.17865400	4.74006700	1.38612300
H	-3.71113400	4.87162600	-1.03807400
H	4.38114600	1.74373200	0.72182800
H	3.75932600	-2.13150900	-2.09580500
H	5.29124600	-0.25801100	-2.77426100

Deprotonated Bortezomib (de2) in diethyl ether

O	0.83971800	0.39685800	-1.45167200
O	-1.33868300	-4.56724100	1.06933300
O	-0.51633800	-3.90190900	-1.11141000
O	3.32825000	-0.39903100	2.73194700
N	-0.38909600	-1.26814500	-0.53113600
N	1.30197900	0.03353200	1.83146100
N	3.33753600	-1.15492300	-0.34356800
N	4.70908300	1.15799500	-1.12968400
C	-2.52017400	-1.51943200	0.78676600
C	-3.47372900	-1.40985500	-0.42123300
C	-1.08323200	-2.04311500	0.52255500
C	0.31863100	0.62249500	0.89821900
C	0.27672700	-0.10442100	-0.47140000
C	0.51160800	2.14888600	0.73744800
C	-3.91326500	-2.76997800	-0.98123200
C	-4.70465100	-0.57156600	-0.04135500
C	-0.73252500	2.87090600	0.26133600
C	-0.99480800	3.05512600	-1.10181400
C	-1.65523700	3.37003400	1.18999700
C	2.64513300	-0.13636300	1.74356500
C	-2.14619300	3.71987400	-1.52296100
C	-2.80829100	4.03354700	0.77230700
C	-3.05752600	4.21095200	-0.58838300
C	3.32964200	-0.04619900	0.39770400
C	4.02525100	1.10280200	0.01369400
C	4.02170200	-1.09787100	-1.48682900
C	4.69834600	0.05409000	-1.88265600
B	-1.00604900	-3.64985100	0.21877900
H	-2.96810600	-2.17393600	1.54382200
H	-2.46678700	-0.52187300	1.24390900
H	-2.94195000	-0.87214900	-1.21648600
H	-0.52362000	-1.91510500	1.45447900
H	-0.63829300	0.45360000	1.38945100
H	-0.28936100	-1.75566600	-1.41428300
H	0.79969600	2.54306700	1.71564200

H	1.33669900	2.32562400	0.04583600
H	-4.44432500	-3.35060500	-0.21934100
H	-4.59026100	-2.63373400	-1.83159600
H	-3.06501000	-3.36600200	-1.32084200
H	-4.41913800	0.42941100	0.29620200
H	-5.38334700	-0.45766900	-0.89281800
H	-5.26693600	-1.05071500	0.76875700
H	0.97052300	-0.08190700	2.78185300
H	-0.29500200	2.66150000	-1.82927200
H	-1.46515500	3.24588200	2.25196800
H	-0.48086300	-4.85651800	-1.23781600
H	-2.33134000	3.85325300	-2.58335400
H	-3.50773600	4.41494400	1.50833900
H	-3.95229300	4.72821000	-0.91666500
H	4.03113900	1.98843800	0.64112800
H	4.03193400	-1.99109200	-2.10283400
H	5.24524100	0.08418500	-2.81943000

Deprotonated Bortezomib (de2) in methanol

O	0.77129400	0.52445000	-1.44562300
O	-1.03276000	-4.55710300	1.13004300
O	-0.15153100	-3.88554600	-1.02248000
O	3.24594700	-0.62469700	2.64199700
N	-0.33820300	-1.22496200	-0.53643900
N	1.26403000	0.09207100	1.83519300
N	3.45563500	-1.05254100	-0.38495100
N	4.54217500	1.43275300	-1.08059700
C	-2.49451100	-1.64662700	0.67823900
C	-3.39530600	-1.67907900	-0.57363800
C	-1.00417900	-2.04157000	0.50168500
C	0.26769400	0.68272200	0.91680900
C	0.25050200	-0.01941700	-0.46468600
C	0.40891800	2.21678000	0.79498600
C	-3.67754200	-3.09736900	-1.08985200
C	-4.71710400	-0.95013300	-0.28519500
C	-0.86386100	2.90145100	0.33885300
C	-1.05893500	3.26532000	-0.99856900
C	-1.87911700	3.18749700	1.26174000
C	2.58158400	-0.18509200	1.70225200
C	-2.23554400	3.89669100	-1.40352700
C	-3.05739000	3.81575000	0.86070200
C	-3.23967100	4.17272400	-0.47586000
C	3.27834000	0.03045300	0.37617000
C	3.83079100	1.26753700	0.03600600
C	4.15983800	-0.88237500	-1.50544000
C	4.69713800	0.35550000	-1.85519300
B	-0.74830400	-3.63576400	0.25828600
H	-2.91919200	-2.30626900	1.44410100
H	-2.54724600	-0.63053700	1.09073200
H	-2.88150300	-1.12548200	-1.36984800
H	-0.51100200	-1.83086700	1.45518100

H	-0.68520900	0.47390800	1.40151900
H	-0.22573700	-1.68422000	-1.43234300
H	0.68433000	2.59268500	1.78381300
H	1.22402000	2.44681600	0.10857400
H	-4.18858100	-3.69352300	-0.32562800
H	-4.32460300	-3.06230000	-1.97237500
H	-2.76327300	-3.62357500	-1.36939600
H	-4.54432300	0.08606800	0.02058000
H	-5.36153500	-0.93682800	-1.16968300
H	-5.26902100	-1.44855300	0.52014200
H	0.93152300	-0.07260600	2.77812700
H	-0.28498300	3.04528500	-1.72451500
H	-1.74271500	2.92467400	2.30638400
H	-0.01664900	-4.83503100	-1.12029600
H	-2.36654800	4.17351900	-2.44396800
H	-3.82883700	4.03173900	1.59169000
H	-4.15361900	4.66450300	-0.78972200
H	3.70394300	2.13314500	0.67759000
H	4.30574500	-1.75098100	-2.13875700
H	5.26759600	0.47461000	-2.77015000

Deprotonated Bortezomib (de2) in water

O	0.75472300	0.55497900	-1.44029200
O	-0.95086400	-4.56808800	1.10993300
O	-0.09159400	-3.86603200	-1.04108800
O	3.23695100	-0.61996300	2.63933600
N	-0.32975500	-1.21376300	-0.53951700
N	1.25505000	0.10559400	1.84064600
N	3.45464100	-1.02889800	-0.38466300
N	4.52213600	1.46626600	-1.07378800
C	-2.47539100	-1.68123600	0.67650200
C	-3.37860700	-1.72698900	-0.57321100
C	-0.97843600	-2.04849200	0.49525100
C	0.25487100	0.69290700	0.92430200
C	0.24392500	-0.00126600	-0.46098000
C	0.38248600	2.22856400	0.81141800
C	-3.63962100	-3.14913200	-1.08983800
C	-4.71103200	-1.01923100	-0.28103300
C	-0.89309200	2.90286400	0.34756600
C	-1.06426400	3.30557800	-0.98186800
C	-1.93469900	3.14053000	1.25478500
C	2.57153200	-0.17283200	1.70355500
C	-2.24307100	3.92774100	-1.39480100
C	-3.11537800	3.75896600	0.84565800
C	-3.27366600	4.15496300	-0.48304500
C	3.26723100	0.05126000	0.37836200
C	3.80993800	1.29348300	0.04123000
C	4.15946500	-0.85106100	-1.50370300
C	4.68760600	0.39177000	-1.85003600

B	-0.68995100	-3.63544900	0.24214000
H	-2.88726300	-2.34873100	1.44278100
H	-2.54421100	-0.66706100	1.09099400
H	-2.87557600	-1.16475400	-1.37025800
H	-0.48802100	-1.83270000	1.44893500
H	-0.69684300	0.47318700	1.40663700
H	-0.21554600	-1.66369100	-1.43990600
H	0.64610200	2.60157400	1.80462200
H	1.20095100	2.47081800	0.13336800
H	-4.14204100	-3.75292000	-0.32584200
H	-4.28715700	-3.12368700	-1.97225700
H	-2.71754500	-3.66143800	-1.36979300
H	-4.55378300	0.01948100	0.02457800
H	-5.35797700	-1.01601800	-1.16373100
H	-5.25272100	-1.52645000	0.52572100
H	0.92452800	-0.05994500	2.78421100
H	-0.26967100	3.12370900	-1.69603500
H	-1.81766900	2.84754600	2.29372800
H	0.06171900	-4.81197700	-1.14596100
H	-2.35516200	4.23531700	-2.42879000
H	-3.90758100	3.93713100	1.56451200
H	-4.18951400	4.63928100	-0.80286500
H	3.67496100	2.15687800	0.68403800
H	4.31367400	-1.71714700	-2.13845500
H	5.25910800	0.51695100	-2.76348200

Ixazomib in gas phase

B	3.66303300	-0.31125500	1.59812600
C	3.98064400	-0.62204800	0.05577300
H	4.65064000	-1.48896000	0.05080400
C	4.63051900	0.53125200	-0.73569900
C	6.08969100	0.85187200	-0.35386600
C	7.05807800	-0.27575700	-0.74306300
C	6.52230600	2.17544900	-1.00045000
N	2.74635300	-1.07838600	-0.61128900
C	1.52097000	-0.67618800	-0.22455900
O	1.33703800	0.04037100	0.75795300
C	0.35410800	-1.18205100	-1.07060500
N	-0.87421700	-0.62430800	-0.55174400

C	-2.08172500	-0.95588400	-1.07475700
O	-2.21400700	-1.70825600	-2.02667800
C	-3.26087900	-0.27727700	-0.41435200
C	-4.29591800	-0.99717200	0.19077900
C	-5.40000700	-0.34163800	0.72824200
C	-5.49247900	1.04512300	0.66183800
C	-4.46334600	1.76426400	0.06507900
C	-3.35330900	1.11722800	-0.46462700
Cl	-4.56533600	3.51545900	-0.02332100
Cl	-4.21479900	-2.74208500	0.33389900
O	3.93966400	0.93785500	2.08706400
O	3.33287700	-1.39682600	2.37164800
H	4.01817800	1.42828400	-0.60282200
H	4.60070800	0.28651900	-1.80675800
H	6.13253800	0.98489800	0.73253400
H	7.04402600	-0.44542600	-1.82587000
H	6.81742700	-1.22347900	-0.25360200
H	8.08420400	-0.01998800	-0.46336600
H	6.47494700	2.11224900	-2.09357200
H	7.55079300	2.43051700	-0.72831700
H	5.87712600	2.99999500	-0.68473100
H	2.83041700	-1.63015900	-1.45299700
H	0.32410100	-2.27729200	-1.04314800
H	0.49462700	-0.89637700	-2.11873000
H	-0.78519100	-0.06599500	0.28862100
H	-6.18593800	-0.91707600	1.20024100
H	-6.35278900	1.55741600	1.07256800
H	-2.56449200	1.69206800	-0.93321400
H	3.73355700	1.07837800	3.01535500
H	3.08080800	-1.20597900	3.27949200

Ixazomib in diethyl ether

B	3.70647100	-0.33100900	1.63698500
C	3.97589700	-0.62434000	0.08395400
H	4.65748300	-1.48142800	0.05417600
C	4.59507100	0.53701900	-0.72165800
C	6.06989100	0.84967100	-0.39690400
C	7.01828600	-0.27090500	-0.85062500
C	6.47657100	2.18498600	-1.03660600
N	2.73701900	-1.10212400	-0.55932000

C	1.51420200	-0.67137300	-0.20874300
O	1.31628200	0.09393800	0.73709200
C	0.35649000	-1.20649600	-1.05204500
N	-0.88372200	-0.67163500	-0.53193500
C	-2.08445200	-0.96835500	-1.07199500
O	-2.22627700	-1.70930800	-2.03937000
C	-3.25943000	-0.27802000	-0.41747400
C	-4.30726900	-0.98464700	0.18076300
C	-5.40496400	-0.31976600	0.71849600
C	-5.47819800	1.06878200	0.65812600
C	-4.43671900	1.77463500	0.06732100
C	-3.33238800	1.11800900	-0.46234400
Cl	-4.51454900	3.52950200	-0.01340600
Cl	-4.24997300	-2.73529100	0.31562900
O	4.00939100	0.90379700	2.14385700
O	3.34618100	-1.41217200	2.40325900
H	3.99141500	1.43497400	-0.55839300
H	4.52080900	0.29978200	-1.79136700
H	6.16254500	0.96034500	0.68903300
H	6.95866600	-0.41453900	-1.93553900
H	6.79314200	-1.22894300	-0.37419300
H	8.05539700	-0.02322500	-0.60601900
H	6.37959900	2.14127000	-2.12740100
H	7.51704600	2.43243900	-0.80583100
H	5.84783700	3.00473000	-0.67740700
H	2.82273200	-1.69441900	-1.37356200
H	0.35106700	-2.30086400	-1.02132400
H	0.49337700	-0.91484800	-2.09825700
H	-0.80046300	-0.08606200	0.29068200
H	-6.20081100	-0.88447800	1.18640300
H	-6.33342500	1.58859600	1.06961900
H	-2.53347000	1.68438200	-0.92381300
H	3.86468600	1.01083800	3.08976000
H	3.16126100	-1.22400900	3.32920300

Ixazomib in methanol

B	3.75754900	-0.50247400	1.62362600
C	3.98038700	-0.62764000	0.04208200
H	4.68232900	-1.45727000	-0.09502200
C	4.54842100	0.62412800	-0.66061900
C	6.03265100	0.92392300	-0.36689600
C	6.97243500	-0.11542500	-0.99734700
C	6.39149100	2.33432800	-0.85676200
N	2.73725500	-1.07796600	-0.61320700
C	1.51720600	-0.65970600	-0.24481300
O	1.31675200	0.06525300	0.73391900
C	0.35984100	-1.15477400	-1.11390800
N	-0.88209200	-0.64758600	-0.56826100
C	-2.08339500	-0.92291800	-1.10977900
O	-2.23455000	-1.63124300	-2.10378800
C	-3.25534000	-0.25788100	-0.42531800

C	-4.28640600	-0.98682500	0.17489000
C	-5.38279100	-0.34647700	0.74343200
C	-5.47120400	1.04235400	0.71215100
C	-4.44637900	1.77036100	0.11911100
C	-3.34307000	1.13786300	-0.44133200
Cl	-4.54370500	3.52630800	0.07564500
Cl	-4.20712200	-2.74106400	0.27219800
O	4.08752900	0.66655000	2.25408500
O	3.37800000	-1.64778300	2.27844600
H	3.94217500	1.49058300	-0.37841900
H	4.43042100	0.49913800	-1.74470300
H	6.17668000	0.90233100	0.71898200
H	6.86509300	-0.12358800	-2.08792700
H	6.77826700	-1.12867200	-0.63542300
H	8.01646300	0.11725500	-0.76753200
H	6.24180200	2.42114000	-1.93896200
H	7.43896400	2.57031200	-0.64632100
H	5.77104200	3.09349200	-0.37148300
H	2.82017000	-1.62913200	-1.45640100
H	0.35631800	-2.24881900	-1.13628600
H	0.49887900	-0.81046300	-2.14343200
H	-0.79743700	-0.08543800	0.27066400
H	-6.16519300	-0.92800200	1.21323200
H	-6.32516000	1.54356400	1.14839700
H	-2.55671500	1.72177800	-0.90234000
H	3.98566600	0.66448800	3.21227400
H	3.24549700	-1.56068500	3.22887300

Ixazomib in water

B	3.76063700	-0.51672900	1.62079800
C	3.98043500	-0.62754700	0.03785800
H	4.68418700	-1.45413500	-0.10784600
C	4.54439900	0.63160500	-0.65498000
C	6.02870300	0.93160700	-0.36180100
C	6.96901400	-0.09972300	-1.00451800
C	6.38360700	2.34740900	-0.83884900
N	2.73747300	-1.07545000	-0.61951100
C	1.51750500	-0.65862400	-0.25001000
O	1.31636600	0.06124000	0.73259300
C	0.36042100	-1.14845600	-1.12255000
N	-0.88168900	-0.64461900	-0.57395800
C	-2.08315000	-0.91724800	-1.11550400
O	-2.23563800	-1.62095300	-2.11300400
C	-3.25464200	-0.25593200	-0.42673600
C	-4.28191200	-0.98821500	0.17582300
C	-5.37813300	-0.35163000	0.74880500
C	-5.47009800	1.03704800	0.71969800
C	-4.44902100	1.76838300	0.12429700
C	-3.34586200	1.13959800	-0.44062500
Cl	-4.55089400	3.52421900	0.08367100
Cl	-4.19756900	-2.74261100	0.27024600

O	4.09023300	0.64710800	2.26107000
O	3.38190400	-1.66749800	2.26642400
H	3.93711400	1.49440400	-0.36387700
H	4.42419300	0.51592700	-1.73980100
H	6.17545900	0.89969900	0.72346300
H	6.85915400	-0.09734600	-2.09485800
H	6.77742300	-1.11681400	-0.65215800
H	8.01309200	0.13269000	-0.77464300
H	6.23103300	2.44438500	-1.91977800
H	7.43114700	2.58335000	-0.62868600
H	5.76290600	3.10068800	-0.34473700
H	2.82098800	-1.62115500	-1.46624300
H	0.35698900	-2.24229800	-1.15155200
H	0.49991300	-0.79768700	-2.14980100
H	-0.79679100	-0.08579700	0.26720700
H	-6.15753900	-0.93565900	1.22045700
H	-6.32386400	1.53541500	1.15953800
H	-2.56235200	1.72610500	-0.90316900
H	3.99164800	0.63566700	3.21959500
H	3.25501300	-1.58947400	3.21847700

Ixazomib deprotonated (de2) in gas phase

B	5.09019900	-0.24130600	-1.56821300
C	3.93579500	0.18209700	-0.48560900
H	3.68905200	1.24073000	-0.61337600
C	4.38298900	-0.04536600	0.97818900
C	5.57984200	0.81669800	1.42345200
C	5.20874000	2.30168400	1.54829800
C	6.14728700	0.29573000	2.75177500
N	2.69518500	-0.57835400	-0.75886300
C	1.46101600	-0.24060900	-0.38747700
O	1.15069900	0.72900200	0.31402200
C	0.34906600	-1.18054100	-0.90759300
N	-0.91752000	-0.63927800	-0.45840400
C	-2.10968100	-1.21392200	-0.69907600
O	-2.27367400	-2.24678700	-1.33657300
C	-3.29750600	-0.44337100	-0.15176800
C	-4.17784000	-0.98560500	0.78909200
C	-5.29941200	-0.27895800	1.21358600
C	-5.56943300	0.98332600	0.69373500
C	-4.69547800	1.52727200	-0.23973000
C	-3.56720400	0.83250300	-0.65762400
Cl	-5.02489000	3.12410000	-0.90448000
Cl	-3.87460200	-2.56020500	1.50232800
O	6.25046700	0.29887100	-1.68405900
O	4.61370800	-1.35965200	-2.37095800
H	4.65333800	-1.10369000	1.09019700
H	3.53562700	0.13775500	1.65410400
H	6.34502200	0.72526000	0.64516700

H	4.40248500	2.44921600	2.27802000
H	4.87707400	2.71274700	0.59248400
H	6.07218000	2.89160800	1.87654900
H	5.39497600	0.33930000	3.55036700
H	7.00925600	0.89003700	3.07602300
H	6.47525400	-0.74501000	2.66058000
H	2.82385500	-1.33601300	-1.42090800
H	0.49612500	-2.19382600	-0.52081900
H	0.37839200	-1.24312100	-1.99966300
H	-0.81031300	0.19012800	0.12238100
H	-5.95892800	-0.71581800	1.95257700
H	-6.44320500	1.53513800	1.01528500
H	-2.89567900	1.27209900	-1.38424400
H	5.33350600	-1.57991100	-2.97115400

Ixazomib deprotonated (de2) in diethyl ether

B	5.04736800	0.51506600	-1.57353900
C	3.93625700	0.40279800	-0.38415700
H	3.66795000	1.40513300	-0.04360700
C	4.42315000	-0.42644900	0.83139100
C	5.61780400	0.15669800	1.60821300
C	5.28169400	1.49844100	2.27502900
C	6.11251000	-0.85380800	2.65400600
N	2.69977900	-0.20592800	-0.92051600
C	1.46486100	-0.01160000	-0.44970900
O	1.17623300	0.69222900	0.52580800
C	0.35427300	-0.74300500	-1.22518100
N	-0.91841900	-0.39549100	-0.62675400
C	-2.09431800	-0.90783200	-1.03138500
O	-2.21526200	-1.70977000	-1.95443700
C	-3.30159100	-0.38546600	-0.28215800
C	-4.11013700	-1.21106800	0.50378900
C	-5.25079600	-0.71647400	1.12846600
C	-5.60856900	0.61907500	0.96848400
C	-4.80477500	1.44410600	0.19049000
C	-3.65853100	0.95870500	-0.42751600
Cl	-5.24443300	3.13517400	-0.01723400
Cl	-3.68299400	-2.89613700	0.76294000
O	5.99414200	1.39356500	-1.65539400
O	4.82553100	-0.53776800	-2.54079400
H	4.69445200	-1.42732700	0.47286200
H	3.58542900	-0.56179500	1.52950100
H	6.42934900	0.33266700	0.89424000
H	4.44343500	1.38813600	2.97328300
H	5.00880800	2.25769800	1.53919800
H	6.13964200	1.87849700	2.83915400
H	5.33093600	-1.06907100	3.39242000
H	6.98328000	-0.46926300	3.19504400
H	6.39981600	-1.80143300	2.18756200
H	2.82488600	-0.76184500	-1.75717000
H	0.51919700	-1.82411200	-1.18098600
H	0.37745700	-0.45405800	-2.28029700

H	-0.85111500	0.22389400	0.17485500
H	-5.85724000	-1.37071200	1.74124800
H	-6.49752800	1.00740900	1.44797300
H	-3.04558800	1.61569400	-1.03139000
H	5.49059100	-0.44931000	-3.23236700

Ixazomib deprotonated (de2) in methanol

B	5.05712100	0.53470900	-1.56252700
C	3.93905500	0.41474500	-0.38394300
H	3.67393800	1.41380700	-0.03072600
C	4.41731300	-0.43304600	0.82310400
C	5.60357100	0.14121100	1.61961500
C	5.24424900	1.44735400	2.34379200
C	6.12240700	-0.90024600	2.62226300
N	2.70261400	-0.17842800	-0.93517200
C	1.46685400	0.02913700	-0.46955800
O	1.18775500	0.74373900	0.50165300
C	0.35307300	-0.69192400	-1.24499300
N	-0.91888000	-0.36520300	-0.63319200
C	-2.08975500	-0.88371200	-1.04136200
O	-2.19844400	-1.67965800	-1.97406700
C	-3.30239100	-0.38437300	-0.28823800
C	-4.10144200	-1.22743000	0.48916000
C	-5.24671100	-0.75312800	1.12057300
C	-5.61856200	0.58052200	0.97607900
C	-4.82409600	1.42276600	0.20697000
C	-3.67326900	0.95761500	-0.41823300
Cl	-5.28054500	3.11169900	0.01961500
Cl	-3.65523700	-2.91193400	0.72977700
O	5.97630900	1.44838100	-1.65443100
O	4.88964500	-0.54239500	-2.50373700
H	4.69198900	-1.42829300	0.45227300
H	3.57441100	-0.58117200	1.51160900
H	6.41040000	0.36065800	0.91112700
H	4.42073300	1.28603300	3.04914300
H	4.93998800	2.23252200	1.64751000
H	6.09991500	1.82479100	2.91249700
H	5.34560800	-1.16477100	3.34909600
H	6.98263100	-0.51765200	3.18055900
H	6.43237100	-1.81992600	2.11654400
H	2.82462000	-0.75488600	-1.75788600
H	0.52507200	-1.77225500	-1.22322300
H	0.36860600	-0.38156500	-2.29422400
H	-0.86335700	0.25638600	0.16665000
H	-5.84623700	-1.41958300	1.72674500
H	-6.51131100	0.95250900	1.46123900
H	-3.06828600	1.62803900	-1.01513300
H	5.55157000	-0.45625800	-3.19899200

Ixazomib deprotonated (de2) in water

B	5.05100900	0.58713900	-1.54862500
C	3.93791400	0.42766500	-0.37009900
H	3.67090100	1.41447100	0.01473200
C	4.42117600	-0.45636000	0.80876400
C	5.61477400	0.09036900	1.61354700
C	5.26525900	1.37646300	2.37733400
C	6.13647100	-0.98104300	2.58254600
N	2.70139700	-0.15124500	-0.93608500
C	1.46584000	0.04394600	-0.46441800
O	1.18761600	0.73561200	0.52360400
C	0.35229000	-0.66000700	-1.25517700
N	-0.92006900	-0.34872200	-0.63617600
C	-2.08997900	-0.85857600	-1.05739500
O	-2.19637900	-1.63307900	-2.00854400
C	-3.30423600	-0.37812500	-0.29498500
C	-4.10295000	-1.23941700	0.46255100
C	-5.24999700	-0.78111100	1.10245300
C	-5.62394900	0.55477700	0.98665300
C	-4.82975700	1.41505500	0.23748100
C	-3.67718400	0.96578200	-0.39606300
Cl	-5.28871000	3.10697700	0.08650900
Cl	-3.65418900	-2.92828300	0.66680600
O	5.95644300	1.51652100	-1.62565700
O	4.89621800	-0.47301600	-2.50996400
H	4.68960500	-1.44145300	0.40759500
H	3.58233100	-0.62165300	1.49835600
H	6.41821600	0.32666700	0.90634900
H	4.44812500	1.19666000	3.08559000
H	4.95578100	2.18167800	1.70673300
H	6.12682500	1.73608500	2.94858700
H	5.36360200	-1.26317000	3.30687300
H	7.00181600	-0.61742000	3.14551900
H	6.43949700	-1.88708000	2.04882400
H	2.82297600	-0.70971900	-1.77115500
H	0.52560000	-1.74032900	-1.25773900
H	0.36730000	-0.32612000	-2.29714600
H	-0.86643800	0.25517000	0.17715100
H	-5.84937000	-1.46158400	1.69296700
H	-6.51813800	0.91432700	1.47845200
H	-3.07255200	1.65013700	-0.97728500
H	5.55363400	-0.36520900	-3.20646900

Ixazomib deprotonated (de1) in gas phase

B	4.95906400	-0.47567400	-1.60090800
C	3.93013000	0.15867100	-0.49246100
H	3.72620900	1.21804100	-0.68642600
C	4.35912300	-0.00403900	0.98418100
C	5.57623900	0.83478200	1.41923100
C	5.28568700	2.34288800	1.38891000
C	6.03814200	0.41048200	2.82163200
N	2.68841600	-0.59055500	-0.74870000

C	1.45768400	-0.25402000	-0.38341400
O	1.13832900	0.71734500	0.31821000
C	0.35000900	-1.19878700	-0.90374500
N	-0.91896700	-0.65450200	-0.46157300
C	-2.11300700	-1.22549100	-0.69562200
O	-2.28782000	-2.26237300	-1.32401100
C	-3.29616300	-0.44329400	-0.15226500
C	-4.17688400	-0.97107600	0.79636200
C	-5.29442800	-0.25518000	1.21606800
C	-5.56033300	1.00273800	0.68354400
C	-4.68591500	1.53243100	-0.25756500
C	-3.56181100	0.82837500	-0.67087600
Cl	-5.01003900	3.12434900	-0.93844600
Cl	-3.87925100	-2.53955500	1.52572500
O	6.22309400	0.18780500	-1.66236800
O	4.59661000	-1.48834300	-2.32502600
H	4.58611200	-1.06436600	1.15501800
H	3.51114600	0.24576100	1.63666600
H	6.38750700	0.63641800	0.71200700
H	4.43925400	2.59237200	2.04019400
H	5.04422300	2.68521600	0.38053300
H	6.15526000	2.91506000	1.73247800
H	5.24737300	0.57652900	3.56380300
H	6.91833700	0.98019800	3.14124200
H	6.29932200	-0.65245500	2.84787100
H	2.87710000	-1.33378700	-1.43642700
H	0.49766900	-2.20922900	-0.51090200
H	0.38616200	-1.26761400	-1.99481900
H	-0.80807400	0.17803000	0.11488800
H	-5.95407300	-0.68142400	1.96115600
H	-6.43095400	1.56181700	1.00109100
H	-2.88968900	1.25704500	-1.40343100
H	6.73102900	-0.27170100	-2.34054900

Ixazomib deprotonated (de1) in diethyl ether

B	4.99598800	-0.21241600	-1.65865800
C	3.94594400	0.23979500	-0.49300000
H	3.75102400	1.31728700	-0.52658100
C	4.36601000	-0.14783400	0.94660700
C	5.56327300	0.62286300	1.53476200
C	5.25268900	2.11209500	1.74698800
C	6.00684700	-0.02165300	2.85670500
N	2.69662300	-0.45195200	-0.85243500
C	1.46630200	-0.13852100	-0.45006800
O	1.17961300	0.76634700	0.34693000
C	0.34849600	-1.00538100	-1.05786800
N	-0.92029900	-0.53360500	-0.54032200
C	-2.10111700	-1.11058100	-0.82441900
O	-2.23238700	-2.08787200	-1.55754200
C	-3.30201200	-0.43174700	-0.19988000
C	-4.12641600	-1.07027500	0.73081400
C	-5.25992600	-0.44213800	1.23765200

C	-5.59558300	0.83968300	0.81155500
C	-4.77667000	1.47885000	-0.11182500
C	-3.63718300	0.86151300	-0.61339600
Cl	-5.18841700	3.10039000	-0.65780900
Cl	-3.73134300	-2.67674400	1.32463500
O	6.20288200	0.54796800	-1.66265000
O	4.71450400	-1.17556600	-2.48892700
H	4.60117800	-1.21980900	0.95375200
H	3.50639600	-0.01272900	1.61646200
H	6.39233600	0.55089500	0.82384300
H	4.40307200	2.23907700	2.42821900
H	5.00680400	2.61709000	0.81004900
H	6.11244200	2.63047700	2.18436400
H	5.20145700	0.01302600	3.60006100
H	6.87236300	0.49808400	3.28077900
H	6.28276900	-1.07120100	2.71427700
H	2.85103900	-1.14454000	-1.58698800
H	0.50526600	-2.05642400	-0.79790400
H	0.37186100	-0.93570200	-2.14963900
H	-0.84435800	0.24170400	0.11091400
H	-5.87831400	-0.95075500	1.96584700
H	-6.47906600	1.33026600	1.19857400
H	-3.01242000	1.37442300	-1.33356500
H	6.75705800	0.21079800	-2.37616200

Ixazomib deprotonated (de1) in methanol

B	5.01495600	-0.00756400	-1.67158900
C	3.95009100	0.29739800	-0.47675100
H	3.75081400	1.37008100	-0.38399800
C	4.37342000	-0.25595800	0.90838000
C	5.54665300	0.46578000	1.59811000
C	5.19103100	1.90510200	2.00052900
C	6.00801000	-0.33199900	2.82695500
N	2.70103100	-0.34935500	-0.91156700
C	1.47012900	-0.06310600	-0.48545900
O	1.19511400	0.77347900	0.38639100
C	0.34855900	-0.86255700	-1.16820300
N	-0.91996600	-0.45117900	-0.60136800
C	-2.09518800	-1.01220000	-0.93184900
O	-2.21468100	-1.92130000	-1.75334100
C	-3.30118100	-0.41491100	-0.24136900
C	-4.08152800	-1.14069600	0.66272700
C	-5.22205400	-0.58735600	1.23570200
C	-5.60756200	0.70846500	0.90392000
C	-4.83139200	1.43470700	0.00820400
C	-3.68539500	0.89120000	-0.56019000
Cl	-5.30558500	3.07522900	-0.41616300
Cl	-3.61637600	-2.76964900	1.13743800
O	6.17475500	0.81891600	-1.61618600
O	4.79218000	-0.92133500	-2.57657800
H	4.63443500	-1.31494100	0.78642400
H	3.50754100	-0.22730800	1.58262300

H	6.37968300	0.50991000	0.88900100
H	4.34615500	1.91531600	2.69903300
H	4.91821100	2.51880300	1.13849200
H	6.03758600	2.39106300	2.49591200
H	5.20092900	-0.41846000	3.56380500
H	6.85588700	0.15465300	3.31939900
H	6.31657500	-1.34506900	2.55088000
H	2.84311900	-0.98772600	-1.69184700
H	0.50922300	-1.93377000	-1.01566600
H	0.36757700	-0.68159700	-2.24733500
H	-0.85687100	0.26838500	0.11116800
H	-5.80716600	-1.16237200	1.94145300
H	-6.49668900	1.14152500	1.34291200
H	-3.09472400	1.47172000	-1.25735200
H	6.75832900	0.57518900	-2.34460500

Ixazomib deprotonated (de1) in water

B	5.01158300	0.03126400	-1.67851200
C	3.95090000	0.30481200	-0.47277200
H	3.74874000	1.37454100	-0.35522600
C	4.38271600	-0.27741300	0.89800100
C	5.54899300	0.44058700	1.60341900
C	5.17665300	1.86444600	2.04428400
C	6.02354800	-0.38295700	2.81000000
N	2.70160300	-0.33565300	-0.91622300
C	1.47194100	-0.06197000	-0.47800300
O	1.19955800	0.75479000	0.41326300
C	0.34947700	-0.84996900	-1.17197100
N	-0.91892700	-0.44675700	-0.59910900
C	-2.09455400	-0.99788400	-0.94376800
O	-2.21341600	-1.89129300	-1.78279400
C	-3.30147100	-0.41069200	-0.24656000
C	-4.08261700	-1.14921800	0.64641600
C	-5.22426400	-0.60453200	1.22537700
C	-5.60993100	0.69555200	0.91081900
C	-4.83287300	1.43441300	0.02623000
C	-3.68577500	0.89946100	-0.54803900
Cl	-5.30725800	3.08035500	-0.37632800
Cl	-3.61693300	-2.78452700	1.09905900
O	6.17077100	0.85701000	-1.60681800
O	4.78697100	-0.85952600	-2.60611600
H	4.65479200	-1.33037800	0.75073200
H	3.51770500	-0.27407000	1.57388900
H	6.37956000	0.51340300	0.89369900
H	4.33444600	1.84579500	2.74578600
H	4.89294900	2.49708900	1.19953500
H	6.01877900	2.34801700	2.54934500
H	5.21947100	-0.49879100	3.54605500
H	6.86631000	0.10161200	3.31306000
H	6.34430100	-1.38448400	2.50705000
H	2.84034300	-0.95509300	-1.71198200

H	0.51023000	-1.92331500	-1.03498000
H	0.36800600	-0.65328900	-2.24828300
H	-0.85664500	0.25995300	0.12615600
H	-5.81016400	-1.18936400	1.92232800
H	-6.49992400	1.12198100	1.35449200
H	-3.09438400	1.48981200	-1.23623700
H	6.75256800	0.63239800	-2.34282900

Delanzomib in gas phase

B	-3.89540600	0.19244800	1.85973200
C	-3.75728600	-0.08509100	0.28667600
H	-4.58316300	0.42610400	-0.22107900
C	-3.79330100	-1.58532300	-0.06343900
C	-5.10330400	-2.31283600	0.29505000
C	-6.31211800	-1.77781400	-0.48710500
C	-4.94144300	-3.82317700	0.07099400
N	-2.49933900	0.49250500	-0.21712800
C	-1.89530000	1.49731200	0.43563500
O	-2.34636400	1.96717100	1.48336900
C	-0.61645000	2.09621800	-0.18277400
H	-0.22437100	2.76187100	0.58624500
C	-0.90717900	2.97259300	-1.43226000
H	-1.30056800	2.33461400	-2.22972400
C	-1.89410200	4.08875700	-1.12661500
O	0.30173800	3.59715100	-1.87092100
N	0.44765500	1.11384700	-0.42968700
C	0.46535200	0.13882200	-1.36411900
O	-0.45058100	-0.09632100	-2.15438500
C	1.73645000	-0.68172900	-1.38191200
C	1.83769300	-1.75421800	-2.26402500
C	3.02037200	-2.48763900	-2.25378600
C	4.03348000	-2.13077600	-1.37397100
C	3.84473100	-1.04125800	-0.50669100
N	2.70669600	-0.33226700	-0.53136700
C	4.89448800	-0.61384300	0.45372600
C	5.80069200	-1.53407600	0.99890000
C	6.78173800	-1.11932400	1.89560000
C	6.87620200	0.22265200	2.25938600
C	5.97803700	1.14627500	1.72528000
C	4.99335500	0.73163300	0.83462200
O	-3.06602900	-0.52028400	2.69812700
O	-5.01148900	0.87803400	2.27869800
H	-2.96165600	-2.07460500	0.45380900
H	-3.61226800	-1.70937500	-1.14091300
H	-5.29695600	-2.15632900	1.36411700
H	-6.15079800	-1.87632600	-1.56679300
H	-6.51146400	-0.72609800	-0.27010200
H	-7.21606700	-2.34062900	-0.23549600
H	-4.73958800	-4.04216600	-0.98362000
H	-5.84956200	-4.36411700	0.35340800
H	-4.11271400	-4.22660900	0.66011700
H	-2.08406200	0.14990800	-1.07981000

H	-2.85853000	3.69179000	-0.80545700
H	-2.04427300	4.69137000	-2.02413000
H	-1.50795200	4.73691600	-0.33590100
H	0.89934300	2.91701900	-2.19825300
H	1.27161900	1.14244600	0.15871500
H	1.01224200	-1.98208100	-2.92454500
H	3.15487800	-3.32336200	-2.93111700
H	4.97085300	-2.67164300	-1.37148200
H	5.72541400	-2.58535800	0.74607800
H	7.46681200	-1.84655100	2.31642700
H	7.64130400	0.54578900	2.95604900
H	6.04632900	2.19239100	2.00168100
H	4.29706600	1.44813000	0.41778700
H	-3.13077600	-0.29780000	3.63077200
H	-5.07996000	1.01489800	3.22769200

Delanzomib in diethyl ether

B	-3.67082700	-0.11287300	2.04478700
C	-3.72897000	-0.02610800	0.45126000
H	-4.55385700	0.64117800	0.17680900
C	-3.95421300	-1.40506100	-0.20338300
C	-5.27633700	-2.10641900	0.16244100
C	-6.50995300	-1.33629800	-0.33169300
C	-5.27803800	-3.53848800	-0.39184200
N	-2.48639100	0.54577900	-0.09461400
C	-1.85041800	1.56054800	0.51026600
O	-2.25143000	2.06831000	1.56189800
C	-0.58745800	2.12098500	-0.17977600
H	-0.15782200	2.80860200	0.54861500
C	-0.91325500	2.95424100	-1.44895500
H	-1.31584600	2.28893300	-2.21843400
C	-1.90392900	4.07209800	-1.16356200
O	0.28380100	3.57295500	-1.93478500
N	0.45676700	1.11892100	-0.43024100
C	0.43302000	0.10739200	-1.32363800
O	-0.51730700	-0.14793400	-2.07010500
C	1.69449500	-0.72546500	-1.35324200
C	1.76930200	-1.82204700	-2.20736500
C	2.94892600	-2.56176900	-2.20659800
C	3.98524900	-2.18650100	-1.36262000
C	3.82270400	-1.07271300	-0.52060500
N	2.68732600	-0.35932700	-0.53564800
C	4.89743900	-0.62380700	0.40275600
C	5.82903900	-1.52927700	0.93111900
C	6.83233900	-1.09403300	1.79378900
C	6.92437400	0.25345500	2.13992100
C	6.00146000	1.16258600	1.62170600
C	4.99489000	0.72768700	0.76482700
O	-2.62535000	-0.79600300	2.61318500
O	-4.75187300	0.35699600	2.74600900
H	-3.11794800	-2.05626400	0.07333900

H	-3.91448500	-1.29138500	-1.29568700
H	-5.33837300	-2.17538800	1.25642900
H	-6.48064900	-1.21766400	-1.42078300
H	-6.58286800	-0.34157500	0.11361100
H	-7.42796800	-1.87607200	-0.08120300
H	-5.21244700	-3.53297400	-1.48556600
H	-6.19629300	-4.06554300	-0.11673500
H	-4.43088100	-4.11530800	-0.00927000
H	-2.09387300	0.17697500	-0.95757700
H	-2.86319800	3.67516300	-0.82715600
H	-2.07059500	4.64878400	-2.07526900
H	-1.51792500	4.74446700	-0.39308900
H	0.90058200	2.88368900	-2.20571500
H	1.30071500	1.16107600	0.12939500
H	0.93035200	-2.06959300	-2.84287800
H	3.06208700	-3.41640500	-2.86331800
H	4.91791700	-2.73489400	-1.36883600
H	5.76015700	-2.58368300	0.69092700
H	7.53731500	-1.80978500	2.20108700
H	7.70658900	0.59195300	2.80975400
H	6.06743900	2.21253100	1.88375200
H	4.28020000	1.43373600	0.36143300
H	-2.62041500	-0.82350100	3.57565300
H	-4.70440200	0.24906700	3.70185400

Delanzomib in methanol

B	-3.57850000	-0.28840000	2.08727200
C	-3.71689500	-0.01848100	0.52007000
H	-4.52022600	0.71080900	0.36744400
C	-4.04837500	-1.30444900	-0.26723900
C	-5.37888200	-1.98806300	0.10171400
C	-6.60033500	-1.11473300	-0.22127600
C	-5.48303200	-3.34389800	-0.61181100
N	-2.48039300	0.55376500	-0.04038000
C	-1.82805000	1.56119000	0.55610000
O	-2.20623700	2.07012800	1.61779900
C	-0.57424900	2.11528200	-0.15627300
H	-0.13151000	2.80579200	0.56137000
C	-0.91592300	2.94169000	-1.42556000
H	-1.31411700	2.27046600	-2.19195000
C	-1.91810100	4.04940400	-1.14034500
O	0.27285900	3.57221000	-1.91863200
N	0.46414500	1.10992000	-0.41533400
C	0.42685300	0.09063700	-1.29881000
O	-0.53972700	-0.17541700	-2.02225100
C	1.69105900	-0.73633100	-1.34651000
C	1.76519200	-1.82806100	-2.20665200
C	2.95029000	-2.55952200	-2.22190400
C	3.99278800	-2.18089200	-1.38719100

C	3.83068500	-1.07234200	-0.53795500
N	2.68971500	-0.36789800	-0.53672000
C	4.91140000	-0.61870900	0.37669700
C	5.86120900	-1.51745500	0.88454300
C	6.86927900	-1.07750300	1.73979500
C	6.94804700	0.26780700	2.09886200
C	6.00725800	1.17028000	1.60055900
C	4.99604500	0.73082200	0.75092000
O	-2.49013200	-0.99671800	2.52512200
O	-4.61495000	0.10275500	2.89457500
H	-3.23094600	-2.01921500	-0.12023500
H	-4.06760200	-1.06673600	-1.33963000
H	-5.37948400	-2.18305600	1.18203400
H	-6.63007800	-0.87248700	-1.28958700
H	-6.59735000	-0.17529500	0.33626500
H	-7.52673800	-1.64112200	0.02646100
H	-5.48271600	-3.21255400	-1.69958000
H	-6.40780600	-3.86052700	-0.33912300
H	-4.64367800	-3.99606500	-0.35308800
H	-2.09732200	0.18189400	-0.90667100
H	-2.87551000	3.64152500	-0.81163600
H	-2.08859800	4.62669200	-2.05101800
H	-1.54241300	4.72392700	-0.36632400
H	0.90562400	2.88972500	-2.17112600
H	1.32255200	1.16531200	0.12119000
H	0.92348900	-2.08106500	-2.83624400
H	3.06246000	-3.41001600	-2.88380800
H	4.92824700	-2.72399800	-1.40580900
H	5.80479200	-2.56990900	0.63336600
H	7.58866600	-1.78804000	2.13059100
H	7.73391900	0.60975800	2.76261600
H	6.06301000	2.21841000	1.87209800
H	4.26822300	1.43232600	0.36322400
H	-2.45561500	-1.16580600	3.47325900
H	-4.52314800	-0.12296800	3.82726500

Delanzomib in water

B	-3.58367800	-0.28507700	2.08843500
C	-3.71773300	-0.01677200	0.52055000
H	-4.51914200	0.71391100	0.36460200
C	-4.05046400	-1.30373700	-0.26477100
C	-5.38339100	-1.98339800	0.10286400
C	-6.60216600	-1.10910600	-0.22758600
C	-5.48793200	-3.34188200	-0.60553600
N	-2.47895200	0.55163600	-0.03882100
C	-1.82779500	1.56212100	0.55324900
O	-2.20788900	2.07694500	1.61173600
C	-0.57280000	2.11271500	-0.15966200
H	-0.12965200	2.80449600	0.55649500
C	-0.91266000	2.93649400	-1.43122400
H	-1.30810000	2.26330100	-2.19730500

C	-1.91691000	4.04360800	-1.15093800
O	0.27665400	3.56748600	-1.92256500
N	0.46489900	1.10591500	-0.41581300
C	0.42755600	0.08426800	-1.29650900
O	-0.53995200	-0.18485400	-2.01777600
C	1.69257500	-0.74136200	-1.34375300
C	1.76803700	-1.83375400	-2.20291600
C	2.95419000	-2.56358300	-2.21786600
C	3.99642700	-2.18267500	-1.38388300
C	3.83297500	-1.07355100	-0.53562900
N	2.69093700	-0.37086000	-0.53450800
C	4.91340800	-0.61724400	0.37807900
C	5.86539700	-1.51381300	0.88577900
C	6.87319600	-1.07130000	1.74008700
C	6.94951500	0.27441200	2.09833000
C	6.00656700	1.17473400	1.60010700
C	4.99562900	0.73274700	0.75138700
O	-2.49792000	-0.99489700	2.53003800
O	-4.62062200	0.11071300	2.89284100
H	-3.23508800	-2.02010900	-0.11419900
H	-4.06675100	-1.06836100	-1.33769400
H	-5.38771400	-2.17410900	1.18394300
H	-6.62853500	-0.87199100	-1.29712300
H	-6.59870800	-0.16686400	0.32525100
H	-7.53028300	-1.63238100	0.02030100
H	-5.48366000	-3.21489600	-1.69381300
H	-6.41470100	-3.85543200	-0.33383900
H	-4.65082600	-3.99470000	-0.34124700
H	-2.09547500	0.17719900	-0.90391400
H	-2.87534600	3.63516700	-0.82594400
H	-2.08443700	4.61972600	-2.06290800
H	-1.54494700	4.71936300	-0.37615200
H	0.91194600	2.88544200	-2.17006600
H	1.32458100	1.16446700	0.11838800
H	0.92675800	-2.08871000	-2.83228300
H	3.06734600	-3.41449900	-2.87902200
H	4.93256700	-2.72458900	-1.40231100
H	5.81111400	-2.56648700	0.63510300
H	7.59433700	-1.78015200	2.13069800
H	7.73519300	0.61834200	2.76128900
H	6.06045400	2.22315400	1.87090600
H	4.26625700	1.43266100	0.36370900
H	-2.46821100	-1.16608900	3.47804800
H	-4.53267400	-0.11597600	3.82576100

Delanzomib deprotonated (de2) in gas phase

B	1.72595300	-1.85192800	0.86027400
C	2.48989400	-0.77434800	-0.10311400
H	2.37836800	-1.07528300	-1.14758000

C	3.99781900	-0.64096500	0.21413300
C	4.80725800	-1.94436300	0.07596800
C	4.83405800	-2.46422500	-1.36864800
C	6.23553900	-1.74053900	0.60283200
N	1.82024000	0.53977900	0.02747000
C	1.75673100	1.49852900	-0.91564200
O	2.38184900	1.49994400	-1.97260700
C	0.77048000	2.65402500	-0.60200300
H	0.68031100	3.22788600	-1.52787600
C	1.28396200	3.62399800	0.49907800
H	1.11679800	3.13709000	1.47208800
C	2.76706000	3.94337700	0.34739100
O	0.59519100	4.86999300	0.45706300
N	-0.53274500	2.04215000	-0.28222100
C	-1.72280900	2.65123200	-0.15944000
O	-1.91110000	3.87300900	-0.13295600
C	-2.88903300	1.68971300	-0.03326500
C	-4.17718200	2.18459700	0.15556600
C	-5.20707300	1.25580900	0.28208800
C	-4.91950700	-0.10146300	0.21284200
C	-3.59237400	-0.51840100	0.00302100
N	-2.61024200	0.38760900	-0.11071200
C	-3.20286100	-1.94883400	-0.08785700
C	-4.12151700	-2.94241400	-0.46100800
C	-3.72105600	-4.27302400	-0.55145100
C	-2.40031700	-4.63141200	-0.27384800
C	-1.47372000	-3.65585900	0.09573200
C	-1.88090700	-2.32712900	0.18735900
O	1.66594200	-3.12842900	0.69950900
O	1.08954300	-1.13507800	1.95587700
H	4.09822300	-0.27577000	1.24462100
H	4.43908300	0.12557100	-0.43817500
H	4.31334700	-2.70760600	0.68729100
H	5.23834500	-1.70763200	-2.05237800
H	3.83291600	-2.73495000	-1.70840900
H	5.46168700	-3.35923100	-1.44730600
H	6.76662800	-0.97910500	0.01804500
H	6.81640500	-2.66798000	0.54505700
H	6.23127700	-1.41264600	1.64771700
H	1.27700400	0.63481100	0.87719400
H	3.38735900	3.05925000	0.50349300
H	3.03759900	4.70075100	1.08673200
H	2.97319300	4.34449000	-0.64803100
H	-0.35077400	4.69945700	0.29024500
H	-0.59263700	1.02874100	-0.30715500
H	-4.33785300	3.25302900	0.20554500
H	-6.22698000	1.58744900	0.44585200
H	-5.70709000	-0.83312300	0.33934800
H	-5.14337500	-2.67903200	-0.71146200
H	-4.43902500	-5.02897700	-0.85307500
H	-2.09258600	-5.66887200	-0.35347600
H	-0.42992900	-3.88484900	0.30768100
H	-1.16018400	-1.57714600	0.48433200
H	0.68326800	-1.79878300	2.52305900

Delanzomib deprotonated (de2) in diethyl ether

B	-1.77555000	-1.97116400	-0.87802000
C	-2.53245900	-0.89733400	0.09077200
H	-2.45730600	-1.21950900	1.13126000
C	-4.02649800	-0.70285600	-0.26883800
C	-4.91353300	-1.95380200	-0.12316900
C	-5.03391500	-2.41915700	1.33551400
C	-6.30494500	-1.68791100	-0.71715100
N	-1.82680300	0.40092700	0.00353900
C	-1.75944000	1.33278700	0.96963200
O	-2.35344100	1.27562500	2.04775800
C	-0.83871200	2.53640300	0.65765800
H	-0.74527400	3.09350600	1.59340700
C	-1.44850100	3.50147400	-0.40097900
H	-1.29804500	3.04501500	-1.39023700
C	-2.93662800	3.74988300	-0.18252700
O	-0.81430200	4.77793600	-0.35817100
N	0.48008800	1.99838200	0.27719300
C	1.62732900	2.67670100	0.10177600
O	1.73038500	3.90945100	0.04731400
C	2.84558300	1.79156100	-0.04551000
C	4.09217600	2.35959300	-0.29113600
C	5.17699500	1.49566000	-0.42419400
C	4.97851800	0.12657400	-0.30447800
C	3.68748500	-0.36497200	-0.04141800
N	2.65012000	0.47590100	0.07689300
C	3.39906000	-1.81554500	0.10465800
C	4.38882800	-2.72569900	0.50555400
C	4.08990100	-4.07904500	0.64778800
C	2.79985400	-4.54546300	0.39188700
C	1.80560800	-3.65034200	-0.00563900
C	2.10631800	-2.29894300	-0.14648000
O	-1.65507000	-3.24508800	-0.68584200
O	-1.23673100	-1.27120600	-2.02561800
H	-4.08362300	-0.35371600	-1.30749700
H	-4.44698700	0.09895900	0.35345900
H	-4.44852200	-2.76544800	-0.69451600
H	-5.45230900	-1.62476200	1.96480800
H	-4.06575900	-2.70419600	1.75247100
H	-5.69400300	-3.28941300	1.41210100
H	-6.81247000	-0.87778400	-0.18007600
H	-6.93988700	-2.57755800	-0.65224900
H	-6.23972400	-1.39899100	-1.77089600
H	-1.30594700	0.53785800	-0.85352600
H	-3.52309500	2.84000300	-0.31968100
H	-3.27745700	4.49280900	-0.90678200
H	-3.12047300	4.14047100	0.82177900
H	0.14871100	4.64017400	-0.25769500
H	0.61664400	0.99394000	0.34136700
H	4.19069200	3.43287500	-0.37730000
H	6.16701600	1.88632500	-0.62952400

H	5.80857200	-0.55582900	-0.43130400
H	5.39010300	-2.37986500	0.73500000
H	4.86366400	-4.76731400	0.96981200
H	2.56991400	-5.59888000	0.50775700
H	0.79092200	-3.97974700	-0.20480300
H	1.33193400	-1.61113100	-0.45901400
H	-0.79825600	-1.91959100	-2.58724200

Delanzomib deprotonated (de2) in methanol

B	-1.95449700	-2.11434800	-0.94054500
C	-2.62293500	-1.01705800	0.06065900
H	-2.54314700	-1.36788500	1.09240100
C	-4.11560600	-0.74421700	-0.26012500
C	-5.07179600	-1.93253500	-0.04553000
C	-5.20543400	-2.31389600	1.43659900
C	-6.45157200	-1.61810600	-0.64219600
N	-1.86294800	0.24932000	-0.01175600
C	-1.80596600	1.18188600	0.95238500
O	-2.40684600	1.11309400	2.02843700
C	-0.91968800	2.40792000	0.64478700
H	-0.83492400	2.96000800	1.58461500
C	-1.57903700	3.36246700	-0.39832800
H	-1.40176400	2.93406200	-1.39489000
C	-3.07817800	3.53419500	-0.18286800
O	-1.00915000	4.66849400	-0.32718500
N	0.41509500	1.92434200	0.24597500
C	1.52468300	2.66803900	0.08772100
O	1.54823600	3.90674900	0.05623300
C	2.79936800	1.87247800	-0.06533200
C	3.99541900	2.53732000	-0.32022900
C	5.14754600	1.76655700	-0.45334700
C	5.06058900	0.38776800	-0.32048100
C	3.81462900	-0.20411000	-0.04828700
N	2.70777000	0.54471300	0.06568800
C	3.66414500	-1.67435200	0.11269600
C	4.72652200	-2.46686400	0.57289800
C	4.57375800	-3.84365500	0.72125300
C	3.35840700	-4.45253600	0.40941500
C	2.29422300	-3.67337000	-0.04674800
C	2.44387900	-2.29684500	-0.19030000
O	-2.00250500	-3.40493500	-0.80019600
O	-1.30378800	-1.44348200	-2.03685300
H	-4.18676200	-0.42035500	-1.30603200
H	-4.46593300	0.09864600	0.34999900
H	-4.65747500	-2.79527900	-0.57898300
H	-5.59952900	-1.47359700	2.01992200
H	-4.24709000	-2.60425400	1.87390700
H	-5.89303800	-3.15643100	1.56088300
H	-6.90194300	-0.74784300	-0.15066400
H	-7.13804300	-2.46149700	-0.51729700
H	-6.38207200	-1.39744900	-1.71186400
H	-1.34110900	0.38673700	-0.86787900

H	-3.62145000	2.60330700	-0.35082300
H	-3.44982100	4.27902000	-0.88964700
H	-3.28731300	3.88791700	0.83002000
H	-0.03996800	4.57016700	-0.23035600
H	0.60205400	0.92819200	0.30890400
H	4.00866900	3.61420200	-0.41373400
H	6.10082000	2.23544000	-0.66660000
H	5.94272300	-0.22616800	-0.44375100
H	5.67164400	-2.00977600	0.84082500
H	5.40261300	-4.43849000	1.08781300
H	3.24023600	-5.52403600	0.52380200
H	1.34585200	-4.13736800	-0.29249100
H	1.61647200	-1.69629600	-0.54653800
H	-0.91195500	-2.10371900	-2.61950500

Delanzomib deprotonated (de2) in water

B	-1.94096800	-2.17672000	-0.77800700
C	-2.64228300	-1.01849000	0.12660900
H	-2.61599300	-1.30982300	1.17923900
C	-4.11423500	-0.74884600	-0.28122400
C	-5.09206000	-1.91916600	-0.06632700
C	-5.30692700	-2.23460500	1.42169600
C	-6.43567900	-1.62017600	-0.74752000
N	-1.86637900	0.23573200	0.02006200
C	-1.82142900	1.20449300	0.94837800
O	-2.44256500	1.17869700	2.01508900
C	-0.92837300	2.41706000	0.61200200
H	-0.84938000	2.99566100	1.53618800
C	-1.57857300	3.34259200	-0.46309500
H	-1.38896500	2.88786500	-1.44548700
C	-3.08020600	3.51875300	-0.27058700
O	-1.00968700	4.65081100	-0.42047000
N	0.40978600	1.92559100	0.23608100
C	1.52047100	2.67157100	0.09668400
O	1.53829200	3.91068100	0.07037800
C	2.80027300	1.88251200	-0.04111200
C	3.99837600	2.55611000	-0.26119600
C	5.15638600	1.79255100	-0.38365900
C	5.07221900	0.41145900	-0.27659100
C	3.82345300	-0.18977600	-0.03995300
N	2.71112000	0.55226500	0.06612200
C	3.67750800	-1.66373400	0.08788800
C	4.73344500	-2.45938600	0.55755800
C	4.58712000	-3.84021000	0.67077300
C	3.38482800	-4.45001200	0.31345500
C	2.32709700	-3.66761800	-0.15209600
C	2.46974200	-2.28701500	-0.26022800
O	-2.00169000	-3.45642300	-0.55923400
O	-1.24508900	-1.57765200	-1.88662300
H	-4.12846300	-0.46951700	-1.34212200
H	-4.48710600	0.12250900	0.27317900
H	-4.65908400	-2.80720800	-0.54042300

H	-5.72385600	-1.36677100	1.94584900
H	-4.37612300	-2.51201900	1.92244100
H	-6.00814100	-3.06582100	1.54579900
H	-6.90214900	-0.72603600	-0.31788100
H	-7.13608900	-2.45199000	-0.62274200
H	-6.30833700	-1.44650700	-1.82049100
H	-1.33052200	0.33684400	-0.83243200
H	-3.62098600	2.58356100	-0.42191800
H	-3.44336500	4.24494100	-1.00080400
H	-3.30238500	3.89766900	0.73031000
H	-0.04431200	4.55744800	-0.28864200
H	0.59379200	0.92896500	0.29807600
H	4.00905500	3.63436700	-0.33718000
H	6.11176600	2.26868500	-0.56946500
H	5.95898500	-0.19701300	-0.39328100
H	5.66822200	-2.00225100	0.85948900
H	5.41070900	-4.43761400	1.04487700
H	3.27184300	-5.52470200	0.39963300
H	1.38932000	-4.13289800	-0.43340700
H	1.64701000	-1.68444500	-0.62419800
H	-0.83329500	-2.27273300	-2.41227300

Delanzomib deprotonated (de1) in gas phase

B	1.70507200	-2.00287900	0.70111900
C	2.60303900	-0.94858700	-0.16958600
H	2.58363700	-1.20314300	-1.23567900
C	4.07695800	-0.78028300	0.26854400
C	5.00414700	-1.97632500	-0.02230900
C	5.19879500	-2.20990500	-1.52813400
C	6.36381200	-1.76981600	0.66249900
N	1.87606100	0.32180200	-0.00825300
C	1.91727800	1.37394800	-0.83898400
O	2.67679400	1.49463200	-1.80021000
C	0.93995500	2.52454500	-0.49997300
H	0.90353500	3.15564400	-1.39123400
C	1.45096900	3.40956000	0.67764000
H	1.17699000	2.89273200	1.60951000
C	2.95962500	3.62737700	0.65378700
O	0.85711100	4.70565900	0.64910800
N	-0.40528200	1.97092900	-0.25714200
C	-1.54596500	2.67569300	-0.21380700
O	-1.62403400	3.91069700	-0.24577900
C	-2.80388700	1.84090300	-0.09997900
C	-4.03258700	2.48446100	0.03767500
C	-5.16404200	1.68641100	0.17292000
C	-5.02823200	0.30436800	0.15854200
C	-3.75279800	-0.26507200	-0.00768900
N	-2.66818800	0.51408400	-0.12695900
C	-3.54709200	-1.73587300	-0.04319500
C	-4.56723900	-2.59280300	-0.48765000

C	-4.37583700	-3.97084500	-0.51959800
C	-3.15901100	-4.51427200	-0.10593200
C	-2.14042200	-3.66921000	0.33080300
C	-2.32278000	-2.28849600	0.36255200
O	2.21180900	-3.33691100	0.70308300
O	0.63015800	-1.60892700	1.31987500
H	4.09232200	-0.58098800	1.34809000
H	4.49359200	0.10941900	-0.22256600
H	4.53673300	-2.87081900	0.40017300
H	5.61813600	-1.31988800	-2.01210000
H	4.25553700	-2.44503400	-2.02564800
H	5.88425300	-3.04624000	-1.70753700
H	6.86348200	-0.87008300	0.28267900
H	7.03239700	-2.62024300	0.48609100
H	6.24877300	-1.65316200	1.74507000
H	1.14789400	0.21259800	0.70303800
H	3.50287300	2.69640400	0.82251100
H	3.21754800	4.33620600	1.44438200
H	3.27402700	4.04694600	-0.30455100
H	-0.07177100	4.61551500	0.36735800
H	-0.52518500	0.96203000	-0.23493400
H	-4.06863800	3.56530200	0.04670800
H	-6.14243000	2.13694000	0.30279700
H	-5.89360400	-0.33101800	0.29668300
H	-5.50743100	-2.18260100	-0.83994300
H	-5.17027600	-4.61716800	-0.87812700
H	-3.00595600	-5.58825600	-0.13222100
H	-1.18443500	-4.06625600	0.65061700
H	-1.49572400	-1.67443600	0.70994500
H	1.62740600	-3.84957000	1.27238700

Delanzomib deprotonated (de1) in diethyl ether

B	-1.83603700	-1.98216300	-0.90752700
C	-2.60940200	-0.96887800	0.11129300
H	-2.52697700	-1.30578300	1.15006100
C	-4.10372800	-0.72589600	-0.21693800
C	-5.05819200	-1.90474000	0.05431200
C	-5.16321800	-2.23917500	1.55008100
C	-6.44923000	-1.60008100	-0.52179100
N	-1.85803200	0.29235400	-0.00481200
C	-1.84963000	1.30075900	0.87635900
O	-2.53799300	1.35110800	1.90044200
C	-0.91165100	2.47868100	0.53235000
H	-0.86321700	3.09533600	1.43335200
C	-1.47697000	3.37151000	-0.61383800
H	-1.23466000	2.87413000	-1.56360700
C	-2.98400200	3.57886600	-0.52546700
O	-0.88726300	4.67186800	-0.58655100
N	0.43287000	1.95000300	0.24377400
C	1.56471100	2.66895300	0.16882100
O	1.61778400	3.90709900	0.17785900
C	2.82767900	1.84738500	0.05580000

C	4.05205600	2.49278700	-0.09525800
C	5.18985600	1.69872900	-0.21011400
C	5.06109000	0.31733500	-0.16433000
C	3.78771300	-0.25418600	0.00706300
N	2.69673000	0.51849200	0.10721200
C	3.59192000	-1.72597600	0.07145100
C	4.60478800	-2.56644500	0.55865900
C	4.41847900	-3.94526400	0.61817900
C	3.21639300	-4.50711300	0.18884700
C	2.20313600	-3.67909900	-0.29427500
C	2.38274100	-2.29945800	-0.35069100
O	-2.27645000	-3.33548400	-0.80791900
O	-0.90973700	-1.55340400	-1.71787600
H	-4.18011500	-0.44973600	-1.27661200
H	-4.45604200	0.14054700	0.35779100
H	-4.66005100	-2.78475800	-0.46044300
H	-5.53364000	-1.37760200	2.11765800
H	-4.19817500	-2.52654700	1.97349800
H	-5.85618100	-3.07102100	1.71477600
H	-6.88562400	-0.71332700	-0.04681200
H	-7.13784000	-2.43589600	-0.35915700
H	-6.40097200	-1.41068700	-1.59877500
H	-1.22171500	0.26481800	-0.80268500
H	-3.52883100	2.64626400	-0.67872600
H	-3.28435800	4.28643700	-1.30129900
H	-3.26214100	3.99466000	0.44596100
H	0.05690900	4.57627900	-0.35418300
H	0.57799200	0.94506900	0.25517900
H	4.09195800	3.57268800	-0.12784900
H	6.16512400	2.15199600	-0.34548900
H	5.93164500	-0.31465200	-0.28058400
H	5.53580800	-2.14413500	0.91878500
H	5.20809400	-4.57801600	1.00804800
H	3.06944300	-5.58052400	0.23415700
H	1.26519400	-4.10340300	-0.63172900
H	1.58179900	-1.67778700	-0.73449300
H	-1.79218800	-3.85115200	-1.46312900

Delanzomib deprotonated (de1) in methanol

B	-1.94311800	-1.88649300	-1.13412300
C	-2.60836600	-0.95690400	0.02720000
H	-2.44368400	-1.37536900	1.02535700
C	-4.12421300	-0.69745800	-0.16646700
C	-5.05369800	-1.90369500	0.06768400
C	-5.05601000	-2.36441100	1.53331300
C	-6.48162300	-1.56465300	-0.38567300
N	-1.86662500	0.31422500	-0.04673500
C	-1.79570500	1.25542900	0.90311500
O	-2.39839600	1.21579300	1.98138300
C	-0.88439000	2.45904900	0.57841600
H	-0.80570900	3.03569500	1.50383000
C	-1.50035000	3.39437500	-0.50443800

H	-1.32388500	2.92669100	-1.48307500
C	-2.99600100	3.61845700	-0.31469200
O	-0.88884700	4.68481500	-0.47167200
N	0.44919000	1.94224000	0.22158200
C	1.58225200	2.65918400	0.12691700
O	1.63461500	3.89785400	0.13012800
C	2.84121200	1.83461900	0.00197100
C	4.06341100	2.47280600	-0.18867400
C	5.19871600	1.67411600	-0.30078600
C	5.06978400	0.29501600	-0.21209100
C	3.79892100	-0.26934500	-0.00386100
N	2.70936100	0.50673300	0.09126300
C	3.60204000	-1.73892300	0.10722600
C	4.62530800	-2.57514400	0.57879200
C	4.42977900	-3.95089500	0.67897000
C	3.20979400	-4.51512900	0.30702200
C	2.18436900	-3.69209100	-0.16061800
C	2.37618700	-2.31657200	-0.25661200
O	-2.22739200	-3.27420500	-0.98052800
O	-1.22149300	-1.37277000	-2.09288100
H	-4.27795100	-0.32933000	-1.18875100
H	-4.43631400	0.11244300	0.50509200
H	-4.69113400	-2.73491200	-0.54626400
H	-5.39351200	-1.55707100	2.19361300
H	-4.06419900	-2.67730100	1.86872900
H	-5.73344300	-3.21283400	1.67298700
H	-6.88743600	-0.72604300	0.19204000
H	-7.15344000	-2.41788200	-0.24830000
H	-6.50753200	-1.28393600	-1.44314400
H	-1.32665200	0.39001300	-0.90694600
H	-3.56400100	2.69787700	-0.45646600
H	-3.33801200	4.34788800	-1.05193200
H	-3.20633800	4.01437900	0.68238000
H	0.06766200	4.56736600	-0.30242100
H	0.60104200	0.93885200	0.24984400
H	4.10879600	3.55110800	-0.25110200
H	6.17167900	2.12196700	-0.46460300
H	5.93920000	-0.33939300	-0.32104500
H	5.57293000	-2.15384800	0.89274300
H	5.22877200	-4.57995800	1.05460900
H	3.05844400	-5.58586100	0.38346200
H	1.23322500	-4.12159500	-0.45406300
H	1.57754000	-1.68442900	-0.62383600
H	-1.81034500	-3.75330700	-1.70662700

Delanzomib deprotonated (de1) in water

B	-2.03824400	-1.90033500	-1.16503000
C	-2.64835700	-0.96839600	0.02337500
H	-2.46244800	-1.39909500	1.01242800
C	-4.16435500	-0.67957200	-0.12468600

C	-5.11005700	-1.86987400	0.12520000
C	-5.07789000	-2.34583800	1.58555100
C	-6.54375800	-1.49783300	-0.28172000
N	-1.88716000	0.29088100	-0.06029300
C	-1.78906000	1.22867200	0.89066000
O	-2.36489100	1.18556000	1.98349800
C	-0.88391200	2.43198800	0.54893300
H	-0.80500100	3.01908800	1.46769800
C	-1.50664400	3.35244400	-0.54220500
H	-1.33571900	2.87297700	-1.51609000
C	-3.00132600	3.57838200	-0.34588000
O	-0.89514300	4.64366300	-0.53037400
N	0.45290400	1.91960600	0.19620500
C	1.58197800	2.64669200	0.12782600
O	1.61925800	3.88586800	0.14420800
C	2.85282800	1.83935200	0.01818700
C	4.06954300	2.49674400	-0.14076200
C	5.21864000	1.71600700	-0.23617100
C	5.10784200	0.33449000	-0.16321200
C	3.84116400	-0.24998900	0.01185600
N	2.73793900	0.50881500	0.09142000
C	3.66461000	-1.72356300	0.10240100
C	4.68974400	-2.55110900	0.58491900
C	4.51185600	-3.93075600	0.66290200
C	3.30853200	-4.50757800	0.25739200
C	2.28114700	-3.69332100	-0.22137300
C	2.45567600	-2.31421300	-0.29467400
O	-2.32609500	-3.28583700	-1.00096500
O	-1.35247700	-1.39135900	-2.15268100
H	-4.33984500	-0.29916600	-1.13891200
H	-4.44209300	0.12970700	0.56240000
H	-4.78335900	-2.70170400	-0.50781100
H	-5.37908700	-1.53880500	2.26354300
H	-4.08308600	-2.68214100	1.88793300
H	-5.76806500	-3.18176300	1.73725000
H	-6.91528600	-0.65780100	0.31655600
H	-7.22798300	-2.33916300	-0.13305500
H	-6.59547700	-1.20514400	-1.33497900
H	-1.37492800	0.37189600	-0.93636300
H	-3.57040600	2.65646500	-0.47374600

H	-3.34825600	4.29939100	-1.08907700
H	-3.20526900	3.98593800	0.64790200
H	0.05751200	4.53265800	-0.33708700
H	0.60855900	0.91678000	0.21717600
H	4.10089000	3.57610600	-0.19164700
H	6.18818400	2.17944600	-0.37468400
H	5.98857200	-0.28624000	-0.25899900
H	5.62449000	-2.12081800	0.92408100
H	5.31175700	-4.55334200	1.04720500
H	3.17153900	-5.58131300	0.31629800
H	1.34321000	-4.13297300	-0.54120700
H	1.65802300	-1.68551700	-0.66902600
H	-1.94540500	-3.76842900	-1.74455200

Warhead model in gas phase

O	-3.24237100	-0.08879200	-1.16806200
O	1.82475400	2.06969200	1.15092500
O	0.61059100	2.46949700	-0.90726100
N	-1.19649600	0.43873100	-0.37771000
C	0.63684100	-0.78179000	0.87197400
C	1.18544400	-1.53006900	-0.35744400
C	-0.11669100	0.55340000	0.60404300
C	-2.82288500	-0.69436300	1.10853800
C	-2.44957300	-0.09112600	-0.23507500
C	2.30108000	-0.76647600	-1.08531600
C	1.67745300	-2.92432200	0.05913600
B	0.83968500	1.76982100	0.24817300
H	1.45625600	-0.57038700	1.56915500
H	-0.04524100	-1.45621000	1.40014000
H	0.35568900	-1.66535800	-1.06142200
H	-0.55376000	0.84498900	1.56921800
H	-1.04691400	0.86291800	-1.28370300
H	3.13971400	-0.55902900	-0.41157800
H	2.68630900	-1.35428800	-1.92363500
H	1.95205000	0.18338300	-1.49902600
H	0.88101700	-3.50321100	0.53588300
H	2.03014400	-3.49252800	-0.80645500
H	2.50816600	-2.85206400	0.77007000
H	2.39927000	2.80911100	0.93227800
H	1.18138600	3.22251900	-1.08614700
H	-3.90927500	-0.71302000	1.17558600
H	-2.45956700	-1.72442700	1.16348300
H	-2.41200400	-0.14732900	1.95932200

Warhead model in diethyl ether

O	-3.23101300	-0.12865700	-1.18310600
O	1.81445000	2.08971000	1.13480900
O	0.58119300	2.46995600	-0.91425400
N	-1.19928000	0.42730000	-0.37894600
C	0.63463400	-0.78233400	0.87241300
C	1.20244000	-1.52211800	-0.35393900
C	-0.11765200	0.55177600	0.60529500
C	-2.83289800	-0.67259100	1.11444800
C	-2.44175400	-0.10229000	-0.23473600
C	2.34079300	-0.76266000	-1.05020500
C	1.67235500	-2.92546800	0.05817500
B	0.82762500	1.77771900	0.24037000
H	1.44476900	-0.57310700	1.58073500
H	-0.05218400	-1.46155200	1.38820900
H	0.38681100	-1.64452900	-1.07693600
H	-0.56217800	0.84297400	1.56568900
H	-1.02826700	0.81481500	-1.29801200
H	3.17065200	-0.58021400	-0.35891000
H	2.73022200	-1.34260800	-1.89211400
H	2.01502600	0.20049800	-1.45176200
H	0.85923200	-3.50224500	0.50869100
H	2.04180400	-3.48558200	-0.80577400
H	2.48604800	-2.86702700	0.78978000
H	2.37179000	2.84085400	0.90452300
H	1.15238600	3.22401900	-1.09567200
H	-3.91851200	-0.64535600	1.19411200
H	-2.51374300	-1.71691300	1.17368700
H	-2.39156700	-0.13767100	1.95622800

Warhead model in methanol

O	-3.22565100	-0.17444900	-1.19107900
O	1.78825900	2.11010700	1.12910800
O	0.54431700	2.47623400	-0.91687700
N	-1.20214100	0.40403200	-0.38369300
C	0.64376300	-0.77791400	0.87299100
C	1.22558100	-1.51144000	-0.35065600
C	-0.12316300	0.54667600	0.60313000
C	-2.83666300	-0.67272600	1.12042200
C	-2.43920500	-0.12706500	-0.23540400
C	2.36016200	-0.74123300	-1.04118400
C	1.70700000	-2.91020000	0.06407200
B	0.80455000	1.78682100	0.23606400
H	1.44890500	-0.55825700	1.58372400
H	-0.03620700	-1.46499000	1.38724600
H	0.41498700	-1.64283000	-1.07797200
H	-0.57642900	0.83269000	1.56026300

H	-1.02209200	0.76816100	-1.31072000
H	3.18697100	-0.55615600	-0.34702800
H	2.75550900	-1.31559200	-1.88424400
H	2.03018900	0.22194800	-1.43906000
H	0.89699100	-3.49446000	0.51058900
H	2.08686800	-3.46620100	-0.79809800
H	2.51619700	-2.84292400	0.79989600
H	2.33086200	2.87309900	0.89990500
H	1.11336200	3.23220000	-1.10128400
H	-3.92351800	-0.67363200	1.18598300
H	-2.48794000	-1.70489300	1.21450900
H	-2.42266100	-0.10220200	1.95238400

Warhead model in water

O	-3.22475100	-0.18707900	-1.19247200
O	1.78124800	2.11426700	1.12822600
O	0.53490800	2.47840800	-0.91682900
N	-1.20295400	0.39769600	-0.38534300
C	0.64647200	-0.77655200	0.87303500
C	1.23154400	-1.50872500	-0.34987200
C	-0.12503900	0.54512300	0.60207200
C	-2.83705600	-0.67339900	1.12228600
C	-2.43913600	-0.13426200	-0.23586600
C	2.36281200	-0.73437700	-1.04119000
C	1.71894000	-2.90498100	0.06633200
B	0.79824400	1.78882100	0.23528700
H	1.45055100	-0.55350200	1.58389700
H	-0.03110400	-1.46580900	1.38749700
H	0.42153100	-1.64442800	-1.07707300
H	-0.58038200	0.82964000	1.55859600
H	-1.02178300	0.75741400	-1.31387800
H	3.18899800	-0.54542400	-0.34734500
H	2.76033000	-1.30777300	-1.88389500
H	2.02897200	0.22723700	-1.43961400
H	0.91147700	-3.49209800	0.51372100
H	2.10102900	-3.46030600	-0.79530400
H	2.52799100	-2.83335300	0.80190500
H	2.32092500	2.87962600	0.89981700
H	1.10235100	3.23571400	-1.10121400
H	-3.92413200	-0.68901000	1.18181700
H	-2.47368100	-1.69921700	1.22918900
H	-2.43608200	-0.08841000	1.95067700

Warhead model (de1) in gas phase

O	-3.25281500	0.08995200	-1.13202000
O	1.90105400	2.12469200	1.11594800

O	0.75199100	2.40696000	-1.00811900
N	-1.17610700	0.54327300	-0.32925600
C	0.62312100	-0.74239500	0.89341300
C	1.12602500	-1.50229800	-0.35189100
C	-0.08230600	0.61674600	0.65112800
C	-2.75917000	-0.73495700	1.05522900
C	-2.40999500	0.01163800	-0.23022100
C	2.29963700	-0.80754300	-1.05713100
C	1.50663100	-2.94293100	0.02644700
B	0.89618300	1.82388000	0.13521200
H	1.46970400	-0.55027200	1.56453700
H	-0.05242500	-1.41841100	1.43742500
H	0.29254100	-1.55376500	-1.06359300
H	-0.48989700	0.93137700	1.62255900
H	-0.98716200	1.12526300	-1.14698300
H	3.15608500	-0.71127700	-0.37921800
H	2.62371400	-1.39036500	-1.92799000
H	2.02890300	0.19340900	-1.39891600
H	0.66185000	-3.47807200	0.47374700
H	1.83736600	-3.51058400	-0.85074900
H	2.32735800	-2.95100900	0.75444200
H	2.42917600	2.83599300	0.73715900
H	-3.83996400	-0.68389100	1.18920300
H	-2.47806500	-1.78746400	0.95293700
H	-2.25283700	-0.33563600	1.93585800

Warhead model (de1) in diethyl ether

O	-3.25395800	0.04281100	-1.14403900
O	1.86746800	2.15364700	1.08833700
O	0.69724700	2.45557900	-1.01473800
N	-1.19296600	0.52473400	-0.34058700
C	0.62026000	-0.73931200	0.87374700
C	1.18465400	-1.47724900	-0.35873300
C	-0.08850100	0.61781100	0.62811100
C	-2.77398800	-0.73369100	1.06677800
C	-2.41944900	-0.01711200	-0.22571400
C	2.45372700	-0.82686200	-0.92783500
C	1.45564200	-2.95052000	-0.01478900
B	0.86364000	1.84007400	0.11702500
H	1.43339400	-0.56094200	1.58797500
H	-0.07838300	-1.42069300	1.37593100
H	0.41499500	-1.45560000	-1.14034000
H	-0.49938000	0.92920100	1.59841300
H	-1.02172700	1.07636900	-1.17921900
H	3.25872000	-0.83382100	-0.18388300
H	2.80749900	-1.37638800	-1.80683900
H	2.28228000	0.20764800	-1.22919000
H	0.54618300	-3.45736200	0.32314000
H	1.84068700	-3.49588700	-0.88260800
H	2.20023300	-3.03405300	0.78589400
H	2.39426200	2.88039500	0.73717200
H	-3.84705300	-0.63395300	1.22991700

H	-2.54705300	-1.79874300	0.96319800
H	-2.23222300	-0.35437400	1.93354800

Warhead model (de1) in methanol

O	-3.24531600	0.02351600	-1.14720300
O	1.85241100	2.17100000	1.08473800
O	0.65194600	2.48544400	-0.99652200
N	-1.19191800	0.50766500	-0.33683700
C	0.63553200	-0.73853000	0.87443200
C	1.19237900	-1.47921400	-0.35913700
C	-0.08675000	0.61209100	0.63041300
C	-2.76906700	-0.76145400	1.06433400
C	-2.41475300	-0.04029100	-0.22181700
C	2.43280200	-0.80653900	-0.96435300
C	1.50630200	-2.93992200	0.00030200
B	0.84342900	1.85092400	0.12557300
H	1.45349700	-0.55294500	1.58120200
H	-0.05425500	-1.42064300	1.38609000
H	0.40620200	-1.48842800	-1.12461200
H	-0.50340100	0.91537900	1.60049200
H	-1.02566700	1.05202500	-1.17994900
H	3.25365200	-0.78396800	-0.23846000
H	2.78150100	-1.35900300	-1.84299900
H	2.23058500	0.22005700	-1.27552900
H	0.61732200	-3.46133200	0.36826000
H	1.88263400	-3.48914000	-0.86849900
H	2.27184300	-2.99298900	0.78322300
H	2.36913800	2.91195400	0.74775700
H	-3.84248000	-0.66946400	1.22881000
H	-2.53439500	-1.82450200	0.95821200
H	-2.23088500	-0.38180700	1.93284300

Warhead model (de1) in water

O	-3.24322000	0.02232100	-1.14663200
O	1.85595800	2.16845100	1.08374300
O	0.64323800	2.49468000	-0.98817400
N	-1.19090100	0.50677700	-0.33495900
C	0.63889800	-0.73813700	0.87508800
C	1.18893900	-1.48197900	-0.35946000
C	-0.08552600	0.61149700	0.63198900
C	-2.76583500	-0.76780900	1.06332400
C	-2.41282800	-0.04315000	-0.22081000
C	2.41715500	-0.80336000	-0.98265300
C	1.51847100	-2.93760600	0.00648300
B	0.84172900	1.85299100	0.12914400
H	1.46074700	-0.55013900	1.57667700
H	-0.04732200	-1.41944300	1.39242400
H	0.39473800	-1.50285100	-1.11640600
H	-0.50261200	0.91326700	1.60238300
H	-1.02589600	1.05248200	-1.17732100

H	3.24508400	-0.76741000	-0.26542600
H	2.76198700	-1.36009000	-1.86008700
H	2.20279700	0.21887700	-1.30035500
H	0.63794100	-3.46309800	0.38860000
H	1.88874700	-3.49000700	-0.86286300
H	2.29356800	-2.97915300	0.78059700
H	2.37024400	2.91236800	0.74947200
H	-3.84010800	-0.68287300	1.22580600
H	-2.52383200	-1.82917700	0.95694100
H	-2.23186600	-0.38550700	1.93328300

Warhead model (de2) in gas phase

O	-3.22684000	0.01659300	-1.13671400
O	1.92317500	2.16218500	1.14433600
O	0.60159000	2.48018500	-0.87085100
N	-1.17096700	0.50977700	-0.30949900
C	0.65749900	-0.75795500	0.89610800
C	1.15397200	-1.49073800	-0.36650700
C	-0.08036900	0.58737300	0.68570100
C	-2.72573700	-0.84196200	1.03795600
C	-2.39119500	-0.06102700	-0.22804400
C	2.23251700	-0.71637700	-1.13723300
C	1.66697500	-2.89127400	0.00599800
B	0.94877400	1.82751100	0.37485200
H	1.51087900	-0.53542100	1.54750600
H	0.01408800	-1.45934700	1.44699200
H	0.29337400	-1.62035900	-1.03586200
H	-0.52208000	0.84947700	1.65613500
H	-1.02861200	1.08457200	-1.13231000
H	3.09927500	-0.51603200	-0.49903400
H	2.57079200	-1.29238400	-2.00748200
H	1.86338100	0.24711900	-1.49165900
H	0.89407800	-3.48069900	0.51133200
H	1.98694100	-3.44799100	-0.88226200
H	2.52722600	-2.82266400	0.68258900
H	1.23130800	3.20145800	-0.97362000
H	-3.80933700	-0.84156700	1.15604500
H	-2.39393700	-1.87825600	0.92468300
H	-2.24984400	-0.43385200	1.93113700

Warhead model (de2) in diethyl ether

O	-3.18479100	-0.08113800	-1.18425900
O	1.84203000	2.25508800	1.15109900
O	0.58501400	2.46309200	-0.90947500
N	-1.16595200	0.47764500	-0.32753800
C	0.65916200	-0.75319200	0.89856100
C	1.19091600	-1.48698600	-0.34892700
C	-0.08347800	0.58854100	0.67178600
C	-2.76147200	-0.78538100	1.06215800
C	-2.38202700	-0.09588100	-0.23660800

C	2.29645800	-0.71895200	-1.08703600
C	1.68431900	-2.88981400	0.03892700
B	0.90521500	1.84925500	0.35302200
H	1.49340400	-0.54485200	1.57860500
H	-0.00362300	-1.44961200	1.42900000
H	0.35058100	-1.61319300	-1.04365500
H	-0.54205700	0.84938400	1.63344800
H	-0.99801500	0.97382000	-1.19457400
H	3.15471500	-0.54371400	-0.42960700
H	2.64716200	-1.28914300	-1.95421500
H	1.94972100	0.25252100	-1.44371900
H	0.89370100	-3.47381900	0.52091400
H	2.02461000	-3.44743600	-0.83973200
H	2.52566400	-2.82751000	0.73890300
H	1.18797700	3.20396900	-1.03575100
H	-3.83675200	-0.67751600	1.20483700
H	-2.53935800	-1.85320200	0.97883500
H	-2.23343900	-0.39583800	1.93228400

Warhead model (de2) in methanol

O	-3.18446100	-0.09853400	-1.19052600
O	1.80249100	2.29161200	1.14845700
O	0.55519300	2.46250300	-0.91753800
N	-1.17301200	0.46380100	-0.32898700
C	0.66217000	-0.75292600	0.89308800
C	1.22554200	-1.46797400	-0.35138900
C	-0.09004600	0.58459100	0.66871500
C	-2.76655400	-0.80730900	1.05724200
C	-2.38563200	-0.11523000	-0.23649200
C	2.37098600	-0.70508600	-1.03167300
C	1.68122000	-2.88731300	0.02173800
B	0.87635200	1.85797200	0.34499800
H	1.48032000	-0.55163200	1.59496100
H	-0.00771200	-1.45761500	1.40118400
H	0.40862700	-1.56408900	-1.07793800
H	-0.55327100	0.83675300	1.63055900
H	-1.00485000	0.94571200	-1.20371300
H	3.21231900	-0.57201200	-0.34259700
H	2.73761300	-1.25859200	-1.90236900
H	2.05957400	0.28362500	-1.37399700
H	0.86168400	-3.46988500	0.45370000
H	2.05129300	-3.42729700	-0.85544100
H	2.49218600	-2.85561200	0.75856700
H	1.14289500	3.21357200	-1.05747400
H	-3.84263200	-0.70745400	1.19869500
H	-2.53619500	-1.87328400	0.97350600
H	-2.24442200	-0.41581900	1.92975300

Warhead model (de2) in water

O	-3.18486100	-0.10111400	-1.19122700
O	1.79358400	2.30003200	1.14872100
O	0.55190400	2.46007700	-0.92107600
N	-1.17392500	0.46124600	-0.32957700
C	0.66283300	-0.75270600	0.89234600
C	1.23150000	-1.46473600	-0.35148000
C	-0.09114000	0.58400300	0.66811400
C	-2.76694100	-0.81078500	1.05650700
C	-2.38623600	-0.11824100	-0.23671500
C	2.38419100	-0.70336100	-1.02116900
C	1.67983400	-2.88715600	0.01877800
B	0.87134200	1.85987400	0.34354200
H	1.47830400	-0.55258100	1.59775800
H	-0.00814100	-1.45883200	1.39674600
H	0.41889500	-1.55509400	-1.08354900
H	-0.55520000	0.83480900	1.62991500
H	-1.00581600	0.94174700	-1.20505900
H	3.22197300	-0.57752900	-0.32627400
H	2.75395800	-1.25414000	-1.89219000
H	2.07922000	0.28832900	-1.36081200
H	0.85520200	-3.46879100	0.44211300
H	2.05461100	-3.42443300	-0.85802600
H	2.48545100	-2.86131200	0.76167100
H	1.13657100	3.21327200	-1.06254500
H	-3.84349200	-0.71448500	1.19665000
H	-2.53274200	-1.87599700	0.97379300
H	-2.24729300	-0.41712100	1.92951700

Deprotonated threonine (Thr1-O^-) in gas phase

O	1.74894900	-1.22813600	-0.65775400
O	-1.47090300	-1.42681800	-0.46544500
O	-1.84429500	0.22974300	0.99750700
N	-0.16328700	1.93938200	-0.51340600
C	1.41769000	-0.06832800	-0.17044500
C	-0.10113700	0.48757200	-0.68364700
C	1.48973700	0.05893500	1.37592400
C	-1.20889100	-0.17998100	0.04026600
H	-0.10415000	0.14390700	-1.72330400
H	2.01476700	0.81275000	-0.57730000
H	1.11670200	1.02334600	1.74001300

H	2.53571900	-0.06461400	1.67740300
H	0.91256900	-0.74853800	1.84004700
H	-0.96993600	2.34577800	-0.97694400
H	0.67609600	2.34693600	-0.91388300
H	-2.09316800	-1.82273500	0.16076100

Deprotonated threonine (Thr1-O$^-$) in diethyl ether

O	1.68069900	-1.30171100	-0.62338400
O	-1.49620900	-1.40894500	-0.42438800
O	-1.81255000	0.28039800	1.01082300
N	-0.14096500	1.92311400	-0.54966900
C	1.38668000	-0.07619700	-0.18779900
C	-0.07287100	0.46559700	-0.68658400
C	1.52875300	0.10393500	1.34361100
C	-1.20021900	-0.17569800	0.06221600
H	-0.12268100	0.11354400	-1.72373600
H	2.02924400	0.73147300	-0.64388800
H	1.24216400	1.10512300	1.68300400
H	2.57403100	-0.07481400	1.61617400
H	0.91862900	-0.63830700	1.87044000
H	-0.97816700	2.29749800	-0.98616300
H	0.65610800	2.33145500	-1.02811100
H	-2.16215300	-1.79153900	0.16689500

Deprotonated threonine (Thr1-O$^-$) in methanol

O	1.69900300	-1.28642800	-0.63776800
O	-1.49877600	-1.40650500	-0.43548300
O	-1.80191800	0.27365700	1.01176800
N	-0.14496400	1.92411500	-0.54328000
C	1.38235700	-0.05877300	-0.17989700
C	-0.06187200	0.46682300	-0.68388400
C	1.50825300	0.08358700	1.35409700
C	-1.19608500	-0.18187400	0.06027100
H	-0.11434900	0.12433400	-1.72405000
H	2.03224700	0.74877100	-0.61095400
H	1.21528900	1.07567300	1.71266000
H	2.54989400	-0.09444300	1.63896400
H	0.89273000	-0.66963600	1.85910500
H	-0.99030700	2.28415900	-0.97691100
H	0.63749300	2.34275100	-1.03731900
H	-2.19063700	-1.78478500	0.12981600

Deprotonated threonine(Thr1-O$^-$) in water

O	1.70788200	-1.27639500	-0.64343000
O	-1.49336400	-1.40977600	-0.43918600
O	-1.80444500	0.26831500	1.00871200

N	-0.14961600	1.92544300	-0.53848100
C	1.38267800	-0.05302200	-0.17711600
C	-0.06166100	0.46893800	-0.68273000
C	1.50201800	0.07717200	1.35833600
C	-1.19538000	-0.18478700	0.05824800
H	-0.11173700	0.12943700	-1.72397600
H	2.03074200	0.76001600	-0.59914200
H	1.20238300	1.06450100	1.72423500
H	2.54338900	-0.09713700	1.64629400
H	0.88847400	-0.68338900	1.85477600
H	-0.99584300	2.28330900	-0.97223200
H	0.63126900	2.34815000	-1.03157500
H	-2.18788700	-1.78994200	0.12178500

Threonine (Thr1-OH) in gas phase

O	1.20274800	-1.62962400	-0.22894300
O	-1.81989000	-1.14257100	-0.30769100
O	-1.50682800	0.48514500	1.21160700
N	0.06435500	1.87679000	-0.79866800
C	1.29805400	-0.20747200	-0.31571900
C	-0.06160400	0.42547600	-0.74009300
C	1.82171100	0.27662900	1.02744200
C	-1.19018600	-0.04743000	0.17674700
H	-0.28326800	0.06126000	-1.74750600
H	2.00769900	0.08119200	-1.10125300
H	1.99166200	1.35464300	1.01167500
H	2.77409100	-0.21519200	1.23458500
H	1.12673900	0.03557600	1.83443700
H	-0.00154900	2.27227600	0.13464700
H	-0.68693200	2.28219200	-1.34628200
H	0.88769400	-1.98449900	-1.06703100
H	-2.48270700	-1.41178300	0.34735400

Threonine (Thr1-OH) in diethyl ether

O	1.28399700	-1.59091700	-0.24994500
O	-1.78888000	-1.19392500	-0.29584500
O	-1.52887800	0.46140800	1.20060300
N	0.00464100	1.86683100	-0.82942700
C	1.30924200	-0.16142500	-0.29716200
C	-0.06963100	0.41183800	-0.74232600
C	1.78049100	0.31610700	1.06763200
C	-1.19210000	-0.07998200	0.17262100
H	-0.26809200	0.02158100	-1.74379200
H	2.01920000	0.17751200	-1.06064400
H	1.86413000	1.40410000	1.09322200
H	2.76762600	-0.10294100	1.27278400
H	1.09793200	-0.00435600	1.85765100
H	-0.06364700	2.28048800	0.09592400
H	-0.77290800	2.23049600	-1.37073100

H	1.08713500	-1.93516100	-1.12841000
H	-2.46177600	-1.47129400	0.34688300

Threonine (Thr1-OH) in methanol

O	1.33435600	-1.56282400	-0.26085300
O	-1.75252000	-1.23789200	-0.28695000
O	-1.55728000	0.44999400	1.18126600
N	-0.03108700	1.86305200	-0.84000800
C	1.31525900	-0.13163600	-0.28411900
C	-0.07332100	0.40661700	-0.74303000
C	1.75229900	0.33977500	1.09414400
C	-1.19352100	-0.10087300	0.16584100
H	-0.25467000	0.00519600	-1.74272400
H	2.02478400	0.23881800	-1.03262700
H	1.78445200	1.42973300	1.14310700
H	2.75628200	-0.03566600	1.30219500
H	1.07801500	-0.02697300	1.87141000
H	-0.12618000	2.28257000	0.08045700
H	-0.80979900	2.20173300	-1.39605100
H	1.19830000	-1.89523400	-1.15568700
H	-2.43431600	-1.51906800	0.34526000

Threonine (Thr1-OH) in water

O	1.34200700	-1.55822400	-0.26254900
O	-1.74537200	-1.24554400	-0.28538000
O	-1.56306300	0.44852900	1.17721000
N	-0.03619600	1.86274200	-0.84107100
C	1.31601400	-0.12701900	-0.28200400
C	-0.07381500	0.40620500	-0.74309100
C	1.74777700	0.34306800	1.09842100
C	-1.19378400	-0.10397700	0.16442900
H	-0.25229600	0.00347100	-1.74265900
H	2.02549500	0.24844700	-1.02796000
H	1.77283400	1.43302500	1.15084300
H	2.75383400	-0.02640100	1.30717600
H	1.07456000	-0.03028400	1.87351500
H	-0.13481400	2.28280800	0.07879100
H	-0.81522900	2.19797100	-1.39881500
H	1.21197300	-1.88858400	-1.15910700
H	-2.42870900	-1.52740600	0.34493200

Level of theory B3LYP/3-21G*

Bortezomib in gas phase

O	0.48067100	-0.44760000	-1.95433200
O	-3.02514800	3.66329100	0.70504400
O	-2.69523100	2.81392100	-1.59158400
O	-2.11465100	-1.61511300	2.81859300
N	-0.70672200	1.13652000	-0.84226600
N	-0.45320000	-0.70083000	1.54661500
N	-1.53947600	-2.13951200	-0.67206300
N	-3.77395800	-3.76879100	-0.26211800
C	-0.11378300	2.78949100	1.00572500
C	0.79275900	3.61985600	0.06725500
C	-1.21844700	1.94473000	0.28526500
C	0.57964200	-0.55975600	0.48172300
C	0.08994800	0.03069900	-0.86895100
C	1.39822400	-1.86158400	0.23273500
C	-0.00730300	4.67149500	-0.73389400
C	1.89723900	4.31436000	0.89576600
C	2.88142200	-1.58199800	0.03865900
C	3.35458600	-1.01108100	-1.15368900
C	3.79861500	-1.87614500	1.05722200
C	-1.54796800	-1.51683700	1.71088600
C	4.71472700	-0.74669800	-1.31780500
C	5.16015800	-1.61049800	0.89229000
C	5.62243900	-1.04420800	-0.29734300
C	-2.09351700	-2.26305800	0.54079300
C	-3.21110200	-3.07602800	0.74813600
C	-2.09478500	-2.82244000	-1.69490800
C	-3.20639700	-3.63481800	-1.48669000
B	-2.39115900	2.86587900	-0.23369400
H	-0.61947500	3.45928900	1.71289600
H	0.52629200	2.11804400	1.59355000
H	1.26664500	2.93019000	-0.64195000
H	-1.63744600	1.26999700	1.04011600
H	1.27118700	0.18311300	0.89402200
H	-1.00545400	1.44674200	-1.76585300
H	1.26831200	-2.50878700	1.10542400
H	0.98173900	-2.35165800	-0.64746800

H	-0.57104000	5.31691000	-0.04807500
H	0.67084600	5.30061500	-1.32177600
H	-0.70780900	4.19360300	-1.42495900
H	2.48956800	3.57997700	1.45407000
H	2.57478300	4.87641000	0.24295000
H	1.45017400	5.01437800	1.61286300
H	-0.20816500	-0.25456300	2.43013300
H	2.63393100	-0.77582400	-1.92658800
H	3.44521200	-2.32483200	1.98053700
H	-3.76204100	4.25824200	0.42261700
H	-3.41909900	3.37699400	-1.95876300
H	5.06797100	-0.30898700	-2.24515900
H	5.85705300	-1.84845700	1.68835500
H	6.67896700	-0.83933300	-0.42871900
H	-3.63859500	-3.15164600	1.73813400
H	-1.63409600	-2.70179300	-2.66707200
H	-3.65403600	-4.18861300	-2.30281500

Bortezomib in diethylether

O	-0.23546600	1.00374400	-1.66116500
O	2.78586000	-2.93722200	1.27931000
O	2.50696400	-2.38667100	-1.10996400
O	1.76408900	2.94722900	2.43662600
N	0.18995400	-0.99170000	-0.62576800
N	0.40711000	1.23328800	1.72563200
N	2.51613100	0.74761900	-0.21022400
N	3.95878000	2.99300000	-1.07437800
C	-0.38125500	-2.78429800	1.06357600
C	-0.96145100	-3.72617500	-0.01802000
C	0.67326200	-1.75272600	0.55392700
C	-0.67222500	0.88781800	0.73406400
C	-0.18528700	0.31033200	-0.62001700
C	-1.56699500	2.12898300	0.46507200
C	0.10622300	-4.67581000	-0.60629400
C	-2.12511000	-4.54892800	0.58045800
C	-2.96644800	1.75315900	0.00158000
C	-3.22502800	1.41662600	-1.33667800

C	-4.02518100	1.72358400	0.92150800
C	1.47520100	2.10016500	1.56867600
C	-4.51455800	1.06689600	-1.74204100
C	-5.31506600	1.37080100	0.51672500
C	-5.56377900	1.04227200	-0.81845500
C	2.35512300	1.95032200	0.37120800
C	3.09264600	3.06178500	-0.04375900
C	3.38501600	0.66833800	-1.24319200
C	4.09386300	1.78525300	-1.67566500
B	2.08113300	-2.38840500	0.22288300
H	0.08306100	-3.38227000	1.85918400
H	-1.21717200	-2.22968800	1.51223300
H	-1.35598900	-3.09531600	-0.82479000
H	0.84720900	-1.02668000	1.35534200
H	-1.26576500	0.11452600	1.22670900
H	0.55552900	-1.31543400	-1.52166400
H	-1.64111700	2.70346700	1.39395000
H	-1.06101000	2.73385200	-0.29252700
H	0.55492600	-5.27788500	0.19461300
H	-0.34951600	-5.35847900	-1.33325400
H	0.89782500	-4.11782400	-1.11315200
H	-2.90640800	-3.89058600	0.97815000
H	-2.57357900	-5.19667000	-0.18181500
H	-1.75832200	-5.18283300	1.39786700
H	0.00423100	1.34712500	2.66064000
H	-2.39881000	1.41603600	-2.03538000
H	-3.83971500	1.98891700	1.95770400
H	3.66971800	-3.34797600	1.10746100
H	3.34204300	-2.86488900	-1.34093800
H	-4.70082600	0.81308800	-2.77980500
H	-6.12306300	1.35746900	1.23970300
H	-6.56452800	0.77201400	-1.13584400
H	2.97505100	4.00719400	0.46868400
H	3.48660100	-0.30458600	-1.70218600
H	4.78483800	1.71700000	-2.50662400

Bortezomib in methanol

O	-0.49301700	1.13279300	-1.55118500
O	3.36522400	-2.27180800	1.32206600
O	3.02960400	-1.67087500	-1.05265900
O	2.01568300	2.17459600	2.75499300
N	0.57569400	-0.62009300	-0.55428200
N	0.09639200	1.33277200	1.84768500
N	3.02184300	1.23463800	0.09214600
N	2.16959900	3.25155900	-1.66383400
C	0.19025000	-2.66170400	0.89841900
C	-0.17412900	-3.56474200	-0.30426000
C	1.09719900	-1.42722900	0.57803700
C	-0.87612300	0.82483300	0.83909700
C	-0.23522200	0.45948000	-0.52381200
C	-2.02038300	1.85147900	0.62944800
C	1.05197400	-4.30982600	-0.87706900
C	-1.25404700	-4.58362500	0.12785900
C	-3.31183200	1.19855800	0.15846700
C	-3.50093400	0.84507700	-1.18728800
C	-4.33512300	0.92398700	1.07830300
C	1.35569600	1.85044200	1.74842900
C	-4.68960000	0.23962300	-1.60094800
C	-5.52368700	0.31540500	0.66543200
C	-5.70508600	-0.02746600	-0.67729900
C	1.95149800	2.01155300	0.37495000
C	1.52491500	3.00707400	-0.50354900
C	3.64306000	1.45079000	-1.08754600
C	3.22954000	2.46238300	-1.95178800
B	2.60839400	-1.79621800	0.26824500
H	0.69953800	-3.26199000	1.66425000
H	-0.74524700	-2.29324000	1.34113600
H	-0.59526800	-2.92178800	-1.08802600
H	1.09373200	-0.79336800	1.46917700
H	-1.29678900	-0.09056900	1.26556400
H	1.03478200	-0.79548100	-1.44872900
H	-2.19962200	2.35107600	1.58617500
H	-1.67590200	2.58964600	-0.09940100

H	1.51435500	-4.92625000	-0.09510300
H	0.74683600	-4.97026100	-1.69746400
H	1.79635600	-3.60777800	-1.25873300
H	-2.14609000	-4.07299400	0.50961200
H	-1.55061000	-5.21364600	-0.71877200
H	-0.86267600	-5.23438800	0.92016700
H	-0.24162900	1.28115800	2.81011900
H	-2.69707200	1.03503900	-1.88629000
H	-4.20316900	1.19570800	2.12080300
H	4.30560400	-2.53032700	1.15218600
H	3.95424900	-1.92728000	-1.29209700
H	-4.82351300	-0.02475000	-2.64415200
H	-6.30581900	0.11350700	1.38869400
H	-6.62753800	-0.49648200	-1.00022700
H	0.66493500	3.62005300	-0.26671200
H	4.47339500	0.80521500	-1.33822800
H	3.75274700	2.64394400	-2.88218500

Bortezomib in water

O	-0.18187300	1.03471300	-1.64127300
O	2.69274600	-3.01584300	1.27709200
O	2.40438200	-2.46557200	-1.11182400
O	1.88128500	2.91637400	2.42545100
N	0.15868900	-0.98186600	-0.61425200
N	0.45555900	1.25157200	1.73464400
N	2.54841500	0.66662000	-0.19692900
N	4.04858100	2.85717800	-1.10248100
C	-0.48277200	-2.75918600	1.06721200
C	-1.09911300	-3.67325300	-0.01845100
C	0.61107300	-1.76625600	0.56437100
C	-0.63484800	0.91958900	0.75174200
C	-0.16467700	0.33263000	-0.60307400
C	-1.50849700	2.17623900	0.48620500
C	-0.07035500	-4.66384500	-0.60833400
C	-2.29637800	-4.44949500	0.57620500
C	-2.90953300	1.82533300	0.00784300

C	-3.16623700	1.52133000	-1.33888600
C	-3.97302200	1.78698900	0.92257800
C	1.55266600	2.07439200	1.56498300
C	-4.45825400	1.19704300	-1.75882000
C	-5.26538000	1.45911600	0.50360200
C	-5.51233700	1.16475100	-0.84039300
C	2.42095200	1.88338500	0.36417600
C	3.18615700	2.96733900	-0.07180700
C	3.41380200	0.54530300	-1.22907200
C	4.15255800	1.63515400	-1.68148000
B	1.99633100	-2.45146300	0.22443100
H	-0.04218300	-3.37978700	1.85903500
H	-1.29606000	-2.17565700	1.52085000
H	-1.46638200	-3.02464400	-0.82431100
H	0.80970500	-1.05096600	1.36953100
H	-1.23897600	0.15988300	1.25161900
H	0.52095200	-1.31734900	-1.50763900
H	-1.58219900	2.74191300	1.42019800
H	-0.98679400	2.78112900	-0.26060700
H	0.34939000	-5.28876700	0.19070700
H	-0.55187000	-5.32260300	-1.34083300
H	0.74671500	-4.13723000	-1.10777700
H	-3.05064400	-3.76148800	0.97627000
H	-2.76971400	-5.07618200	-0.18871700
H	-1.95590400	-5.10044100	1.39158100
H	0.05977000	1.38081400	2.67069300
H	-2.33647100	1.52312100	-2.03341500
H	-3.78864200	2.02390900	1.96565600
H	3.55519800	-3.46560500	1.09164200
H	3.22506600	-2.96398500	-1.35307800
H	-4.64278100	0.96846800	-2.80277000
H	-6.07652400	1.43781300	1.22277100
H	-6.51477100	0.91404800	-1.16818500
H	3.09326800	3.92551900	0.42192100
H	3.49023400	-0.43725400	-1.67165000
H	4.84212700	1.53141200	-2.50969700

Bortezomib (de1) in gas phase

O	0.65865800	-1.16691500	1.61561900
O	0.31376400	3.94799600	-1.38483600
O	1.01525800	3.18561300	0.85569600
O	3.49773000	-2.96107300	-1.29654800
N	-0.05091800	0.71116900	0.47786800
N	1.42290800	-2.02987200	-1.26196400
N	2.69236000	0.30187700	-0.10038400
N	5.27806700	-0.00621000	0.93173600
C	-1.65465400	1.65482000	-1.23782500
C	-2.68057300	2.01473300	-0.13360200
C	-0.18826300	1.55590900	-0.74342400
C	0.15830400	-1.42686500	-0.73040000
C	0.32051300	-0.57944900	0.55575500
C	-0.72472400	-2.64970400	-0.32809500
C	-2.37099700	3.37315200	0.53601100
C	-4.10665400	2.02354300	-0.73056500
C	-2.13461000	-2.29126500	0.10009200
C	-2.38153700	-1.83892600	1.40688800
C	-3.21055200	-2.40951400	-0.78914400
C	2.75928600	-2.01076400	-0.94613000
C	-3.67860700	-1.51927500	1.80861600
C	-4.50997400	-2.08969000	-0.38641200
C	-4.74739800	-1.64520700	0.91589100
C	3.36014700	-0.85082600	-0.22941600
C	4.65653900	-1.00205400	0.26967700
C	3.28006300	1.30975700	0.59771800
C	4.56853500	1.13984200	1.10010100
B	0.45117400	2.98955000	-0.32742100
H	-1.67622100	2.42835300	-2.01663500
H	-1.97952700	0.70350900	-1.69090800
H	-2.62696400	1.22733200	0.62773700
H	0.43868200	1.13055100	-1.53810700
H	-0.32316900	-0.84890800	-1.51800200
H	0.23713200	1.31481600	1.26664400
H	-0.76956700	-3.35011700	-1.17245700

H	-0.18584600	-3.11406500	0.50321100
H	-2.30791800	4.15905300	-0.22643300
H	-3.16085800	3.63339600	1.25405500
H	-1.40891100	3.34174400	1.05688700
H	-4.35147900	1.04623000	-1.16341100
H	-4.85116000	2.25425700	0.04268000
H	-4.18075700	2.78623400	-1.51756700
H	1.22949600	-2.86400200	-1.81901300
H	-1.53637000	-1.72214400	2.07576300
H	-3.02886100	-2.75909700	-1.80091700
H	0.72017200	4.80278600	-1.08591800
H	-3.85510200	-1.16544100	2.81848000
H	-5.33276400	-2.18716100	-1.08666800
H	-5.75412100	-1.39306200	1.23051900
H	5.17670800	-1.93871700	0.12195700
H	2.65175700	2.19476900	0.75867800
H	5.05051400	1.93893600	1.65188300

Bortezomib (de1) in diethylether

O	0.65734800	-1.16436400	1.61843200
O	0.47088300	3.89954400	-1.42247400
O	1.00113300	3.21425600	0.88763800
O	3.47988500	-2.97782600	-1.29699200
N	-0.05874200	0.70874500	0.48571800
N	1.40713800	-2.04123700	-1.25692500
N	2.69861300	0.28939800	-0.09839800
N	5.27620800	-0.05135000	0.94216900
C	-1.63796900	1.69285900	-1.22805500
C	-2.66910700	2.04374500	-0.12679200
C	-0.17336400	1.56672600	-0.72765600
C	0.14671800	-1.42469900	-0.72938900
C	0.31313600	-0.58463300	0.55644600
C	-0.75703600	-2.63930100	-0.34763200
C	-2.36946400	3.39947400	0.55075200
C	-4.09370300	2.04972500	-0.72655100
C	-2.16402000	-2.26954900	0.08285900

C	-2.41817500	-1.87249800	1.40589400
C	-3.23332500	-2.33778100	-0.82087000
C	2.74059400	-2.02722900	-0.94502100
C	-3.71601500	-1.55889200	1.81359300
C	-4.53341500	-2.02350200	-0.41344300
C	-4.77830100	-1.63584300	0.90661200
C	3.35104900	-0.87337600	-0.22433000
C	4.64378800	-1.04033400	0.27994000
C	3.30163500	1.28799400	0.59770800
C	4.58565200	1.10682800	1.10608400
B	0.49475700	2.98690000	-0.31650600
H	-1.65092200	2.47730700	-1.99600700
H	-1.96567000	0.75242800	-1.69948700
H	-2.61261400	1.25457300	0.63231300
H	0.44285900	1.12760100	-1.52271600
H	-0.32118800	-0.83861000	-1.51914000
H	0.20735800	1.29425300	1.29164700
H	-0.80954100	-3.32035400	-1.20583300
H	-0.23118900	-3.13542500	0.47338600
H	-2.37976600	4.20134300	-0.19865100
H	-3.12957700	3.62273200	1.31092800
H	-1.38191100	3.38997100	1.02157600
H	-4.33816100	1.07156900	-1.15700800
H	-4.83963500	2.28222100	0.04399400
H	-4.16784300	2.80884200	-1.51648000
H	1.20220400	-2.86535300	-1.82553500
H	-1.58106700	-1.79753000	2.09004600
H	-3.04875400	-2.65091900	-1.84372300
H	0.87881900	4.76232400	-1.15004400
H	-3.89929000	-1.25553100	2.83836800
H	-5.35187200	-2.08790000	-1.12199500
H	-5.78623900	-1.39601200	1.22574900
H	5.15333200	-1.98343500	0.13800300
H	2.69141200	2.18027200	0.75582400
H	5.07688900	1.90106700	1.65563900

Bortezomib (de1) in methanol

O	-1.39389600	-0.00437300	2.93442000
O	-0.71135900	4.50515100	-0.91765500
O	-2.24715700	3.91780700	0.92818400
O	3.72514100	0.86655400	2.17599800
N	-1.22650600	1.48095800	1.19034600
N	1.56221600	0.19700200	1.91971400
N	2.35237700	-0.68038600	-0.78521700
N	4.98831200	-0.10208900	-1.54904800
C	-0.88836000	1.43599200	-1.34659700
C	-2.32047900	0.87111800	-1.51043600
C	-0.61227500	2.15472200	0.01451200
C	0.35266800	-0.39277600	1.28561400
C	-0.85021000	0.38487800	1.86339400
C	0.23879700	-1.88931700	1.64993400
C	-3.39006200	1.98610000	-1.51786200
C	-2.41011300	0.02964500	-2.80411100
C	-0.85683900	-2.57696900	0.85384700
C	-2.19536100	-2.51886300	1.27490300
C	-0.55169200	-3.24780100	-0.33981400
C	2.82967200	0.36189800	1.45752600
C	-3.20120500	-3.13756300	0.52817200
C	-1.55755400	-3.86589300	-1.08840200
C	-2.88572700	-3.81585300	-0.65375200
C	3.19978100	-0.05673600	0.05690700
C	4.51442500	0.22660300	-0.33134800
C	2.82034800	-1.01483400	-2.01034300
C	4.12821400	-0.72822700	-2.38911000
B	-1.27830300	3.63876300	0.07341300
H	-0.69881700	2.17447600	-2.13712900
H	-0.17868800	0.60956300	-1.49754400
H	-2.51446700	0.21109200	-0.65596000
H	0.47698700	2.20535200	0.15688100
H	0.44070400	-0.27734600	0.21513700
H	-1.95699600	2.10570800	1.57930600
H	1.20202600	-2.36317800	1.44023300
H	0.02757300	-1.94649800	2.72139400

H	-3.23594000	2.64145200	-2.38628300
H	-4.39588100	1.55252400	-1.59063700
H	-3.31581000	2.59396200	-0.61141900
H	-1.69590700	-0.80259700	-2.77973400
H	-3.41801200	-0.38529300	-2.92822900
H	-2.18638800	0.65428600	-3.67874200
H	1.44928800	0.46180700	2.89957300
H	-2.42583100	-1.95898300	2.17297700
H	0.47840300	-3.28099900	-0.67952000
H	-1.16078900	5.38946400	-0.90525200
H	-4.23049200	-3.08909100	0.86643000
H	-1.30575100	-4.38642900	-2.00578100
H	-3.66643900	-4.29783000	-1.23125500
H	5.17106100	0.72387500	0.36765200
H	2.13709300	-1.51466500	-2.68446700
H	4.49378200	-1.00072300	-3.37105700

Bortezomib (de1) in water

O	-1.41959400	-0.01234800	2.92310800
O	-0.70408600	4.51210900	-0.90512100
O	-2.25262000	3.92056500	0.92854000
O	3.70774200	0.88687000	2.17654900
N	-1.23724600	1.48004900	1.18724500
N	1.54794200	0.20833700	1.92000300
N	2.34397200	-0.67291300	-0.78236700
N	4.98273700	-0.09973800	-1.53991200
C	-0.88370500	1.44444900	-1.34761000
C	-2.31451500	0.87931100	-1.52114700
C	-0.61521400	2.15786100	0.01797400
C	0.34275700	-0.39249500	1.28788200
C	-0.86594800	0.38102700	1.85880000
C	0.23721100	-1.88798100	1.65866900
C	-3.38525500	1.99320800	-1.52594400
C	-2.39840000	0.04669700	-2.82094000
C	-0.84728500	-2.58726200	0.85731100
C	-2.18934100	-2.53888900	1.26824600

C	-0.52763700	-3.25998100	-0.33157100
C	2.81552400	0.37577500	1.45856300
C	-3.18436200	-3.16945800	0.51681800
C	-1.52268600	-3.88951600	-1.08507600
C	-2.85436500	-3.84972100	-0.66003800
C	3.18951900	-0.04766300	0.06051300
C	4.50549100	0.23333300	-0.32468200
C	2.81530800	-1.01189200	-2.00491800
C	4.12474200	-0.72790900	-2.38054500
B	-1.27910700	3.64263500	0.07854800
H	-0.69085500	2.18603200	-2.13438800
H	-0.17243200	0.61940300	-1.49813100
H	-2.51136800	0.21361500	-0.67165900
H	0.47312000	2.20621100	0.16730900
H	0.43214500	-0.28172200	0.21691600
H	-1.97015700	2.10290000	1.57401000
H	1.20526500	-2.35587400	1.45815000
H	0.01860400	-1.94216100	2.72878900
H	-3.22945300	2.65247600	-2.39105200
H	-4.39045900	1.55911200	-1.60357600
H	-3.31395700	2.59731000	-0.61676500
H	-1.68139300	-0.78327200	-2.80153900
H	-3.40471300	-0.37034900	-2.95065400
H	-2.17445400	0.67837600	-3.69037700
H	1.43405100	0.47560600	2.89906300
H	-2.43120600	-1.97766300	2.16245800
H	0.50508500	-3.28543500	-0.66379500
H	-1.15130500	5.39753800	-0.89302200
H	-4.21645300	-3.12900500	0.84751900
H	-1.25970800	-4.41117200	-1.99864300
H	-3.62657000	-4.34095200	-1.24114400
H	5.16059300	0.73234800	0.37451300
H	2.13379300	-1.51334200	-2.67954400
H	4.49297300	-1.00423800	-3.36039700

Bortezomib (de2) in gas phase

O	-0.09487800	0.57258500	-1.93936200
O	0.91207500	-4.94647800	0.31297900
O	0.61259700	-3.70757000	-1.80835200
O	2.73908200	0.34201900	2.78942400
N	0.20088100	-1.40057100	-0.81961400
N	0.81537000	0.37015500	1.56021700
N	2.40279200	1.36853600	-0.62427300
N	5.16298400	1.61724600	-0.24479500
C	-1.04460300	-2.51296900	1.12572400
C	-2.34969700	-2.55231900	0.29257400
C	0.27080900	-2.39237000	0.29580000
C	-0.18695100	0.66689000	0.49940200
C	0.00786600	-0.07411100	-0.86151600
C	-0.38696900	2.19128200	0.25892100
C	-2.38375600	-3.75557100	-0.67572000
C	-3.57454100	-2.58838000	1.23593600
C	-1.84462100	2.56005100	0.03294300
C	-2.49771600	2.18652400	-1.15421800
C	-2.56578300	3.26999200	1.00315400
C	2.15558000	0.58461300	1.70814900
C	-3.83676800	2.52034300	-1.35732900
C	-3.90692600	3.60506800	0.79798500
C	-4.54760100	3.23060600	-0.38476100
C	2.96607900	1.08212200	0.55526600
C	4.34622600	1.20920100	0.74636700
C	3.20925500	1.75842500	-1.63385500
C	4.58095900	1.88392100	-1.44184800
B	0.66338200	-3.85134400	-0.33725900
H	-0.94871300	-3.44761900	1.69442600
H	-1.14004300	-1.69172700	1.85877800
H	-2.39939700	-1.63091400	-0.30311500
H	1.06910100	-2.09894700	0.98744400
H	-1.11677400	0.26994100	0.91744600
H	0.31802300	-1.88532700	-1.71807500
H	-0.01365000	2.72525400	1.13848300
H	0.20884200	2.45347700	-0.61552700

H	-2.33110900	-4.69125800	-0.10527400
H	-3.31491800	-3.74775600	-1.25915900
H	-1.52426100	-3.72847400	-1.35084200
H	-3.58187300	-1.72152400	1.90961600
H	-4.50932700	-2.58622600	0.66086400
H	-3.54777200	-3.50013700	1.84671800
H	0.42548700	-0.07133100	2.39136300
H	-1.92433900	1.62532100	-1.88346300
H	-2.07063400	3.56427900	1.92368200
H	0.83134800	-4.58153700	-2.21853100
H	-4.32921300	2.22264700	-2.27720900
H	-4.44919300	4.15506200	1.56019800
H	-5.58943000	3.48666000	-0.54575000
H	4.76757100	0.96798600	1.71199100
H	2.73184800	1.95570100	-2.58523200
H	5.23125700	2.20124600	-2.24864500

Bortezomib (de2) in diethylether

O	-0.28568200	0.48361500	-1.97209000
O	1.98511600	-4.59679100	0.35195000
O	1.31873400	-3.54920200	-1.78086900
O	2.54548000	0.81344100	2.79005200
N	0.42181200	-1.35488100	-0.82324500
N	0.66985000	0.50738900	1.53359400
N	2.13182600	1.70382800	-0.65915200
N	4.78100400	2.47939700	-0.19080400
C	-0.58108300	-2.67635500	1.11630900
C	-1.82946700	-3.03757200	0.27453700
C	0.69573400	-2.30018500	0.29892300
C	-0.35173100	0.63554700	0.46088000
C	-0.03497500	-0.09093600	-0.87877300
C	-0.77919000	2.10902900	0.19433000
C	-1.58730500	-4.26954700	-0.62458700
C	-3.03907400	-3.28159500	1.20601700
C	-2.28120800	2.25671300	0.00322300
C	-2.91024700	1.75056400	-1.14714900

C	-3.06694400	2.89319300	0.97511700
C	1.95408700	0.94089800	1.69227000
C	-4.28939700	1.88247300	-1.31576600
C	-4.44838600	3.02604200	0.80568700
C	-5.06463700	2.52041700	-0.34163400
C	2.69613800	1.55107400	0.54712200
C	4.01904900	1.93930000	0.78090800
C	2.88830600	2.23286900	-1.64565900
C	4.20439500	2.61977600	-1.41088500
B	1.42654200	-3.62623000	-0.31447300
H	-0.31028400	-3.53513800	1.74549600
H	-0.86392500	-1.85728600	1.79774800
H	-2.05814200	-2.17959700	-0.37141000
H	1.39254100	-1.82538600	0.99890400
H	-1.21817500	0.11199400	0.87743800
H	0.60946500	-1.81259000	-1.72136600
H	-0.46858100	2.70999100	1.05414500
H	-0.24529500	2.44132200	-0.69584300
H	-1.35458300	-5.14253300	-0.00096400
H	-2.48500900	-4.49402000	-1.21560500
H	-0.74155800	-4.09207800	-1.29344900
H	-3.23931600	-2.40313200	1.83243600
H	-3.94141100	-3.50224400	0.62241100
H	-2.83895800	-4.13624600	1.86518900
H	0.32823200	0.06271000	2.38459100
H	-2.28881200	1.25261000	-1.88153500
H	-2.59278400	3.29096300	1.86706400
H	1.73744500	-4.34275200	-2.20001400
H	-4.76154700	1.48699100	-2.20883400
H	-5.04020200	3.52297200	1.56683400
H	-6.13600800	2.62180900	-0.47510000
H	4.44522200	1.79965100	1.76413500
H	2.42568400	2.33727000	-2.61839000
H	4.80827800	3.04700300	-2.20184000

Bortezomib (de2) in methanol

O	0.07318600	1.12399500	-1.47936000
O	2.31078400	-3.84877100	1.06118400
O	2.12264700	-2.75435300	-1.14178300
O	2.77444200	1.01311300	2.73127800
N	0.54845300	-0.86536800	-0.44674100
N	0.65801000	1.16609300	1.88526400
N	3.67684700	0.75001000	0.03522800
N	2.84558000	2.96245000	-1.47942100
C	-0.77372900	-2.56705100	0.89882200
C	-1.54012000	-3.02997400	-0.36447200
C	0.61052700	-1.88137600	0.64471400
C	-0.44130700	0.93739800	0.89895700
C	0.08406700	0.39289000	-0.45154600
C	-1.28159600	2.21958600	0.68319200
C	-0.77531200	-4.11849800	-1.14875200
C	-2.94259900	-3.54367700	0.03503800
C	-2.69379300	1.90830000	0.21035500
C	-2.94585600	1.55104900	-1.12470800
C	-3.76832700	1.95409200	1.11085000
C	2.02119300	1.18705000	1.75281600
C	-4.24381900	1.25422500	-1.54608200
C	-5.06757000	1.65597800	0.68972600
C	-5.30942800	1.30585300	-0.64153800
C	2.60838900	1.49306100	0.40328500
C	2.20072900	2.59359300	-0.35175500
C	4.29826400	1.09242400	-1.11317100
C	3.88837100	2.19332600	-1.86398600
B	1.78115100	-2.95416000	0.27283400
H	-0.58682300	-3.43657000	1.54402000
H	-1.43557400	-1.88730800	1.45703900
H	-1.66472300	-2.15427900	-1.01496100
H	0.88853500	-1.37092100	1.57466000
H	-1.08010300	0.17202900	1.34573700
H	1.01590800	-1.20904300	-1.29165700
H	-1.32675500	2.75978100	1.63352100
H	-0.76734200	2.83599200	-0.05810600
H	-0.65716400	-5.01293900	-0.52242500

H	-1.33233500	-4.40312500	-2.05066300
H	0.21630000	-3.75569000	-1.42977600
H	-3.51123800	-2.76889900	0.56387100
H	-3.51223800	-3.84638800	-0.85201500
H	-2.84947200	-4.41481400	0.69639700
H	0.36528500	1.05852100	2.85734200
H	-2.10690100	1.49630000	-1.80674200
H	-3.58721500	2.23140300	2.14446400
H	2.81539000	-3.39773500	-1.43650900
H	-4.42457800	0.98245100	-2.58034500
H	-5.88752000	1.69966000	1.39791900
H	-6.31664000	1.07649300	-0.97046400
H	1.37099400	3.20485700	-0.02760300
H	5.14110400	0.48852200	-1.42585100
H	4.40814600	2.46884400	-2.77333900

Bortezomib (de2) in water

O	0.06506400	1.12373400	-1.48121000
O	2.33569500	-3.83484500	1.05353300
O	2.13227100	-2.74402900	-1.14924500
O	2.76655400	1.02307400	2.73357600
N	0.55004800	-0.86304000	-0.44912500
N	0.65070300	1.16641800	1.88487600
N	3.67475500	0.76210700	0.04085100
N	2.83582100	2.96699500	-1.48017400
C	-0.75909600	-2.57128100	0.90100000
C	-1.52725800	-3.03881700	-0.35951800
C	0.62089500	-1.87875000	0.64204300
C	-0.44717200	0.93550100	0.89755300
C	0.07996100	0.39334000	-0.45298300
C	-1.29148800	2.21526100	0.68330800
C	-0.75977400	-4.12347400	-1.14649600
C	-2.92577300	-3.55953700	0.04475200
C	-2.70264500	1.90034300	0.20965000
C	-2.95415000	1.54722700	-1.12661700
C	-3.77696900	1.93930900	1.11080300
C	2.01349700	1.19362200	1.75408200

C	-4.25140900	1.24817700	-1.54876100
C	-5.07547400	1.63886200	0.68897500
C	-5.31684500	1.29324200	-0.64361200
C	2.60174700	1.50074200	0.40524500
C	2.19065600	2.59790000	-0.35261700
C	4.29715300	1.10520600	-1.10673600
C	3.88317000	2.20235200	-1.86101600
B	1.79651200	-2.94465800	0.26620800
H	-0.56649700	-3.43905700	1.54697500
H	-1.42224500	-1.89404500	1.46054700
H	-1.65840200	-2.16387600	-1.00977700
H	0.89856200	-1.36646000	1.57110200
H	-1.08382200	0.16796100	1.34371400
H	1.01615600	-1.20418900	-1.29570700
H	-1.33880600	2.75330800	1.63471700
H	-0.77915000	2.83532200	-0.05624000
H	-0.63580500	-5.01782700	-0.52113500
H	-1.31824500	-4.41032600	-2.04675300
H	0.22917000	-3.75590700	-1.43061700
H	-3.49633300	-2.78781400	0.57596600
H	-3.49701500	-3.86472900	-0.84038700
H	-2.82609200	-4.43042600	0.70545900
H	0.35656700	1.05965400	2.85664300
H	-2.11534100	1.49725500	-1.80917100
H	-3.59630300	2.21322200	2.14537700
H	2.82624600	-3.38450300	-1.44741700
H	-4.43182800	0.97996000	-2.58401500
H	-5.89526200	1.67737400	1.39762900
H	-6.32351200	1.06235400	-0.97308600
H	1.35786000	3.20638400	-0.03114900
H	5.14401100	0.50531400	-1.41628800
H	4.40340000	2.47815600	-2.76998000

Threonine (Thr1-OH) in gas phase

O	1.90792400	-0.78708000	-0.70789700
O	-0.55299600	-1.73831900	-0.10610400
O	-2.19620300	-0.25174300	0.46557300

N	-0.69775500	1.89165900	-0.33187000
C	1.28507600	0.39327800	-0.13822000
C	-0.17564800	0.56567100	-0.66421800
C	1.32931700	0.43176900	1.39395800
C	-1.10344000	-0.46055600	-0.04332200
H	-0.13012900	0.34196100	-1.74352500
H	1.84467200	1.24396100	-0.53711000
H	0.80853600	1.32857400	1.73961000
H	2.36641000	0.44406000	1.74023800
H	0.84044400	-0.45895400	1.80689600
H	-1.64642100	1.84436600	0.04360700
H	-0.60096900	2.57709800	-1.07867400
H	1.29999600	-1.54303000	-0.47464500
H	-1.17990100	-2.38348300	0.32493600

Threonine (Thr1-OH) in diethylether

O	1.85758300	-0.89862700	-0.62276700
O	-0.64525600	-1.74959400	-0.16700100
O	-2.11008500	-0.17976200	0.63781900
N	-0.62909400	1.92286700	-0.49534300
C	1.28428100	0.35694600	-0.16626800
C	-0.17420600	0.54066700	-0.69738000
C	1.35878900	0.51449600	1.35925900
C	-1.10961200	-0.44621100	-0.01403400
H	-0.13804500	0.29628700	-1.76360700
H	1.86269000	1.15289100	-0.64082300
H	0.96125900	1.49003700	1.65518500
H	2.40020000	0.43886000	1.68492200
H	0.78609600	-0.27723100	1.85751300
H	-1.17987600	1.99203000	0.36654200
H	-1.19710800	2.25617400	-1.27870700
H	1.16924800	-1.59393800	-0.41445400
H	-1.23425800	-2.38670000	0.32695800

Threonine (Thr1-OH) in methanol

O	1.85012200	-0.91272400	-0.61333900
O	-0.65094400	-1.74770500	-0.16747100

O	-2.10475500	-0.17196000	0.64891100
N	-0.62346200	1.92354600	-0.50570800
C	1.28387500	0.35275900	-0.17136900
C	-0.17472100	0.53705000	-0.70038000
C	1.36377800	0.52537700	1.35235900
C	-1.11006300	-0.44518200	-0.00934000
H	-0.14488900	0.28865900	-1.76563900
H	1.86594500	1.14017000	-0.65638400
H	0.97047800	1.50487400	1.64083300
H	2.40578400	0.45019800	1.67672800
H	0.78954300	-0.25920300	1.85968800
H	-1.15780700	1.99954800	0.36660200
H	-1.21682300	2.23808600	-1.27895900
H	1.15088100	-1.59861600	-0.40596400
H	-1.23147000	-2.38944300	0.33063400

Threonine (Thr1-OH) in water

O	1.84941100	-0.91430400	-0.61227500
O	-0.65180800	-1.74753000	-0.16772400
O	-2.10414800	-0.17099700	0.65015600
N	-0.62282500	1.92355600	-0.50674400
C	1.28394100	0.35219100	-0.17184300
C	-0.17469600	0.53667200	-0.70065800
C	1.36425300	0.52653600	1.35172500
C	-1.11014400	-0.44499700	-0.00883500
H	-0.14561200	0.28772800	-1.76579300
H	1.86647100	1.13869200	-0.65788900
H	0.97099300	1.50637300	1.63911900
H	2.40628900	0.45175900	1.67614700
H	0.78998300	-0.25737100	1.85997800
H	-1.15702600	1.99999800	0.36564500
H	-1.21692200	2.23707300	-1.27989500
H	1.14915600	-1.59923500	-0.40476500
H	-1.23131400	-2.38967600	0.33107500

Deprotonated threonine (Thr1-O$^-$) in gas phase

O	3.60468300	-0.00809500	-0.65235900
O	-3.16393700	-0.64348900	-0.19398300
O	-0.94408600	-1.22773200	-0.19038200
N	-0.33795900	1.76313000	0.17343100
C	2.49897200	0.10223700	-0.09800800
C	-1.63084500	1.10971800	0.07437000
C	1.82899800	-0.91018400	0.78187600
C	-1.75915200	-0.24437100	-0.09834700
H	-2.54900800	1.69362500	0.12052600
H	1.84386800	0.98358600	-0.22955800
H	1.65806700	-0.46415400	1.77345200
H	2.42985800	-1.82017100	0.87492600
H	0.80201900	-1.09948300	0.37399700
H	-0.18286400	2.23102000	1.07768300
H	-0.17472100	2.45418200	-0.57185200
H	-3.06263200	-1.63040200	-0.29873800

Deprotonated threonine (Thr1-O$^-$) in diethylether

O	1.51492000	-1.52248200	-0.50312900
O	-1.59706700	-1.30229600	-0.46545400
O	-1.67212200	0.33206300	1.13802400
N	0.02509900	1.94716700	-0.61099200
C	1.38762800	-0.26147100	-0.21370400
C	-0.13168600	0.49921000	-0.76224700
C	1.47972700	0.15813600	1.29669900
C	-1.19495900	-0.06026200	0.06311700
H	-0.19430200	0.10232100	-1.78107600
H	2.04457100	0.50314900	-0.76589700
H	1.24560000	1.21816500	1.42749900
H	2.49295700	-0.05404000	1.66483300
H	0.76636400	-0.43578800	1.87917900
H	-0.56073100	2.48291200	-1.26181100
H	1.01080300	2.19775200	-0.76547900
H	-2.19105700	-1.71659200	0.22097400

Deprotonated threonine (Thr1-O$^-$) in methanol

O	1.43986100	-1.57314200	-0.47141800
O	-1.62209200	-1.27785700	-0.43297400
O	-1.66110500	0.39002800	1.13237900
N	0.06156600	1.93045500	-0.62657700
C	1.36306200	-0.28775000	-0.22675700
C	-0.10272700	0.47909900	-0.76000500
C	1.52237000	0.17000800	1.26884900
C	-1.19096100	-0.04457800	0.07230300
H	-0.19452400	0.08239200	-1.77795600
H	2.05542500	0.42284500	-0.80853400
H	1.36838900	1.24744700	1.37424600
H	2.52785400	-0.10067000	1.61799500
H	0.78691800	-0.35630600	1.88823400
H	-0.55594100	2.45657300	-1.25581600
H	1.03646700	2.18025800	-0.83654900
H	-2.25933200	-1.65863300	0.23417700

Deprotonated threonine (Thr1-O^-) in water

O	1.43323200	-1.57726300	-0.47005800
O	-1.62620700	-1.27438400	-0.43079200
O	-1.65871300	0.39467800	1.13291700
N	0.06510400	1.92934100	-0.62748900
C	1.36092100	-0.28996300	-0.22817100
C	-0.10005300	0.47768800	-0.75966700
C	1.52617000	0.16894300	1.26675200
C	-1.19070900	-0.04287100	0.07328700
H	-0.19476300	0.08176500	-1.77777000
H	2.05637400	0.41615000	-0.81162400
H	1.38093500	1.24769900	1.37179200
H	2.53026500	-0.10885100	1.61414700
H	0.78817000	-0.35101300	1.88851600
H	-0.55272500	2.45470600	-1.25705400
H	1.03975300	2.17844200	-0.83933600
H	-2.26820600	-1.65132200	0.23400500

Level of theory B3LYP/6-31+G(d,p)

Bortezomib in gas phase

O	0.29342700	0.43218100	-2.00134600
O	-3.68758600	-3.10907200	0.74759700
O	-2.83415300	-2.91680900	-1.51784600
O	1.68612400	-2.00108200	2.79269300
N	-1.27142600	-0.72938200	-0.87691000
N	0.65254100	-0.50368500	1.45420900
N	2.05506400	-1.75995400	-0.71382200
N	4.33026400	-3.28890900	-0.12972300
C	-2.92668400	-0.12990300	0.95172300
C	-3.80731400	0.76733800	0.05869200
C	-2.08265800	-1.22769100	0.24005100
C	0.49885900	0.52659600	0.40718100
C	-0.15196800	0.04583500	-0.92011700
C	1.79484600	1.34774500	0.14607400
C	-4.92065600	-0.00627700	-0.66559300
C	-4.40694900	1.90880200	0.89645300
C	1.55198100	2.83811500	-0.00686800
C	1.16733000	3.39718000	-1.23596400
C	1.70582600	3.69257800	1.09642600
C	1.57557400	-1.49560700	1.67294500
C	0.93992100	4.77098100	-1.35257700
C	1.47730300	5.06669800	0.98234200
C	1.09189500	5.61091700	-0.24538900
C	2.42290100	-2.01145900	0.54455100
C	3.56028300	-2.78155000	0.83779200
C	2.81755800	-2.27086700	-1.68315500
C	3.95277800	-3.03205900	-1.38951800
B	-2.93158600	-2.49319300	-0.21546100
H	-3.55337500	-0.62907800	1.70253300
H	-2.24840200	0.52459800	1.51554600
H	-3.15951700	1.21809800	-0.70566200
H	-1.40643900	-1.63290400	1.00309100
H	-0.23357300	1.21627000	0.84088900

H	-1.58150300	-0.98283500	-1.80784800
H	2.45891400	1.18944400	1.00136000
H	2.28454900	0.95124200	-0.74481200
H	-5.57179200	-0.52215700	0.05128200
H	-5.54576800	0.67665500	-1.25150800
H	-4.52310700	-0.75074600	-1.36336700
H	-3.62540200	2.50183200	1.38471900
H	-4.99817500	2.58756200	0.27190800
H	-5.06791300	1.51665300	1.68003900
H	0.19798900	-0.28204400	2.33262800
H	1.03785200	2.74251100	-2.09171400
H	2.01921400	3.27998200	2.05327700
H	-4.18273200	-3.89323900	0.48359300
H	-3.32975700	-3.70565200	-1.76713700
H	0.64516900	5.18598900	-2.31265100
H	1.60749900	5.70969200	1.84853700
H	0.91697900	6.67896000	-0.33962200
H	3.83578300	-2.98118200	1.86813500
H	2.51754800	-2.06342700	-2.70737900
H	4.57133500	-3.44272600	-2.18388700

Bortezomib in diethylether

O	0.73397100	0.55477800	-1.49724300
O	-1.21427400	-4.39176100	1.20105500
O	-0.12061100	-3.92267500	-0.91734300
O	3.22404600	-0.40770200	2.72497700
N	-0.38974900	-1.18447100	-0.58757700
N	1.21396700	0.08248000	1.80892300
N	3.22865300	-1.12435100	-0.37960500
N	4.70852300	1.15411700	-1.09823300
C	-2.52879900	-1.60269400	0.68635100
C	-3.46031400	-1.64422400	-0.54229800
C	-1.03682400	-1.98381400	0.46289100
C	0.26138700	0.71868000	0.87547900
C	0.22353100	0.01863900	-0.50728500
C	0.48566900	2.24524100	0.74665400

C	-3.67660100	-3.06099500	-1.09830200
C	-4.81108900	-0.99971300	-0.18844400
C	-0.74798100	3.00168100	0.29074800
C	-0.98374700	3.27343100	-1.06569300
C	-1.68659600	3.44935800	1.23474700
C	2.55819000	-0.11614300	1.72646700
C	-2.12830200	3.96933600	-1.46644400
C	-2.83312500	4.14308100	0.83771400
C	-3.05799000	4.40495600	-0.51733900
C	3.25853300	-0.02900900	0.38878300
C	4.00690300	1.10176900	0.03854200
C	3.93586700	-1.07089500	-1.51339000
C	4.66595200	0.06428700	-1.87531600
B	-0.77829700	-3.53998200	0.22147400
H	-2.92650400	-2.26210800	1.46924000
H	-2.56524900	-0.58497100	1.09619600
H	-2.99952300	-1.03536200	-1.33260000
H	-0.51684900	-1.78051600	1.40736300
H	-0.71132800	0.56449900	1.34754200
H	-0.29992000	-1.61649200	-1.50038600
H	0.78220200	2.60754600	1.73654000
H	1.31591900	2.42310200	0.05890400
H	-4.09836500	-3.72311300	-0.33183700
H	-4.37587800	-3.03932000	-1.94158100
H	-2.74947000	-3.51302900	-1.46496200
H	-4.68324600	0.03115800	0.16007200
H	-5.47715900	-0.97992500	-1.05818000
H	-5.31738500	-1.56283300	0.60623200
H	0.88647800	0.00586400	2.76659300
H	-0.26908300	2.92762400	-1.80565600
H	-1.51259200	3.26367600	2.29246900
H	-1.04997600	-5.33317400	1.06027400
H	0.06689600	-4.86450900	-1.02045600
H	-2.29190900	4.17230600	-2.52119400
H	-3.54381300	4.48452100	1.58520200
H	-3.94580300	4.94749500	-0.82940600
H	4.04033600	1.97407100	0.68596900

H	3.92134500	-1.95198300	-2.14913700
H	5.22957300	0.09199900	-2.80387100

Bortezomib in methanol

O	0.73085100	0.59254100	-1.46435800
O	-1.24204600	-4.39840800	1.16167600
O	-0.04511600	-3.91069900	-0.89599100
O	3.18842300	-0.70967500	2.64285900
N	-0.36445500	-1.17641300	-0.57847600
N	1.22515800	0.06087700	1.82700400
N	3.37600800	-1.11779900	-0.40588700
N	4.57931700	1.33439000	-1.06040600
C	-2.54150100	-1.61018500	0.62045100
C	-3.43038800	-1.65312000	-0.63956700
C	-1.04151100	-1.98510300	0.44757300
C	0.26270500	0.71040100	0.91384800
C	0.22702700	0.03459100	-0.48030100
C	0.46656300	2.24088900	0.81857800
C	-3.62731700	-3.06991000	-1.20248000
C	-4.79290300	-1.00920000	-0.33223300
C	-0.77362900	2.98723300	0.36373400
C	-0.92424900	3.41571700	-0.96361400
C	-1.80042800	3.27313700	1.27889900
C	2.54006100	-0.24360700	1.69704600
C	-2.07009700	4.10894000	-1.36717800
C	-2.94859100	3.96267900	0.87923600
C	-3.08721500	4.38333000	-0.44777500
C	3.24845500	-0.03745000	0.37554200
C	3.85797900	1.18202000	0.05605800
C	4.09157600	-0.96014600	-1.52488100
C	4.68732800	0.26104200	-1.85369400
B	-0.76403000	-3.53971100	0.20896100
H	-2.96290200	-2.27318500	1.38751900
H	-2.59479300	-0.59434200	1.03254900
H	-2.94434100	-1.04398100	-1.41463700

H	-0.55619400	-1.78014600	1.40944800
H	-0.70520600	0.53607400	1.38772200
H	-0.28675200	-1.58550200	-1.50313200
H	0.74836000	2.58475100	1.81920800
H	1.29912300	2.44990400	0.14370800
H	-4.08204600	-3.72973200	-0.45320800
H	-4.29130700	-3.04693400	-2.07380100
H	-2.68761200	-3.52612800	-1.52978500
H	-4.67750500	0.02159700	0.02102900
H	-5.42862200	-0.99032400	-1.22445900
H	-5.32521400	-1.57274400	0.44484100
H	0.89043500	-0.08593900	2.77396500
H	-0.14061900	3.19789100	-1.68268800
H	-1.69657700	2.96244400	2.31616700
H	-1.06086900	-5.33764500	1.02316300
H	0.12891500	-4.85541700	-1.00421800
H	-2.16641600	4.43504600	-2.39906300
H	-3.72905600	4.17696400	1.60393400
H	-3.97657700	4.92304900	-0.76015500
H	3.76926100	2.04325300	0.71255200
H	4.19866200	-1.82520800	-2.17328800
H	5.26572100	0.36981100	-2.76675500

Bortezomib in water

O	0.72664800	0.59893300	-1.46261000
O	-1.22972200	-4.39776900	1.16691300
O	-0.01899300	-3.90778400	-0.88216600
O	3.18859000	-0.71239400	2.63698100
N	-0.36069600	-1.17465000	-0.57631700
N	1.22564800	0.06628800	1.82802400
N	3.38221700	-1.11015900	-0.40776800
N	4.56916700	1.34956400	-1.06310300
C	-2.54040500	-1.61757300	0.61294900
C	-3.42286600	-1.67015400	-0.65125700
C	-1.03769200	-1.98498100	0.44869400
C	0.26029500	0.71364400	0.91633900

C	0.22568900	0.03861600	-0.47806300
C	0.45882700	2.24482800	0.82269500
C	-3.60936700	-3.09005800	-1.20985300
C	-4.79035700	-1.03253000	-0.35294600
C	-0.78398700	2.98733800	0.36866800
C	-0.92966800	3.42935300	-0.95476900
C	-1.81798800	3.25664800	1.28082900
C	2.53909900	-0.24080800	1.69428800
C	-2.07757600	4.11964500	-1.35773000
C	-2.96825900	3.94301800	0.88175300
C	-3.10191700	4.37723200	-0.44146000
C	3.24643900	-0.03010500	0.37289700
C	3.84789900	1.19325200	0.05296600
C	4.09746000	-0.94852700	-1.52645900
C	4.68519500	0.27648000	-1.85565700
B	-0.74947700	-3.53860200	0.21585800
H	-2.96202800	-2.27988300	1.38044900
H	-2.60073600	-0.60049300	1.02097200
H	-2.93652100	-1.06123200	-1.42635600
H	-0.55841500	-1.77424400	1.41225300
H	-0.70654900	0.53568500	1.39097600
H	-0.28405500	-1.58204900	-1.50182100
H	0.73949200	2.58833400	1.82375000
H	1.29057800	2.45794900	0.14817200
H	-4.06472900	-3.74937800	-0.46055500
H	-4.26869300	-3.07378700	-2.08486200
H	-2.66560800	-3.54280500	-1.53028200
H	-4.68226800	0.00010500	-0.00271900
H	-5.42177900	-1.02035300	-1.24833800
H	-5.32333200	-1.59618400	0.42357200
H	0.89202700	-0.08221800	2.77514700
H	-0.14030100	3.22503900	-1.67158500
H	-1.71833800	2.93535500	2.31524700
H	-1.04203000	-5.33627600	1.03177300
H	0.15867700	-4.85222400	-0.98779200
H	-2.16988400	4.45646900	-2.38653400
H	-3.75441400	4.14417000	1.60404700
H	-3.99298500	4.91448400	-0.75321100
H	3.75310400	2.05431600	0.70875700
H	4.21109600	-1.81324200	-2.17417500

H	5.26363400	0.38838600	-2.76827800

Bortezomib (de1) in gas phase

O	0.76981600	-1.08911800	1.52601900
O	0.48453300	3.99208600	-1.45529600
O	1.20035100	3.28719800	0.75855500
O	3.36808300	-2.84125800	-1.62156900
N	0.02618500	0.78762300	0.46596700
N	1.34017000	-1.87813800	-1.41271600
N	2.89382900	0.33206800	-0.26463100
N	5.08974400	-0.42689600	1.31256300
C	-1.69089700	1.79154900	-1.08449300
C	-2.67548800	2.23399600	0.02019100
C	-0.19715100	1.67076700	-0.69801400
C	0.11258200	-1.36258500	-0.76257700
C	0.35476900	-0.51416700	0.50529300
C	-0.74596600	-2.60459700	-0.38504100
C	-2.42580600	3.66126200	0.53373300
C	-4.12280400	2.09595400	-0.48321700
C	-2.16317400	-2.32030300	0.06624700
C	-2.44430200	-1.98418500	1.40059000
C	-3.23334900	-2.41570100	-0.83607100
C	2.67420500	-1.95190200	-1.10903900
C	-3.75761700	-1.75181100	1.81521500
C	-4.54881900	-2.18526800	-0.42399300
C	-4.81547600	-1.85329100	0.90661400
C	3.33465600	-0.92706000	-0.23375600
C	4.43846100	-1.30541000	0.54489200
C	3.52653900	1.21781700	0.51731400
C	4.62048300	0.83001200	1.29712100
B	0.57325200	3.07770200	-0.36206600
H	-1.74741000	2.50223600	-1.92076500
H	-2.05440900	0.82519600	-1.47082300
H	-2.55102700	1.54372100	0.86647100
H	0.33199100	1.24715300	-1.56140600

H	-0.42167600	-0.76840800	-1.50770100
H	0.35963500	1.32284000	1.27198600
H	-0.78762800	-3.26021600	-1.26458800
H	-0.19846000	-3.13341900	0.40093800
H	-2.50321300	4.38824000	-0.28488400
H	-3.17049700	3.92862000	1.29555200
H	-1.43309900	3.76988800	0.97895700
H	-4.34272100	1.06916600	-0.79721600
H	-4.84303200	2.36957300	0.29859000
H	-4.29992600	2.75752900	-1.34266800
H	1.13108900	-2.61992600	-2.07314500
H	-1.62208400	-1.89524500	2.10474400
H	-3.03458500	-2.68005000	-1.87271700
H	0.97492900	4.78046400	-1.18626100
H	-3.95386700	-1.48748000	2.85094400
H	-5.36187900	-2.26483300	-1.14104100
H	-5.83634000	-1.67059500	1.23129500
H	4.78698200	-2.33354900	0.53955400
H	3.10346200	2.22297900	0.54924800
H	5.13067600	1.55328000	1.92928000

Bortezomib (de1) in diethylether

O	0.83798400	-1.10291400	1.54231000
O	0.54124400	3.94766600	-1.42279600
O	1.06054500	3.34886900	0.87249100
O	3.41122400	-2.82430900	-1.62064300
N	0.04442700	0.75734800	0.50037600
N	1.35474800	-1.93577800	-1.37028700
N	2.96483400	0.36197700	-0.37189200
N	5.04777600	-0.39788100	1.35400200
C	-1.68106600	1.74372300	-1.06154400
C	-2.70008700	2.20679800	0.00279600
C	-0.18975400	1.65538100	-0.65056800
C	0.13443400	-1.38635700	-0.73658500
C	0.39095100	-0.54045300	0.52579100
C	-0.75727400	-2.60894000	-0.36964000

C	-2.52377200	3.67279300	0.42972400
C	-4.13152200	1.98075200	-0.51354200
C	-2.17319000	-2.29724100	0.06868200
C	-2.46876200	-1.99992200	1.40886000
C	-3.23031300	-2.34482400	-0.85340600
C	2.68833800	-1.95855400	-1.09971100
C	-3.78401900	-1.75573600	1.81331300
C	-4.54766600	-2.10463300	-0.45189200
C	-4.82876800	-1.80967300	0.88529100
C	3.33936200	-0.91361700	-0.23912400
C	4.38528900	-1.29258700	0.61592400
C	3.62425300	1.26120700	0.37036800
C	4.65991400	0.88025700	1.22694100
B	0.53448800	3.07607200	-0.28963000
H	-1.73541400	2.42268600	-1.92396600
H	-2.01507800	0.75986100	-1.42405900
H	-2.56297800	1.57467500	0.89171700
H	0.35637500	1.25041300	-1.51183400
H	-0.37750000	-0.78328200	-1.48924500
H	0.33477500	1.27720400	1.32957100
H	-0.80381600	-3.25503500	-1.25433000
H	-0.23187900	-3.16084100	0.41537200
H	-2.64280800	4.34410800	-0.43085500
H	-3.27987100	3.94829600	1.17551600
H	-1.53904800	3.85848300	0.86556500
H	-4.30724700	0.92961000	-0.76570200
H	-4.87404400	2.27446800	0.23840500
H	-4.31779200	2.58075700	-1.41474500
H	1.13771000	-2.67735200	-2.02899600
H	-1.66114000	-1.96144200	2.13421300
H	-3.02213300	-2.58529300	-1.89360900
H	0.98451400	4.76516100	-1.15775200
H	-3.99242100	-1.52805300	2.85503100
H	-5.35166500	-2.15235900	-1.18125100
H	-5.85151800	-1.62474100	1.20141300
H	4.67789200	-2.33455400	0.70107800
H	3.28826300	2.29203900	0.30619800

H	5.18581100	1.62042000	1.82404200

Bortezomib (de1) in methanol

O	-0.81739800	-0.13197300	3.13837600
O	0.66920400	4.24919100	-0.49925100
O	-0.53293900	4.11150000	1.60717700
O	3.95790800	-0.60487900	2.07401900
N	-0.71955200	1.34482100	1.41705200
N	1.72037400	-0.85486700	1.84997500
N	2.43735300	-1.55181100	-0.92973400
N	4.50487200	0.01919100	-1.99969200
C	-1.01114200	1.59007200	-1.07335400
C	-2.51350000	1.95027500	-1.08666400
C	-0.19197200	1.97025500	0.18700100
C	0.39955600	-0.87039900	1.20255300
C	-0.45075900	0.15772700	1.98113200
C	-0.20289500	-2.29414400	1.27097200
C	-2.78135800	3.45480400	-1.25531600
C	-3.23172100	1.16401800	-2.19665500
C	-1.50452700	-2.47015000	0.51567100
C	-2.74481900	-2.31332000	1.15437400
C	-1.49430100	-2.81931300	-0.84480000
C	2.96641100	-0.72832600	1.33664300
C	-3.94110400	-2.49988600	0.45421900
C	-2.68744400	-3.00861200	-1.54785200
C	-3.91666100	-2.84962800	-0.89948500
C	3.18002400	-0.74714400	-0.15709600
C	4.21573100	0.03280400	-0.69437800
C	2.73930500	-1.57831100	-2.23278600
C	3.76641300	-0.79498700	-2.76638500
B	-0.05719100	3.56432700	0.51931300
H	-0.52314300	2.05943900	-1.93882500
H	-0.92958400	0.50489400	-1.23035200
H	-2.94203200	1.63415300	-0.12460900
H	0.82850300	1.59993500	0.02128800
H	0.52160700	-0.57744500	0.16375800

H	-1.13620200	2.03212900	2.04581700
H	0.55303300	-2.96786100	0.85788800
H	-0.34304100	-2.54920600	2.32564300
H	-2.37193700	3.81619400	-2.20771100
H	-3.85954100	3.65508400	-1.25928300
H	-2.33641200	4.04597900	-0.45092900
H	-3.11985900	0.08311000	-2.06012900
H	-4.30384900	1.39311200	-2.21261600
H	-2.82297100	1.42261300	-3.18268500
H	1.70377800	-0.87168200	2.86568200
H	-2.77094500	-2.04871600	2.20776300
H	-0.54257000	-2.95452800	-1.35271900
H	0.73151600	5.18365200	-0.25703000
H	-4.89061800	-2.37786000	0.96807700
H	-2.65691800	-3.28509600	-2.59814900
H	-4.84519300	-3.00010900	-1.44272700
H	4.80870100	0.67577900	-0.05210000
H	2.15180800	-2.23596100	-2.86719700
H	3.99485800	-0.82569000	-3.82797200

Bortezomib (de1) in water

O	-0.81446500	-0.13535900	3.13720200
O	0.67660700	4.24419700	-0.49892500
O	-0.51154200	4.10601300	1.61500600
O	3.95774900	-0.61044200	2.07317000
N	-0.71722400	1.34055900	1.41562800
N	1.72066100	-0.86310600	1.84814500
N	2.43903800	-1.55705800	-0.93100200
N	4.50000800	0.02190700	-2.00167100
C	-1.01240400	1.59253300	-1.07385100
C	-2.51469500	1.95279600	-1.08212300
C	-0.18980900	1.96711500	0.18597900
C	0.40006100	-0.87575500	1.20026500
C	-0.44850100	0.15319100	1.97928600
C	-0.20557200	-2.29815800	1.26671900
C	-2.78309000	3.45893200	-1.23477700

C	-3.23357600	1.17809600	-2.19982400
C	-1.50868100	-2.46871100	0.51268900
C	-2.74761400	-2.31534500	1.15486700
C	-1.50123400	-2.80887000	-0.85009700
C	2.96654200	-0.73397900	1.33515800
C	-3.94549000	-2.49652100	0.45591700
C	-2.69593100	-2.99264900	-1.55198900
C	-3.92382200	-2.83717900	-0.90018000
C	3.17984400	-0.75048800	-0.15847200
C	4.21218600	0.03346600	-0.69595100
C	2.73977400	-1.58168100	-2.23429400
C	3.76376500	-0.79431200	-2.76829300
B	-0.04632800	3.55945000	0.52176500
H	-0.52652200	2.06541000	-1.93857600
H	-0.93115500	0.50810700	-1.23574600
H	-2.94257400	1.62661200	-0.12308100
H	0.82886500	1.59320100	0.01751100
H	0.52299500	-0.58193800	0.16179100
H	-1.13287100	2.02693300	2.04581500
H	0.54818100	-2.97279200	0.85120200
H	-0.34502000	-2.55535700	2.32094900
H	-2.37423500	3.83039400	-2.18350800
H	-3.86128500	3.65896400	-1.23625300
H	-2.33796400	4.04194600	-0.42444000
H	-3.12029100	0.09582500	-2.07568200
H	-4.30593700	1.40618200	-2.21178400
H	-2.82637200	1.44808000	-3.18338000
H	1.70387300	-0.88373000	2.86377000
H	-2.77150700	-2.05816700	2.21016200
H	-0.55068700	-2.94137800	-1.36090500
H	0.74570800	5.17781700	-0.25524700
H	-4.89393400	-2.37771600	0.97249100
H	-2.66763200	-3.26217200	-2.60413000
H	-4.85346300	-2.98359400	-1.44260600
H	4.80353600	0.67821300	-0.05392400
H	2.15400400	-2.24083400	-2.86866100
H	3.99124300	-0.82355000	-3.83009400

Bortezomib (de2) in gas phase

O	0.82324900	0.36758100	-1.48832400
O	-1.47014800	-4.60751800	0.99435200
O	-0.50512100	-3.92696700	-1.13699400
O	3.34461000	0.12520100	2.79592900
N	-0.39186000	-1.30260700	-0.53190900
N	1.29768500	0.10630800	1.81640500
N	3.17655100	-1.09163900	-0.28447400
N	4.95311500	0.92775800	-1.10576900
C	-2.44369500	-1.58251400	0.93374200
C	-3.44719500	-1.33293300	-0.21239100
C	-1.03069700	-2.09283700	0.54808900
C	0.29620800	0.62492100	0.85719900
C	0.26681900	-0.13605600	-0.49605900
C	0.46533400	2.15119900	0.64242300
C	-3.81259200	-2.60374800	-0.99617000
C	-4.71811500	-0.66525200	0.34154900
C	-0.78131800	2.85250000	0.13964900
C	-1.10686800	2.88160000	-1.22635200
C	-1.64378900	3.49341100	1.04302200
C	2.66133100	0.10975300	1.76772100
C	-2.26107200	3.53239100	-1.66936000
C	-2.79944800	4.14431300	0.60252300
C	-3.11225800	4.16613800	-0.75905900
C	3.36206400	0.01904500	0.43375000
C	4.26240600	1.01661200	0.03421600
C	3.87517600	-1.18675600	-1.41830000
C	4.74885300	-0.17941900	-1.83251500
B	-1.03422100	-3.69926800	0.19083100
H	-2.85187100	-2.33676300	1.61930600
H	-2.37737200	-0.64289900	1.50806700
H	-2.98306200	-0.62572400	-0.91542400
H	-0.40097300	-2.01178200	1.44302600
H	-0.65869600	0.44691400	1.35374300
H	-0.27929400	-1.81364000	-1.40337700
H	0.75201300	2.58711200	1.60605300
H	1.29003200	2.30217900	-0.06046300
H	-4.23078700	-3.36683200	-0.32912000

H	-4.55821800	-2.37412600	-1.76961600
H	-2.94165400	-3.04652900	-1.48576200
H	-4.48724400	0.27438500	0.85827200
H	-5.43083400	-0.43929800	-0.46193100
H	-5.22359500	-1.32657800	1.05821800
H	0.97989100	0.08893100	2.77932500
H	-0.45490600	2.37176600	-1.92893800
H	-1.40456700	3.48655400	2.10457800
H	-0.53184300	-4.88390600	-1.26754600
H	-2.49793800	3.53937300	-2.73013800
H	-3.45224100	4.63258200	1.32157900
H	-4.01091000	4.66891300	-1.10659900
H	4.42227100	1.89809200	0.64975700
H	3.72695400	-2.08517100	-2.01245900
H	5.29992700	-0.26652600	-2.76624500

Bortezomib (de2) in diethylether

O	0.85142000	0.37995100	-1.44215000
O	-1.41953300	-4.54657900	1.08269800
O	-0.61120400	-3.90353000	-1.11357400
O	3.33267100	-0.56137200	2.69979800
N	-0.41674100	-1.26556800	-0.52683200
N	1.32065200	-0.00305200	1.82984200
N	3.44152800	-1.20094800	-0.35399300
N	4.69555100	1.19399800	-1.12146600
C	-2.57061000	-1.47220700	0.76047000
C	-3.51201200	-1.36513400	-0.45920600
C	-1.13942300	-2.02630700	0.51848900
C	0.33301200	0.60517100	0.91332600
C	0.27170800	-0.11375700	-0.45917400
C	0.53930900	2.13180200	0.75972500
C	-3.97966200	-2.72771900	-0.99476300
C	-4.72739600	-0.48952900	-0.10830900
C	-0.70017000	2.86781900	0.28743700
C	-0.96639800	3.05098900	-1.07873300
C	-1.61381700	3.38454600	1.22026100

C	2.65559300	-0.22238300	1.72150800
C	-2.11391800	3.72988500	-1.49881900
C	-2.76310500	4.06283100	0.80394500
C	-3.01701000	4.23791500	-0.55985100
C	3.35090800	-0.08864000	0.38392600
C	3.98836300	1.10072900	0.00939800
C	4.14474300	-1.10429400	-1.48683800
C	4.76314000	0.08755000	-1.87320900
B	-1.08319400	-3.63651400	0.21848100
H	-3.03862800	-2.10749700	1.52485700
H	-2.50108800	-0.46630900	1.20186100
H	-2.96000500	-0.85585400	-1.26235900
H	-0.59030400	-1.90515200	1.45981500
H	-0.62099400	0.44401100	1.41689000
H	-0.32806000	-1.74586200	-1.41683600
H	0.83354300	2.51770500	1.74143100
H	1.36527300	2.30413100	0.06542400
H	-4.52788400	-3.28131600	-0.22219300
H	-4.65055600	-2.59379800	-1.85257900
H	-3.14181500	-3.35002400	-1.31813200
H	-4.42141800	0.51340700	0.21107300
H	-5.39699800	-0.37837100	-0.96967800
H	-5.30893000	-0.93891700	0.70778600
H	0.99301300	-0.15290500	2.77820600
H	-0.27347100	2.64441800	-1.80857700
H	-1.41989800	3.26185000	2.28369600
H	-0.58274300	-4.86310200	-1.22760500
H	-2.30202600	3.86093800	-2.56097900
H	-3.45513200	4.45691400	1.54317700
H	-3.90820600	4.76610300	-0.88702200
H	3.93086700	1.98801400	0.63460300
H	4.21934700	-1.99792900	-2.10065800
H	5.32878200	0.14775600	-2.79914400

Bortezomib (de2) in methanol

O	0.76819400	0.52368900	-1.45335700

O	-1.02359600	-4.57244600	1.13390800
O	-0.12369200	-3.90142900	-1.01484600
O	3.24114000	-0.63734200	2.63911000
N	-0.32869000	-1.23538000	-0.53518000
N	1.26118500	0.09974100	1.83328100
N	3.49288400	-1.03763200	-0.38061200
N	4.53259900	1.47757500	-1.07550200
C	-2.48831600	-1.65999600	0.67440000
C	-3.38736900	-1.69398300	-0.58041600
C	-0.99555800	-2.05383500	0.50156100
C	0.26144000	0.68478400	0.91465300
C	0.24820700	-0.02263000	-0.46456800
C	0.39529300	2.22078500	0.78871500
C	-3.65769800	-3.11327800	-1.10522300
C	-4.71675400	-0.97604900	-0.29078400
C	-0.88410000	2.90017500	0.33789400
C	-1.09331800	3.25105500	-1.00450000
C	-1.89182500	3.19761600	1.27041400
C	2.57857800	-0.18155100	1.69645000
C	-2.27704200	3.87929100	-1.40520100
C	-3.07737300	3.82297300	0.87408300
C	-3.27412700	4.16617200	-0.46777000
C	3.28240500	0.04706800	0.37644300
C	3.81157400	1.29825500	0.03706100
C	4.20531100	-0.85327400	-1.49737400
C	4.71952700	0.39880300	-1.84662500
B	-0.73244600	-3.64946300	0.25916900
H	-2.91433800	-2.32099500	1.44105600
H	-2.54313300	-0.64180400	1.08647400
H	-2.87534500	-1.13139200	-1.37443600
H	-0.50376900	-1.84081800	1.45768000
H	-0.69143200	0.47342300	1.40236500
H	-0.21486400	-1.69092300	-1.43453400
H	0.67532100	2.59877400	1.77740800
H	1.20677200	2.45242200	0.09566200
H	-4.16221700	-3.71985400	-0.34224000
H	-4.30782300	-3.07904100	-1.98770100

H	-2.73760300	-3.63011000	-1.39007700
H	-4.55271600	0.06093200	0.02382200
H	-5.35962100	-0.96096800	-1.17862900
H	-5.26850900	-1.48527600	0.51040500
H	0.93005200	-0.07381500	2.77670900
H	-0.32504500	3.02286800	-1.73683000
H	-1.74369200	2.94549100	2.31810500
H	0.01505500	-4.85424200	-1.10530000
H	-2.41863500	4.14512800	-2.44913400
H	-3.84222000	4.04708300	1.61240700
H	-4.19295200	4.65548200	-0.77802500
H	3.65914900	2.16386600	0.67570100
H	4.37702200	-1.72174400	-2.12712700
H	5.29743500	0.52863100	-2.75730400

Bortezomib (de2) in water

O	0.75582400	0.54250400	-1.45169100
O	-0.97099000	-4.57956300	1.12111200
O	-0.08258200	-3.88930400	-1.02576200
O	3.22904600	-0.64945500	2.63365700
N	-0.32514900	-1.22848100	-0.53873000
N	1.25281100	0.10547400	1.83544000
N	3.49387500	-1.02275900	-0.38274000
N	4.51924300	1.50178400	-1.06419500
C	-2.47833000	-1.68324200	0.67077700
C	-3.37827500	-1.72671200	-0.58307800
C	-0.98095400	-2.05861000	0.49582500
C	0.25207900	0.69084300	0.91801200
C	0.24225900	-0.01136700	-0.46367600
C	0.37990500	2.22767200	0.79847200
C	-3.63336200	-3.14862100	-1.10829700
C	-4.71522400	-1.02369100	-0.29176500
C	-0.90044500	2.90320600	0.34434300
C	-1.09280100	3.28456100	-0.99218100
C	-1.92550800	3.16756000	1.26799800
C	2.56831100	-0.18134000	1.69541900
C	-2.27683300	3.91018600	-1.39626800

C	-3.11154700	3.78976900	0.86820600
C	-3.29138300	4.16349200	-0.46793800
C	3.27371800	0.05756100	0.37812600
C	3.79551800	1.31357700	0.04525900
C	4.20863500	-0.82939000	-1.49656700
C	4.71604000	0.42743200	-1.83900600
B	-0.69492800	-3.64932700	0.24843800
H	-2.89626300	-2.34897500	1.43795300
H	-2.54395500	-0.66612800	1.08361000
H	-2.87338000	-1.15815100	-1.37742700
H	-0.49172400	-1.84128300	1.45220900
H	-0.70057600	0.47398000	1.40381600
H	-0.21135500	-1.67727200	-1.44146300
H	0.65397700	2.60259500	1.79003000
H	1.19334200	2.46627400	0.11020500
H	-4.13299200	-3.76021400	-0.34605700
H	-4.28264900	-3.12119700	-1.99154900
H	-2.70759500	-3.65592500	-1.39216700
H	-4.56228900	0.01497700	0.02284200
H	-5.35923100	-1.01567100	-1.17883100
H	-5.26028400	-1.53908000	0.51003300
H	0.92166400	-0.07048200	2.77847500
H	-0.31088600	3.08307100	-1.71792300
H	-1.79089100	2.89220800	2.31164300
H	0.06871200	-4.83982900	-1.12040300
H	-2.40499000	4.20018600	-2.43548800
H	-3.89000300	3.98799700	1.59965200
H	-4.21054300	4.65061500	-0.78058400
H	3.63532800	2.17564800	0.68667900
H	4.38817900	-1.69406000	-2.12932000
H	5.29637600	0.56445400	-2.74703800

Threonine (Thr1-OH) in gas phase

O	1.20340700	-1.63072300	-0.22612200
O	-1.82486900	-1.14297900	-0.30743400

O	-1.50826400	0.48891800	1.21572000
N	0.06415700	1.87597100	-0.80060400
C	1.30062600	-0.20821600	-0.31699900
C	-0.06068000	0.42424300	-0.74240100
C	1.82615000	0.27750300	1.02694700
C	-1.19041500	-0.04769100	0.17433400
H	-0.28366000	0.06045500	-1.75186200
H	2.01128900	0.07935700	-1.10489000
H	2.00018100	1.35666800	1.00817100
H	2.77815300	-0.21847700	1.23555400
H	1.12886700	0.04037200	1.83554900
H	-0.00203700	2.27073200	0.13481500
H	-0.68856500	2.28187500	-1.34902500
H	0.88205600	-1.99223600	-1.06305100
H	-2.49165300	-1.40729800	0.35037100

Threonine (Thr1-OH) in diethylether

O	1.28329100	-1.59306100	-0.24688100
O	-1.79664100	-1.19175600	-0.29609300
O	-1.52757600	0.46486100	1.20682400
N	0.00519800	1.86588000	-0.83161200
C	1.31160600	-0.16311500	-0.29899100
C	-0.06881400	0.41042000	-0.74460100
C	1.78582600	0.31648500	1.06607500
C	-1.19229700	-0.07961800	0.17104100
H	-0.26908600	0.02040700	-1.74793900
H	2.02237900	0.17390000	-1.06551300
H	1.87448800	1.40597200	1.08818800
H	2.77266500	-0.10743800	1.27231700
H	1.10087600	0.00094700	1.85844600
H	-0.06497900	2.27933100	0.09551000
H	-0.77280600	2.22994000	-1.37512100
H	1.08307600	-1.94458300	-1.12548600
H	-2.47352700	-1.46501300	0.34893300

Threonine (Thr1-OH) in methanol

O	1.33201100	-1.56618200	-0.25730500
O	-1.76138600	-1.23454300	-0.28729900
O	-1.55538000	0.45421700	1.18825300
N	-0.02932500	1.86207100	-0.84257100
C	1.31744200	-0.13434900	-0.28615000
C	-0.07225000	0.40521600	-0.74527000
C	1.75862000	0.33969600	1.09188400
C	-1.19374000	-0.09957300	0.16460300
H	-0.25592800	0.00369100	-1.74655800
H	2.02782300	0.23303700	-1.03835800
H	1.79652000	1.43144100	1.13694300
H	2.76245000	-0.04111100	1.30049100
H	1.08204700	-0.02135100	1.87228900
H	-0.12530800	2.28189400	0.07948800
H	-0.80890000	2.20126400	-1.40013000
H	1.19192800	-1.90596800	-1.15249200
H	-2.44775100	-1.51127700	0.34673500

Threonine (Thr1-OH) in water

O	1.33897500	-1.56207000	-0.25887400
O	-1.75534500	-1.24121800	-0.28597500
O	-1.56020200	0.45279800	1.18481000
N	-0.03398600	1.86173600	-0.84359500
C	1.31817500	-0.13016200	-0.28423900
C	-0.07268500	0.40475500	-0.74532100
C	1.75444400	0.34271100	1.09576200
C	-1.19397000	-0.10242100	0.16341400
H	-0.25380600	0.00196400	-1.74648300
H	2.02851700	0.24179200	-1.03415200
H	1.78560400	1.43452700	1.14404700
H	2.76027100	-0.03240700	1.30502700
H	1.07887800	-0.02453500	1.87418100
H	-0.13400100	2.28200100	0.07784300
H	-0.81337100	2.19774900	-1.40336400
H	1.20550900	-1.89997700	-1.15581900
H	-2.44290500	-1.51864800	0.34649800

Deprotonated threonine (Thr1-O^{-}) in gas phase

O	1.86697600	-1.06930000	-0.70428200
O	-1.30804500	-1.52704300	-0.45549100
O	-1.96024400	0.16333100	0.87175300
N	-0.25900200	1.94344200	-0.43806800
C	1.43053100	0.02685000	-0.13592400
C	-0.09595700	0.50435000	-0.66232100
C	1.43437600	0.02359100	1.41873600
C	-1.19771000	-0.24063800	-0.00289700
H	-0.05795100	0.20204900	-1.71694300
H	1.97934800	0.97651900	-0.43967300
H	0.97837800	0.92760300	1.84455200
H	2.47460800	-0.05443200	1.75932000
H	0.90052200	-0.86239100	1.78731000
H	-1.16915800	2.28031800	-0.74208300
H	0.46396200	2.44089700	-0.94928900
H	-1.97363700	-1.93547600	0.12187600

Deprotonated threonine (Thr1-O^{-}) in diethylether

O	1.68559200	-1.30424100	-0.61875900
O	-1.49359000	-1.41665900	-0.41759000
O	-1.82495200	0.28696700	1.00569700
N	-0.14358500	1.92013100	-0.55488900
C	1.38987600	-0.07198000	-0.18675500
C	-0.06868500	0.46173800	-0.68732700
C	1.53318000	0.11266800	1.34474000
C	-1.20073100	-0.17857700	0.05998400
H	-0.11915300	0.10557700	-1.72530200
H	2.03351500	0.73382700	-0.64811500
H	1.25100400	1.11812600	1.68173700
H	2.57907600	-0.07085700	1.61901400
H	0.91879800	-0.62560500	1.87598400
H	-0.99125400	2.28811800	-0.98054600
H	0.64480900	2.33142800	-1.04840100

H	-2.16994000	-1.79315700	0.17120900

Deprotonated threonine (Thr1-O$^-$) in methanol

O	1.70925700	-1.28175800	-0.63846400
O	-1.49494200	-1.41386000	-0.43446100
O	-1.81373500	0.27486000	1.00805000
N	-0.14972500	1.92324100	-0.54443600
C	1.38603100	-0.05245500	-0.17697900
C	-0.05921000	0.46585600	-0.68391200
C	1.50790700	0.08492800	1.35842500
C	-1.19645600	-0.18539000	0.05674500
H	-0.10913100	0.12134800	-1.72573200
H	2.03501200	0.75882100	-0.60644400
H	1.20913700	1.07588000	1.72088800
H	2.55098000	-0.09031200	1.64657300
H	0.89379200	-0.67407000	1.86046200
H	-1.00052500	2.27849000	-0.97526400
H	0.62915400	2.34583500	-1.04402500
H	-2.19460400	-1.79024900	0.12792200

Deprotonated threonine (Thr1-O$^-$) in water

O	1.72493600	-1.26560800	-0.64634900
O	-1.48790900	-1.41909400	-0.43985100
O	-1.81790100	0.26716900	1.00315800
N	-0.15716800	1.92432000	-0.53806500
C	1.38708200	-0.04325400	-0.17267600
C	-0.05844900	0.46769400	-0.68214500
C	1.49905900	0.07632100	1.36483100
C	-1.19572200	-0.19022100	0.05382600
H	-0.10502400	0.12681600	-1.72536200
H	2.03239300	0.77714400	-0.58861200
H	1.18924000	1.05983600	1.73809700
H	2.54192100	-0.09269300	1.65734800
H	0.88890700	-0.69418300	1.85404300
H	-1.01468200	2.27446000	-0.95980700
H	0.61332700	2.35339800	-1.04511100
H	-2.19072100	-1.79798900	0.11717400

Level of theory B3LYP/6-311++G(d,p)

Bortezomib in gas phase

O	0.54476800	0.53757200	-1.66239100
O	-1.22930600	-4.40363400	1.09276700
O	-0.42782600	-3.89614700	-1.13769200
O	3.06345900	-0.18036200	2.80429500
N	-0.57602300	-1.16142100	-0.69303800
N	1.09930900	0.01098100	1.69747500
N	2.83751600	-1.02801300	-0.48998800
N	5.20389200	0.44776600	-0.76375700
C	-2.54529800	-1.59487400	0.83216600
C	-3.61617700	-1.54609000	-0.27429300
C	-1.10038300	-1.98209400	0.40381600
C	0.23402800	0.72048300	0.72750800
C	0.10044200	0.01477300	-0.64858300
C	0.62065500	2.21095200	0.56167500
C	-3.92222500	-2.92158900	-0.88434600
C	-4.90127000	-0.90845100	0.27477400
C	-0.53882100	3.09459000	0.14657600
C	-0.82058700	3.35394000	-1.19970800
C	-1.36314600	3.66886700	1.12287700
C	2.47012600	-0.09652600	1.73802400
C	-1.89752500	4.16420300	-1.55633700
C	-2.44220400	4.47655400	0.76866700
C	-2.71311500	4.72636400	-0.57558600
C	3.25184000	-0.18373100	0.45445000
C	4.44214500	0.54241900	0.32418100
C	3.61103900	-1.13622000	-1.57070400
C	4.78324600	-0.39682000	-1.71010900
B	-0.91244600	-3.52732800	0.09008100
H	-2.85963700	-2.29373200	1.61636700
H	-2.51184900	-0.60587100	1.30335300
H	-3.23819500	-0.89691700	-1.07316700
H	-0.46195700	-1.82018400	1.27901300
H	-0.75494300	0.68475700	1.18871200
H	-0.54117200	-1.59207400	-1.60792700

H	0.99889600	2.55606900	1.52798100
H	1.43283400	2.28421100	-0.16350900
H	-4.25858100	-3.62618200	-0.11588000
H	-4.71689100	-2.84220300	-1.63193900
H	-3.05491200	-3.35593400	-1.38839100
H	-4.71334500	0.09610300	0.66439900
H	-5.66389800	-0.82609200	-0.50497000
H	-5.32176100	-1.51012800	1.08840600
H	0.74561400	0.04767200	2.64654600
H	-0.19818300	2.90428300	-1.96361600
H	-1.14992100	3.49470300	2.17380900
H	-1.10497500	-5.33862400	0.90544800
H	-0.28757100	-4.83585900	-1.28665700
H	-2.09866000	4.35701300	-2.60453900
H	-3.06379400	4.91657900	1.54088100
H	-3.54858900	5.35836800	-0.85550400
H	4.77276000	1.20573900	1.11604500
H	3.28500100	-1.82251700	-2.34570200
H	5.39774200	-0.48765900	-2.60051300

Bortezomib in diethylether

O	0.74106000	0.56740000	-1.47634900
O	-1.26927900	-4.38019500	1.15332400
O	-0.18540800	-3.89946500	-0.96154100
O	3.22497500	-0.47782300	2.71851700
N	-0.40666900	-1.16405100	-0.59410300
N	1.22195000	0.04715200	1.81781100
N	3.20682600	-1.15033800	-0.39150300
N	4.71790400	1.10646900	-1.07832800
C	-2.54329100	-1.57219200	0.68738000
C	-3.47882000	-1.57348000	-0.53696100
C	-1.05886400	-1.96819400	0.44862000
C	0.27581200	0.70437200	0.89319400
C	0.22752400	0.02634600	-0.49987500
C	0.51207600	2.22849100	0.78409200
C	-3.72165100	-2.97383800	-1.11815500
C	-4.81526500	-0.91284400	-0.16593400
C	-0.70915800	2.99247500	0.31298700
C	-0.87451600	3.35132200	-1.02951400

C	-1.70670300	3.35487600	1.22781400
C	2.56259300	-0.16803200	1.73218800
C	-2.00629900	4.05232100	-1.44584700
C	-2.84049700	4.05285800	0.81487300
C	-2.99380500	4.40395600	-0.52632800
C	3.25812500	-0.07170800	0.39256700
C	4.02180300	1.04925000	0.05736100
C	3.90849800	-1.09214000	-1.52481500
C	4.65487500	0.03280000	-1.87057700
B	-0.82787100	-3.52139900	0.18489700
H	-2.94631600	-2.24241900	1.45553500
H	-2.56501600	-0.56553100	1.11976100
H	-3.01107900	-0.95877600	-1.31558500
H	-0.53193700	-1.78629000	1.39141900
H	-0.69682900	0.55277800	1.36196100
H	-0.32583300	-1.58730700	-1.51007700
H	0.79290300	2.58064000	1.78047700
H	1.35314800	2.41048800	0.11403600
H	-4.15544000	-3.64079700	-0.36533600
H	-4.41934200	-2.92330600	-1.95921600
H	-2.80483400	-3.43598400	-1.49315700
H	-4.66783800	0.10774000	0.19870900
H	-5.48349900	-0.86660700	-1.03065600
H	-5.32680300	-1.47968800	0.62013900
H	0.89219000	-0.03729900	2.77243000
H	-0.11496000	3.06929900	-1.74881500
H	-1.59027000	3.09877900	2.27673700
H	-1.12209000	-5.31811000	0.99068500
H	-0.02007200	-4.84164700	-1.07462800
H	-2.11536400	4.32442800	-2.49001300
H	-3.59815100	4.32885300	1.54011400
H	-3.87236900	4.95068700	-0.85021200
H	4.07226200	1.90940800	0.71714900
H	3.87670200	-1.96118900	-2.17364800
H	5.21470500	0.06503900	-2.79945700

Bortezomib in methanol

O	0.73074400	0.60005800	-1.45660400
O	-1.23149300	-4.38633900	1.16396400

O	-0.04553500	-3.89879400	-0.89403700
O	3.20050900	-0.67655800	2.64440400
N	-0.36522700	-1.16634600	-0.57889400
N	1.23019600	0.06257900	1.82876900
N	3.34858500	-1.11441800	-0.40871500
N	4.58054800	1.31157100	-1.07041500
C	-2.53797500	-1.60750100	0.62355300
C	-3.42603600	-1.64901900	-0.63523500
C	-1.03814200	-1.97612000	0.44816400
C	0.26677000	0.71129600	0.91649400
C	0.23072700	0.04129500	-0.48005900
C	0.46666900	2.24095200	0.82519700
C	-3.62420100	-3.06414400	-1.19698700
C	-4.78629400	-1.00401200	-0.32828400
C	-0.77312900	2.98190200	0.36571300
C	-0.91478300	3.41810900	-0.95642900
C	-1.80893900	3.25006200	1.27097700
C	2.54679500	-0.23151600	1.70150400
C	-2.06009200	4.10286300	-1.36427200
C	-2.95643600	3.93077500	0.86655300
C	-3.08578100	4.35978000	-0.45494000
C	3.24576300	-0.03662200	0.37328500
C	3.86935200	1.17072700	0.04970100
C	4.05565400	-0.96865800	-1.53105200
C	4.66557900	0.24010700	-1.86347100
B	-0.75963600	-3.52718400	0.21114100
H	-2.95629500	-2.27341300	1.38705600
H	-2.59479900	-0.59523800	1.03887500
H	-2.94022800	-1.04159800	-1.40878400
H	-0.55375300	-1.76967600	1.40798900
H	-0.69965700	0.53310800	1.38781400
H	-0.28802000	-1.57728400	-1.50102100
H	0.74184100	2.58535000	1.82552400
H	1.30048000	2.45439000	0.15621400
H	-4.08319200	-3.72103700	-0.45043100
H	-4.28348600	-3.04016400	-2.06964700
H	-2.68583800	-3.52249000	-1.51965700
H	-4.66940500	0.02567000	0.02183400
H	-5.42163900	-0.98659800	-1.21854600
H	-5.31743300	-1.56433800	0.44931600

H	0.89673600	-0.07494100	2.77599900
H	-0.12422900	3.21330200	-1.66883000
H	-1.71293200	2.93287100	2.30501700
H	-1.05087200	-5.32175400	1.01788600
H	0.12465600	-4.84201100	-0.99835700
H	-2.14979800	4.43609200	-2.39245100
H	-3.74453800	4.13233600	1.58371500
H	-3.97537000	4.89305100	-0.77097700
H	3.79959200	2.03145700	0.70653400
H	4.14404300	-1.83338500	-2.18006400
H	5.23677800	0.33992900	-2.78022300

Bortezomib in water

O	0.72757800	0.60619300	-1.45417500
O	-1.22336800	-4.38585500	1.16645700
O	-0.02281000	-3.89553400	-0.88246800
O	3.20082900	-0.68227700	2.63867700
N	-0.36186100	-1.16436900	-0.57679700
N	1.23117900	0.06651400	1.83022800
N	3.35457500	-1.10856600	-0.41071800
N	4.57095500	1.32505000	-1.07256500
C	-2.53738100	-1.61315100	0.61669100
C	-3.41960000	-1.66195200	-0.64597400
C	-1.03529500	-1.97569900	0.44891800
C	0.26514900	0.71377400	0.91963000
C	0.22988000	0.04509700	-0.47741500
C	0.46029900	2.24403500	0.83047600
C	-3.60918700	-3.07936900	-1.20488900
C	-4.78399700	-1.02204100	-0.34674900
C	-0.78163200	2.98157200	0.37119300
C	-0.91675600	3.43322400	-0.94641200
C	-1.82578700	3.23149600	1.27214200
C	2.54617400	-0.23083500	1.69908600
C	-2.06369700	4.11534700	-1.35429500
C	-2.97495900	3.90934900	0.86766100
C	-3.09774400	4.35385100	-0.44939500
C	3.24405400	-0.03113200	0.37095200
C	3.85991900	1.18013700	0.04724500
C	4.06110900	-0.95864000	-1.53288600

C	4.66344000	0.25397100	-1.86533200
B	-0.74803700	-3.52590700	0.21619400
H	-2.95626200	-2.27880200	1.38007600
H	-2.60010000	-0.60007400	1.02913200
H	-2.93312300	-1.05444200	-1.41908100
H	-0.55628000	-1.76486000	1.41042000
H	-0.70024700	0.53215200	1.39165000
H	-0.28578800	-1.57343500	-1.49986600
H	0.73381100	2.58772300	1.83149900
H	1.29376400	2.46151400	0.16242300
H	-4.06924200	-3.73608900	-0.45886100
H	-4.26417500	-3.06037100	-2.08090700
H	-2.66743900	-3.53495900	-1.52159200
H	-4.67310300	0.00895100	0.00149500
H	-5.41539400	-1.00957900	-1.23990500
H	-5.31613500	-1.58277200	0.42985900
H	0.89866500	-0.07310100	2.77752100
H	-0.11957300	3.24318600	-1.65557000
H	-1.73529300	2.90203000	2.30280500
H	-1.03723800	-5.32064800	1.02295400
H	0.14967300	-4.83859200	-0.98539200
H	-2.14818500	4.46076700	-2.37888800
H	-3.76967200	4.09646300	1.58142100
H	-3.98871500	4.88487400	-0.76531400
H	3.78436500	2.04064600	0.70365600
H	4.15558800	-1.82290200	-2.18162100
H	5.23458100	0.35703000	-2.78173000

Bortezomib (de1) in gas phase

O	0.79109900	-1.08616000	1.53068200
O	0.44608900	3.96918400	-1.46638300
O	1.16568100	3.28620500	0.74990700
O	3.39734800	-2.83996100	-1.58303700
N	0.01961100	0.77431700	0.47295100
N	1.36131700	-1.90405000	-1.38340100
N	2.89194000	0.34347200	-0.28010500
N	5.09312400	-0.36332500	1.29868900
C	-1.70581800	1.75892000	-1.07998300

C	-2.69221400	2.19689500	0.02277400
C	-0.21276700	1.65068100	-0.69411200
C	0.13232700	-1.37222700	-0.75017100
C	0.36763900	-0.52099000	0.51654000
C	-0.73646300	-2.60582700	-0.37469200
C	-2.45377100	3.62625800	0.53062900
C	-4.13685300	2.04625700	-0.47973200
C	-2.15271300	-2.31334800	0.06843100
C	-2.43846000	-1.97448700	1.39745700
C	-3.21592400	-2.40402800	-0.83731300
C	2.69742200	-1.95500300	-1.08860400
C	-3.74965200	-1.73666000	1.80403600
C	-4.52906500	-2.16799800	-0.43299600
C	-4.80036900	-1.83455900	0.89241900
C	3.34608700	-0.90642200	-0.23262700
C	4.45338700	-1.25888200	0.54770900
C	3.51489200	1.24705500	0.48406700
C	4.61157500	0.88491600	1.26566800
B	0.54330900	3.06389100	-0.36530700
H	-1.76799000	2.46538400	-1.91688600
H	-2.06124700	0.79051600	-1.46294100
H	-2.56255400	1.51164700	0.86964000
H	0.31931400	1.23009200	-1.55430800
H	-0.38947400	-0.77801400	-1.50105800
H	0.34536000	1.31774800	1.27464100
H	-0.77767000	-3.26285600	-1.25100800
H	-0.19778400	-3.13638700	0.41363700
H	-2.54200300	4.34929100	-0.28793300
H	-3.19646500	3.88824800	1.29370000
H	-1.46186600	3.74546200	0.97079800
H	-4.34820900	1.01831100	-0.78883100
H	-4.85770800	2.31703000	0.29981900
H	-4.31832400	2.70198900	-1.34033500
H	1.15356600	-2.66147000	-2.02372700
H	-1.62152800	-1.88869100	2.10527200
H	-3.01366100	-2.66955000	-1.87091900
H	0.92127700	4.76556400	-1.20524000
H	-3.95021800	-1.47087200	2.83652400
H	-5.33763000	-2.24366300	-1.15255400
H	-5.82019600	-1.64713400	1.21091100

H	4.81326200	-2.28135900	0.55648100
H	3.08235000	2.24634500	0.50266200
H	5.11359100	1.62255800	1.88490800

Bortezomib (de1) in diethylether

O	0.84643500	-1.10174400	1.53656800
O	0.53884400	3.93346200	-1.43254300
O	1.05756000	3.34275600	0.86155800
O	3.41449100	-2.81427000	-1.61697000
N	0.04430000	0.75231000	0.50131100
N	1.35697100	-1.93892700	-1.36790000
N	2.96939800	0.36481900	-0.38378000
N	5.01794800	-0.39153600	1.37033200
C	-1.68207900	1.73925400	-1.05793400
C	-2.69662600	2.19923800	0.00979900
C	-0.19158300	1.64760700	-0.65108700
C	0.13807000	-1.38604600	-0.73508200
C	0.39578000	-0.54266300	0.52828300
C	-0.75532300	-2.60572100	-0.37015000
C	-2.51711600	3.66208200	0.43996600
C	-4.12801900	1.97683600	-0.50334500
C	-2.16944500	-2.29259200	0.06715700
C	-2.46548200	-1.99794800	1.40418500
C	-3.22272800	-2.33521800	-0.85441100
C	2.69042700	-1.95683700	-1.10106100
C	-3.77774700	-1.75350100	1.80642700
C	-4.53683300	-2.09420300	-0.45491100
C	-4.81854200	-1.80326200	0.87921700
C	3.33398500	-0.90832800	-0.23874200
C	4.36392900	-1.28580800	0.63103600
C	3.62208100	1.26438000	0.35879600
C	4.64049700	0.88491200	1.23025900
B	0.53297800	3.06619400	-0.29502500
H	-1.73792600	2.42042900	-1.91602000
H	-2.01773600	0.75910300	-1.42314100
H	-2.55836100	1.56530400	0.89462000
H	0.35143600	1.24165600	-1.51096300
H	-0.37076000	-0.78094900	-1.48530600
H	0.33456700	1.27623200	1.32613600

H	-0.80261200	-3.25037800	-1.25364800
H	-0.23291500	-3.15949700	0.41291900
H	-2.64074600	4.33516000	-0.41622100
H	-3.26771800	3.93511700	1.18962100
H	-1.53139000	3.84548000	0.87033200
H	-4.30476700	0.92850000	-0.75832800
H	-4.86696800	2.26811000	0.25032400
H	-4.31548700	2.57914900	-1.40049600
H	1.13754900	-2.68162200	-2.02228500
H	-1.66061000	-1.96252300	2.12994000
H	-3.01427600	-2.57277700	-1.89320100
H	0.97590300	4.75229400	-1.17271200
H	-3.98732600	-1.52869900	2.84653400
H	-5.33881000	-2.13796400	-1.18387800
H	-5.83958900	-1.61772800	1.19377900
H	4.64981900	-2.32714600	0.72643500
H	3.29427500	2.29517600	0.28270400
H	5.16112900	1.62522000	1.82892600

Bortezomib (de1) in methanol

O	-0.80782700	-0.14059300	3.13110600
O	0.67381200	4.22862300	-0.51347100
O	-0.49543000	4.09619300	1.60713100
O	3.95546800	-0.61612700	2.06713700
N	-0.71620300	1.33422700	1.41458900
N	1.72231900	-0.86638900	1.84564600
N	2.44782300	-1.55672300	-0.93125500
N	4.48479200	0.04085400	-1.99705600
C	-1.01743500	1.58706800	-1.07279300
C	-2.52065700	1.93630000	-1.06879700
C	-0.19088600	1.95786000	0.18344400
C	0.40261700	-0.87586400	1.19662200
C	-0.44639200	0.14957000	1.97976900
C	-0.20320000	-2.29671900	1.25740100
C	-2.79994900	3.44158100	-1.18849000
C	-3.23619100	1.17927700	-2.19849000
C	-1.50646000	-2.46354200	0.50599500
C	-2.74111200	-2.31404200	1.14989900
C	-1.50194400	-2.79378700	-0.85579000

C	2.96807100	-0.73583200	1.33575600
C	-3.93733300	-2.49047100	0.45378600
C	-2.69521800	-2.97247400	-1.55452800
C	-3.91853800	-2.82155600	-0.90091500
C	3.17771700	-0.74650300	-0.15896900
C	4.19933500	0.04725100	-0.69464400
C	2.74790600	-1.57765200	-2.23170600
C	3.75983700	-0.78021600	-2.76344900
B	-0.04251100	3.54784100	0.51589800
H	-0.54140400	2.06840400	-1.93588500
H	-0.93121600	0.50615200	-1.24329200
H	-2.94176500	1.58773800	-0.11720500
H	0.82478500	1.58315200	0.01235300
H	0.52494100	-0.57645800	0.16181200
H	-1.12636300	2.02375500	2.04270300
H	0.54730800	-2.97040400	0.83917800
H	-0.34112700	-2.55845500	2.30883300
H	-2.39369100	3.83694500	-2.12649500
H	-3.87802000	3.63268200	-1.18693600
H	-2.36097400	4.00817000	-0.36538700
H	-3.11418100	0.09750900	-2.09572000
H	-4.30857000	1.39859900	-2.20218200
H	-2.83514500	1.47133800	-3.17623200
H	1.70163800	-0.88675400	2.85968300
H	-2.76334000	-2.06344700	2.20484600
H	-0.55443900	-2.92314000	-1.36905700
H	0.74296100	5.16045800	-0.27532700
H	-4.88325100	-2.37517800	0.97177400
H	-2.66977000	-3.23450400	-2.60667800
H	-4.84766500	-2.96383400	-1.44145300
H	4.78368100	0.69548400	-0.05239000
H	2.17086300	-2.24208700	-2.86587700
H	3.98698100	-0.80538000	-3.82386200

Bortezomib (de1) in water

O	-0.80423000	-0.14381900	3.13014400
O	0.67286800	4.22650200	-0.51296600
O	-0.48313800	4.09067400	1.61433300
O	3.95652900	-0.61435800	2.06629900

N	-0.71655600	1.32976500	1.41287500
N	1.72418800	-0.87060100	1.84401000
N	2.45163700	-1.55927900	-0.93166700
N	4.48079300	0.04664300	-1.99959100
C	-1.02135200	1.58861500	-1.07348500
C	-2.52471700	1.93686100	-1.06536700
C	-0.19230200	1.95556900	0.18226500
C	0.40469600	-0.87940300	1.19459800
C	-0.44418500	0.14560100	1.97800300
C	-0.20197500	-2.29989800	1.25391500
C	-2.80535200	3.44270100	-1.17422200
C	-3.24090600	1.18732800	-2.19964600
C	-1.50633000	-2.46400100	0.50377200
C	-2.73993800	-2.31985800	1.15089000
C	-1.50388900	-2.78618800	-0.85996300
C	2.96960000	-0.73626700	1.33437800
C	-3.93735900	-2.49367500	0.45608200
C	-2.69831800	-2.96218900	-1.55742700
C	-3.92063200	-2.81667800	-0.90063100
C	3.17900900	-0.74590400	-0.16020200
C	4.19663300	0.05203100	-0.69678200
C	2.75050800	-1.57932900	-2.23232000
C	3.75870300	-0.77765700	-2.76517000
B	-0.03882000	3.54414200	0.51811100
H	-0.54739500	2.07295900	-1.93600800
H	-0.93459900	0.50839700	-1.24774200
H	-2.94470300	1.58140000	-0.11577500
H	0.82229600	1.57920900	0.00862700
H	0.52739600	-0.57925500	0.16001700
H	-1.12723400	2.01757900	2.04227100
H	0.54755600	-2.97337900	0.83365500
H	-0.33888400	-2.56350400	2.30499900
H	-2.40133400	3.84466800	-2.11035700
H	-3.88351000	3.63304400	-1.16930700
H	-2.36513600	4.00421900	-0.34823500
H	-3.11712400	0.10498200	-2.10535200
H	-4.31356700	1.40515300	-2.20010100
H	-2.84165900	1.48727800	-3.17568400
H	1.70331000	-0.89382700	2.85797700
H	-2.76049800	-2.07598300	2.20746000

H	-0.55730200	-2.91138100	-1.37591900
H	0.74653000	5.15761500	-0.27329500
H	-4.88245000	-2.38301000	0.97657600
H	-2.67452100	-3.21800000	-2.61112100
H	-4.85056100	-2.95710600	-1.44024800
H	4.77885000	0.70303100	-0.05536200
H	2.17572000	-2.24625300	-2.86585000
H	3.98487800	-0.80217400	-3.82577300

Bortezomib (de2) in gas phase

O	0.84921200	0.28655700	-1.47851900
O	-1.80428400	-4.51494900	0.96576500
O	-0.79031600	-3.89781700	-1.15976100
O	3.33055000	-0.29163200	2.78685200
N	-0.48335200	-1.29810100	-0.54490800
N	1.30170400	-0.05734400	1.81061200
N	3.20208000	-1.24265200	-0.33565800
N	4.92728500	0.84398500	-1.06297200
C	-2.56534000	-1.43091500	0.89536300
C	-3.53636100	-1.11670600	-0.26115600
C	-1.18963000	-2.04060200	0.52610900
C	0.34223700	0.55372800	0.86507300
C	0.25757300	-0.18388600	-0.49935100
C	0.61938400	2.06523900	0.67374300
C	-4.00077600	-2.36371000	-1.02714600
C	-4.74922800	-0.33797500	0.27329400
C	-0.57585500	2.85581400	0.18356900
C	-0.88638800	2.94299800	-1.17977600
C	-1.40451300	3.51915100	1.09718300
C	2.65944400	-0.15763500	1.76845200
C	-1.99211000	3.67384700	-1.61085500
C	-2.51179100	4.24944500	0.66838800
C	-2.80938000	4.32949900	-0.69074300
C	3.36256100	-0.16514900	0.43058500
C	4.23745200	0.86691300	0.07655100
C	3.89764000	-1.26989300	-1.47144700
C	4.74663000	-0.22915600	-1.83877600
B	-1.30613900	-3.64070400	0.16869100

H	-3.03220600	-2.14985900	1.57847900
H	-2.43794000	-0.49774000	1.46599700
H	-3.01155000	-0.46266900	-0.96961900
H	-0.56828600	-2.00848900	1.42737500
H	-0.62153400	0.43881800	1.35886300
H	-0.40569400	-1.81456000	-1.41535500
H	0.93532700	2.46561000	1.64151100
H	1.44969000	2.17173700	-0.02721900
H	-4.48984500	-3.07493100	-0.35394400
H	-4.71453200	-2.08528400	-1.81199900
H	-3.16734700	-2.88713900	-1.49788500
H	-4.44420900	0.58784100	0.77197100
H	-5.43701700	-0.07073800	-0.53662200
H	-5.30848800	-0.94085800	0.99863900
H	0.96144700	-0.14706200	2.75974600
H	-0.26172100	2.41630000	-1.89165000
H	-1.17637700	3.46740700	2.15793200
H	-0.87813700	-4.84620300	-1.30129500
H	-2.21831400	3.72658800	-2.67067000
H	-3.13962500	4.75482300	1.39482400
H	-3.67100000	4.89506200	-1.02917000
H	4.37891600	1.72272700	0.72925400
H	3.76808200	-2.14110600	-2.10626400
H	5.29644200	-0.26128200	-2.77491400

Bortezomib (de2) in diethylether

O	0.85229400	0.38038500	-1.43542800
O	-1.44482700	-4.52736000	1.06745800
O	-0.65810400	-3.87686600	-1.13117000
O	3.32772100	-0.60622400	2.68998900
N	-0.43289100	-1.25119700	-0.53197600
N	1.32356000	-0.02510000	1.82904800
N	3.42762400	-1.21996700	-0.36394000
N	4.70156700	1.16148000	-1.10828700
C	-2.58569900	-1.44890700	0.75467000
C	-3.52555700	-1.34121500	-0.46450400
C	-1.15868800	-2.00919600	0.51248300
C	0.34151200	0.59752200	0.91662800
C	0.27140600	-0.11081800	-0.46076600

C	0.55995100	2.12167600	0.77268400
C	-4.01795700	-2.70166600	-0.97783600
C	-4.72301100	-0.43936400	-0.12589400
C	-0.67135500	2.86642700	0.29809400
C	-0.90774000	3.09268600	-1.06332700
C	-1.60733600	3.34426200	1.22461900
C	2.65554000	-0.25652600	1.72118800
C	-2.04720600	3.77712700	-1.48538400
C	-2.74871500	4.02707600	0.80606400
C	-2.97222500	4.24624200	-0.55306900
C	3.34937600	-0.11749000	0.38324800
C	3.99655500	1.06611800	0.01951900
C	4.12933900	-1.12134800	-1.49390400
C	4.75787600	0.06441800	-1.86877000
B	-1.11434400	-3.61679500	0.20877600
H	-3.05614200	-2.07892900	1.51921400
H	-2.51102000	-0.44432700	1.19338100
H	-2.96639800	-0.85495300	-1.27392800
H	-0.61018000	-1.89372600	1.45230100
H	-0.61258400	0.44073600	1.41760600
H	-0.35030800	-1.73279500	-1.42017400
H	0.85120800	2.50117700	1.75582900
H	1.38882100	2.29333600	0.08488400
H	-4.58432200	-3.22671900	-0.20108100
H	-4.67740700	-2.57002000	-1.84256300
H	-3.19247500	-3.34686400	-1.28119500
H	-4.39838500	0.56256600	0.17096000
H	-5.39355000	-0.33422500	-0.98496100
H	-5.30713200	-0.86160900	0.70033400
H	0.99178600	-0.17885200	2.77365800
H	-0.19730600	2.71644100	-1.78974000
H	-1.43724400	3.18781600	2.28573900
H	-0.63477500	-4.83190700	-1.25660100
H	-2.21218500	3.94308500	-2.54452100
H	-3.45899100	4.39147900	1.54034900
H	-3.85774100	4.77891600	-0.88186300
H	3.94896600	1.94682800	0.65200800
H	4.19459300	-2.00852500	-2.11531100
H	5.32240800	0.12689900	-2.79339400

Bortezomib (de2) in methanol

O	0.77166100	0.52615300	-1.44495200
O	-1.03378000	-4.55740700	1.12678700
O	-0.14859700	-3.88407400	-1.02355400
O	3.24655000	-0.62427900	2.64186200
N	-0.33802300	-1.22384600	-0.53703900
N	1.26437300	0.09227600	1.83551000
N	3.45858600	-1.05195800	-0.38406400
N	4.54214200	1.43468200	-1.07984200
C	-2.49403700	-1.64667800	0.67791800
C	-3.39523700	-1.67808000	-0.57368800
C	-1.00371700	-2.04120200	0.50070600
C	0.26764800	0.68280500	0.91751200
C	0.25078100	-0.01841500	-0.46443700
C	0.40764800	2.21702500	0.79645100
C	-3.67622600	-3.09574800	-1.09228500
C	-4.71762300	-0.95091300	-0.28344700
C	-0.86567600	2.90026600	0.33967000
C	-1.05807600	3.26911800	-0.99674600
C	-1.88425800	3.17975400	1.26093500
C	2.58191600	-0.18473700	1.70225100
C	-2.23534900	3.89884700	-1.40239600
C	-3.06331200	3.80605000	0.85918200
C	-3.24287400	4.16804400	-0.47641700
C	3.27869500	0.03121000	0.37621100
C	3.82948000	1.26900700	0.03587100
C	4.16394500	-0.88130900	-1.50378700
C	4.69985000	0.35717400	-1.85354100
B	-0.74771600	-3.63523600	0.25637100
H	-2.91835200	-2.30707700	1.44332100
H	-2.54701100	-0.63098000	1.09133600
H	-2.88225300	-1.12257500	-1.36910000
H	-0.51031000	-1.83101200	1.45421100
H	-0.68514200	0.47291100	1.40194500
H	-0.22546700	-1.68253400	-1.43322400
H	0.68206500	2.59279000	1.78561900
H	1.22294500	2.44823000	0.11066100
H	-4.18613200	-3.69392700	-0.32889000
H	-4.32380600	-3.05976800	-1.97438400

H	-2.76152600	-3.62045800	-1.37332400
H	-4.54569400	0.08488000	0.02418900
H	-5.36237400	-0.93657800	-1.16767600
H	-5.26879000	-1.45128500	0.52119500
H	0.93235000	-0.07186200	2.77871500
H	-0.28150500	3.05439900	-1.72151600
H	-1.74994700	2.91323800	2.30493600
H	-0.01382100	-4.83354300	-1.12174700
H	-2.36417200	4.17979500	-2.44202000
H	-3.83729000	4.01711000	1.58895600
H	-4.15727200	4.65871100	-0.79077700
H	3.70055600	2.13477400	0.67682700
H	4.31218300	-1.75008300	-2.13634500
H	5.27165700	0.47649500	-2.76763900

Bortezomib (de2) in water

O	0.75567800	0.55592000	-1.43949300
O	-0.95578000	-4.56837600	1.10602500
O	-0.09155600	-3.86519100	-1.04261200
O	3.23752600	-0.62305800	2.63846400
N	-0.32974100	-1.21301100	-0.54014700
N	1.25594200	0.10464900	1.84096400
N	3.45842300	-1.02905300	-0.38438800
N	4.52273400	1.46794300	-1.07201800
C	-2.47554300	-1.67975700	0.67616300
C	-3.37915500	-1.72260900	-0.57334400
C	-0.97896400	-2.04801300	0.49405500
C	0.25546600	0.69222600	0.92515200
C	0.24454500	-0.00087500	-0.46065800
C	0.38218200	2.22800000	0.81320700
C	-3.64002600	-3.14340600	-1.09372800
C	-4.71158400	-1.01583900	-0.27875800
C	-0.89383000	2.90112400	0.34877700
C	-1.06213100	3.30958500	-0.97923400
C	-1.93879900	3.13171500	1.25398200
C	2.57217300	-0.17458600	1.70324600
C	-2.24144700	3.93038800	-1.39283600
C	-3.12009100	3.74847800	0.84414900
C	-3.27546700	4.15027100	-0.48317600

C	3.26810100	0.05081300	0.37836800
C	3.80902700	1.29398100	0.04185800
C	4.16452300	-0.84999300	-1.50244400
C	4.69119700	0.39369900	-1.84796700
B	-0.69210100	-3.63506500	0.23971600
H	-2.88771600	-2.34816700	1.44146500
H	-2.54361600	-0.66621300	1.09231800
H	-2.87650400	-1.15813100	-1.36905000
H	-0.48801900	-1.83348000	1.44777600
H	-0.69610700	0.47162700	1.40732000
H	-0.21578600	-1.66225300	-1.44091400
H	0.64488000	2.60072300	1.80678100
H	1.20084900	2.47137300	0.13580500
H	-4.14167500	-3.74958300	-0.33112500
H	-4.28813600	-3.11570300	-1.97565400
H	-2.71793700	-3.65461700	-1.37572700
H	-4.55434400	0.02202300	0.02974200
H	-5.35879200	-1.01019300	-1.16124200
H	-5.25297200	-1.52535100	0.52675400
H	0.92575800	-0.06102700	2.78462300
H	-0.26488600	3.13339800	-1.69190000
H	-1.82404200	2.83434700	2.29195300
H	0.06057800	-4.81129300	-1.14786700
H	-2.35123100	4.24266100	-2.42567800
H	-3.91488000	3.92127600	1.56147900
H	-4.19166000	4.63365700	-0.80348300
H	3.67176400	2.15713300	0.68451000
H	4.32125100	-1.71583900	-2.13692400
H	5.26420600	0.51968300	-2.76037100

Threonine (Thr1-OH) in gas phase

O	1.20315100	-1.62990100	-0.22915800
O	-1.82266000	-1.14020100	-0.30855600
O	-1.50407600	0.48393800	1.21347300
N	0.06454700	1.87627600	-0.79934300
C	1.29812500	-0.20765900	-0.31594500
C	-0.06160200	0.42503100	-0.74036100

C	1.82107600	0.27653200	1.02744300
C	-1.18986100	-0.04728700	0.17714900
H	-0.28378100	0.06042400	-1.74751700
H	2.00790900	0.08118300	-1.10131800
H	1.98993200	1.35470900	1.01209300
H	2.77382800	-0.21445300	1.23482400
H	1.12593100	0.03457300	1.83402200
H	-0.00053900	2.27148000	0.13412900
H	-0.68721100	2.28181000	-1.34624700
H	0.88916600	-1.98482000	-1.06764100
H	-2.48480600	-1.40922700	0.34726000

Threonine (Thr1-OH) in diethylether

O	1.28424500	-1.59126200	-0.24998100
O	-1.79142700	-1.19181100	-0.29682700
O	-1.52660400	0.46054100	1.20217700
N	0.00494300	1.86642600	-0.82980500
C	1.30933300	-0.16170400	-0.29734600
C	-0.06958700	0.41147700	-0.74248500
C	1.78021900	0.31588700	1.06754400
C	-1.19190200	-0.07981700	0.17294500
H	-0.26842100	0.02106200	-1.74382400
H	2.01933100	0.17731600	-1.06078000
H	1.86324000	1.40392300	1.09324500
H	2.76750900	-0.10268100	1.27294500
H	1.09754700	-0.00494500	1.85732500
H	-0.06295500	2.27976200	0.09570300
H	-0.77281300	2.23029200	-1.37071700
H	1.08784000	-1.93555500	-1.12854600
H	-2.46396100	-1.46895300	0.34638900

Threonine (Thr1-OH) in methanol

O	1.33148000	-1.56496700	-0.26030200
O	-1.75724200	-1.23344700	-0.28775300
O	-1.55301500	0.45009500	1.18437600

N	-0.02919000	1.86273600	-0.84037300
C	1.31491200	-0.13359000	-0.28513900
C	-0.07318000	0.40631700	-0.74326000
C	1.75416700	0.33850600	1.09222000
C	-1.19322100	-0.09942300	0.16669900
H	-0.25588200	0.00492100	-1.74266700
H	2.02434700	0.23468600	-1.03480700
H	1.78766200	1.42844500	1.14012900
H	2.75792000	-0.03792800	1.29960400
H	1.08012100	-0.02659900	1.87044700
H	-0.12180000	2.28208500	0.08042700
H	-0.80879400	2.20241400	-1.39463200
H	1.19378200	-1.89793500	-1.15473600
H	-2.43888000	-1.51355600	0.34515800

Threonine (Thr1-OH) in water

O	1.33818300	-1.56088900	-0.26205200
O	-1.75103700	-1.24005700	-0.28661600
O	-1.55750500	0.44847100	1.18108600
N	-0.03351700	1.86262500	-0.84114500
C	1.31555100	-0.12946000	-0.28323400
C	-0.07358400	0.40608200	-0.74340700
C	1.74959500	0.34113700	1.09628600
C	-1.19363200	-0.10200200	0.16521100
H	-0.25372200	0.00366500	-1.74282100
H	2.02512100	0.24349200	-1.03040100
H	1.77653500	1.43112300	1.14758800
H	2.75519100	-0.02983500	1.30460600
H	1.07625500	-0.03015300	1.87225500
H	-0.13020100	2.28215300	0.07916600
H	-0.81275100	2.19949800	-1.39766300
H	1.20695800	-1.89189100	-1.15824000
H	-2.43346800	-1.52116900	0.34505000

Deprotonated threonine (Thr1-O^-) in gas phase

O	1.75243800	-1.22057100	-0.66205600
O	-1.46535500	-1.42938300	-0.46761000
O	-1.84704800	0.22589700	0.99466900
N	-0.16696100	1.94064700	-0.50865400
C	1.41733200	-0.06403400	-0.16908500
C	-0.10102100	0.48962900	-0.68253100
C	1.48743100	0.05337600	1.37802600
C	-1.20822100	-0.18208300	0.03911700
H	-0.10242000	0.14823900	-1.72292400
H	2.01408000	0.82059000	-0.56986800
H	1.11027800	1.01391400	1.74816800
H	2.53351300	-0.06800800	1.67993600
H	0.91277100	-0.75963500	1.83550600
H	-0.97464200	2.34646100	-0.97132200
H	0.67144100	2.35152700	-0.90794900
H	-2.08969900	-1.82649500	0.15584200

Deprotonated threonine (Thr1-O$^-$) in diethylether

O	1.68232600	-1.29933100	-0.62438400
O	-1.49434600	-1.41002900	-0.42515700
O	-1.81339900	0.27898200	1.01004500
N	-0.14238100	1.92350500	-0.54818800
C	1.38672600	-0.07465300	-0.18753800
C	-0.07301000	0.46621100	-0.68644500
C	1.52771100	0.10259300	1.34427800
C	-1.19995700	-0.17650100	0.06184400
H	-0.12226400	0.11492200	-1.72388300
H	2.02895200	0.73448200	-0.64178100
H	1.23755700	1.10206500	1.68571600
H	2.57348500	-0.07322100	1.61692300
H	0.91973800	-0.64293800	1.86892600
H	-0.97996500	2.29750700	-0.98442000
H	0.65428700	2.33280900	-1.02658900
H	-2.16058900	-1.79304600	0.16555800

Deprotonated threonine (Thr1-O$^-$) in methanol

O	1.70185600	-1.28261800	-0.63963200
O	-1.49636400	-1.40781200	-0.43707400

O	-1.80318200	0.27128700	1.01079800
N	-0.14685100	1.92482700	-0.54127100
C	1.38260800	-0.05709900	-0.17918400
C	-0.06227600	0.46783500	-0.68353400
C	1.50638000	0.08144300	1.35547100
C	-1.19575200	-0.18283400	0.05958300
H	-0.11360300	0.12633300	-1.72407900
H	2.03149300	0.75324100	-0.60708500
H	1.20903200	1.07131500	1.71642400
H	2.54839600	-0.09338700	1.64100400
H	0.89314400	-0.67578600	1.85729000
H	-0.99247300	2.28444000	-0.97472600
H	0.63533200	2.34473100	-1.03468800
H	-2.18760900	-1.78760800	0.12800900

Deprotonated threonine (Thr1-O^-) in water

O	1.71086000	-1.27261700	-0.64521400
O	-1.49101700	-1.41116100	-0.44056700
O	-1.80575700	0.26624600	1.00751400
N	-0.15153200	1.92598200	-0.53654700
C	1.38280800	-0.05097500	-0.17632900
C	-0.06179500	0.46974000	-0.68235200
C	1.50019300	0.07503300	1.35964200
C	-1.19505400	-0.18591000	0.05763200
H	-0.11089800	0.13122000	-1.72395700
H	2.03005200	0.76434700	-0.59529200
H	1.19629200	1.06006900	1.72820700
H	2.54190700	-0.09615700	1.64825500
H	0.88892100	-0.68940200	1.85288200
H	-0.99828000	2.28319300	-0.96989500
H	0.62876000	2.35000600	-1.02951300
H	-2.18562400	-1.79222200	0.11972100

Level of theory M06-2X/6-311++G(d,p)

Bortezomib in gas phase

O	0.73900300	1.44170700	-1.67551700
O	0.53180200	-3.79682700	0.92723000
O	1.00321000	-2.92874800	-1.27860500
O	3.54577600	3.18485500	0.36579000
N	0.02431000	-0.44907400	-0.67556800
N	1.47629700	2.45549000	0.89663800
N	2.75136900	-0.13091800	0.97622100
N	4.78934100	-0.54764100	-0.89404100
C	-1.46475900	-1.50415000	1.08439300
C	-2.63658200	-1.73999700	0.12792300
C	-0.07007000	-1.39656600	0.43905600
C	0.29539800	1.62752000	0.65653900
C	0.40177800	0.85270300	-0.66293700
C	-0.87978800	2.62436200	0.51123700
C	-2.38747400	-2.89676300	-0.84123000
C	-3.90483100	-2.00388800	0.94054300
C	-2.21813800	2.05254700	0.11360800
C	-2.53000400	1.87549500	-1.23737100
C	-3.19152700	1.76522600	1.07059600
C	2.76216400	2.26468700	0.48398600
C	-3.79053000	1.42999100	-1.61966400
C	-4.45496200	1.32296000	0.68966700
C	-4.75839300	1.15650200	-0.65719200
C	3.24473100	0.85981900	0.24488900
C	4.26404100	0.65310200	-0.69080600
C	3.29635800	-1.33301500	0.78728600
C	4.30758700	-1.53762400	-0.14198300
B	0.51215700	-2.80077000	-0.00927500
H	-1.41838200	-2.32373700	1.81218500
H	-1.67207700	-0.58997300	1.65142100
H	-2.77802500	-0.82605100	-0.45707800
H	0.61518400	-1.06468800	1.22633400
H	0.12282200	0.95611000	1.49898800
H	0.12459100	-0.85553000	-1.59902100
H	-0.97710200	3.15868100	1.46096900
H	-0.56902500	3.34341300	-0.25204400
H	-2.12057000	-3.80843200	-0.29348900
H	-3.28863100	-3.11253800	-1.42044700
H	-1.59086600	-2.67443300	-1.55566400
H	-4.04634300	-1.24273200	1.71084000

H	-4.79085400	-2.00352600	0.30032500
H	-3.84269800	-2.97884300	1.43571600
H	1.28904100	3.44542500	0.99864600
H	-1.77778400	2.09876200	-1.98735500
H	-2.96565600	1.91197100	2.12266100
H	0.87093400	-4.64690200	0.63807700
H	1.36881100	-3.78101800	-1.52542100
H	-4.01997800	1.30597200	-2.67152500
H	-5.20469500	1.11652000	1.44467300
H	-5.74457200	0.82032400	-0.95522100
H	4.64053900	1.48635100	-1.27217300
H	2.91515700	-2.14985500	1.39410200
H	4.74186600	-2.52179300	-0.28430900

Bortezomib in diethylether

O	0.90928600	0.63417700	-1.29717300
O	-1.41427900	-4.49385800	0.70798100
O	-0.14049100	-3.82818300	-1.23620800
O	3.46110600	-0.18862500	2.75165200
N	-0.05958600	-1.23056400	-0.49173400
N	1.37325600	0.14025900	1.96335400
N	3.35876200	-0.89242000	-0.31201900
N	4.24919900	1.59298500	-1.24608800
C	-2.15347300	-1.58955100	0.90473700
C	-2.98435100	-0.96530300	-0.22011700
C	-0.73530400	-2.07500800	0.49334300
C	0.34782300	0.64644200	1.04654000
C	0.42898600	0.01585300	-0.35828500
C	0.29244100	2.17653900	0.93954000
C	-3.14017300	-1.92122000	-1.40367900
C	-4.35567600	-0.56284400	0.32342500
C	-1.03896000	2.58646900	0.35227100
C	-1.17325300	2.90391700	-0.99958000
C	-2.18200900	2.57386500	1.15672400
C	2.71918600	0.05007200	1.81503700
C	-2.42378000	3.20030800	-1.53560400
C	-3.43212200	2.87043600	0.62433000
C	-3.55649200	3.18062500	-0.72714500
C	3.28014800	0.21192200	0.42452900

C	3.73992400	1.44601900	-0.02712200
C	3.86955400	-0.74212500	-1.53034100
C	4.29894700	0.49715300	-1.99960400
B	-0.76769500	-3.56703500	-0.05300500
H	-2.69146600	-2.45547600	1.30599100
H	-2.09625500	-0.87352000	1.73013300
H	-2.47691300	-0.05482900	-0.56639700
H	-0.11827700	-2.11631600	1.40068900
H	-0.59188700	0.32280800	1.48998400
H	0.08596800	-1.62833000	-1.41218400
H	0.40589200	2.59097500	1.94438600
H	1.11717000	2.52982500	0.31845700
H	-3.57741000	-2.86908100	-1.06910500
H	-3.80390400	-1.49610200	-2.16022300
H	-2.18615900	-2.13795200	-1.89013700
H	-4.25815900	0.09551900	1.19009400
H	-4.93613700	-0.03336900	-0.43615200
H	-4.92120100	-1.44931000	0.62888000
H	1.07881600	0.02895900	2.92646800
H	-0.29402200	2.89491400	-1.63292900
H	-2.08987900	2.33209200	2.21220000
H	-1.40388300	-5.39614000	0.37656300
H	-0.17001100	-4.73573100	-1.55197100
H	-2.51280600	3.44260300	-2.58825800
H	-4.30832400	2.85731100	1.26231800
H	-4.52968600	3.40809600	-1.14615000
H	3.69277200	2.32425400	0.60905100
H	3.94001900	-1.62674800	-2.15325100
H	4.69933000	0.59939900	-3.00202900

Bortezomib in methanol

O	0.85476200	0.70218300	-1.27053900
O	-1.34553500	-4.52362800	0.63140300
O	0.03262400	-3.81758000	-1.22661600
O	3.40089400	-0.60244500	2.63144300
N	-0.03201400	-1.21585200	-0.49424200
N	1.36137200	0.10043000	1.97440200
N	3.43827100	-0.82304300	-0.41855400
N	4.13015400	1.80179900	-1.10349600

C	-2.15696200	-1.64023700	0.82501900
C	-2.97611500	-1.07594700	-0.33927500
C	-0.71489600	-2.08745100	0.46438700
C	0.32754500	0.63590000	1.08283100
C	0.40706700	0.04574600	-0.33927400
C	0.25896700	2.16689400	1.03255700
C	-3.06665800	-2.06437900	-1.50279300
C	-4.37438900	-0.70469500	0.15552200
C	-1.06927800	2.59638500	0.45104400
C	-1.16973600	3.07367600	-0.85605900
C	-2.23598500	2.46523400	1.20998300
C	2.67662400	-0.13318700	1.76704400
C	-2.40842300	3.41379000	-1.39403100
C	-3.47519700	2.80347300	0.67568600
C	-3.56478700	3.27683000	-0.63090900
C	3.24489500	0.19981000	0.40981700
C	3.60572900	1.50386700	0.08184200
C	3.95177700	-0.52034500	-1.60723500
C	4.28436200	0.78835000	-1.95226300
B	-0.67759900	-3.57917500	-0.08682500
H	-2.67571700	-2.51341200	1.23548300
H	-2.14631800	-0.89873600	1.62948300
H	-2.48738400	-0.15820700	-0.69295700
H	-0.12893500	-2.11312200	1.39238000
H	-0.60602800	0.28898500	1.52171000
H	0.10704900	-1.58572700	-1.42751200
H	0.36440600	2.54068500	2.05393500
H	1.08123100	2.55583400	0.43117100
H	-3.48052200	-3.01853800	-1.15775700
H	-3.72237700	-1.67991200	-2.28760000
H	-2.09314700	-2.26138400	-1.95892600
H	-4.32197800	-0.01725900	1.00330800
H	-4.95396000	-0.22220700	-0.63547900
H	-4.91606200	-1.60092200	0.47497400
H	1.05930300	-0.11721500	2.91697200
H	-0.27101400	3.16130500	-1.45579200
H	-2.17267000	2.10031600	2.23152500
H	-1.29177500	-5.42378800	0.29596100
H	0.04170700	-4.72535500	-1.54568600
H	-2.47021500	3.78258700	-2.41137600

H	-4.36992500	2.69771100	1.27824900
H	-4.52908600	3.53878500	-1.05028600
H	3.47315700	2.31337300	0.79234700
H	4.10935800	-1.33438900	-2.30552600
H	4.69522300	1.01260000	-2.93012800

Bortezomib in water

O	0.84259100	0.71668600	-1.26986000
O	-1.30643400	-4.53185600	0.62903700
O	0.08116800	-3.81485500	-1.21794400
O	3.39436700	-0.60202800	2.62509400
N	-0.02225800	-1.21165900	-0.49411800
N	1.35571000	0.11055900	1.97559000
N	3.44232000	-0.80520300	-0.42007800
N	4.11304600	1.82624100	-1.09976600
C	-2.14581500	-1.65733800	0.81896300
C	-2.96795700	-1.10733100	-0.35002700
C	-0.69819300	-2.08986100	0.46370100
C	0.31929400	0.64181100	1.08441900
C	0.40312800	0.05444900	-0.33849900
C	0.24152400	2.17237200	1.03822500
C	-3.04596500	-2.10220400	-1.50895000
C	-4.37085200	-0.74699000	0.13968200
C	-1.08833200	2.59658900	0.45648500
C	-1.18764900	3.09121300	-0.84424200
C	-2.25699700	2.44503500	1.20866500
C	2.66949400	-0.12555100	1.76474600
C	-2.42699800	3.42825900	-1.38276800
C	-3.49693300	2.77983100	0.67374700
C	-3.58532700	3.27052200	-0.62657200
C	3.23740700	0.21482500	0.40914900
C	3.58749200	1.52245200	0.08366300
C	3.95630400	-0.49667400	-1.60710700
C	4.27873100	0.81531500	-1.94943000
B	-0.64175000	-3.58205900	-0.08493000
H	-2.65578900	-2.53449900	1.23184200
H	-2.14538600	-0.91221300	1.62013300
H	-2.48722300	-0.18675400	-0.70721500

H	-0.11477900	-2.10727200	1.39346600
H	-0.61239900	0.28799000	1.52176300
H	0.11821800	-1.57881900	-1.42826900
H	0.34368000	2.54345800	2.06090500
H	1.06208900	2.56845100	0.43923900
H	-3.45144500	-3.05864900	-1.16049300
H	-3.70316600	-1.72781800	-2.29739200
H	-2.06939400	-2.29215200	-1.96170600
H	-4.32691800	-0.05410000	0.98350600
H	-4.95372900	-0.27532900	-0.65542100
H	-4.90416400	-1.64663400	0.46350400
H	1.05379700	-0.11106400	2.91735300
H	-0.28730500	3.19547800	-1.43894900
H	-2.19491000	2.06696800	2.22545800
H	-1.24098000	-5.43193800	0.29525800
H	0.10061700	-4.72292300	-1.53608000
H	-2.48776100	3.81081300	-2.39508800
H	-4.39309100	2.65802800	1.27112200
H	-4.55012800	3.52997000	-1.04633900
H	3.44584700	2.32994900	0.79465700
H	4.12290200	-1.30836000	-2.30602200
H	4.69077400	1.04423400	-2.92569800

Bortezomib (de1) in gas phase

O	0.99757200	-1.13554400	1.48254400
O	-0.22342300	3.99963600	-1.18457700
O	0.76838900	3.28123600	0.90568600
O	3.69219700	-2.81904900	-1.22084100
N	0.01009700	0.65598000	0.49229700
N	1.58151700	-2.05344800	-1.24996600
N	2.77812600	0.46783000	-0.49867200
N	4.99775700	0.28894300	1.19617300
C	-1.68417900	1.39638000	-1.23334100
C	-2.83288600	1.48432000	-0.22138000
C	-0.26490500	1.52704700	-0.66309900
C	0.34429100	-1.41253500	-0.78750800
C	0.50701200	-0.58804600	0.49860300

C	-0.62298000	-2.57637100	-0.46522300
C	-2.74260700	2.71382600	0.68338600
C	-4.16772100	1.48483900	-0.96989200
C	-1.97886000	-2.18614100	0.06843500
C	-2.15568200	-1.94357200	1.43379100
C	-3.08586700	-2.10040200	-0.77571000
C	2.90121200	-1.93107200	-0.93603600
C	-3.41302000	-1.63297600	1.93856600
C	-4.34628000	-1.79409300	-0.27148800
C	-4.51364000	-1.56159100	1.08876900
C	3.39857300	-0.68267100	-0.27031700
C	4.51582400	-0.77339900	0.55929600
C	3.24282600	1.53506400	0.15116800
C	4.34522800	1.43396800	0.99725800
B	0.14566100	3.03937600	-0.19827000
H	-1.79439300	2.20290200	-1.96843400
H	-1.81093500	0.44946300	-1.78007500
H	-2.78969000	0.59130100	0.41082000
H	0.44798300	1.27386500	-1.45787500
H	-0.06241600	-0.79365300	-1.58670200
H	0.25987000	1.20487400	1.31771000
H	-0.75448400	-3.17092900	-1.37596400
H	-0.10854300	-3.19556800	0.27431600
H	-2.68178000	3.62607800	0.08124700
H	-3.62818100	2.77903300	1.32428700
H	-1.85867900	2.68853600	1.32389900
H	-4.22202000	0.66068000	-1.68685900
H	-5.01134700	1.38746400	-0.27898400
H	-4.28672800	2.42298500	-1.52341500
H	1.42933800	-2.95036700	-1.69454800
H	-1.29275400	-1.98588300	2.09045200
H	-2.95925500	-2.28107300	-1.83914400
H	0.08138500	4.84852100	-0.85292300
H	-3.53171300	-1.43902200	2.99835100
H	-5.19481700	-1.72712600	-0.94287900
H	-5.49309900	-1.31578600	1.48284400
H	5.00823300	-1.72790000	0.70242900
H	2.69368700	2.46620300	0.04907200
H	4.70362800	2.30769800	1.53073100

Bortezomib (de1) in diethylether

O	0.83089200	-1.36955100	1.64063000
O	0.45397900	3.86922800	-0.99343000
O	0.97175500	3.07219800	1.23207100
O	3.58063700	-3.14643900	-0.59360800
N	0.09254600	0.51724600	0.62884200
N	1.49442900	-2.37608800	-0.96314400
N	2.77297800	0.20345200	-0.91317900
N	4.75565800	0.50842000	1.03328600
C	-1.47056100	1.49520900	-1.11198500
C	-2.65359900	1.68710700	-0.15715100
C	-0.06464600	1.45564400	-0.49466400
C	0.30619500	-1.56305800	-0.67557500
C	0.45131400	-0.77225200	0.63268100
C	-0.83775400	-2.58866300	-0.49535700
C	-2.45928400	2.85081800	0.81523800
C	-3.92988100	1.89714200	-0.97487400
C	-2.18161200	-2.05272300	-0.06857400
C	-2.46610600	-1.87125100	1.28787300
C	-3.18639000	-1.80326900	-1.00385300
C	2.79021000	-2.21205400	-0.59912200
C	-3.72961800	-1.45923600	1.69834000
C	-4.45308400	-1.39534200	-0.59545000
C	-4.72895000	-1.22488700	0.75723900
C	3.27486600	-0.83469200	-0.25195600
C	4.27097600	-0.68769500	0.71163300
C	3.25949600	1.39980300	-0.59497800
C	4.24336000	1.54962200	0.37976400
B	0.49848000	2.88853300	0.04242900
H	-1.46084300	2.32168100	-1.83370800
H	-1.66351200	0.57948900	-1.68803800
H	-2.76426300	0.76778000	0.42672700
H	0.61924600	1.15036800	-1.29313200
H	0.09544400	-0.89456400	-1.51034600
H	0.30379700	1.00688200	1.49888000
H	-0.94689400	-3.13035300	-1.43972600
H	-0.49018800	-3.29770500	0.26109300
H	-2.21455100	3.76965700	0.27069000
H	-3.37606700	3.02777900	1.38583900

H	-1.64866800	2.66280100	1.52115400
H	-4.04163500	1.12693800	-1.74234300
H	-4.81876400	1.86919200	-0.33759300
H	-3.90018200	2.87169900	-1.47488700
H	1.30056000	-3.34808300	-1.17281500
H	-1.68748500	-2.05781500	2.02042800
H	-2.97928300	-1.94584600	-2.06020000
H	0.80429900	4.68787300	-0.63009200
H	-3.93438100	-1.32419300	2.75405500
H	-5.22450600	-1.21135800	-1.33440900
H	-5.71544800	-0.90900200	1.07628300
H	4.66551200	-1.55599500	1.22625900
H	2.85167600	2.26017300	-1.11480600
H	4.61677200	2.53389200	0.63723000

Bortezomib (de1) in methanol

O	1.11212100	0.51954400	-3.11002600
O	-2.03075100	3.72717200	0.78234100
O	-0.68025200	4.19700100	-1.17032800
O	-3.48119700	-1.50511600	-2.12944400
N	0.14414600	1.60443900	-1.37652300
N	-1.23927400	-1.19462900	-2.10948300
N	-1.56355400	-2.35875000	0.57739700
N	-3.45419300	-0.91906600	2.05292800
C	-0.33432500	1.25811100	1.11083800
C	1.15546000	1.41940200	1.42745200
C	-0.80023000	1.78786000	-0.26497200
C	0.02223700	-0.84176600	-1.46485600
C	0.48243800	0.50110400	-2.05078700
C	1.09261700	-1.92306000	-1.63769200
C	1.60934400	2.87499200	1.31856600
C	1.43885500	0.87134900	2.82654900
C	2.26096100	-1.62020800	-0.73204800
C	3.33811900	-0.84643000	-1.16792700
C	2.22951800	-2.04183600	0.60004800
C	-2.42978200	-1.41013200	-1.51127800
C	4.36054000	-0.49783000	-0.28836800
C	3.25198400	-1.69992700	1.47875200
C	4.31913300	-0.92159900	1.03723400

C	-2.44047200	-1.54279200	-0.00662600
C	-3.39334700	-0.83649300	0.72602400
C	-1.64418600	-2.46172900	1.90142300
C	-2.57799600	-1.73355700	2.63720400
B	-1.14729100	3.38270100	-0.27406200
H	-0.91481100	1.79774400	1.86804200
H	-0.59185300	0.19969400	1.24724400
H	1.73110300	0.82362700	0.70732100
H	-1.73876800	1.27332200	-0.51730900
H	-0.16650800	-0.71779900	-0.40569300
H	0.36603000	2.49107000	-1.82928000
H	0.64058100	-2.87858800	-1.36484600
H	1.40181500	-1.96107400	-2.68382000
H	1.02044700	3.50764400	1.99228700
H	2.66218300	2.97274800	1.59814100
H	1.48640000	3.26242200	0.30510800
H	1.11234100	-0.16899300	2.91565200
H	2.50750000	0.90732500	3.05463100
H	0.90791700	1.46148100	3.58134800
H	-1.27315100	-1.14377500	-3.12207400
H	3.36116400	-0.50488700	-2.19693700
H	1.39004000	-2.63836900	0.94594900
H	-2.22987500	4.66598400	0.71830100
H	5.18931800	0.10656200	-0.63897800
H	3.21192700	-2.03208800	2.50984700
H	5.11344600	-0.64791200	1.72193300
H	-4.10377200	-0.18917800	0.22338100
H	-0.94818300	-3.13091900	2.39468900
H	-2.61327600	-1.81506100	3.71758500

Bortezomib (de1) in water

O	-1.11578800	0.52166600	3.10689800
O	2.04436000	3.72121500	-0.77694800
O	0.69015600	4.19524000	1.17185100
O	3.47587500	-1.51060500	2.13219800
N	-0.14138500	1.60368000	1.37584200
N	1.23438500	-1.19829800	2.10932600
N	1.55982100	-2.36146000	-0.57710200
N	3.45366200	-0.92426600	-2.05079200

C	0.33917000	1.25808900	-1.11108300
C	-1.15030600	1.42114500	-1.42823400
C	0.80483100	1.78573500	0.26572500
C	-0.02599800	-0.84314100	1.46374700
C	-0.48386200	0.50080600	2.04895600
C	-1.09873500	-1.92227600	1.63556500
C	-1.60304700	2.87688300	-1.31667600
C	-1.43318500	0.87602700	-2.82852700
C	-2.26627200	-1.61618500	0.72987900
C	-3.34441700	-0.84488100	1.16766000
C	-2.23306900	-2.03248700	-0.60389900
C	2.42532200	-1.41473600	1.51238000
C	-4.36595200	-0.49293700	0.28826600
C	-3.25456600	-1.68731300	-1.48244100
C	-4.32256000	-0.91107900	-1.03908400
C	2.43774800	-1.54711900	0.00782800
C	3.39239400	-0.84240300	-0.72374500
C	1.64121400	-2.46441100	-1.90100600
C	2.57661200	-1.73714200	-2.63599700
B	1.15671300	3.37916600	0.27636400
H	0.92019700	1.79797600	-1.86769400
H	0.59588100	0.19971400	-1.24852000
H	-1.72678100	0.82421500	-0.70968400
H	1.74161900	1.26853000	0.51897500
H	0.16381500	-0.71952100	0.40467200
H	-0.36341500	2.49026500	1.82829400
H	-0.64857500	-2.87847100	1.36197500
H	-1.40814700	-1.96086900	2.68160400
H	-1.01379300	3.51028100	-1.98941500
H	-2.65575000	2.97613400	-1.59614800
H	-1.47972200	3.26215800	-0.30239400
H	-1.10723300	-0.16429400	-2.91936400
H	-2.50162800	0.91324100	-3.05725900
H	-0.90137500	1.46731300	-3.58179800
H	1.26668800	-1.15086700	3.12215400
H	-3.36942700	-0.50838300	2.19829600
H	-1.39307000	-2.62731400	-0.95141200
H	2.24736700	4.65922700	-0.71299300
H	-5.19578000	0.10910800	0.64045800
H	-3.21305500	-2.01524700	-2.51480600

H	-5.11612700	-0.63496600	-1.72366700
H	4.10386400	-0.19675700	-0.22042900
H	0.94462200	-3.13229900	-2.39509500
H	2.61212000	-1.81817600	-3.71637600

Bortezomib (de2) in gas phase

O	0.66394500	1.47366100	-1.71115300
O	0.98522900	-3.72677400	0.93666700
O	1.03039400	-2.85366000	-1.32324900
O	3.51770300	3.22058900	0.29455300
N	0.05809700	-0.44434000	-0.66442000
N	1.44811300	2.50234100	0.84169100
N	2.74555100	-0.05161800	1.09492200
N	4.56564800	-0.64496700	-0.94609500
C	-1.40277900	-1.55579200	1.08118400
C	-2.55387200	-1.84849300	0.11468400
C	-0.00423300	-1.39048300	0.46990100
C	0.27486100	1.64743100	0.62888400
C	0.37230000	0.85787500	-0.68774100
C	-0.91489600	2.62503600	0.49728900
C	-2.25501700	-3.02092600	-0.82070600
C	-3.82837900	-2.13156400	0.91345000
C	-2.24658700	2.02405500	0.12238000
C	-2.56721700	1.81729800	-1.22225100
C	-3.19990500	1.71784100	1.09303200
C	2.73797400	2.29637100	0.44915300
C	-3.81797700	1.32994400	-1.58389500
C	-4.45257200	1.23045400	0.73302600
C	-4.76675500	1.03922200	-0.60784500
C	3.20016400	0.87982300	0.26310400
C	4.11561400	0.59025800	-0.74721600
C	3.19719800	-1.29072700	0.90334600
C	4.09394400	-1.57991000	-0.12273200
B	0.70586400	-2.81492400	0.07786000
H	-1.31157000	-2.38382000	1.79582900
H	-1.67902500	-0.66020900	1.65725000
H	-2.71757600	-0.95112300	-0.49283200

H	0.62814000	-0.99878500	1.26736400
H	0.13173400	0.98046100	1.47901600
H	0.20194200	-0.89663100	-1.56318500
H	-1.01066800	3.16725600	1.44338400
H	-0.63031400	3.34102100	-0.27961100
H	-1.94862000	-3.89943500	-0.24243400
H	-3.14406100	-3.28217100	-1.40430900
H	-1.44372600	-2.79484900	-1.51416800
H	-4.00853300	-1.35585300	1.66238800
H	-4.70548300	-2.18253100	0.26009200
H	-3.73690000	-3.09041500	1.43544700
H	1.25183900	3.49465300	0.87276500
H	-1.81939100	2.03275900	-1.97893900
H	-2.95941900	1.86871100	2.14112100
H	1.45695900	-3.69998900	-1.47943900
H	-4.04908100	1.17038100	-2.63083300
H	-5.18101500	0.99484400	1.50049900
H	-5.74053300	0.65519200	-0.88933200
H	4.46869000	1.37907700	-1.40115900
H	2.80087200	-2.08060100	1.53476800
H	4.42519700	-2.60003500	-0.28250100

Bortezomib (de2) in diethylether

O	0.98415600	0.39648600	-1.30717400
O	-1.99730000	-4.49292800	0.53863800
O	-0.56913300	-3.84836000	-1.30447100
O	3.45032000	-0.67047300	2.68589600
N	-0.18852600	-1.34945100	-0.47995000
N	1.40820400	-0.07905900	1.93358400
N	3.50324200	-1.01122900	-0.38627500
N	4.33389500	1.57126500	-1.08263900
C	-2.30709800	-1.45225200	0.91836900
C	-3.05801400	-0.70114100	-0.18658700
C	-0.97398300	-2.11842300	0.49914100
C	0.40215200	0.48935900	1.02851600
C	0.42238300	-0.16588400	-0.36885900
C	0.45885200	2.01913700	0.91356600
C	-3.30408000	-1.58000400	-1.41412400
C	-4.38728700	-0.18084900	0.36360100

C	-0.84648800	2.53681900	0.35600000
C	-0.99835900	2.80090100	-1.00577300
C	-1.95242900	2.68671700	1.19689400
C	2.73937700	-0.26644800	1.77955100
C	-2.23003800	3.20281400	-1.51583400
C	-3.18323600	3.09026000	0.69074000
C	-3.32620700	3.34568700	-0.67029100
C	3.34373700	0.02698300	0.42792900
C	3.77750300	1.30794400	0.09574300
C	4.05096600	-0.74424200	-1.56740800
C	4.45050600	0.54270000	-1.91955100
B	-1.25490200	-3.62846900	-0.06646400
H	-2.94067200	-2.26633900	1.28717500
H	-2.18053300	-0.76385900	1.76393500
H	-2.45796600	0.16668500	-0.49385400
H	-0.36193900	-2.25169400	1.40013200
H	-0.54729300	0.23266900	1.49301300
H	-0.06980500	-1.80318900	-1.37938300
H	0.63139800	2.42910100	1.91205800
H	1.28819900	2.30530500	0.26608700
H	-3.83072800	-2.49504800	-1.12353400
H	-3.91514800	-1.05032800	-2.15071400
H	-2.37228800	-1.87737000	-1.89896600
H	-4.23456800	0.41945000	1.26443600
H	-4.90072900	0.44433800	-0.37234000
H	-5.04482200	-1.01854700	0.61934500
H	1.08381900	-0.31474300	2.86344500
H	-0.14964000	2.66107700	-1.66489600
H	-1.84703900	2.48225500	2.25879200
H	-0.77195600	-4.74051500	-1.59872500
H	-2.33420600	3.39753800	-2.57701800
H	-4.03202000	3.19825500	1.35633800
H	-4.28628400	3.65286900	-1.06849900
H	3.67399100	2.12912900	0.79809300
H	4.17820600	-1.57282000	-2.25504600
H	4.88493900	0.73918700	-2.89344700

Bortezomib (de2) in methanol

O	0.73121300	0.78842800	-1.33982700

O	-0.58484400	-4.84411100	0.28782500
O	0.44340800	-3.69401200	-1.57011800
O	3.18579500	-0.56650800	2.62832000
N	0.17256600	-1.25602800	-0.56650500
N	1.16853800	0.18845400	1.95344200
N	3.54388900	-0.45657600	-0.35274300
N	3.88705600	2.28047300	-0.83365600
C	-1.75442700	-2.02076700	0.90477100
C	-2.72559700	-1.38984900	-0.09836900
C	-0.31741500	-2.27103300	0.37994100
C	0.14997300	0.63579400	0.99637700
C	0.37778600	0.05821000	-0.41577700
C	-0.05895300	2.15484800	0.95424900
C	-2.86963100	-2.24612900	-1.35712300
C	-4.08741000	-1.17148100	0.56169100
C	-1.41683100	2.46912200	0.36855600
C	-1.55608000	2.92315900	-0.94320500
C	-2.56902600	2.24908900	1.12876700
C	2.49191200	-0.02248500	1.78225100
C	-2.81892600	3.15319100	-1.48395600
C	-3.83218600	2.47401300	0.59136500
C	-3.96037000	2.92631900	-0.71968400
C	3.11721600	0.47067500	0.50076700
C	3.30256600	1.83201700	0.27409600
C	4.10957000	-0.00565300	-1.46801100
C	4.27094200	1.35749200	-1.71188200
B	-0.18610300	-3.75553200	-0.28795500
H	-2.14638100	-2.99871400	1.20466800
H	-1.75327500	-1.41327500	1.81756700
H	-2.33981600	-0.40467000	-0.39516600
H	0.35444100	-2.28255300	1.24737400
H	-0.77616300	0.19834800	1.36543200
H	0.39862700	-1.61052800	-1.48929700
H	0.00426000	2.52976200	1.97883600
H	0.72590400	2.62326500	0.36036700
H	-3.23558400	-3.24419200	-1.09306300
H	-3.58327600	-1.79666400	-2.05288700
H	-1.91716900	-2.36680700	-1.87827900
H	-3.99759200	-0.54674100	1.45463200
H	-4.78142200	-0.67831400	-0.12432700

H	-4.52620000	-2.12986500	0.85850400
H	0.82266200	-0.13937100	2.84766500
H	-0.66737400	3.07650300	-1.54450400
H	-2.47529700	1.89561400	2.15209100
H	0.49668300	-4.58417600	-1.92921200
H	-2.91104700	3.50488700	-2.50512000
H	-4.71498600	2.29395100	1.19400900
H	-4.94296200	3.10117500	-1.14201400
H	2.98371300	2.56534600	1.00767100
H	4.45220800	-0.74085600	-2.18722600
H	4.73298700	1.70169800	-2.63025800

Bortezomib (de2) in water

O	0.68687400	0.83747300	-1.33671300
O	-0.38931400	-4.85418700	0.25743000
O	0.60562300	-3.65076800	-1.58452200
O	3.16168700	-0.52406000	2.61980900
N	0.22310100	-1.23248000	-0.56999300
N	1.13919700	0.23009800	1.95903200
N	3.54886400	-0.36730000	-0.34396900
N	3.80759600	2.38030900	-0.81596000
C	-1.66677400	-2.08465700	0.90089300
C	-2.66554900	-1.48769000	-0.09602000
C	-0.22259800	-2.27321800	0.37044300
C	0.11270800	0.65212600	0.99875900
C	0.36688700	0.08959800	-0.41461100
C	-0.14375100	2.16386900	0.96494600
C	-2.78046800	-2.34294400	-1.35842400
C	-4.03245400	-1.32443800	0.56938300
C	-1.50736900	2.43953700	0.37279600
C	-1.65188100	2.91859100	-0.92941300
C	-2.65736100	2.15941300	1.11648100
C	2.46324300	0.02756500	1.78224600
C	-2.91747300	3.11389000	-1.47734900
C	-3.92321800	2.34944800	0.57186600
C	-4.05650200	2.82684300	-0.72982100
C	3.08401000	0.54356300	0.50760700
C	3.22707200	1.91068400	0.28526900
C	4.10984900	0.10411300	-1.45310500

C	4.22936500	1.47254500	-1.69252100
B	-0.02992000	-3.74604100	-0.30774000
H	-2.01842300	-3.07915300	1.19640100
H	-1.68623800	-1.48304700	1.81722600
H	-2.31946300	-0.48704000	-0.38980900
H	0.45099200	-2.26296300	1.23670500
H	-0.80204000	0.18373700	1.35864600
H	0.46488500	-1.57181700	-1.49445300
H	-0.09864400	2.53332900	1.99247300
H	0.62939500	2.66153900	0.37951500
H	-3.11015300	-3.35459400	-1.09862800
H	-3.51081800	-1.91655500	-2.05131200
H	-1.82496900	-2.42662000	-1.88142500
H	-3.96387100	-0.69925600	1.46390400
H	-4.74750600	-0.85684300	-0.11289900
H	-4.43270800	-2.29992600	0.86487000
H	0.79767200	-0.10791400	2.85123100
H	-0.76441600	3.11997400	-1.51827400
H	-2.55983500	1.78615100	2.13240600
H	0.69730300	-4.53502600	-1.95059000
H	-3.01352400	3.48584000	-2.49095100
H	-4.80397400	2.12292800	1.16167900
H	-5.04109000	2.97497300	-1.15765500
H	2.87827700	2.63149800	1.01751900
H	4.48318300	-0.61770700	-2.17055300
H	4.68885200	1.83361800	-2.60566900

Threonine (Thr1-OH) in gas phase

O	1.04520500	-1.67870100	-0.19195700
O	-1.81922900	-1.07168800	-0.30731100
O	-1.46831600	0.55591900	1.18836500
N	0.18889600	1.87703600	-0.75703100
C	1.26508700	-0.28598200	-0.33274800
C	-0.03446300	0.44377000	-0.75323900
C	1.81924100	0.20173700	0.99229300
C	-1.17177100	0.00985600	0.16168400
H	-0.28484800	0.12067200	-1.76775800

H	1.99339800	-0.08534600	-1.12728800
H	2.11901700	1.24786500	0.92387700
H	2.69730800	-0.39337700	1.24500000
H	1.08093300	0.08486300	1.78850400
H	0.16552600	2.22538700	0.19731200
H	-0.55196800	2.35238900	-1.25956300
H	0.60426600	-2.02018000	-0.97539000
H	-2.47575400	-1.33206400	0.35380600

Threonine (Thr1-OH) in diethylether

O	1.13236800	-1.64293400	-0.21405800
O	-1.78914700	-1.12724700	-0.29439700
O	-1.48588100	0.52438500	1.18261400
N	0.11847700	1.87276800	-0.78778400
C	1.28017400	-0.23535300	-0.31603000
C	-0.04618200	0.43040600	-0.75574200
C	1.78017700	0.25100200	1.03060500
C	-1.17378300	-0.02801700	0.15971600
H	-0.27506000	0.07791300	-1.76481200
H	2.01269600	0.01951300	-1.08994500
H	1.98788200	1.32118100	1.00404400
H	2.70649500	-0.27052400	1.27422400
H	1.04863100	0.04543400	1.81480900
H	0.08502600	2.24139700	0.15870000
H	-0.64837500	2.30363800	-1.29286700
H	0.80723100	-1.99271200	-1.04961400
H	-2.45489400	-1.39707800	0.35538500

Threonine (Thr1-OH) in methanol

O	1.18176700	-1.61989300	-0.22390500
O	-1.76544000	-1.16395900	-0.28717500
O	-1.50184800	0.50744700	1.17356900
N	0.07927400	1.87050000	-0.79990900
C	1.28789300	-0.20644600	-0.30624300
C	-0.05179300	0.42403800	-0.75720300

C	1.75655100	0.27879500	1.05212300
C	-1.17478400	-0.04981200	0.15609000
H	-0.26678600	0.05898600	-1.76428000
H	2.02163100	0.07776900	-1.06833400
H	1.90720000	1.35886800	1.04921600
H	2.70844700	-0.19642700	1.29229200
H	1.03272700	0.02094700	1.82814700
H	0.02321900	2.24802000	0.14203300
H	-0.69219000	2.27652200	-1.31886000
H	0.92740900	-1.97129800	-1.08343100
H	-2.43960400	-1.43509600	0.35406200

Threonine (Thr1-OH) in water

O	1.18647500	-1.61759100	-0.22481700
O	-1.76297800	-1.16758800	-0.28645800
O	-1.50352200	0.50581900	1.17257800
N	0.07550000	1.87027900	-0.80091000
C	1.28860700	-0.20364700	-0.30529200
C	-0.05229500	0.42345500	-0.75732100
C	1.75432000	0.28142200	1.05415700
C	-1.17487100	-0.05192100	0.15568500
H	-0.26586700	0.05729300	-1.76422900
H	2.02238100	0.08329300	-1.06629200
H	1.89955100	1.36225100	1.05348100
H	2.70850800	-0.18934200	1.29405300
H	1.03137200	0.01868400	1.82938100
H	0.01659500	2.24856700	0.14055800
H	-0.69604400	2.27386100	-1.32169300
H	0.93887500	-1.96887500	-1.08641500
H	-2.43823400	-1.43866300	0.35372700

Deprotonated threonine (Thr1-O^-) in gas phase

O	1.90724800	-0.93286600	-0.71408000
O	-1.11592500	-1.57750200	-0.46785200

O	-1.98001700	0.06470100	0.76180700
N	-0.34246400	1.94574300	-0.35036400
C	1.40149900	0.10760900	-0.10408600
C	-0.08213900	0.54157700	-0.65490200
C	1.30828300	-0.01261800	1.43745500
C	-1.14776800	-0.29387600	-0.04627100
H	-0.01809600	0.29654500	-1.72094700
H	1.93340600	1.08809300	-0.29699700
H	0.78540200	0.83515700	1.89385600
H	2.32302900	-0.08136900	1.83912400
H	0.78963300	-0.94528400	1.68795100
H	-1.29956900	2.20862800	-0.55878000
H	0.28481200	2.52522600	-0.89430700
H	-1.77106000	-2.03801600	0.07048600

Deprotonated threonine (Thr1-O^-) in diethylether

O	1.67573300	-1.28145500	-0.61130200
O	-1.44522600	-1.41066600	-0.44737000
O	-1.77224200	0.26026200	0.98667200
N	-0.13452200	1.92574300	-0.52413000
C	1.36924600	-0.05879400	-0.17311000
C	-0.04826200	0.47865400	-0.70168900
C	1.42276200	0.08774000	1.36115800
C	-1.16558200	-0.18667300	0.03941500
H	-0.09392400	0.14572100	-1.74483400
H	2.03759100	0.74912100	-0.57831000
H	1.05890300	1.06090800	1.70260100
H	2.45525600	-0.05091500	1.69242700
H	0.81633700	-0.69978900	1.82104700
H	-0.99671600	2.29341500	-0.91322500
H	0.63068000	2.35809400	-1.03091400
H	-2.10157600	-1.80744900	0.14148300

Deprotonated threonine (Thr1-O^-) in methanol

O	1.86416700	-1.03739200	-0.69294200
O	-1.23480700	-1.52250600	-0.48202700
O	-1.90970200	0.14029300	0.83444700
N	-0.25840900	1.94401900	-0.39972300

C	1.37592300	0.07275400	-0.10845800
C	-0.04951100	0.52329900	-0.66517500
C	1.30997800	-0.01605500	1.42789800
C	-1.15489600	-0.26218700	-0.02142700
H	-0.03714000	0.26428300	-1.73038300
H	1.97712100	0.98842400	-0.33506300
H	0.85140200	0.86981400	1.87670100
H	2.32144800	-0.13113600	1.82512800
H	0.73374900	-0.90094700	1.72109700
H	-1.17397200	2.24410500	-0.71966300
H	0.42667300	2.47638800	-0.92571300
H	-1.93664100	-1.96909300	0.01310100

Deprotonated threonine (Thr1-O^-) in water

O	1.85837300	-1.04512700	-0.69244400
O	-1.24173600	-1.51975900	-0.48032200
O	-1.90618500	0.14424700	0.83999800
N	-0.25550900	1.94367000	-0.40397500
C	1.37529100	0.07017400	-0.11069400
C	-0.04932200	0.52181200	-0.66546000
C	1.31609800	-0.01288700	1.42604200
C	-1.15574300	-0.26037700	-0.01855000
H	-0.03964700	0.26060000	-1.73007300
H	1.97954100	0.98173100	-0.34373100
H	0.86631800	0.87751200	1.87459000
H	2.32882800	-0.13270000	1.81859900
H	0.73553000	-0.89264300	1.72625100
H	-1.16979500	2.24423300	-0.72726900
H	0.43090300	2.47291300	-0.93151300
H	-1.94468200	-1.96455800	0.01509400

3. Frequency and IR Intensity

Bortezomib calculations at level of theory - B3LYP/6-31+G(d,p)

Bortezomib in gas phase

Frequency (cm^{-1})	IR Intensity (KM/Mole)
12.8369	0.6999
18.0386	0.3546
19.7861	0.1033
21.9936	0.1434
32.0185	0.8818
38.3626	0.0583
47.4024	0.2594
53.0935	0.1444
62.2918	1.4188
64.7467	0.0323
88.9305	0.1313
97.2829	2.9074
106.4663	0.3895
129.5518	3.3320
158.4042	1.0126
165.3409	0.1013

Frequency (cm^{-1})	IR Intensity (KM/Mole)
174.3714	1.4892
198.3763	0.2531
225.4119	1.1338
232.1367	1.5395
241.7717	0.4341
262.8067	0.2753
279.2161	2.1901
310.2629	3.2051
325.6366	2.3389
342.7400	6.3936
365.6801	4.6149
378.9656	4.8723
386.1768	3.5135
392.1981	5.1389
417.5466	0.2739
419.2449	0.6586
426.0958	0.5013
431.3485	13.3104
449.1827	14.6263

Frequency (cm⁻¹)	IR Intensity (KM/Mole)
461.7147	29.9496
478.2426	20.2448
495.9376	24.3248
528.6515	1.8052
550.4801	13.1594
571.9023	202.4924
583.7833	30.8041
592.2986	66.5415
616.0688	35.5057
629.3531	44.4607
634.6774	3.3593
639.2770	21.3596
666.4159	33.1369
701.3723	51.4622
714.0800	39.9607
736.9305	5.9545
742.5145	32.1412
768.3539	39.3378
770.4994	22.9378

Frequency (cm⁻¹)	IR Intensity (KM/Mole)
786.1244	19.2095
799.4223	1.5238
807.7228	8.2819
837.1946	6.7308
863.9366	0.5425
874.2874	1.7199
885.5761	20.7199
890.6953	10.3882
914.9050	161.6191
930.7567	6.7239
931.9424	5.6514
944.7532	5.5165
951.1211	9.2228
966.0467	0.3160
977.6758	1.3971
987.7029	1.3449
991.2590	4.3443
998.2462	0.1174
1002.7636	104.6923

Frequency (cm⁻¹)	IR Intensity (KM/Mole)
1005.9510	8.4569
1015.1960	0.0511
1024.9518	10.1448
1038.7254	34.0128
1052.8958	7.3212
1069.2142	13.2476
1094.6419	11.3018
1111.6029	18.2071
1115.3377	12.3940
1131.0008	5.4965
1164.7389	10.7761
1182.5119	1.9567
1186.0404	64.6983
1189.9627	16.0340
1197.9169	3.1214
1206.1030	2.2397
1215.0955	9.5620
1222.7041	0.4817
1228.0420	18.9200

Frequency (cm^{-1})	IR Intensity (KM/Mole)
1258.3564	8.6196
1267.3263	25.7535
1291.5028	46.3529
1292.8967	20.7689
1313.8656	6.3261
1325.3186	119.3676
1338.7425	12.6468
1354.4832	25.3871
1357.4764	58.1794
1361.0038	73.5495
1365.2614	24.4523
1379.2276	115.5064
1386.6544	27.1100
1402.4518	76.5287
1408.4275	133.8804
1411.3622	164.5242
1415.0299	72.4037
1429.0198	12.5474
1456.3119	91.2606

Frequency (cm⁻¹)	IR Intensity (KM/Mole)
1482.5007	1.7691
1484.4154	57.1965
1487.5051	0.5793
1489.1816	18.2757
1492.8407	11.3991
1497.2163	4.3236
1502.3496	5.2151
1505.6557	22.0748
1510.4498	6.1828
1520.5143	10.2115
1530.7829	17.1360
1594.9827	1.1425
1614.8482	9.9334
1628.5864	2.2124
1651.0018	10.6093
1717.1989	519.2667
1723.6603	258.5543
3019.9121	32.6507
3024.8734	52.2215

Frequency (cm^{-1})	IR Intensity (KM/Mole)
3025.8997	1.6148
3031.9410	22.8951
3050.3510	18.0472
3060.9631	28.1099
3064.6456	15.7348
3069.4205	24.4245
3090.2717	13.9549
3096.7949	50.6775
3101.6705	40.3331
3103.7824	48.0370
3139.1991	2.3732
3163.2863	9.7726
3176.7416	0.9563
3178.0416	1.5334
3187.1442	26.8059
3194.5778	25.9128
3199.9930	23.5437
3213.1039	6.5987
3218.5630	0.2162

Frequency (cm^{-1})	IR Intensity (KM/Mole)
3587.7884	26.6616
3593.2711	46.6596
3859.3434	14.4301
3863.8780	84.9376

Bortezomib in diethylether

Frequency (cm^{-1})	IR Intensity (KM/Mole)
14.0936	2.3288
17.5655	1.2272
21.7220	0.3426
23.7879	0.2966
29.2544	0.3621
40.9811	0.4789
45.1145	1.4877
49.0747	0.2917
53.6519	0.7700
70.8701	0.8161
78.3120	0.0332
94.3212	4.2145

Frequency (cm⁻¹)	IR Intensity (KM/Mole)
106.3582	0.7107
114.7353	4.8759
155.8384	0.5020
159.3814	4.2645
187.2032	0.2674
194.1534	0.4419
203.2083	0.6413
226.7260	0.2210
242.8496	0.8339
254.8648	1.1189
260.3656	2.3950
290.1153	5.7119
327.8507	0.3460
338.8979	14.8141
347.7656	7.2014
355.6140	1.7081
376.5836	15.6308
390.5043	2.9655
414.5711	30.1830

Frequency (cm⁻¹)	IR Intensity (KM/Mole)
416.9361	0.1857
424.7306	1.3221
447.1334	52.5978
459.1468	9.2025
472.5436	34.5270
491.3074	21.0539
510.4284	6.3481
538.0139	40.7535
567.2635	239.7528
570.7145	59.1828
596.2822	63.4325
599.9873	22.5819
612.5281	48.0301
631.8196	16.9505
633.0687	1.1675
639.8220	56.5203
669.2127	108.5578
692.2348	22.9744
711.2998	61.3788

Frequency (cm^{-1})	IR Intensity (KM/Mole)
741.0368	5.6785
763.4463	13.0635
767.3621	68.9262
771.5167	16.7838
782.8886	3.0295
797.5607	4.7120
815.2859	11.1378
838.3964	11.2827
861.2702	0.2485
865.1547	10.2149
881.9932	25.0632
889.6715	16.5662
928.6136	24.3089
933.8288	205.0943
935.3450	14.7242
944.7138	14.1850
954.6112	7.6560
964.3197	2.5383
965.6829	0.1790

Frequency (cm^{-1})	IR Intensity (KM/Mole)
988.1710	0.6159
994.9324	0.3117
996.1497	9.5250
1005.0600	1.3053
1006.0857	110.5360
1013.7883	0.0397
1032.7186	34.9290
1050.3580	8.5862
1051.5678	17.3342
1070.1208	30.0504
1088.1980	4.7768
1109.7554	42.3935
1113.0387	5.8581
1121.4573	4.3140
1162.7979	14.4242
1179.4709	0.2521
1187.1951	1.4738
1190.5609	86.4642
1199.8183	1.6590

Frequency (cm⁻¹)	IR Intensity (KM/Mole)
1201.3701	0.9134
1209.0379	48.9972
1222.4604	2.6663
1225.3032	14.3857
1236.6550	10.5486
1267.6045	38.4602
1294.4296	62.0768
1311.3361	112.1148
1320.0080	29.0981
1324.0041	12.5193
1336.6869	11.5194
1353.3196	20.2909
1357.3458	28.9201
1360.7110	79.3144
1367.8791	107.7229
1380.7650	32.1625
1384.6370	262.8571
1399.2467	103.3369
1405.2182	44.4406

Frequency (cm⁻¹)	IR Intensity (KM/Mole)
1414.9143	193.6117
1422.2694	104.5479
1424.6098	30.4879
1446.0035	85.6401
1479.9698	11.2808
1482.2211	4.9773
1486.0628	4.3609
1487.7898	15.1131
1492.0274	6.2261
1495.7686	5.0212
1497.2367	51.9597
1502.9960	13.3209
1512.7056	68.5027
1513.0916	13.8929
1529.4796	17.2364
1586.5638	4.2043
1621.3046	7.4082
1627.4570	1.6215
1648.8316	11.6216

Frequency (cm^{-1})	IR Intensity (KM/Mole)
1697.9316	748.7321
1710.5173	628.9802
3021.1344	20.7199
3022.1919	9.1096
3024.5740	77.7395
3032.1101	38.0943
3054.3002	22.5030
3062.8467	29.2908
3064.5701	45.9651
3088.8250	23.6406
3095.7489	79.1702
3101.2393	59.9919
3104.6851	8.0721
3106.3473	50.6094
3127.2919	12.8968
3167.3817	14.3453
3180.5069	0.7175
3186.4391	1.0520
3189.9000	19.8369

Frequency (cm^{-1})	IR Intensity (KM/Mole)
3190.0331	31.5773
3201.6281	31.4020
3202.1608	29.8381
3209.9203	8.7111
3575.3977	43.9843
3589.7608	60.9512
3839.5720	18.1575
3846.1715	153.1639

Bortezomib in methanol

Frequency (cm^{-1})	IR Intensity (KM/Mole)
17.3239	0.9431
22.4186	0.6336
23.2412	1.5750
29.9003	0.6171
34.0603	3.5957
40.3152	0.0222
44.5755	0.4506
48.8472	0.1633

Frequency (cm^{-1})	IR Intensity (KM/Mole)
56.6032	0.9145
74.4768	1.2956
76.8490	0.0472
93.1849	3.3429
107.2120	1.1755
112.2354	4.6086
156.4374	0.5228
159.9154	4.9447
189.3867	0.3046
195.5202	0.3430
202.7354	0.8485
227.1932	0.1745
243.6685	0.9168
253.5453	0.6778
262.1921	3.3534
288.0724	6.1768
327.7519	1.1552
341.7078	17.6456
346.9213	8.8304

Frequency (cm^{-1})	IR Intensity (KM/Mole)
356.7594	0.7012
373.4445	19.0035
390.0842	2.5197
413.3935	34.8679
416.1678	0.0873
424.6191	1.5142
451.5626	54.6635
460.5264	9.2803
482.4169	67.9543
502.7123	2.8317
518.6990	3.8240
539.8984	38.7590
566.0902	294.4066
572.6001	61.6431
598.3821	85.3755
603.4463	5.0665
609.0312	101.7284
628.5961	37.0222
631.6408	11.0872

Frequency (cm^{-1})	IR Intensity (KM/Mole)
632.4888	0.5606
667.8908	146.0383
692.9697	26.5241
710.0978	72.0211
740.4829	2.1587
761.8656	0.1019
762.7223	81.7628
771.9985	20.1719
782.9703	6.0189
796.8187	5.3928
816.0412	9.4793
838.7881	11.6284
859.7938	0.0208
866.5922	8.7320
882.4311	33.5289
889.2155	17.6528
927.8658	10.8263
935.3637	13.3420
940.7119	207.3485

Frequency (cm^{-1})	IR Intensity (KM/Mole)
944.9090	72.9010
954.7234	11.4192
965.0211	0.2947
967.6420	2.8893
988.4194	0.2542
997.2482	0.3934
998.3387	0.3851
1006.0766	0.0805
1012.9956	0.1996
1029.5224	159.8143
1032.8401	36.5237
1049.5059	11.8030
1052.9265	18.9745
1070.5097	33.3511
1088.7379	5.3486
1110.8450	52.1218
1113.1000	9.4568
1118.7594	8.3978
1163.6921	13.0958

Frequency (cm⁻¹)	IR Intensity (KM/Mole)
1177.8293	0.0665
1186.6307	3.3204
1192.0352	73.5621
1199.0777	2.9462
1200.9472	4.7246
1211.0973	53.9029
1222.8673	3.7402
1226.1744	9.1784
1233.6907	19.3485
1268.0820	31.9710
1297.2835	74.9941
1314.6577	128.6923
1321.6314	25.9679
1324.6556	9.0780
1337.1339	12.0089
1351.7490	21.6769
1357.6599	30.8822
1360.3266	89.8453
1370.8514	216.2370

Frequency (cm^{-1})	IR Intensity (KM/Mole)
1379.6284	107.2021
1382.4529	234.4743
1398.8029	62.4241
1403.4196	58.5690
1416.3665	201.2366
1422.7289	34.8605
1424.5999	106.7651
1445.4272	106.4623
1478.5140	11.0995
1480.0087	8.7440
1484.8851	11.8615
1486.2962	5.3549
1490.5740	15.4489
1492.6932	3.8456
1496.2370	52.8904
1500.0373	27.9133
1509.0531	17.0002
1516.0507	83.9353
1529.2604	17.5089

Frequency (cm^{-1})	IR Intensity (KM/Mole)
1585.8397	4.4133
1620.6934	14.4077
1626.7981	1.0856
1647.7424	15.7952
1682.2520	933.2092
1697.9812	749.8493
3018.8680	13.9853
3021.9928	19.7288
3025.1061	106.7943
3032.2349	40.7345
3057.9507	33.1063
3065.8840	34.5355
3066.4614	46.8807
3088.2823	37.3072
3095.1009	92.7181
3099.8597	72.7160
3105.6640	61.4464
3110.4708	5.4951
3133.8509	18.7105

Frequency (cm^{-1})	IR Intensity (KM/Mole)
3170.2328	14.9503
3181.4167	0.1950
3190.6966	32.2053
3191.1443	0.8044
3194.2598	21.2169
3200.7682	39.6131
3205.9898	31.1335
3207.5856	15.4857
3578.3263	64.5441
3586.8335	66.4804
3835.3668	80.1810
3838.3360	120.8117

Bortezomib in water

Frequency (cm^{-1})	IR Intensity (KM/Mole)
17.4394	0.9453
21.5974	0.7393
23.4189	1.2162
28.1305	0.7941

Frequency (cm^{-1})	IR Intensity (KM/Mole)
34.3217	3.8120
39.8207	0.0378
44.5096	0.4602
48.7382	0.1540
57.1767	0.9020
74.2423	1.2909
76.2798	0.0988
93.0468	3.3319
106.5265	1.2074
111.5414	4.6160
156.1745	0.5407
159.8943	4.9821
188.9713	0.3175
195.5010	0.3700
202.4139	0.8849
227.0119	0.1771
243.7412	0.9750
253.0603	0.6162
261.8551	3.5695

Frequency (cm^{-1})	IR Intensity (KM/Mole)
288.0864	6.2709
327.6663	1.2032
341.8479	17.5928
346.8971	9.0771
356.6852	0.7266
373.0643	19.6567
389.9768	2.4193
413.2305	35.3550
415.7749	0.0743
424.4587	1.6303
451.4401	56.0292
460.4284	9.3834
482.3253	71.1565
500.3116	2.8590
519.0089	3.7581
539.6493	38.4499
565.6783	293.0072
572.5532	64.5604
597.6564	89.7834

Frequency (cm^{-1})	IR Intensity (KM/Mole)
603.4492	4.4433
608.9856	106.5790
628.1725	37.5470
631.5443	11.8034
632.4377	0.6212
667.2158	148.2121
692.9957	26.3969
709.7366	73.6797
740.4415	1.9373
761.5586	0.5883
762.3180	81.7046
771.8291	20.7958
783.0615	6.4647
796.7079	5.4845
816.0720	9.5916
838.8470	11.7436
859.4852	0.0092
866.6726	8.4465
882.4799	34.5971

Frequency (cm^{-1})	IR Intensity (KM/Mole)
889.1383	17.8847
927.7891	10.7444
935.1024	12.2608
941.0368	199.6663
944.9147	86.5624
954.6429	11.8951
964.8918	0.2449
967.9798	2.9814
988.3242	0.2026
997.3578	0.3756
998.6075	0.4011
1006.1725	0.0730
1012.9146	0.1926
1031.1723	164.9756
1032.8468	35.0561
1049.4346	11.7409
1053.1289	20.0185
1070.5903	33.9450
1088.8993	5.6069

Frequency (cm⁻¹)	IR Intensity (KM/Mole)
1111.0484	53.1489
1113.0628	10.0909
1118.3430	8.7905
1163.7288	13.3130
1177.7307	0.0625
1186.5283	3.4525
1192.0292	73.5771
1199.1084	3.0908
1201.0252	4.9827
1211.2563	54.2867
1222.8870	3.7981
1226.3249	8.7828
1233.3944	20.3397
1268.0413	31.5782
1297.3299	75.9247
1314.9230	130.1486
1321.9628	24.5574
1325.3422	9.8904
1336.9827	12.0477

Frequency (cm^{-1})	IR Intensity (KM/Mole)
1351.7099	21.0869
1357.6857	35.7530
1360.3762	89.5039
1371.3357	259.4054
1379.3972	104.4040
1382.3423	211.1794
1398.7903	60.0619
1403.2294	61.4387
1416.6165	198.5037
1422.5113	37.9614
1425.0097	104.2734
1445.5918	108.6935
1478.0825	11.0091
1479.4771	9.7447
1484.3602	10.9833
1486.2162	5.4779
1490.4084	16.0731
1492.3047	3.6889
1496.1569	53.8787

Frequency (cm^{-1})	IR Intensity (KM/Mole)
1499.6891	29.8955
1508.5749	17.2739
1516.3594	85.2217
1529.3169	17.3982
1585.6365	4.4848
1620.7019	15.1530
1626.6766	1.0207
1647.6333	16.4890
1680.5987	954.3823
1696.3652	765.9211
3018.2566	15.9730
3022.0902	20.9515
3025.2441	108.1575
3032.2594	40.8562
3058.9453	34.5347
3066.1119	34.0977
3066.7963	47.8233
3088.2566	39.1411
3095.0444	93.9511

Frequency (cm⁻¹)	IR Intensity (KM/Mole)
3099.6922	74.4213
3105.3955	62.7462
3110.1514	5.5780
3134.0536	19.3393
3170.9008	14.7581
3181.5011	0.1624
3190.7773	32.0739
3191.6722	0.7687
3194.8878	21.5201
3200.5443	41.2331
3206.4543	30.9913
3207.4072	16.3080
3577.9813	66.8947
3586.7555	66.3811
3834.2051	77.3431
3837.5993	126.2999

Bortezomib (de1) in gas phase

Frequency (cm^{-1})	IR Intensity (KM/Mole)
18.4439	0.8097
24.5080	0.6243
33.1594	0.1835
37.6261	0.0883
47.9472	0.1467
50.6027	2.3097
59.3186	0.7976
61.5468	0.7818
71.7760	0.1813
86.2705	2.9421
100.2991	1.1037
106.5744	0.7509
116.2596	0.3284
132.3230	17.6588
146.6829	3.4276
171.2436	10.2349
182.0039	1.5031
201.2411	3.8907

Frequency (cm^{-1})	IR Intensity (KM/Mole)
209.8386	0.1404
229.6110	2.0641
241.1381	4.7159
252.0580	6.8181
265.2403	1.1729
300.7500	4.3844
320.6709	8.3147
333.7435	10.0272
350.4313	17.0877
373.0528	3.0261
381.1405	13.1394
390.3609	2.0408
416.3700	0.2608
423.8757	0.0495
429.4118	12.3172
448.1118	29.4722
474.5566	5.3792
489.4522	4.1074
519.2223	9.0412

Frequency (cm⁻¹)	IR Intensity (KM/Mole)
523.7226	48.6301
552.8012	3.6213
574.4706	79.9931
588.8473	31.0989
614.5172	6.8488
630.5417	2.6233
633.8041	1.0708
654.9853	32.1097
665.0342	13.9913
696.7967	50.6969
710.6905	48.5591
742.2723	10.5227
751.1548	4.8576
756.0881	13.9064
757.7544	64.0034
790.1451	12.7741
794.6468	27.5339
806.7152	34.8844
831.1172	45.7318

Frequency (cm^{-1})	IR Intensity (KM/Mole)
840.7503	43.7606
859.6832	1.2955
866.3181	2.0994
886.3290	16.9086
894.5462	20.7822
931.1313	4.8474
933.0365	4.4413
942.4735	5.2282
961.6911	14.2592
963.9727	4.6464
966.2129	0.1933
982.6265	0.3094
991.4864	169.8233
998.0327	72.3339
1002.0449	0.5297
1014.5929	18.2045
1015.2592	1.1325
1023.4513	8.1734
1038.1028	16.6381

Frequency (cm⁻¹)	IR Intensity (KM/Mole)
1052.9591	6.9270
1072.5317	37.2143
1092.8523	24.3497
1102.5734	8.9436
1106.0613	5.8016
1123.0428	1.7724
1130.3586	56.4032
1167.6418	18.4229
1180.1004	0.0413
1188.8233	11.0149
1193.2509	58.3893
1201.9311	0.5548
1204.4340	34.8075
1206.4337	1.7000
1222.7380	27.7792
1229.2138	1.8940
1250.2297	6.4514
1273.4073	1.3019
1304.5851	78.6577

Frequency (cm^{-1})	IR Intensity (KM/Mole)
1315.7324	0.9848
1322.7973	14.0227
1336.6404	2.4601
1337.4197	2.1501
1350.3721	72.4537
1358.8876	5.8284
1366.1147	2.3920
1375.2698	2.3187
1388.0967	13.9706
1399.9817	7.3857
1405.3504	94.2047
1423.1075	64.3990
1425.2184	28.8792
1450.7459	72.7491
1456.9122	107.8957
1477.7970	0.6520
1483.1605	26.6707
1486.5941	4.8240
1494.8541	14.9004

Frequency (cm^{-1})	IR Intensity (KM/Mole)
1496.6399	3.6955
1504.5218	4.7373
1505.0454	9.6563
1510.7595	20.7787
1522.5726	13.4715
1531.0171	11.2894
1536.3345	300.4647
1585.6593	5.6299
1619.6764	8.8978
1628.5589	2.3409
1651.7528	12.1116
1694.3339	518.9749
1700.1788	415.2584
2972.1256	67.6063
3007.8379	31.7341
3015.0395	86.3815
3017.5589	12.7530
3027.7716	42.6060
3035.6594	21.9228

Frequency (cm^{-1})	IR Intensity (KM/Mole)
3045.5407	35.7455
3066.5976	6.3824
3076.2172	97.9407
3089.8849	12.8654
3090.5536	45.4335
3107.9395	11.8862
3117.6755	6.5346
3139.1373	79.9214
3160.1049	7.5712
3171.5453	3.9419
3176.3164	29.7467
3182.0406	33.7606
3193.7144	25.2487
3202.4764	11.9427
3203.2017	5.9300
3445.9040	157.0645
3562.5725	12.0920
3823.2631	8.1502

Bortezomib (de1) in diethylether

Frequency (cm^{-1})	IR Intensity (KM/Mole)
24.5947	0.9415
32.3362	1.3519
39.9083	1.3437
51.0484	2.4662
55.7081	0.5425
61.4053	1.3500
64.0928	2.0382
73.7231	1.1417
87.2921	1.2431
90.0948	1.9107
96.7365	5.6138
105.8337	2.7206
114.1952	0.5806
127.8235	15.9251
140.7942	0.9462
170.9196	22.0542
181.3714	0.1546
203.0620	3.0938

Frequency (cm^{-1})	IR Intensity (KM/Mole)
208.5247	0.1118
235.3742	11.3805
238.7035	0.9723
252.6334	8.8489
295.1783	2.1466
298.7049	4.9059
320.8250	17.6005
336.5468	13.2708
348.9068	19.2246
366.1056	7.2278
385.0311	16.0459
399.4809	1.2021
418.3010	0.2466
424.2225	12.2607
426.6811	8.2708
443.1275	37.2288
470.5355	36.1271
487.6955	1.2727
517.6080	19.8561

Frequency (cm⁻¹)	IR Intensity (KM/Mole)
529.0539	65.6850
552.3673	101.5388
560.1832	31.4912
584.8046	35.1260
613.6644	2.1577
631.9966	3.8879
633.0692	0.8580
652.5892	50.4846
665.9996	5.5320
695.1292	97.2973
712.4096	62.4349
737.7710	47.2067
748.5905	13.8197
751.6761	84.4232
758.0632	7.9612
776.0405	91.9076
786.8228	14.2758
798.4333	3.7650
827.8313	36.2435

Frequency (cm^{-1})	IR Intensity (KM/Mole)
839.0555	30.6931
862.1140	1.1606
865.7113	0.9295
878.1721	26.8938
892.9760	26.7693
934.7479	7.5054
935.5149	9.2511
944.8423	2.6405
960.6847	33.2396
963.4094	16.3509
967.5938	0.4918
981.0040	258.2454
988.9855	0.1113
997.7581	21.1199
999.9166	22.5223
1005.4938	0.5842
1013.6306	0.4460
1021.1491	16.6182
1036.0284	28.4684

Frequency (cm⁻¹)	IR Intensity (KM/Mole)
1052.0286	9.3657
1072.9359	37.4947
1092.9878	38.7355
1103.1611	15.6205
1107.6592	12.5529
1120.7502	53.8705
1124.0845	44.7893
1166.0675	21.2426
1179.3330	0.0209
1188.7555	14.5585
1196.1096	70.5759
1201.3006	1.0486
1204.5928	1.2143
1209.3219	38.5605
1223.8387	34.5504
1230.2792	6.8476
1248.0991	12.3589
1273.9360	1.9124
1309.2489	132.0321

Frequency (cm⁻¹)	IR Intensity (KM/Mole)
1319.5162	6.1546
1327.6786	17.2686
1328.6124	9.2278
1341.3646	0.7457
1352.6100	57.2231
1357.0987	2.8999
1368.3468	3.2578
1374.5834	3.2108
1392.8135	18.4536
1402.9438	6.0225
1411.4476	120.4143
1423.7415	15.0683
1424.6511	132.9482
1451.3651	94.7362
1462.3940	166.4031
1477.7619	1.4461
1486.2723	11.7509
1487.4265	35.7539
1493.1086	23.7457

Frequency (cm⁻¹)	IR Intensity (KM/Mole)
1495.7965	6.3599
1502.2706	101.0683
1505.5443	83.8713
1508.6094	131.0067
1517.4708	188.9542
1524.8411	17.8593
1530.8064	12.1326
1585.0576	12.5483
1622.4306	16.9100
1627.7480	3.3689
1649.4575	19.8719
1672.4143	729.0527
1681.7025	697.9210
2993.6145	71.5949
3013.9576	34.9666
3017.2469	10.6353
3021.2540	86.3655
3039.2404	39.3614
3043.5043	55.7255

Frequency (cm^{-1})	IR Intensity (KM/Mole)
3044.2217	65.6688
3073.3488	0.4399
3080.3982	138.0913
3097.0734	6.9416
3101.4561	52.8250
3113.8597	20.9421
3120.8589	12.4927
3168.6646	10.6022
3178.7510	0.9867
3187.3884	22.9319
3188.6759	18.0761
3195.4497	28.5256
3203.6427	19.1913
3204.0137	22.8797
3206.5908	7.6224
3477.7475	188.8108
3562.5223	34.1905
3824.1152	18.6506

Bortezomib (de1) in methanol

Frequency (cm^{-1})	IR Intensity (KM/Mole)
19.1400	0.1437
22.0261	2.8614
23.6429	1.4202
29.8995	0.8939
34.9057	1.0775
38.0735	0.7564
42.3284	0.2081
54.5036	0.8706
56.8671	1.1385
78.7931	2.9297
86.8589	1.4666
98.7012	3.3483
105.6439	2.4746
114.3432	11.7610
157.2514	11.9914
167.9626	0.9489
183.8258	2.8467
185.1212	1.6223

Frequency (cm^{-1})	IR Intensity (KM/Mole)
211.0109	3.1104
230.1848	8.9407
248.0430	2.5043
252.1961	21.7270
257.2278	0.4997
289.5168	11.9986
328.8609	9.4614
339.3851	31.2167
348.5947	1.7889
369.9834	5.6954
371.5484	23.7224
394.0489	3.2453
409.9923	11.9318
415.6725	0.0340
420.9940	0.4297
437.2182	26.6537
465.9058	51.4736
476.4863	34.5660
516.8694	2.2209

Frequency (cm^{-1})	IR Intensity (KM/Mole)
537.4747	94.9195
545.1205	124.3888
568.0442	52.1410
587.2711	25.7879
623.4480	6.3731
627.3510	9.5911
632.5649	0.5137
646.6962	91.8095
654.5160	39.5067
701.3863	84.1535
710.8441	58.1443
727.4078	78.1246
736.8567	54.9894
746.4269	12.6859
763.8772	47.9833
774.9793	66.2678
783.0245	9.1189
793.5603	19.9614
816.0673	10.0657

Frequency (cm^{-1})	IR Intensity (KM/Mole)
838.9646	40.7309
850.4749	16.7682
861.9181	0.1799
879.4029	19.8129
890.0130	33.4869
924.6192	9.0225
932.6297	6.5116
944.4709	5.2852
964.4797	0.9824
967.3395	2.3491
974.2645	191.4032
984.8639	125.8590
989.6289	1.7210
1000.1670	1.6261
1000.8593	9.5898
1006.1312	0.0722
1012.7918	0.0415
1035.0210	43.8539
1048.6805	6.4533

Frequency (cm⁻¹)	IR Intensity (KM/Mole)
1053.5635	33.6416
1072.5266	15.3195
1095.8184	12.2213
1101.7011	1.1262
1109.4265	50.2499
1116.2420	118.5708
1120.0937	38.4259
1163.7676	30.6065
1176.5230	0.0520
1186.8159	12.4043
1190.9732	95.1546
1193.4432	1.8320
1197.9981	0.3875
1202.9805	6.7605
1223.2522	34.5828
1224.7392	6.8365
1244.9889	24.7738
1271.5730	3.9076
1292.7645	43.0851

Frequency (cm⁻¹)	IR Intensity (KM/Mole)
1312.0437	19.9149
1318.3520	2.4779
1321.6909	10.5221
1339.6375	8.3097
1351.7089	7.4995
1358.5241	9.4058
1367.9708	1.1769
1371.4530	16.2457
1391.7858	28.0996
1396.6182	299.9798
1399.6328	8.8232
1418.6762	17.4577
1423.1272	113.1552
1448.2559	101.1401
1459.1591	189.7796
1475.2925	6.1859
1479.8441	47.5699
1484.5003	2.3993
1485.7141	78.2533

Frequency (cm⁻¹)	IR Intensity (KM/Mole)
1487.9760	254.1100
1491.7327	6.1975
1493.7126	280.0126
1496.3589	9.6184
1507.4023	82.0813
1510.9988	23.4304
1528.6928	18.2326
1583.4269	10.7054
1618.4891	42.9352
1626.1113	2.8225
1647.6390	28.6102
1659.8261	1009.5925
1671.3811	915.4087
3002.9026	62.2372
3012.4683	14.2367
3017.6030	46.7011
3024.1617	96.0754
3029.2415	72.4695
3050.5534	78.3462

Frequency (cm^{-1})	IR Intensity (KM/Mole)
3067.3850	30.7482
3078.3853	0.9111
3083.9000	159.0101
3102.3656	54.2439
3117.9767	12.0084
3121.4932	27.3962
3175.4403	5.8622
3180.9040	0.5206
3182.7397	5.5737
3189.5652	23.0253
3190.9171	1.1157
3196.1916	48.8240
3205.0053	39.5792
3205.1995	26.1560
3214.7574	1.2521
3479.8431	229.8673
3566.7687	85.5078
3816.4498	22.8566

Bortezomib (de1) in water

Frequency (cm^{-1})	IR Intensity (KM/Mole)
7.6355	2.0755
20.2149	0.6947
22.1526	2.1025
24.6047	2.0274
30.0837	0.4982
37.3943	0.0285
41.1821	0.1571
53.7233	1.4034
55.9347	0.7821
76.0861	2.8414
86.3278	1.5544
98.3207	3.5184
103.8120	3.0480
112.0030	11.5945
156.5721	11.7456
167.8270	0.9289
183.5859	2.6413
184.9957	1.8812

Frequency (cm^{-1})	IR Intensity (KM/Mole)
210.6074	3.1708
228.7044	7.5216
247.5524	2.1108
250.0179	16.8817
252.5218	7.4785
289.2376	12.3023
328.4092	8.9468
339.4046	32.3792
348.4719	1.7769
369.6782	6.7751
371.2725	22.9712
392.1600	3.6641
409.9192	12.2619
415.2913	0.0342
420.8523	0.4302
437.1131	26.9933
464.8824	52.6026
476.4321	36.2594
516.7430	2.0616

Frequency (cm^{-1})	IR Intensity (KM/Mole)
536.9766	95.8561
544.0639	128.9624
567.3445	52.0612
587.2567	26.4588
622.6793	7.9169
627.1945	9.4081
632.5174	0.5672
645.9586	98.5283
654.4034	36.1870
700.6495	92.7864
710.5578	58.4484
725.4135	81.2161
736.6978	53.5296
746.1146	12.0058
763.5932	53.3339
773.6432	61.8910
782.9436	8.7821
793.3439	18.7804
815.9111	9.8930

Frequency (cm^{-1})	IR Intensity (KM/Mole)
838.5738	40.4140
850.2839	17.1029
861.7054	0.1034
879.6041	20.1541
889.6438	33.6456
923.9558	9.1166
932.5197	6.5591
944.1946	5.2203
964.0858	1.2378
966.8765	2.3329
974.0042	195.4443
984.7586	126.1083
989.6186	1.3378
1000.5001	6.8209
1000.8788	5.1181
1006.4221	0.0751
1012.7560	0.0529
1034.9362	44.3435
1048.6039	6.7522

Frequency (cm^{-1})	IR Intensity (KM/Mole)
1053.5837	34.1376
1072.4457	15.5266
1095.8318	12.7462
1101.8273	1.2354
1109.4051	52.0333
1115.9922	122.0983
1119.7667	38.8597
1163.5696	31.2145
1176.3458	0.0410
1186.5425	12.7050
1190.9726	96.7437
1193.5138	1.8370
1197.8828	0.4100
1202.8816	7.1721
1223.3979	33.6076
1224.7281	8.4816
1244.8150	25.9230
1271.4499	4.0192
1293.0961	44.4992

Frequency (cm⁻¹)	IR Intensity (KM/Mole)
1311.9488	20.2538
1318.3274	2.5095
1321.6554	10.9928
1339.2267	8.1804
1351.5037	7.6051
1358.5178	9.4053
1367.7762	1.2719
1371.7342	16.3296
1391.4584	28.8919
1396.4377	306.7035
1399.3341	9.4932
1418.1976	17.4846
1423.1283	118.7561
1448.2502	102.9225
1458.9906	199.6581
1474.8693	9.9536
1479.3177	55.2277
1484.4052	2.4017
1484.7265	131.9340

Frequency (cm^{-1})	IR Intensity (KM/Mole)
1486.3255	261.3460
1491.1442	3.2989
1493.3675	224.1145
1495.7324	7.6233
1507.3282	86.2398
1508.6900	20.1782
1528.6557	18.4572
1583.3863	10.9458
1618.5153	45.6652
1626.0286	2.8832
1647.4997	30.0155
1658.2488	1023.2099
1670.1468	930.4931
3003.3904	63.1507
3011.7059	16.8453
3017.8487	47.5882
3024.2940	94.1829
3030.1493	75.0350
3050.4375	80.1969

Frequency (cm^{-1})	IR Intensity (KM/Mole)
3067.6664	31.0860
3078.8625	0.9464
3084.2003	162.3402
3101.9533	54.6506
3118.2886	12.1755
3120.1259	29.2868
3175.8747	5.9882
3181.0983	0.5571
3182.5108	5.5249
3189.8586	22.8731
3191.6856	0.9655
3196.4115	50.7167
3205.4384	20.1568
3205.5912	46.2150
3214.3478	1.1058
3482.5015	227.2267
3566.4335	86.8124
3816.2516	23.2721

Bortezomib (de2) in gas phase

Frequency (cm^{-1})	IR Intensity (KM/Mole)
4.8527	1.8362
13.8422	0.0815
20.8885	1.1353
25.1098	0.9312
30.4575	0.2286
38.6435	1.3189
46.7070	1.7040
51.4736	0.4238
56.7293	0.2951
66.7574	3.6724
73.2045	0.6713
100.5766	1.3378
112.6062	3.6419
123.4202	7.9497
162.5140	1.3640
169.0485	6.4081
179.1578	1.5517
199.6513	0.2436

Frequency (cm^{-1})	IR Intensity (KM/Mole)
208.2290	2.9727
224.7216	1.1314
241.1174	2.2497
256.8865	2.1357
263.7092	2.3317
289.3757	5.6991
322.5105	1.1525
339.5931	9.0073
352.4910	6.0946
355.7946	3.6047
382.6597	7.4867
391.6369	8.4726
417.5462	6.7415
418.1532	14.1281
427.1637	0.0942
442.6769	44.7944
461.2198	0.2321
475.9831	12.0060
506.1717	11.0008

Frequency (cm^{-1})	IR Intensity (KM/Mole)
542.0177	26.3606
569.2626	37.9663
575.0717	79.5471
586.3539	47.5888
612.7308	15.2255
615.6020	13.9886
632.4158	2.4142
634.5490	3.1226
643.1057	15.4469
692.5574	40.3053
711.6290	47.5777
730.8430	5.2192
749.5706	35.2413
750.4016	18.6932
761.8060	21.7113
769.5046	57.3130
785.7012	8.1617
802.0585	2.8525
814.0187	24.2425

Frequency (cm⁻¹)	IR Intensity (KM/Mole)
826.3746	2.9828
860.4504	3.8663
862.6332	5.9022
881.5546	14.8358
893.1990	19.9374
922.1348	50.8288
931.0669	4.7158
935.0350	3.9383
951.9326	1.8741
957.4864	3.0903
965.5093	22.7698
970.6376	155.0808
983.2152	0.4140
986.7132	0.5431
988.3517	13.3032
1003.3532	2.4307
1015.4954	0.0847
1033.1560	24.4291
1052.0308	2.0034

Frequency (cm^{-1})	IR Intensity (KM/Mole)
1053.3263	14.1834
1067.0116	27.6308
1084.9823	4.8169
1100.3947	15.1502
1105.4052	36.7374
1108.5952	27.9836
1115.5176	0.6101
1163.0413	7.5169
1179.8928	0.0711
1188.4299	39.3943
1188.9602	50.0352
1199.9941	0.7836
1205.3502	11.3768
1207.5817	25.3932
1210.3036	2.5408
1223.2940	0.0800
1245.3765	3.7761
1270.5095	1.9412
1311.4092	4.5828

Frequency (cm⁻¹)	IR Intensity (KM/Mole)
1312.8159	40.1094
1317.3169	36.4304
1320.0113	7.9237
1337.3478	2.1426
1355.8537	43.6777
1358.6576	54.5650
1365.0188	1.8680
1377.5629	3.6167
1386.4488	2.7773
1400.5464	6.1919
1412.0100	85.6966
1425.4339	31.7734
1426.4221	22.1465
1448.4153	54.5920
1455.4095	44.0554
1480.7032	10.5380
1482.7127	15.6046
1485.0834	10.9351
1490.7685	36.0881

Frequency (cm^{-1})	IR Intensity (KM/Mole)
1493.9637	3.5549
1496.2507	6.5181
1504.7583	7.7474
1508.3508	32.3151
1517.6575	16.2241
1529.9623	17.9140
1577.8959	449.5405
1589.3966	2.5991
1620.2563	2.2702
1627.4423	2.5349
1650.7232	13.1296
1699.9873	515.5829
1711.3888	540.1873
2959.2281	89.1527
3008.5188	22.0491
3009.9178	35.5936
3017.5161	81.0152
3040.2064	23.7168
3049.0250	29.3250

Frequency (cm^{-1})	IR Intensity (KM/Mole)
3056.2684	17.5985
3072.7016	27.2505
3081.1132	61.6942
3084.5482	65.3551
3104.5900	1.8494
3122.0521	8.3105
3125.4180	10.4704
3159.4047	7.5405
3168.9805	9.3085
3170.4745	4.1618
3181.4756	36.7811
3185.0056	28.4535
3190.2098	21.4887
3193.9140	29.6473
3207.6117	9.5248
3553.0065	105.3552
3581.1742	19.8948
3832.3526	11.6032

Bortezomib (de2) in diethylether

Frequency (cm⁻¹)	IR Intensity (KM/Mole)
15.7065	1.3700
20.2195	0.1219
22.9774	1.1810
25.4927	0.6881
30.8518	0.6677
42.2067	0.6980
47.4879	0.7783
51.7707	1.8169
58.3360	0.9696
75.6647	4.2472
77.4643	1.3033
106.2857	2.8046
111.7675	4.4394
119.9064	12.6650
161.9517	6.6668
164.8944	2.5939
189.1834	0.6120
194.0963	0.6966

Frequency (cm^{-1})	IR Intensity (KM/Mole)
209.4885	1.4057
226.6980	0.9977
241.7800	4.0882
248.5403	0.3829
258.2186	3.3034
285.6346	8.2185
330.3740	3.7090
341.3460	15.4275
349.0111	6.7599
357.7567	8.2123
378.4242	13.0476
396.9518	5.1316
414.9799	31.6669
417.3093	0.2373
430.4033	1.0421
449.1843	50.7882
461.8873	11.7509
479.3812	23.1272
505.6576	13.4387

Frequency (cm⁻¹)	IR Intensity (KM/Mole)
542.4874	46.9120
565.1058	148.4071
574.1765	32.5985
598.6176	76.7093
606.9290	26.4080
616.3807	14.0630
630.5018	4.5231
632.9626	1.2567
636.0196	8.1055
690.5065	94.7742
710.0317	44.7910
713.4398	80.2242
737.1985	16.9159
750.5241	0.4918
764.1492	40.4320
767.9453	47.4084
782.3491	3.0642
797.1833	2.7889
814.0383	14.5626

Frequency (cm⁻¹)	IR Intensity (KM/Mole)
829.9952	8.5842
862.2850	1.6561
863.8766	11.1892
880.5206	25.2959
893.6036	24.3833
924.7918	61.2549
934.9386	7.8469
936.0632	21.0655
953.9363	5.9533
963.1829	8.0854
965.5690	9.1557
971.1555	207.0928
988.7528	0.5658
991.8467	1.4745
992.8575	11.0237
1004.4664	0.8966
1013.8014	0.0666
1032.7167	33.8283
1049.8277	8.4931

Frequency (cm^{-1})	IR Intensity (KM/Mole)
1051.1069	14.3584
1069.8621	36.5493
1087.5091	4.5539
1103.2490	32.6885
1109.1261	40.5358
1111.5707	8.1421
1114.7082	62.4664
1167.9005	23.2087
1178.1197	0.0962
1187.9904	10.3979
1190.8754	67.7511
1198.8851	2.1008
1202.6821	3.8413
1209.2854	56.5790
1216.6370	4.2653
1223.0467	1.3035
1236.5931	11.4186
1270.5472	5.9475
1313.4197	34.4569

Frequency (cm^{-1})	IR Intensity (KM/Mole)
1315.9547	48.5365
1319.9909	46.0224
1322.7621	8.5815
1338.8016	0.5890
1353.9619	18.2381
1361.4122	71.8537
1367.1040	39.0826
1374.8485	4.8865
1390.4028	5.4585
1401.0007	5.9487
1416.8958	130.3083
1423.2813	10.6641
1428.2205	75.3847
1446.5274	66.2189
1464.2584	89.5438
1482.7006	16.8424
1482.9963	21.7467
1485.7548	6.8429
1488.9845	30.5769

Frequency (cm^{-1})	IR Intensity (KM/Mole)
1493.5125	53.1162
1496.9000	6.2390
1500.4039	23.3072
1511.4494	34.9633
1514.6668	68.2339
1528.7817	547.1694
1529.7195	76.6295
1586.8427	4.0668
1621.8763	9.9951
1626.8807	2.4422
1648.8306	15.8702
1687.1355	763.4523
1691.4207	749.2797
2990.2344	87.0484
3013.6614	10.6853
3015.7964	52.7765
3025.9176	71.4997
3049.4380	33.3534
3052.7790	66.0223

Frequency (cm⁻¹)	IR Intensity (KM/Mole)
3060.9781	32.7625
3077.8770	9.9435
3084.9580	119.8119
3091.1769	71.1781
3115.8062	1.0331
3129.2568	19.1795
3129.8618	14.8791
3167.3239	11.9514
3178.3412	1.4791
3183.9789	2.1872
3186.7001	22.8777
3188.1169	35.6722
3199.8192	44.8067
3199.8830	21.9115
3208.5420	9.8030
3573.6631	109.0577
3581.5935	45.8045
3827.5637	22.4656

Bortezomib (de2) in methanol

Frequency (cm⁻¹)	IR Intensity (KM/Mole)
16.3080	2.3731
21.2209	0.9410
23.2501	1.7038
25.4135	1.4719
34.6102	0.4806
40.6523	0.2541
44.4214	1.5525
47.7301	1.9907
56.4125	0.3796
70.6515	9.4550
74.5561	0.2761
98.9601	2.4389
105.3824	7.7658
111.8487	11.4243
159.4398	3.6486
159.9558	4.3969
183.3011	2.4883
194.7000	0.7862

Frequency (cm^{-1})	IR Intensity (KM/Mole)
197.6191	2.3985
227.0288	0.8362
243.5731	2.5382
253.0777	1.4148
261.2742	4.1821
284.4493	8.8752
328.1577	2.4666
341.7565	13.9943
345.5845	25.9720
357.0103	0.7774
373.1085	14.2738
389.1704	4.2239
412.6441	33.6716
414.8155	0.1484
418.5732	0.5685
447.4230	75.3589
460.9587	2.5789
472.2404	50.0831
505.7817	16.1380

Frequency (cm⁻¹)	IR Intensity (KM/Mole)
538.8389	74.4605
556.4078	105.1027
568.3684	52.0154
603.0599	116.9750
604.0822	6.4269
610.1453	42.2021
627.2596	10.7990
632.4104	2.2844
632.9198	8.5660
681.7266	178.1261
697.4633	15.3806
709.5090	76.0454
737.2542	9.8549
747.9902	2.3697
761.0500	37.7093
767.2767	55.6482
782.7696	6.7699
795.9236	2.7431
813.0725	16.6633

Frequency (cm⁻¹)	IR Intensity (KM/Mole)
828.8686	6.7349
859.3972	0.0379
866.5038	9.5720
882.3146	34.9799
888.0084	20.8747
921.7184	53.8293
934.4961	8.7946
935.2924	32.3668
953.2969	11.8110
964.0176	15.2938
965.9050	6.3540
970.3616	236.5285
987.7858	0.3359
989.9073	16.4457
996.8466	0.4076
1005.4512	0.1914
1013.0517	0.0702
1033.2249	38.1463
1049.1879	12.5872

Frequency (cm^{-1})	IR Intensity (KM/Mole)
1051.2451	17.3904
1070.7581	40.7579
1088.2629	4.0410
1102.6152	35.8910
1108.5805	28.7357
1111.1424	39.4290
1114.3350	76.7074
1167.6801	41.2472
1177.3425	0.1773
1186.3224	12.0369
1191.4027	71.5750
1198.2570	4.1849
1199.9354	2.2106
1210.5213	60.3392
1219.0538	5.5841
1222.7396	3.4573
1233.8519	20.3226
1262.4561	11.0072
1315.3953	103.5380

Frequency (cm^{-1})	IR Intensity (KM/Mole)
1315.8771	28.2606
1322.1854	27.3758
1324.7533	6.1976
1338.8638	0.2312
1352.4836	21.9543
1359.4387	38.1578
1370.3232	28.4357
1372.3373	63.5816
1388.4594	5.5394
1399.1234	8.3545
1417.2761	92.2652
1417.8455	37.3147
1425.6415	95.9466
1445.1739	84.1253
1462.6215	162.7043
1471.4418	57.8946
1480.2798	30.6755
1484.4450	17.9357
1486.2295	15.4528

Frequency (cm^{-1})	IR Intensity (KM/Mole)
1489.9358	17.9758
1494.5490	92.2161
1495.1745	88.1504
1503.6385	492.9067
1508.6471	96.0869
1517.3621	79.4634
1528.7832	17.2669
1585.8418	3.8308
1621.0983	16.4484
1626.4503	1.6369
1647.4057	18.8227
1675.7549	1041.4032
1681.0308	735.2355
2999.9215	88.3189
3011.4479	17.2643
3018.4485	62.3921
3026.9668	66.6334
3052.5608	58.7766
3055.3057	64.9134

Frequency (cm^{-1})	IR Intensity (KM/Mole)
3064.9482	34.6471
3081.0106	6.4876
3085.9655	152.9835
3093.7402	76.4473
3114.1939	3.9670
3121.5550	32.4174
3133.1495	20.2182
3170.6267	14.2404
3180.9160	0.4047
3190.1849	0.9175
3190.2580	35.3175
3194.3296	23.9998
3200.8858	39.7345
3205.1605	31.4261
3207.8178	14.1512
3577.1856	99.2485
3580.8599	60.9710
3819.6410	26.5298

Bortezomib (de2) in water

Frequency (cm⁻¹)	IR Intensity (KM/Mole)
16.5050	2.4727
18.7777	0.5715
24.3093	2.1146
24.5492	1.1730
34.9407	1.1531
38.4240	1.2028
42.1376	0.6833
48.2297	1.5928
57.1577	0.3587
72.8130	9.5803
75.6115	0.1918
98.7543	2.9655
105.7793	6.9794
111.3711	11.6814
159.8760	4.8145
160.7305	3.5297
185.0354	1.9538
194.8267	0.6264

Frequency (cm^{-1})	IR Intensity (KM/Mole)
198.7163	2.9827
227.7594	0.7549
243.9522	2.5045
254.5504	1.5318
261.7272	4.0357
284.5217	8.9081
328.4407	3.4519
342.3526	15.1626
346.5100	25.6247
356.2656	0.5615
371.9005	13.8714
389.7454	4.0631
412.9125	34.3696
414.6447	0.0800
420.3570	0.6861
448.1509	77.3414
461.0471	2.5295
472.2262	53.1235
505.6743	16.3218

Frequency (cm⁻¹)	IR Intensity (KM/Mole)
538.7359	76.6065
556.0744	103.8579
568.3791	53.3614
603.4161	123.5307
604.1345	2.9739
611.1025	40.1034
627.1805	11.8586
632.2808	2.9204
633.0045	8.2968
682.6694	179.6663
697.9772	18.5696
709.3532	78.2679
737.5096	9.5474
747.9028	3.4201
760.5062	37.2345
766.9896	56.8380
782.9160	7.2344
795.8437	2.8843
813.2104	16.7349

Frequency (cm^{-1})	IR Intensity (KM/Mole)
829.0986	6.9792
858.9742	0.0258
866.7339	8.8454
882.4811	36.0145
888.4486	20.7341
921.4135	54.5291
934.2631	7.4513
935.4096	36.1303
953.1658	13.5013
963.9133	18.2059
966.3818	10.5653
969.8308	231.8803
987.6193	0.3088
990.7987	16.0048
997.6430	0.4164
1005.5377	0.1750
1012.9325	0.0691
1033.2160	38.4808
1048.9960	12.2174

Frequency (cm^{-1})	IR Intensity (KM/Mole)
1051.7279	19.0842
1070.6686	41.1874
1088.3078	4.6239
1102.5170	34.5649
1108.5555	27.6940
1111.2014	42.6728
1114.7210	81.6910
1167.9339	42.5412
1177.3285	0.1676
1186.2362	11.7461
1191.5335	72.3029
1198.0855	4.3618
1200.0228	3.7826
1210.6976	59.5244
1219.1535	5.8032
1222.7419	3.7996
1233.5726	21.6081
1264.2433	11.2082
1315.1957	30.5932

Frequency (cm^{-1})	IR Intensity (KM/Mole)
1315.9493	109.8480
1322.3082	25.2794
1325.0175	4.4335
1338.4896	0.3009
1352.2576	23.4176
1359.2547	36.6512
1370.5264	23.7341
1373.0283	68.7958
1388.5144	5.3344
1398.9428	8.5510
1417.2386	44.8371
1417.6853	86.2704
1425.7571	97.3681
1445.2937	86.3813
1462.7767	177.8015
1472.6009	55.6729
1479.4155	33.6765
1484.1093	18.0154
1486.2018	16.1619

Frequency (cm^{-1})	IR Intensity (KM/Mole)
1489.6923	22.7636
1493.9072	141.7242
1495.3064	77.3347
1501.9797	496.9943
1507.8885	52.8134
1517.5385	80.1229
1528.7739	17.3807
1585.6732	3.8350
1621.0057	17.0379
1626.3213	1.4905
1647.2481	19.3821
1674.3426	1075.6041
1679.8540	734.9964
3000.9699	87.7272
3011.2923	17.6346
3018.6565	62.4258
3026.6921	66.0893
3051.2800	70.5133
3055.8199	60.4242

Frequency (cm⁻¹)	IR Intensity (KM/Mole)
3064.9888	34.7854
3081.1408	5.9646
3085.9849	156.9407
3094.1044	76.5945
3113.8947	4.1078
3120.9244	34.0211
3133.3659	21.0478
3170.9897	14.4867
3181.1015	0.3004
3190.4412	35.0584
3190.8387	0.7990
3195.3362	24.2804
3200.8462	41.2155
3205.7667	30.6782
3207.6795	14.7679
3577.5520	99.0365
3580.3427	62.8261
3818.4018	27.3314

Threonine calculations at level of theory - B3LYP/6-31+G(d,p)

Threonine (Thr1-OH) in gas phase

Frequency (cm^{-1})	IR Intensity (KM/Mole)
59.7591	0.8790
79.8950	0.5082
161.5920	0.1213
226.6977	8.1661
238.0533	10.5530
273.1367	12.2070
295.6190	58.4334
312.3007	129.0367
374.5317	11.0238
398.1834	5.7028
483.6770	6.3229
521.2309	17.2999
621.6103	113.4149
668.2660	16.0334
756.9012	17.1468
811.9812	4.8345
844.3270	126.5609

Frequency (cm⁻¹)	IR Intensity (KM/Mole)
914.3744	44.5393
959.7081	50.5529
1049.9767	18.4856
1094.4478	7.8549
1119.1236	4.7868
1130.2986	63.4387
1148.1346	240.6051
1226.8991	52.3369
1272.5647	41.7559
1285.6251	12.9011
1322.1408	35.8172
1364.6671	19.0164
1399.8806	8.3530
1431.5105	14.9704
1442.8833	17.8525
1498.6077	2.6398
1504.7498	7.1940
1675.4303	43.2801
1809.6483	270.6362

Frequency (cm⁻¹)	IR Intensity (KM/Mole)
3018.5394	36.5215
3058.2486	14.4849
3066.8174	19.1725
3132.9195	15.9340
3139.3164	15.6661
3510.2451	2.3610
3596.3522	7.9103
3739.2693	56.6177
3817.3202	22.8025

Threonine (Thr1-OH) in diethylether

Frequency (cm⁻¹)	IR Intensity (KM/Mole)
50.4676	1.5323
85.1141	0.8603
163.9780	0.0186
229.3288	8.7723
239.1128	11.0325
266.1352	21.6695
296.6496	71.6156

Frequency (cm⁻¹)	IR Intensity (KM/Mole)
316.9360	173.6627
375.3252	13.4184
399.4810	9.3759
483.5530	9.1493
521.1352	23.8627
616.4838	158.9870
668.9893	19.8420
756.7235	23.2427
812.5779	6.5138
848.7846	154.4627
915.8868	62.5801
957.5073	81.8514
1051.2354	27.4404
1094.8823	11.9463
1111.3502	8.8949
1126.7491	129.3994
1147.5467	282.6461
1222.2682	67.7730
1271.2202	58.8779

Frequency (cm^{-1})	IR Intensity (KM/Mole)
1287.7549	6.4433
1321.9500	53.2240
1368.0063	23.7305
1400.9253	11.9884
1432.0026	22.0929
1443.0566	20.6028
1490.1350	3.8743
1497.0190	8.8941
1665.6231	57.7717
1781.2334	414.5549
3029.8472	46.2226
3056.7323	18.7460
3078.7646	21.7343
3130.1549	25.5100
3136.8563	24.3950
3508.7906	3.3807
3592.2706	12.0208
3728.2492	84.8325
3811.5407	41.9706

Threonine (Thr1-OH) in methanol

Frequency (cm^{-1})	IR Intensity (KM/Mole)
46.0303	1.9347
86.3721	0.9976
164.5204	0.0231
228.2784	10.6770
239.5154	12.5645
264.9669	31.0240
296.1901	68.2570
317.0051	199.1816
375.4258	16.9589
401.1045	9.5600
483.0324	11.1523
521.6455	26.9383
611.4698	185.6830
667.9735	22.1404
755.3729	26.4216
813.1014	7.4005
850.3709	162.9594
916.2125	75.2165

Frequency (cm^{-1})	IR Intensity (KM/Mole)
956.1036	100.7805
1052.2538	31.5529
1094.4832	12.1081
1108.5886	15.7220
1125.0238	177.3058
1147.5005	285.9371
1220.7633	73.8352
1270.3878	66.0079
1286.8526	5.5092
1322.0893	65.0006
1368.0896	25.1912
1399.4675	14.6135
1432.8804	26.1426
1441.4882	22.0904
1484.9472	4.5181
1493.1819	10.1699
1661.3498	67.2181
1767.7740	501.6262
3035.4589	51.2219

Frequency (cm^{-1})	IR Intensity (KM/Mole)
3055.0156	21.9431
3086.9479	23.3585
3127.6661	32.8250
3134.9881	30.0307
3507.6282	4.4553
3590.1771	14.9942
3722.9591	96.6605
3807.8488	55.9547

Threonine (Thr1-OH) in water

Frequency (cm^{-1})	IR Intensity (KM/Mole)
45.2947	1.9862
86.0848	1.0121
164.2192	0.0277
227.9787	10.8262
239.0376	12.7113
264.4983	33.2065
295.8908	68.2203
316.2907	200.9983

Frequency (cm^{-1})	IR Intensity (KM/Mole)
375.4464	17.5480
400.9372	9.1898
482.8642	11.3866
521.7481	27.1852
610.8963	188.6043
667.7552	22.3909
755.1603	26.7185
813.1139	7.5043
850.4571	163.7609
916.3001	76.4669
955.9339	103.1492
1052.1117	32.1479
1094.4580	12.0567
1108.4262	16.7538
1124.8503	182.6766
1147.4929	285.6471
1220.6026	74.5095
1270.3612	67.2853
1286.7673	5.2382

Frequency (cm^{-1})	IR Intensity (KM/Mole)
1322.0830	66.3467
1368.0782	25.2044
1399.1888	14.5342
1432.8520	27.1753
1441.0676	22.1064
1484.3534	4.5757
1492.7243	10.3017
1660.8758	68.2932
1766.4491	511.8085
3036.0243	51.7261
3054.9133	22.3179
3087.8781	23.5812
3127.5127	33.7911
3134.8511	30.6491
3507.5460	4.5666
3589.9836	15.3120
3722.6131	97.7991
3807.3423	57.8028

Deprotonated threonine (Thr1-O-) in gas phase

Frequency (cm^{-1})	IR Intensity (KM/Mole)
32.0511	6.9631
82.3039	2.1966
147.0571	11.9240
155.3378	21.6713
230.0609	4.0284
256.7128	3.6170
308.6169	3.4396
347.6302	35.9863
411.8483	16.5234
473.0524	71.1108
476.9445	14.2281
576.6037	77.5721
631.9448	68.3302
638.5401	34.2617
765.2356	44.3200
815.9968	85.7829
821.1302	89.0732
903.0753	39.1688

Frequency (cm^{-1})	IR Intensity (KM/Mole)
1024.8843	8.0270
1061.1182	33.6100
1083.9332	198.2332
1136.7025	142.4584
1164.2917	18.9519
1214.7777	112.9231
1276.0363	141.1779
1304.3537	22.9091
1316.8422	28.4880
1353.5668	19.8371
1367.5800	215.7137
1436.6014	53.0285
1475.9255	0.5555
1489.9202	5.2419
1624.3761	72.4125
1739.6672	421.6181
2535.1140	510.7594
3000.1945	52.2424
3022.3233	39.6586

Frequency (cm⁻¹)	IR Intensity (KM/Mole)
3072.6793	60.0880
3078.2820	56.6993
3488.4043	16.2227
3590.1301	1.1545
3755.1212	16.6692

Deprotonated threonine (Thr1-O-) in diethylether

Frequency (cm^{-1})	IR Intensity (KM/Mole)
81.7145	17.3207
90.1308	6.0000
160.2921	13.5380
206.8908	60.2742
212.2684	5.1083
257.8152	9.6715
308.3913	2.2942
389.9088	11.0369
408.8620	5.4635
489.3017	21.0914
504.3355	67.0386

Frequency (cm^{-1})	IR Intensity (KM/Mole)
595.7081	130.2900
647.0160	55.7755
695.3040	28.8220
784.6820	30.9138
830.3748	124.8498
850.7959	92.5049
927.3523	130.0330
1038.0279	30.0791
1072.9480	25.1802
1101.2270	198.1172
1134.2659	165.6861
1190.9292	33.0911
1203.6080	461.4078
1256.0934	55.3194
1304.1559	35.7117
1334.9410	35.9675
1347.5825	105.0733
1373.0956	30.0814
1437.7272	46.9623

Frequency (cm⁻¹)	IR Intensity (KM/Mole)
1473.2571	2.7684
1481.9713	4.0036
1638.2366	80.4605
1741.7076	558.8437
2623.4296	529.1240
3010.9049	61.5120
3019.6036	59.8046
3081.3426	75.6051
3085.8081	76.1735
3492.7278	3.0912
3579.0175	1.5749
3744.3061	55.5173

Deprotonated threonine (Thr1-O-) in methanol

Frequency (cm^{-1})	IR Intensity (KM/Mole)
57.6079	13.3347
69.0638	16.3437
154.7084	25.5030
194.3689	69.4011

Frequency (cm^{-1})	IR Intensity (KM/Mole)
223.7276	5.5915
258.3579	7.9559
311.8456	6.7507
386.9137	8.6100
413.0809	4.1342
486.2564	24.1373
508.2947	50.5672
597.3340	166.0601
649.6224	65.4311
713.7365	11.4062
789.7499	30.8610
836.0930	118.7853
859.4681	85.4312
933.2449	208.9342
1039.9795	34.8301
1071.4283	25.1228
1102.1832	193.3840
1128.6265	188.5561
1186.2952	425.5554

Frequency (cm^{-1})	IR Intensity (KM/Mole)
1197.6122	181.8500
1258.5505	20.0811
1302.6774	44.9518
1337.9903	32.4267
1349.5509	70.7017
1369.3958	42.8366
1437.1809	49.0577
1470.8658	5.1341
1478.4237	2.2484
1638.8527	81.9439
1740.0964	638.2536
2686.2854	497.7637
3013.5386	68.2833
3019.3847	66.7939
3081.4851	87.0358
3090.7949	81.0863
3495.6450	0.4489
3578.0850	3.5858
3735.6021	79.0939

Deprotonated threonine (Thr1-O-) in water

Frequency (cm^{-1})	IR Intensity (KM/Mole)
49.6485	15.1806
74.9508	16.9545
151.9674	22.9543
191.2063	72.6062
226.6325	5.7249
259.7357	6.8737
312.6146	7.7286
385.9596	8.2937
413.7653	4.4224
486.5436	23.7732
509.3467	49.5482
598.4468	169.6113
649.6203	66.1857
715.7951	10.3153
790.3699	31.7790
836.7721	116.4685
860.7002	86.0479
934.3783	217.1532

Frequency (cm⁻¹)	IR Intensity (KM/Mole)
1040.1973	34.5504
1071.3776	27.2334
1102.1561	192.6126
1127.5474	192.6487
1185.0048	467.7620
1197.6879	148.1264
1258.9937	18.2552
1302.5342	45.3596
1338.5061	32.4061
1351.0864	67.8765
1370.1832	43.0969
1437.1610	49.2449
1471.5435	5.4221
1478.4989	2.1735
1638.5968	82.4612
1739.7910	646.8094
2693.4825	492.0720
3014.3103	72.8060
3018.7233	63.9413

Frequency (cm^{-1})	IR Intensity (KM/Mole)
3082.5171	87.5048
3091.1261	81.9154
3495.8071	0.2874
3578.1563	3.8795
3733.6863	81.8666

Bortezomib calculations at level of theory - B3LYP/6-311++G(d,p)

Bortezomib in gas phase

Frequency (cm^{-1})	IR Intensity (KM/Mole)
7.3063	0.4644
17.0862	0.8817
18.9264	0.4464
23.6952	0.1911
29.6973	0.1558
38.4439	0.2509
50.4058	1.6416
53.3417	0.4637
59.0294	0.7636
65.3432	0.0827

Frequency (cm^{-1})	IR Intensity (KM/Mole)
76.6976	0.3607
97.3727	3.0915
105.2117	0.5584
122.7731	3.5139
156.6201	0.4197
167.8118	3.3865
174.1076	0.6137
197.7737	0.2544
211.3728	0.6342
226.0925	0.1147
242.2391	0.6579
254.6003	0.1225
275.0378	3.3995
294.9074	3.6632
325.2451	0.2606
342.6960	8.7127
353.8199	2.7743
366.4300	3.4805
378.2317	8.3081

Frequency (cm^{-1})	IR Intensity (KM/Mole)
389.5239	2.9292
410.8743	1.2411
415.3798	0.1957
422.2974	1.5289
424.3175	14.1254
451.4143	51.7848
463.3974	3.5008
475.0569	26.5842
509.0797	16.5681
534.9866	31.1068
558.2605	13.2347
568.0903	143.2127
579.4689	62.3154
601.7138	32.2121
615.6254	38.5900
633.9036	14.8524
635.8985	0.7762
650.0548	65.0463
677.6384	25.0096

Frequency (cm⁻¹)	IR Intensity (KM/Mole)
695.7610	37.0866
713.0742	46.7045
739.7384	7.2884
765.3443	20.6021
767.0264	6.3603
773.0150	45.9885
792.9231	19.3084
799.2235	7.5513
813.9960	18.1703
837.1168	8.8869
859.3535	0.5149
865.5925	6.7892
885.0734	15.8158
888.4187	11.2449
929.5576	8.4140
933.6352	7.3102
939.0223	128.4414
944.2710	14.1448
949.4998	13.2219

Frequency (cm^{-1})	IR Intensity (KM/Mole)
964.6610	1.4419
964.9052	1.2563
987.8680	0.7732
993.2405	1.3845
995.2066	0.0895
1004.3163	0.2569
1011.8915	117.9066
1017.7850	0.0187
1037.2443	28.7482
1046.3635	11.0426
1050.7497	7.0717
1067.3169	23.8668
1085.1913	4.1095
1109.2569	24.0458
1113.3753	3.4106
1125.1387	3.5042
1164.6409	13.0681
1176.0241	113.0947
1180.8635	4.5870

Frequency (cm^{-1})	IR Intensity (KM/Mole)
1187.5967	9.8902
1198.6352	3.4602
1203.5719	14.1155
1204.6055	4.1173
1220.2046	0.2425
1226.9789	4.7415
1233.0573	9.9765
1269.2719	23.6042
1295.3707	24.8306
1301.3296	118.2683
1310.5034	60.0851
1315.9739	6.2251
1338.8305	15.5730
1340.9447	8.9687
1352.4792	64.5858
1359.3404	96.7776
1364.8205	1.0782
1379.5676	137.7733
1383.4926	15.1379

Frequency (cm⁻¹)	IR Intensity (KM/Mole)
1398.8246	98.5126
1403.6456	115.4232
1405.5622	74.1120
1408.0259	96.4486
1424.5894	19.7909
1442.7891	76.6197
1480.0618	4.0079
1481.2747	6.2950
1482.1616	3.5125
1487.3723	29.0015
1491.4509	7.7774
1494.6566	9.6259
1497.8773	3.8587
1501.2618	14.2861
1506.8839	5.7876
1516.5176	10.9125
1525.4552	15.3364
1578.6452	1.1447
1610.2692	4.6927

Frequency (cm⁻¹)	IR Intensity (KM/Mole)
1621.2570	1.7916
1642.4997	7.7391
1718.8603	460.6121
1726.5342	413.4565
3008.5651	20.6961
3010.7856	6.3039
3012.8543	39.5400
3020.1290	23.2795
3039.1299	12.9603
3045.8606	20.1831
3046.8142	31.9409
3071.5799	8.0791
3072.5104	8.2488
3079.1381	59.4151
3084.5900	35.5370
3087.6396	43.7614
3103.3457	7.7564
3144.4065	9.9013
3155.8491	1.4578

Frequency (cm⁻¹)	IR Intensity (KM/Mole)
3160.4775	0.6042
3170.9766	21.5061
3172.7778	35.1498
3179.5653	0.0451
3183.6647	20.4573
3196.2877	4.1474
3558.5785	20.2099
3584.6558	41.8716
3857.1372	13.5182
3861.4797	91.5241

Bortezomib in diethylether

Frequency (cm⁻¹)	IR Intensity (KM/Mole)
17.8377	1.1667
20.2475	0.7835
21.4246	1.4037
23.7388	0.4425
28.9042	0.7714
39.5627	0.4586

Frequency (cm^{-1})	IR Intensity (KM/Mole)
43.3801	1.5247
48.3229	0.2344
55.8367	0.8267
71.6951	0.8115
77.1669	0.0468
96.3147	4.3412
105.7643	0.6285
113.2376	4.3458
155.9944	0.2935
160.6397	4.3449
186.8517	0.3921
194.1679	0.4461
202.6885	0.5759
226.0599	0.1941
242.0248	0.7249
255.2897	1.3081
259.7257	2.1481
290.7089	5.6206
326.4053	0.3285

Frequency (cm^{-1})	IR Intensity (KM/Mole)
338.6223	14.2248
346.8827	7.9049
355.8268	2.0540
374.5992	14.9669
389.3276	2.6503
414.7063	29.1959
415.1654	0.1308
422.2671	1.3478
445.0280	58.8467
458.5038	8.8013
470.2267	9.4181
479.3041	48.1120
509.0400	6.5150
537.9519	43.3336
564.8795	216.2248
571.0332	65.2049
596.9609	54.6642
600.8923	32.3528
613.4545	50.1654

Frequency (cm^{-1})	IR Intensity (KM/Mole)
634.0354	19.6926
634.4544	0.6355
639.2692	42.1559
671.1323	105.6702
692.0663	26.3182
711.9892	66.7680
739.8632	6.9243
763.5008	13.4483
770.8703	76.1331
772.3406	1.1361
786.4287	2.8060
796.7445	5.3862
815.2974	13.5720
837.2982	12.0781
858.5221	0.2418
865.5086	10.7952
882.6875	24.7593
888.8415	14.9075
927.4336	8.8333

Frequency (cm^{-1})	IR Intensity (KM/Mole)
934.8583	5.3107
943.3366	8.0357
952.8606	11.8913
954.3810	216.1552
964.0649	0.3663
966.1883	2.9053
990.1118	0.4634
995.1148	2.9938
997.9774	0.3839
1007.3150	0.1162
1010.7918	133.0116
1015.9445	0.0486
1035.2878	33.0046
1048.0583	11.3431
1050.8931	14.7489
1067.6799	31.5405
1085.3371	6.3353
1107.2824	45.7472
1111.9799	4.9974

Frequency (cm^{-1})	IR Intensity (KM/Mole)
1118.9970	5.5090
1161.7859	12.7857
1176.9389	1.1317
1185.3204	17.4577
1186.5490	92.1380
1197.6313	2.1855
1198.5352	0.5394
1205.2352	44.4438
1214.1186	7.9473
1220.1271	1.0256
1226.6538	13.9915
1268.6496	36.3501
1294.1967	60.3751
1304.3599	126.4726
1316.4474	32.6729
1320.6656	15.0112
1337.8199	18.2162
1338.5356	14.4644
1353.6123	37.5266

Frequency (cm^{-1})	IR Intensity (KM/Mole)
1357.3503	158.3011
1365.9294	85.4329
1376.8550	271.2302
1380.4717	13.4071
1395.4000	63.6091
1399.9458	73.4315
1409.9374	186.0007
1415.1487	97.9383
1420.4602	18.8415
1440.4542	92.8850
1477.2862	9.9740
1478.8591	4.0781
1482.2950	5.4280
1485.3874	8.9739
1490.0569	10.1475
1492.4672	3.3812
1494.8625	37.2040
1499.8900	11.7012
1506.3825	59.7955

Frequency (cm^{-1})	IR Intensity (KM/Mole)
1509.4036	14.1421
1523.7724	18.2454
1575.6022	4.5122
1613.5634	6.0766
1619.6603	1.5695
1639.8132	11.3636
1690.6116	769.1961
1702.7328	635.0534
3008.5900	5.1882
3009.4246	14.9820
3012.3952	75.5680
3019.6027	34.3247
3041.9723	21.0827
3048.9429	44.7981
3050.2316	23.8202
3070.6590	18.9431
3077.9926	83.2097
3083.1572	56.6912
3087.0623	54.4439

Frequency (cm^{-1})	IR Intensity (KM/Mole)
3092.4600	4.0663
3112.2735	14.9762
3147.7096	14.5665
3162.5282	0.4591
3163.8880	1.0935
3167.6435	19.6085
3172.1850	27.5735
3179.9467	30.5612
3183.7923	27.4299
3191.9458	8.2758
3565.1982	44.3420
3579.1283	60.5982
3837.0125	18.2815
3843.8377	163.6548

Bortezomib in methanol

Frequency (cm^{-1})	IR Intensity (KM/Mole)
16.7453	0.8289
21.0563	1.8617

Frequency (cm^{-1})	IR Intensity (KM/Mole)
22.2258	0.3704
28.8204	0.6232
33.2474	3.2832
40.2391	0.0553
44.7182	0.4632
48.1614	0.2821
55.2904	1.0052
74.6769	0.6254
76.2820	0.4166
93.9343	3.6309
106.7584	1.0262
112.8022	4.6764
156.3945	0.5721
160.0103	5.0062
188.7368	0.3427
194.9398	0.3341
201.8880	0.8060
226.8633	0.1634
242.8978	0.8953

Frequency (cm^{-1})	IR Intensity (KM/Mole)
253.3517	0.6928
261.0112	3.1349
288.2917	6.0292
326.4842	0.9462
340.8182	16.8744
346.1048	9.5282
355.4246	0.7369
372.8362	18.3890
388.7347	2.6123
412.4998	34.7817
415.1540	0.1236
422.1930	1.7060
449.7627	59.9494
459.5442	10.3529
480.2413	69.8746
494.7012	2.1554
517.0748	4.3856
539.7176	42.7647
564.0126	267.9857

Frequency (cm^{-1})	IR Intensity (KM/Mole)
572.1293	68.1852
598.7174	84.2962
603.9607	10.1412
610.7956	92.7045
630.5960	38.4569
633.8302	3.2307
634.3938	7.5777
669.7268	141.0898
692.4143	28.3788
711.9817	77.4823
739.7587	4.0406
762.7846	18.0002
768.0634	58.7508
772.1477	23.5815
785.3986	2.9418
796.1436	6.4651
815.8467	12.3738
837.6547	12.8558
858.9593	0.0671

Frequency (cm^{-1})	IR Intensity (KM/Mole)
867.3432	9.3412
883.4796	32.9130
888.1164	15.7954
926.7232	9.5021
935.9593	5.6711
942.9950	5.8081
953.0832	6.8980
962.0170	265.2297
963.9599	2.9852
967.1655	5.8538
991.8526	0.2501
995.6571	0.3857
1001.7067	0.3447
1008.9214	0.0577
1015.2425	0.1176
1030.5579	174.4953
1035.0292	34.6622
1047.5889	12.2492
1051.3461	18.3841

Frequency (cm^{-1})	IR Intensity (KM/Mole)
1068.0372	36.0232
1085.7314	6.4985
1108.2145	59.3722
1110.9204	6.0515
1116.1632	7.9344
1162.4815	12.4697
1175.4167	0.3405
1185.0442	3.1925
1188.5758	95.5017
1196.8585	6.5350
1198.3157	5.5177
1207.3394	49.8760
1211.0119	12.9380
1220.4649	1.8826
1226.5470	10.3017
1268.4884	29.4011
1296.3057	73.3121
1309.1261	146.6058
1317.4694	20.0841

Frequency (cm^{-1})	IR Intensity (KM/Mole)
1322.3101	25.9477
1337.1090	21.4017
1338.4363	17.0849
1354.8212	26.7665
1357.3379	238.9902
1365.9018	200.3331
1374.5612	235.2628
1379.4756	21.8605
1394.9306	21.6149
1398.5141	90.2019
1412.3646	196.7506
1417.7452	98.7803
1418.9747	30.7982
1440.5660	118.0225
1475.0252	13.3722
1476.8597	5.6655
1481.6556	11.0761
1481.7933	4.3355
1487.8794	12.0052

Frequency (cm⁻¹)	IR Intensity (KM/Mole)
1490.4800	4.5452
1494.1289	42.2881
1497.6678	20.2035
1505.0829	16.7478
1509.3437	74.9395
1523.3864	19.4768
1574.7699	5.1899
1613.1050	11.8676
1618.4971	1.1619
1638.6470	15.7436
1674.6539	953.5232
1689.6752	758.9016
3005.3754	8.7646
3009.7603	20.6937
3013.0810	94.4825
3019.6205	39.1769
3046.5743	33.2445
3050.6215	43.7455
3053.9005	29.6185

Frequency (cm^{-1})	IR Intensity (KM/Mole)
3070.1437	30.8769
3077.1331	99.2731
3081.7114	70.2529
3086.6915	61.5537
3097.3014	4.1164
3117.1495	20.2660
3152.6238	13.0995
3163.2340	0.2455
3168.0294	1.0487
3170.9410	21.0855
3172.6776	29.0367
3182.7555	36.3220
3183.2106	30.3547
3189.7894	13.7256
3566.4815	63.4717
3576.2083	67.3617
3830.5770	83.6111
3835.9808	128.7150

Bortezomib in water

Frequency (cm⁻¹)	IR Intensity (KM/Mole)
16.7432	0.7989
20.2383	1.4577
21.9979	0.6051
27.0227	0.8763
33.2821	3.3715
39.8075	0.0383
44.4094	0.4136
47.9667	0.3236
55.6793	0.9762
74.0300	0.6544
75.7200	0.4710
93.7016	3.6500
105.9470	1.0736
111.8737	4.6815
156.0986	0.6012
159.8429	5.0316
188.2030	0.3627
194.8653	0.3607

Frequency (cm^{-1})	IR Intensity (KM/Mole)
201.5233	0.8411
226.7619	0.1647
242.9964	0.9440
252.9225	0.6492
260.5779	3.3001
288.2847	6.1119
326.3513	0.9917
340.9113	16.6994
345.9936	9.9727
355.3013	0.7644
372.4234	19.0481
388.5642	2.5084
412.2499	35.2981
414.7331	0.1337
422.0613	1.8105
449.7036	61.0868
459.4868	10.6497
480.2099	73.3872
492.5321	1.5110

Frequency (cm^{-1})	IR Intensity (KM/Mole)
517.0830	4.4219
539.5072	42.5392
563.7291	268.3714
571.9978	70.4948
598.0940	88.0506
603.9769	9.4632
610.6392	97.4673
630.1133	38.6083
633.7283	3.7690
634.3564	7.7262
669.1559	143.5280
692.4124	28.3961
711.6472	79.1665
739.7260	3.7837
762.5114	18.3913
767.7189	57.9150
771.9946	25.1814
785.4024	3.2259
796.0518	6.5695

Frequency (cm^{-1})	IR Intensity (KM/Mole)
815.8285	12.5315
837.6771	12.9939
858.6831	0.0406
867.3985	9.0538
883.5099	34.0316
888.0330	15.9793
926.6308	9.6010
935.7421	5.5457
942.9099	5.8557
953.0410	7.1106
962.2652	266.5514
963.8630	6.5005
967.4792	5.8168
991.8180	0.2008
995.7202	0.4082
1001.9266	0.3559
1009.1311	0.0539
1015.1630	0.1182
1031.5011	177.5631

Frequency (cm^{-1})	IR Intensity (KM/Mole)
1035.0394	34.4821
1047.4511	12.2780
1051.6069	19.3666
1068.0748	36.6572
1085.8440	6.7960
1108.3280	61.2854
1110.8338	6.1327
1115.8036	8.2048
1162.4534	12.8589
1175.2860	0.3313
1184.9411	3.3675
1188.5950	95.1021
1196.8284	6.5131
1198.2350	6.2400
1207.4739	50.6684
1210.7066	13.6315
1220.4330	1.9434
1226.6124	10.0165
1268.3653	29.2100

Frequency (cm⁻¹)	IR Intensity (KM/Mole)
1296.3113	74.2273
1309.3518	147.8794
1317.7028	19.9783
1322.9202	26.1686
1337.0311	21.3308
1338.2971	17.8676
1354.8513	32.4319
1357.1662	250.4244
1365.8399	227.6076
1374.6337	207.8188
1379.3509	24.2931
1394.8473	19.3530
1398.3721	93.1893
1412.5734	196.5268
1417.7136	77.8116
1419.2838	51.7636
1440.7565	120.6536
1474.5548	13.7449
1476.3528	6.4243

Frequency (cm^{-1})	IR Intensity (KM/Mole)
1481.1064	9.0921
1481.6454	6.2519
1487.5379	11.6711
1490.1619	4.4512
1494.0558	44.6637
1497.3491	21.0502
1504.6102	16.9438
1509.6451	75.9489
1523.3853	19.4687
1574.5710	5.2893
1613.1170	12.5760
1618.3925	1.0918
1638.5340	16.3798
1672.9874	975.2010
1688.0370	774.7750
3004.8444	10.2038
3009.8067	22.1085
3013.1941	95.3715
3019.6126	39.5965

Frequency (cm^{-1})	IR Intensity (KM/Mole)
3047.2651	35.3767
3050.9100	42.9414
3054.0130	30.0895
3070.0953	32.4509
3077.0387	101.0337
3081.5396	71.8619
3086.3732	62.9040
3097.1612	4.1648
3117.3111	20.8961
3153.1888	12.9572
3163.2821	0.2080
3168.5101	0.9993
3171.3849	21.2828
3172.7113	28.9318
3182.4952	37.5114
3183.6149	30.8244
3189.5803	14.4008
3566.1478	65.7955
3576.1076	67.4086

Frequency (cm^{-1})	IR Intensity (KM/Mole)
3829.4188	82.7242
3835.1449	132.2840

Bortezomib (de1) in gas phase

Frequency (cm^{-1})	IR Intensity (KM/Mole)
18.5387	0.8779
24.4310	0.5768
33.2727	0.1666
37.9555	0.0903
49.5604	0.1797
50.0562	2.3342
60.1459	0.7947
61.0806	0.7100
72.1705	0.1308
86.5678	2.9214
99.6134	1.0553
106.4582	0.6862
116.1423	0.3109
132.5223	17.6949

Frequency (cm^{-1})	IR Intensity (KM/Mole)
145.8318	3.1749
171.6746	10.5172
180.9323	1.3561
201.5320	3.8074
209.4100	0.0873
228.1691	1.7296
239.7057	4.6158
251.4267	7.0217
265.2501	1.2784
300.3908	3.9386
319.2724	8.0502
332.7482	10.1188
350.5319	16.4897
372.2182	3.3724
380.0865	12.4734
389.6856	1.8891
415.9346	0.2504
422.1810	0.0499
428.0642	11.9783

Frequency (cm^{-1})	IR Intensity (KM/Mole)
447.0954	28.7438
473.4970	7.0098
488.3698	4.6959
519.1517	11.6329
524.0531	45.5869
553.6962	2.3926
571.5659	85.1707
586.8085	25.3112
614.8248	3.5335
632.8638	2.8493
635.3815	0.9008
654.6165	32.3082
666.5120	11.5249
696.6623	50.8086
712.5691	52.1872
742.0359	10.3698
752.6662	4.6735
755.4633	8.4951
757.8007	59.4364

Frequency (cm^{-1})	IR Intensity (KM/Mole)
792.5264	13.0998
793.6347	23.0239
806.7721	32.6694
832.4689	49.4517
839.8078	43.3179
858.1205	1.4898
866.5792	4.0781
885.1824	15.4895
893.4152	21.1117
931.4484	4.9991
932.1519	5.4798
941.9596	6.9965
959.4073	19.8158
963.1444	3.9027
964.7781	0.1607
986.1191	0.4617
988.1196	187.2231
997.2412	64.7695
1003.7904	0.5594

Frequency (cm⁻¹)	IR Intensity (KM/Mole)
1014.9500	18.6336
1017.4170	0.2134
1020.7678	6.6632
1039.7416	17.2011
1051.0321	6.7010
1071.7193	40.3054
1092.8021	29.1848
1100.4213	9.1931
1105.2004	3.6581
1120.5978	3.3878
1127.6919	46.1642
1165.5509	14.7085
1177.6584	0.0454
1187.2666	12.5594
1189.0655	78.8196
1200.3177	21.6245
1201.4548	0.8216
1204.1434	0.5728
1221.4981	21.5858

Frequency (cm^{-1})	IR Intensity (KM/Mole)
1226.4077	3.3171
1229.6724	4.8181
1273.6338	0.9874
1301.9823	88.6318
1315.3401	1.1341
1319.5800	12.3192
1334.7621	0.9781
1338.4263	2.7003
1344.1037	15.2560
1348.2832	74.0652
1365.8346	3.2803
1375.5896	2.0535
1385.7080	19.4369
1394.2930	6.9162
1400.5721	101.5886
1419.2273	87.8102
1420.5243	14.3971
1448.5434	138.1113
1455.2127	23.0607

Frequency (cm⁻¹)	IR Intensity (KM/Mole)
1475.0381	0.3354
1479.8553	22.2266
1481.8107	4.2963
1491.7031	14.0054
1492.3903	4.4309
1501.6013	7.7677
1503.6418	3.8195
1506.9348	18.5319
1518.1807	13.5335
1525.2156	11.9602
1531.9003	299.8195
1574.5440	5.4167
1612.4405	9.2643
1620.4474	2.4677
1642.8473	11.8847
1687.2439	577.5749
1690.9109	376.2642
2962.1856	64.2608
2996.2084	27.3149

Frequency (cm^{-1})	IR Intensity (KM/Mole)
3002.1533	37.2779
3004.2379	57.3021
3016.6015	40.9216
3025.8548	22.5479
3031.9118	30.2530
3049.4876	3.8476
3059.6644	99.0572
3074.3086	49.9411
3076.0061	3.4249
3092.9410	14.1459
3097.2841	6.5628
3120.9377	76.8295
3143.8182	6.8232
3154.7968	3.1943
3154.9941	28.9630
3165.2000	30.2908
3176.6823	20.9302
3181.5087	5.8086
3184.7219	13.3525

Frequency (cm⁻¹)	IR Intensity (KM/Mole)
3436.0324	152.8367
3554.2980	12.6732
3826.7045	9.6482

Bortezomib (de1) in diethylether

Frequency (cm⁻¹)	IR Intensity (KM/Mole)
28.5738	0.5124
33.7811	1.5702
38.8858	1.1043
51.4658	2.4349
57.3515	0.4404
60.2062	1.3468
67.0205	2.0202
74.1296	1.0610
89.1446	0.8698
91.7660	3.6253
97.7322	4.1601
104.6933	2.5698
114.6928	0.6748

Frequency (cm^{-1})	IR Intensity (KM/Mole)
127.6353	16.1370
141.1061	1.4019
171.0364	21.1553
182.8894	0.5640
202.6677	3.0330
208.1570	0.1315
234.2234	11.7931
237.9184	0.8996
252.2485	8.2728
291.0058	1.8459
298.2085	5.1506
319.8018	16.2249
335.3324	14.1129
349.0192	18.5887
365.9455	7.5368
383.5886	16.0797
399.3841	1.1167
418.0143	0.2178
422.9698	6.2778

Frequency (cm^{-1})	IR Intensity (KM/Mole)
425.2349	14.5178
442.2023	37.6080
466.5998	42.3285
486.9102	0.9610
518.3252	20.3034
530.0817	66.1950
549.2600	102.8221
561.4317	14.6818
585.7400	38.6279
614.7351	1.6954
634.4014	3.4147
634.6632	2.0654
652.0065	48.6655
667.2861	4.7077
695.4695	95.6278
714.4604	67.6195
738.3917	44.2037
750.0848	20.7781
753.1983	55.1451

Frequency (cm^{-1})	IR Intensity (KM/Mole)
758.5281	21.9811
777.0962	87.0505
790.1976	15.5312
797.7060	4.9108
828.3445	42.7783
838.6514	30.2747
861.4205	1.2842
865.6727	2.0155
876.5642	24.4778
891.9999	28.6379
933.9028	10.5808
936.0561	9.7989
943.7654	4.8477
958.6449	56.7117
964.7282	4.4626
966.0193	1.1992
978.1894	261.3200
993.2698	0.0681
997.0775	23.5115

Frequency (cm^{-1})	IR Intensity (KM/Mole)
998.8946	23.9746
1008.1212	1.1077
1015.4964	2.6113
1019.5902	13.2521
1038.0717	28.8893
1049.7346	9.5983
1071.4813	40.3000
1092.5890	49.5305
1100.1786	16.1361
1106.6630	7.1044
1117.5859	45.6637
1121.8095	36.8402
1164.5279	16.9749
1176.6731	0.0074
1187.1096	15.9902
1191.9416	96.3603
1200.8292	1.6267
1201.2452	0.3206
1204.9280	23.8067

Frequency (cm^{-1})	IR Intensity (KM/Mole)
1222.7704	24.0393
1225.6647	16.1296
1229.0281	6.7825
1274.3491	1.1544
1306.0263	143.3382
1318.1080	12.6106
1325.2410	3.9797
1326.0748	12.5113
1342.1208	2.8212
1344.1655	7.2067
1350.1281	66.3045
1367.3090	5.0128
1374.7306	3.6016
1390.3947	23.8502
1397.5499	5.1460
1407.2366	147.4277
1419.4922	17.9857
1421.3076	146.8441
1451.7047	145.3398

Frequency (cm⁻¹)	IR Intensity (KM/Mole)
1456.2350	79.1008
1474.5898	1.1570
1481.5816	6.3812
1483.1530	33.4679
1489.6776	25.6179
1492.1315	6.6078
1498.8229	130.8063
1502.2026	154.9064
1504.8854	28.6580
1512.4633	184.2792
1520.5629	14.1043
1524.6462	13.6581
1573.9295	11.6889
1615.0418	17.3549
1619.5120	3.6442
1640.3218	18.8213
1665.3465	761.6352
1671.9071	692.8958
2982.4009	69.3370

Frequency (cm^{-1})	IR Intensity (KM/Mole)
3001.8116	27.7120
3003.8798	12.2187
3008.9956	79.3990
3029.1215	54.0873
3032.0876	35.2537
3032.3260	60.0347
3055.7435	0.6661
3063.6349	136.8589
3082.8153	4.2971
3084.8024	51.8030
3098.9732	24.1760
3099.7728	11.1265
3151.4326	9.3107
3160.8387	0.8483
3166.5294	18.1137
3169.5344	20.0513
3177.4516	26.2745
3181.9333	15.0542
3185.0672	10.4083

Frequency (cm^{-1})	IR Intensity (KM/Mole)
3186.4365	21.1557
3466.2822	186.9632
3552.1992	34.5504
3826.7598	21.2615

Bortezomib (de1) in methanol

Frequency (cm^{-1})	IR Intensity (KM/Mole)
16.6564	0.4064
21.0013	3.1637
23.3650	1.7792
30.2511	0.2195
32.1535	0.9042
38.0479	0.2947
42.3561	0.1734
53.7282	1.0332
55.3488	0.9233
75.6874	2.3814
85.5575	1.5490
97.6647	3.3185

Frequency (cm⁻¹)	IR Intensity (KM/Mole)
105.9208	2.3093
112.8165	12.1405
155.7409	11.6741
167.5322	1.2174
183.0601	3.1276
185.1704	1.6759
210.5041	3.2133
229.2647	6.6100
247.5047	4.7029
249.9824	19.9304
253.8591	0.8537
289.1779	11.6313
327.2185	6.5019
338.3989	33.8349
347.9873	1.6697
368.0049	5.7098
370.5212	23.7600
390.8152	3.5445
408.8849	11.6049

Frequency (cm^{-1})	IR Intensity (KM/Mole)
415.3908	0.0358
419.1922	0.3941
436.0901	26.8055
464.0072	54.7140
475.3857	37.4764
518.1310	1.2718
537.4097	110.8687
542.2637	99.1084
568.3887	52.6797
588.2142	24.6469
623.0671	9.1235
629.8310	8.4554
634.0389	0.5490
646.2556	99.8770
655.3586	29.5633
701.2147	89.7146
713.6678	67.3380
725.6006	75.8361
741.5631	48.8990

Frequency (cm^{-1})	IR Intensity (KM/Mole)
745.7809	15.4997
765.0843	40.6675
776.3730	54.8042
785.2759	5.8803
792.1729	27.4045
816.4696	12.9611
838.0576	41.2270
850.2743	17.3378
861.7854	0.3424
879.9269	19.5133
888.8753	35.2842
925.5728	10.5238
933.7881	7.3320
943.0337	9.5745
963.1358	3.1657
967.2894	3.5390
971.7016	201.9878
983.5455	131.0016
994.3529	1.0349

Frequency (cm^{-1})	IR Intensity (KM/Mole)
999.3194	17.2076
1000.7049	0.2663
1010.4557	0.0557
1015.0043	0.0934
1036.9547	42.6794
1046.8250	6.9433
1051.0128	33.9812
1070.5305	18.8657
1093.2717	11.4306
1102.0051	0.6225
1107.1028	52.9941
1112.7903	116.1249
1118.1079	24.2411
1161.7772	24.0433
1173.9368	0.1425
1185.4863	14.8434
1186.5893	111.4309
1191.9761	3.6856
1195.4823	0.6064

Frequency (cm^{-1})	IR Intensity (KM/Mole)
1199.6183	3.8889
1221.6141	13.3760
1222.8593	30.1408
1223.2870	9.9064
1271.8439	2.8217
1291.0493	52.6726
1311.5277	26.7438
1316.0023	0.5621
1320.4044	8.2710
1337.5359	6.8141
1340.4072	6.6517
1356.8698	14.4704
1369.1764	3.7826
1371.1472	11.0253
1389.4916	90.7997
1390.1802	284.2635
1394.0923	9.7550
1414.4746	18.9206
1423.2592	170.2548

Frequency (cm^{-1})	IR Intensity (KM/Mole)
1442.5689	120.0478
1456.1193	91.9784
1472.7222	10.0654
1476.7280	49.4890
1479.8507	0.7738
1482.8559	131.6255
1484.3648	292.3155
1487.7207	0.8847
1490.5222	198.5714
1493.6049	14.2531
1501.0404	64.4586
1505.8154	19.5582
1522.7437	20.5257
1572.6150	9.4452
1610.8136	47.8317
1617.8266	3.1657
1638.5365	29.4973
1649.8496	1029.5212
1662.4723	929.9081

Frequency (cm^{-1})	IR Intensity (KM/Mole)
2990.7492	61.6893
2999.2195	8.1104
3004.8146	40.5084
3011.0943	88.2994
3018.7014	71.5806
3035.7281	73.6490
3055.4773	29.3388
3060.2993	0.9562
3066.5024	159.2465
3082.8760	54.3644
3098.9430	25.9420
3101.4250	11.9139
3157.3469	5.1902
3162.8545	0.3789
3168.1627	1.0550
3168.3821	5.6017
3171.5804	21.3423
3178.3552	44.5018
3182.3760	42.8302

Frequency (cm⁻¹)	IR Intensity (KM/Mole)
3187.4551	20.8379
3191.5391	0.9591
3470.2831	224.7240
3555.6719	85.3744
3817.7189	26.3912

Bortezomib (de1) in water

Frequency (cm^{-1})	IR Intensity (KM/Mole)
18.0769	0.1140
21.3617	3.0689
24.6438	2.0314
29.2037	0.2568
34.6859	0.6524
38.8717	0.6044
41.7819	0.1361
53.2256	1.3241
55.1014	0.7121
74.2742	2.7982
85.3474	1.5249

Frequency (cm⁻¹)	IR Intensity (KM/Mole)
97.4189	3.5299
104.2685	2.8842
109.7811	11.7444
155.3485	11.3599
167.3930	1.1309
182.8567	3.0047
185.0337	1.8061
210.1457	3.3268
229.9499	6.6497
247.2567	4.6851
250.1189	21.3293
256.0681	0.0654
288.9000	11.9251
327.0799	6.0197
338.4519	34.8604
348.0724	1.6209
367.8624	5.7795
370.3110	24.3416
389.8094	3.6346

Frequency (cm^{-1})	IR Intensity (KM/Mole)
408.8479	11.9712
415.0244	0.0426
419.3692	0.3880
435.9962	27.0997
462.9335	56.6550
475.4726	39.2213
517.9374	1.1639
536.9384	114.7455
541.4209	99.1506
567.9393	53.4484
588.1975	25.2770
622.4783	10.3304
629.7718	8.5755
633.9841	0.6034
645.4624	104.9800
655.3510	27.6118
700.7201	97.1521
713.3623	67.6167
724.5113	76.7598

Frequency (cm^{-1})	IR Intensity (KM/Mole)
741.4137	48.9701
745.5273	14.9774
764.9586	43.7360
775.6428	54.2801
785.1938	5.4723
792.0424	26.8721
816.3296	12.8698
837.9093	41.4625
850.1004	17.6750
861.5583	0.2238
880.1735	19.8372
888.7203	35.3717
924.8291	10.5461
933.6381	7.4054
943.0087	9.6952
963.0294	3.1285
966.8907	3.4191
971.5255	207.0024
983.4370	131.5027

Frequency (cm^{-1})	IR Intensity (KM/Mole)
994.3546	0.8587
999.2832	17.5680
1001.2040	0.1964
1010.7937	0.0569
1014.9530	0.1021
1036.8751	43.1546
1046.7510	7.2263
1051.0152	34.5118
1070.4548	19.1005
1093.2842	11.9259
1102.1080	0.7192
1107.0869	55.1626
1112.5037	118.9383
1117.8770	25.2914
1161.6318	24.4270
1173.7779	0.1237
1185.3424	14.1990
1186.6268	114.1261
1192.1062	3.8856

Frequency (cm^{-1})	IR Intensity (KM/Mole)
1195.4120	0.6720
1199.5249	4.2042
1221.5820	13.1009
1222.9238	28.3530
1223.1817	13.6340
1271.5956	2.8548
1291.3935	54.4230
1311.5162	27.1092
1315.9841	0.5581
1320.3406	8.6670
1337.3607	6.9781
1340.0856	6.5084
1356.8771	14.5734
1369.1449	2.7426
1371.2674	12.3532
1389.1722	82.3390
1389.9878	300.5455
1393.8177	10.6869
1413.9156	19.3222

Frequency (cm⁻¹)	IR Intensity (KM/Mole)
1423.3838	178.4844
1442.5875	122.8779
1456.0049	93.5378
1472.2144	15.7410
1476.2262	57.8119
1479.7837	0.8932
1481.7695	385.2473
1483.1584	82.9619
1487.3142	1.5144
1490.2574	159.2883
1492.9599	8.7776
1500.9645	66.2897
1505.7827	20.6236
1522.7207	20.7631
1572.5697	9.6320
1610.8497	50.9399
1617.7494	3.2314
1638.3921	31.0284
1648.2420	1043.3989

Frequency (cm^{-1})	IR Intensity (KM/Mole)
1661.2255	944.7679
2991.2649	62.4006
2998.5828	9.8049
3005.1034	41.5731
3011.2365	87.3952
3019.6545	72.9113
3035.5407	75.7509
3055.7558	29.6239
3060.7467	1.0303
3066.7607	162.6880
3082.7641	54.5809
3098.0313	27.9446
3101.7402	12.0737
3157.7284	5.2864
3163.0244	0.3261
3168.0414	6.0816
3168.8978	0.5881
3171.8230	21.1651
3178.5287	46.1709

Frequency (cm⁻¹)	IR Intensity (KM/Mole)
3182.8932	43.6158
3187.6553	20.7963
3191.1629	0.7902
3472.5973	222.3179
3555.2822	86.7104
3817.4048	26.9053

Bortezomib (de2) in gas phase

Frequency (cm^{-1})	IR Intensity (KM/Mole)
7.8219	1.5418
14.9456	0.2100
22.3300	0.5607
25.2356	1.0756
28.9316	0.3040
36.3053	1.5560
45.2771	1.1192
49.9900	0.4601
54.6184	0.3490
67.2265	3.9139

Frequency (cm^{-1})	IR Intensity (KM/Mole)
72.5752	0.6771
101.0925	1.3476
111.5239	3.4289
122.3157	7.6141
162.3650	1.6942
167.6175	6.0956
179.6803	1.1403
197.3155	0.2364
206.5139	2.9137
222.2317	0.8075
238.2326	1.9808
256.1952	2.5368
262.2310	1.8290
287.2891	5.8455
321.1743	0.7424
336.9001	9.9987
348.7566	5.6075
355.4422	3.6547
381.3711	7.3628

Frequency (cm^{-1})	IR Intensity (KM/Mole)
391.7268	7.0980
415.5381	18.2779
415.9682	2.5868
424.5398	0.0954
442.6539	45.5846
459.7282	0.8981
473.7533	14.3616
506.0361	10.5855
542.3273	24.9730
568.1786	103.8638
573.8315	12.4922
583.6447	62.6521
609.7815	16.5769
615.0471	5.8440
633.1388	1.7793
635.6072	2.4714
639.8714	5.6460
691.1483	37.0089
711.9140	49.4658

Frequency (cm⁻¹)	IR Intensity (KM/Mole)
729.8180	4.8048
748.0962	10.9751
749.7535	39.1607
765.9101	20.8163
770.3382	49.9476
787.0343	6.3404
800.5375	2.6357
813.7900	25.4543
825.3758	3.3111
858.0439	1.6877
862.0869	11.0043
881.8317	13.4175
892.2087	20.4998
918.7865	69.5449
930.4354	5.1774
933.8273	4.4462
950.5917	2.9680
957.5707	3.8194
963.2214	42.2430

Frequency (cm^{-1})	IR Intensity (KM/Mole)
967.7288	123.7049
984.3392	0.3441
986.9718	14.6300
988.7882	0.3217
1002.7335	2.1979
1017.5221	0.2308
1035.4564	22.6750
1047.4800	4.2838
1050.8475	9.9800
1066.5384	30.9585
1082.8534	4.0266
1098.1811	26.5366
1103.6288	22.0461
1106.5577	24.9195
1112.6852	1.0857
1161.0929	6.7631
1177.4852	0.5064
1184.5843	95.6139
1186.4133	3.9361

Frequency (cm^{-1})	IR Intensity (KM/Mole)
1198.0088	0.9698
1202.7888	8.9977
1205.1629	21.8814
1208.2513	1.4514
1220.9044	2.0836
1221.7217	0.7349
1270.2579	1.5239
1306.4637	17.2236
1311.5245	47.8517
1314.3746	19.1957
1316.5615	11.9242
1338.2171	2.9994
1341.9286	10.0226
1354.2272	96.7291
1364.7105	2.4195
1377.4564	2.7474
1384.2557	4.4143
1394.6522	5.9466
1408.5675	87.0120

Frequency (cm^{-1})	IR Intensity (KM/Mole)
1418.8374	64.2884
1421.1530	2.5411
1442.3207	60.7903
1455.0488	29.6679
1476.5421	9.8961
1480.0400	12.8790
1480.7527	5.9460
1487.4840	37.1420
1489.5749	2.6248
1492.2490	4.9884
1501.1730	6.8399
1502.7945	29.1942
1513.6899	15.1038
1524.1022	18.8084
1573.4600	458.8500
1578.3258	4.1312
1612.0497	1.9196
1619.3759	2.4856
1641.7898	12.9572

Frequency (cm^{-1})	IR Intensity (KM/Mole)
1691.1526	541.2225
1706.1195	559.4804
2949.6138	85.2212
2994.8974	13.8712
2998.2492	32.2569
3005.3683	81.5320
3029.0630	24.7571
3039.1969	29.7399
3039.9283	14.0818
3055.3510	22.0168
3064.3454	59.3885
3067.1203	71.3107
3091.6014	1.9998
3107.6764	9.9104
3110.2726	7.3618
3143.1811	6.7755
3147.0239	7.9799
3153.8222	3.6129
3160.6596	25.0206

Frequency (cm⁻¹)	IR Intensity (KM/Mole)
3164.8465	33.6186
3167.9326	27.6068
3177.3662	26.2866
3189.6846	9.4198
3539.4543	110.1495
3576.3448	21.7153
3835.2254	12.9567

Bortezomib (de2) in diethylether

Frequency (cm^{-1})	IR Intensity (KM/Mole)
17.0848	2.1249
23.1193	0.2719
28.3586	0.5637
30.4599	0.8078
33.1703	0.3827
41.7066	0.7607
46.2289	0.8916
50.5946	1.7647
56.9655	0.7025

Frequency (cm⁻¹)	IR Intensity (KM/Mole)
76.1381	0.9688
85.4345	5.8827
107.7670	4.1640
112.7158	3.3897
122.9677	11.0737
161.8109	6.0411
166.9504	2.9131
188.2612	0.7095
193.9095	0.7213
207.2824	1.6403
226.6280	0.5043
243.0530	3.1190
245.5145	1.2003
257.9747	3.1572
285.3821	8.0506
329.5090	3.7951
341.3322	14.9623
349.1810	7.4829
357.6526	8.0762

Frequency (cm^{-1})	IR Intensity (KM/Mole)
377.2817	12.7364
397.8962	4.2726
414.9140	31.6192
416.1035	0.1498
430.9802	1.0619
449.9679	43.9021
463.3440	17.6762
481.9525	26.6103
506.6517	12.9857
542.4177	56.3319
563.3973	132.6482
574.0696	35.7307
599.1661	78.6019
607.9503	20.7272
618.0697	13.3625
632.7141	4.5971
634.4821	0.7992
637.6635	7.9937
690.8020	82.9519

Frequency (cm^{-1})	IR Intensity (KM/Mole)
711.6829	58.1938
715.4698	74.6795
737.4001	19.3542
749.2803	0.9076
767.9215	43.3383
769.7793	38.9319
785.7255	2.2319
796.3843	3.4575
814.6622	18.2171
829.3248	9.0784
858.5566	0.7161
865.0893	12.2313
881.8890	24.6503
892.3630	25.7264
920.8432	76.9741
933.2807	8.3148
934.7007	30.1125
952.1604	9.6268
964.1046	18.7350

Frequency (cm^{-1})	IR Intensity (KM/Mole)
964.6835	1.9581
970.0324	188.7316
989.7423	0.5221
991.7156	16.9249
996.3298	0.4497
1005.1464	0.4447
1016.1360	0.0914
1035.1047	31.7656
1048.0055	9.6657
1049.0011	11.8714
1067.4317	38.8531
1084.5318	5.0597
1101.0598	37.1056
1106.0223	39.4337
1109.4649	15.5956
1112.8319	37.9751
1165.3686	19.9180
1175.8360	0.4198
1186.3887	15.6442

Frequency (cm^{-1})	IR Intensity (KM/Mole)
1187.4408	83.4028
1196.9678	1.8695
1200.1382	3.1281
1205.4422	52.3150
1213.6692	7.1074
1216.3331	3.7911
1221.0168	0.6427
1270.5764	4.9737
1308.9595	54.8973
1316.5818	45.5273
1316.7641	37.4932
1319.2899	10.0294
1339.0658	19.8745
1340.7947	1.1145
1356.0671	109.7786
1365.7685	16.9585
1373.7169	4.5981
1388.4154	6.6950
1395.5983	5.3515

Frequency (cm^{-1})	IR Intensity (KM/Mole)
1413.2232	135.6931
1417.8319	14.9032
1421.2709	90.7963
1441.3197	85.3133
1462.8528	73.5563
1478.7668	11.5690
1478.9799	13.7252
1481.7916	9.0404
1484.9928	41.8648
1491.1611	39.9216
1493.5991	2.8659
1496.8731	12.7590
1507.2579	43.9491
1508.1187	62.4435
1523.5687	275.6796
1524.3399	352.7100
1575.7008	4.5196
1613.8609	8.4093
1618.7259	2.4282

Frequency (cm⁻¹)	IR Intensity (KM/Mole)
1639.8947	15.5887
1677.9943	777.0846
1683.5084	766.2463
2979.1467	83.0867
3001.3226	7.9433
3004.2299	47.3751
3013.4852	66.9207
3037.0693	47.8732
3041.9920	46.5892
3049.3182	31.5400
3060.8096	8.4685
3067.6790	119.9106
3073.0877	70.2167
3101.8259	0.4904
3111.1566	17.0562
3115.9361	16.4419
3149.8816	10.9123
3160.5295	1.2741
3161.4823	2.2387

Frequency (cm^{-1})	IR Intensity (KM/Mole)
3164.7545	23.2273
3170.4449	31.9266
3177.7429	34.4617
3182.1323	27.9055
3190.5038	9.3055
3560.4931	112.9350
3570.6809	45.9647
3831.4678	25.4235

Bortezomib (de2) in methanol

Frequency (cm^{-1})	IR Intensity (KM/Mole)
18.4625	1.9522
20.0891	1.1469
24.3640	1.6252
25.0131	1.4565
34.7049	0.7384
41.7424	0.2634
45.4062	1.9476
48.1241	1.7623

Frequency (cm^{-1})	IR Intensity (KM/Mole)
57.0547	0.3541
68.7982	8.8646
75.1623	0.3333
99.9975	2.1123
105.1607	7.7672
110.9634	11.8134
159.3652	3.8781
159.4052	3.9046
182.5659	3.0450
194.9234	0.6063
196.7493	2.2517
226.7083	0.8981
242.8697	2.4452
253.6534	1.5907
260.2976	3.7533
284.8487	8.8187
326.7124	2.0191
341.4936	13.1121
345.4212	26.6954

Frequency (cm^{-1})	IR Intensity (KM/Mole)
356.0427	1.0205
372.5513	13.3172
388.2465	4.0314
412.4152	33.4439
414.2226	0.1636
416.4617	0.9486
446.0850	78.9461
460.2016	4.0539
469.8172	51.3342
506.1730	15.9647
537.9769	85.3030
553.7859	80.7324
568.8179	57.0078
602.9497	100.0971
605.8090	22.7856
610.5721	40.1406
628.9159	12.0149
633.8836	0.8119
635.2183	8.5604

Frequency (cm^{-1})	IR Intensity (KM/Mole)
682.5547	166.8257
697.7704	22.0229
711.5893	80.2823
736.1973	11.0247
747.0808	0.7722
767.0020	32.9340
768.7350	59.9349
785.9063	3.3050
795.4260	3.6452
813.3113	21.0977
827.6494	6.0469
858.6238	0.1072
867.3291	9.9149
883.4238	35.3125
887.0972	20.7650
919.3754	73.6524
933.5876	36.8557
935.2083	5.2219
951.8838	15.9040

Frequency (cm^{-1})	IR Intensity (KM/Mole)
962.7575	13.1962
967.5265	20.2671
970.0549	219.2869
988.8949	23.9687
991.3057	0.3434
1000.3206	0.3956
1008.3125	0.1458
1015.2206	0.0649
1035.6266	36.1300
1047.1262	13.2470
1050.0127	15.2111
1068.3087	43.8179
1085.3121	5.0090
1100.2707	41.9952
1105.2798	33.7703
1108.3066	45.3982
1112.5419	45.8539
1165.0111	36.2212
1174.9022	0.5617

Frequency (cm⁻¹)	IR Intensity (KM/Mole)
1184.5544	12.2653
1188.5260	91.7236
1196.2261	2.8459
1197.7395	6.8648
1206.8291	57.1333
1210.9939	12.4923
1218.6462	4.0165
1220.5233	2.4478
1261.6490	8.7231
1311.0070	100.1073
1316.8559	47.1028
1318.6687	22.9959
1322.1993	8.1142
1337.5637	26.0673
1340.4535	1.4761
1356.2239	74.6983
1368.1461	64.3529
1370.6542	10.0757
1386.6828	6.0838

Frequency (cm⁻¹)	IR Intensity (KM/Mole)
1393.3427	8.6488
1412.7834	33.2538
1413.3974	114.7157
1420.3488	109.5249
1441.6086	108.3093
1460.4157	147.1705
1467.9732	68.8581
1476.2095	27.4919
1480.9724	19.3896
1481.8102	20.0228
1486.0806	20.6398
1491.1534	157.5915
1492.9811	54.4779
1498.8980	474.4393
1504.2532	44.3846
1510.7127	68.9966
1522.7779	19.3912
1574.7365	4.7868
1613.5952	13.9385

Frequency (cm⁻¹)	IR Intensity (KM/Mole)
1618.1522	1.7085
1638.2408	18.5240
1666.9826	1055.9680
1672.4335	753.0209
2988.3622	83.7415
2998.3627	10.7278
3006.0447	57.1090
3014.3309	63.6283
3037.1566	62.0925
3045.0345	53.4461
3052.7423	33.6769
3062.7758	5.4342
3068.4732	154.1460
3075.6971	75.3340
3101.0519	2.0793
3103.2207	30.9624
3117.0333	22.0197
3152.8911	12.5544
3162.7164	0.4244

Frequency (cm⁻¹)	IR Intensity (KM/Mole)
3167.2222	0.7845
3171.5459	24.5649
3172.2298	31.5630
3182.5145	32.2380
3182.7865	34.4138
3189.8423	12.8165
3565.3962	102.7755
3569.1449	60.3443
3819.9333	29.8824

Bortezomib (de2) in water

Frequency (cm⁻¹)	IR Intensity (KM/Mole)
17.2104	1.5204
18.9281	1.8870
23.9057	1.4093
24.3867	1.4510
34.3727	1.6265
36.8555	0.9845
44.1603	0.6287

Frequency (cm^{-1})	IR Intensity (KM/Mole)
48.6576	1.5171
58.4296	0.3419
71.9241	9.2189
75.8817	0.2654
99.7004	2.6318
105.6390	6.4739
110.6174	12.1765
160.1584	5.9644
160.5904	2.3813
185.0660	2.1132
194.9901	0.5296
198.0094	2.9230
227.3830	0.7847
243.5816	2.4009
254.5804	1.7470
260.2952	3.5171
285.3548	8.7465
327.2778	3.0427
342.2119	14.6167

Frequency (cm^{-1})	IR Intensity (KM/Mole)
346.6466	26.5181
355.3764	0.6033
371.1309	13.0960
389.0292	3.8278
412.5428	34.3115
414.1370	0.0640
418.4507	0.9788
446.8000	81.5276
460.4788	4.2902
469.7780	54.6354
506.2440	15.8870
537.6282	87.8944
553.6768	79.1151
568.8532	57.7200
604.1378	81.8894
606.1332	42.4865
612.0245	36.3907
629.1164	14.2467
633.8397	1.0886

Frequency (cm⁻¹)	IR Intensity (KM/Mole)
635.3323	8.7401
684.0183	164.4668
698.9205	29.6373
711.5274	83.2025
736.6359	11.2958
747.0200	1.1302
766.6539	32.9234
768.5736	60.8506
786.0080	3.5068
795.4095	3.9110
813.4504	21.5374
827.9086	6.3743
858.0442	0.0714
867.4829	9.0663
883.5646	36.0074
887.7213	20.6356
918.9639	74.5335
933.5948	39.7326
934.8168	6.3346

Frequency (cm⁻¹)	IR Intensity (KM/Mole)
951.8391	18.6739
962.5794	17.2010
968.0385	56.5480
969.5417	179.4821
989.6692	23.8718
991.0525	0.2851
1000.9868	0.4014
1008.6161	0.1219
1015.1575	0.0528
1035.5637	36.5001
1046.9072	12.7225
1050.8998	17.2990
1068.1337	43.8784
1085.3791	6.0598
1100.1463	39.7685
1105.2656	34.2315
1108.4055	48.2204
1112.9030	50.7732
1165.1706	37.5392

Frequency (cm^{-1})	IR Intensity (KM/Mole)
1174.8777	0.6147
1184.4209	11.9248
1188.6040	92.6759
1196.1353	3.0059
1197.4566	7.6588
1206.8849	57.6225
1210.6869	13.3733
1218.7720	4.0036
1220.4824	2.6648
1263.2714	8.8634
1311.1392	97.8965
1316.2252	58.8292
1318.6921	18.3360
1322.7278	5.9158
1337.3930	27.9214
1339.9717	2.1627
1356.0114	69.9642
1368.9535	64.7652
1370.8386	16.2926

Frequency (cm^{-1})	IR Intensity (KM/Mole)
1386.4829	5.7234
1393.1085	8.9371
1412.4102	8.3902
1413.4039	141.2058
1420.5357	110.5292
1441.7666	111.8896
1460.7481	164.8312
1469.7980	64.9180
1475.2587	30.7593
1480.5314	19.9438
1481.7891	20.7935
1485.8718	26.5029
1490.3840	207.5595
1493.2013	49.6256
1497.3764	439.9495
1503.5349	28.7862
1510.9313	70.2333
1522.8205	19.3622
1574.5413	4.8114

Frequency (cm^{-1})	IR Intensity (KM/Mole)
1613.4989	14.5103
1618.0838	1.5332
1638.1519	19.0542
1665.6237	1092.3914
1671.2575	750.1850
2989.3442	83.0852
2998.1866	10.6565
3006.2391	56.7329
3013.9429	62.9200
3035.4291	69.9859
3045.8423	53.9428
3052.4997	33.8276
3062.8577	5.0394
3068.4404	157.8415
3076.0848	75.1409
3100.5681	2.2640
3102.5715	32.5073
3117.1216	22.8469
3153.1943	12.7767

Frequency (cm^{-1})	IR Intensity (KM/Mole)
3162.9060	0.3084
3167.9041	0.6713
3172.3937	33.5155
3172.4653	22.5317
3182.7320	38.0403
3183.1281	29.7645
3189.7511	13.2955
3565.8593	102.9155
3568.3984	62.1371
3818.8190	30.9815

Threonine calculations at level of theory - B3LYP/6-311++G(d,p)

Threonine (Thr1-OH) in gas phase

Frequency (cm^{-1})	IR Intensity (KM/Mole)
59.6107	0.8253
80.2839	0.4957
162.8379	0.1162
227.2413	9.5830
238.0010	9.3297

Frequency (cm^{-1})	IR Intensity (KM/Mole)
269.4773	14.4715
293.3597	40.5944
313.8397	133.6290
374.8706	10.5639
397.7397	5.1945
483.6325	5.8034
521.4249	16.7336
618.7244	115.3411
669.7161	13.4439
757.9931	16.3232
813.7376	6.0281
842.1274	119.0258
912.2575	41.4396
956.1700	51.9728
1048.0913	15.1337
1095.2981	8.4728
1115.5721	11.1802
1125.7990	74.6196
1141.2907	238.8981

Frequency (cm⁻¹)	IR Intensity (KM/Mole)
1227.6845	52.6850
1275.6749	36.7299
1289.2516	19.2941
1322.1368	28.3644
1365.2944	12.4861
1399.2605	9.2165
1429.8678	11.4700
1440.1639	17.2028
1493.5731	2.6093
1500.2983	6.8184
1670.8607	40.7147
1805.0230	276.4539
3004.8814	34.2380
3045.1110	12.7119
3055.0729	18.6452
3113.6123	16.2813
3119.9107	15.6521
3504.6019	2.6221
3580.5946	7.6415

Frequency (cm⁻¹)	IR Intensity (KM/Mole)
3742.8781	59.3613
3824.1392	25.3269

Threonine (Thr1-OH) in diethylether

Frequency (cm⁻¹)	IR Intensity (KM/Mole)
50.8981	1.5125
86.3611	0.7946
165.0721	0.0204
229.9870	11.0734
239.5433	10.6935
261.5697	23.7282
294.3722	53.5162
317.4759	174.7043
375.6789	13.0954
399.5846	8.6738
483.3661	8.4067
521.3167	23.4570
613.3871	160.5605
670.5979	17.3650

Frequency (cm^{-1})	IR Intensity (KM/Mole)
757.8957	22.7659
814.5463	8.0395
846.1210	147.0444
913.3855	57.9628
953.9168	83.7258
1049.5992	22.7109
1095.3939	12.1721
1107.5344	13.7430
1122.4707	163.6688
1141.7016	268.6197
1223.2205	69.3463
1274.8363	52.9215
1290.5827	12.7169
1321.8922	42.9485
1368.8347	16.3576
1399.8375	13.7105
1430.8050	16.2269
1441.5188	19.9667
1485.5964	3.9280

Frequency (cm^{-1})	IR Intensity (KM/Mole)
1493.0303	8.6675
1661.7573	54.3999
1775.9187	423.4656
3016.0225	42.9115
3043.2558	17.1745
3065.9763	21.1497
3110.6058	26.2063
3117.1338	24.5411
3502.4529	3.7481
3575.8395	11.6722
3730.2028	87.9458
3816.6360	45.7511

Threonine (Thr1-OH) in methanol

Frequency (cm^{-1})	IR Intensity (KM/Mole)
46.2471	1.8872
87.8807	0.9874
165.8349	0.0309
229.0657	13.6183

Frequency (cm^{-1})	IR Intensity (KM/Mole)
239.6435	12.2703
258.2439	33.0527
294.0066	50.9626
317.7519	198.3505
375.6138	16.8669
400.2196	8.4671
482.7650	10.1944
521.7285	26.5844
608.8290	187.0892
669.8033	19.9704
756.6728	26.0871
814.9803	9.0535
847.4824	156.6483
913.4044	68.9142
952.4847	103.2325
1049.4154	27.0041
1094.7595	12.0713
1104.4378	20.3644
1120.5410	218.9014

Frequency (cm^{-1})	IR Intensity (KM/Mole)
1142.2153	269.3822
1221.5804	76.1673
1273.3120	60.8233
1289.2179	10.2606
1321.7135	52.9294
1369.1941	17.3314
1397.9549	15.5589
1431.1544	20.7038
1439.4668	21.1558
1480.7766	4.6779
1489.2807	10.0273
1657.5794	63.3015
1762.0191	512.7610
3021.5328	47.5556
3041.6379	20.4853
3073.5746	22.9320
3108.3157	33.7180
3115.4073	30.3019
3500.7621	4.8374

Frequency (cm⁻¹)	IR Intensity (KM/Mole)
3573.3275	14.5062
3724.4688	99.9429
3812.0969	60.3116

Threonine (Thr1-OH) in water

Frequency (cm⁻¹)	IR Intensity (KM/Mole)
45.6618	1.9388
87.9083	1.0097
165.6711	0.0348
229.1785	14.1651
239.2260	12.4988
257.5509	34.4669
293.9119	50.4802
317.5921	200.8124
375.6421	17.5017
400.1307	8.0843
482.6428	10.3808
521.8555	26.8496
608.3779	189.9361

Frequency (cm^{-1})	IR Intensity (KM/Mole)
669.6273	20.2964
756.5126	26.4076
814.9891	9.2138
847.5495	157.5322
913.4942	70.0852
952.3395	105.5628
1049.2914	27.5804
1094.7036	11.9159
1104.2523	21.4329
1120.3709	225.1952
1142.2800	268.8685
1221.4517	76.8973
1273.3022	62.0729
1289.1125	9.9558
1321.6774	53.9959
1369.2406	17.3553
1397.7372	15.5557
1431.1544	21.4202
1439.0939	21.2851

Frequency (cm⁻¹)	IR Intensity (KM/Mole)
1480.2665	4.7485
1488.8715	10.1657
1657.1340	64.3479
1760.6311	523.1973
3021.9264	48.0109
3041.3959	20.8835
3074.1798	23.2115
3108.0570	34.7022
3115.0639	30.9593
3500.6290	4.9456
3573.0962	14.8051
3723.9720	101.0713
3811.3482	62.2161

Deprotonated threonine (Thr1-O-) in gas phase

Frequency (cm^{-1})	IR Intensity (KM/Mole)
22.8051	5.9184
89.8786	2.2061
144.3628	15.9285

Frequency (cm^{-1})	IR Intensity (KM/Mole)
166.4091	29.5286
227.5017	2.2566
248.6374	4.4035
301.3241	9.3088
333.8068	53.6895
401.1203	19.1449
468.1747	64.0257
479.6245	17.7993
568.6624	67.6775
621.1287	74.3670
643.2357	29.8771
770.9720	32.5260
811.6770	99.1843
821.5695	76.7954
909.1583	44.2093
1026.0902	20.8020
1057.4565	15.1916
1073.2997	251.2328
1131.5501	118.1896

Frequency (cm^{-1})	IR Intensity (KM/Mole)
1160.2247	21.3691
1208.4459	120.0554
1279.4230	128.5571
1302.9797	23.2341
1320.5130	25.1714
1350.9151	37.1984
1362.7627	193.5837
1429.0041	52.2036
1469.2799	0.5075
1482.7497	5.9933
1624.3234	57.4889
1740.5437	419.2689
2488.2826	585.6947
2991.0356	53.4686
3019.7916	36.7721
3059.1056	56.6438
3063.9305	56.6382
3473.6569	26.6324
3554.5701	0.4828

Frequency (cm^{-1})	IR Intensity (KM/Mole)
3764.3262	17.7087

Deprotonated threonine (Thr1-O-) in diethylether

Frequency (cm^{-1})	IR Intensity (KM/Mole)
52.9349	11.2872
87.6823	9.6662
158.3516	13.8135
188.2570	11.5424
201.5554	51.8603
257.7020	10.6629
308.6711	2.0168
386.7238	12.8911
407.7735	6.0339
488.7544	19.4668
501.5841	72.5231
589.9754	123.8737
648.2643	53.0825
688.4237	38.5497
785.5287	29.3244

Frequency (cm^{-1})	IR Intensity (KM/Mole)
826.3810	134.5274
843.6947	88.1512
925.0151	121.9547
1034.5401	38.0570
1066.4143	32.9660
1092.8595	219.6628
1127.6697	149.4210
1188.0288	44.0662
1198.2220	483.1716
1254.3663	51.5711
1305.6839	21.0443
1334.7976	31.8012
1342.0406	92.6776
1364.3583	26.0746
1432.9439	40.8461
1466.4287	3.1236
1476.3390	3.2661
1633.4649	75.9662
1735.5039	567.5229

Frequency (cm^{-1})	IR Intensity (KM/Mole)
2606.6092	539.2005
2998.8822	59.5435
3008.9148	57.4538
3063.6161	75.2955
3069.1342	74.8969
3486.8871	3.6238
3562.8003	1.6231
3748.4379	58.7374

Deprotonated threonine (Thr1-O-) in methanol

Frequency (cm^{-1})	IR Intensity (KM/Mole)
58.9875	15.4741
77.1384	12.4047
159.3721	29.5773
187.7084	62.4914
221.5176	5.3052
257.7453	9.7341
311.0946	5.7751
385.6669	8.9814

Frequency (cm⁻¹)	IR Intensity (KM/Mole)
413.1265	4.2295
485.8221	24.5168
506.4085	52.5992
592.1628	161.8082
651.4186	61.6598
710.0424	16.6240
791.1117	29.6385
832.1188	130.9056
854.0887	85.9942
930.8689	199.1982
1038.5059	41.5359
1065.4199	31.7969
1094.6676	217.0981
1120.9309	181.6162
1180.4951	508.6966
1196.4772	116.9453
1257.9358	18.3264
1304.2698	26.3481
1337.5125	29.4287

Frequency (cm⁻¹)	IR Intensity (KM/Mole)
1346.6155	63.4594
1362.1035	36.6602
1432.8049	43.0429
1466.5585	5.5438
1473.8456	2.2540
1634.7837	77.3786
1733.8607	648.4292
2669.6165	499.4783
3000.8249	63.5148
3009.0342	66.9828
3063.5074	86.2251
3072.9949	80.4331
3489.4732	0.4747
3562.2067	3.7820
3737.4639	82.3915

Deprotonated threonine (Thr1-O-) in water

Frequency (cm^{-1})	IR Intensity (KM/Mole)
51.9050	15.6287

Frequency (cm^{-1})	IR Intensity (KM/Mole)
80.5290	14.4122
155.7214	27.2065
186.4667	66.0182
224.8031	5.6168
258.1913	8.6697
311.9465	6.4921
384.9091	8.7086
413.4880	4.3850
486.0382	24.2458
507.3523	51.7724
593.0359	165.2861
651.5517	62.4752
711.9128	15.3486
791.5773	30.2323
832.6563	130.6279
855.3777	85.8407
931.8891	207.3764
1038.8855	41.3215
1065.4140	33.2789

Frequency (cm⁻¹)	IR Intensity (KM/Mole)
1094.9382	218.6293
1120.0441	185.7815
1178.9570	525.3963
1196.4840	104.6543
1258.4644	17.2765
1304.0816	26.6929
1337.6983	28.8724
1347.6727	61.5131
1362.8207	36.9514
1432.6160	43.4819
1467.3062	5.8252
1473.7736	2.2589
1634.8012	77.6510
1733.7554	658.0258
2677.7138	494.0766
3001.5819	64.7782
3008.9290	67.2772
3064.4422	86.9470
3073.2072	81.5310

Frequency (cm⁻¹)	IR Intensity (KM/Mole)
3489.5899	0.3255
3561.9571	4.0568
3736.1193	85.0867

Bortezomib calculations at level of theory - M06-2X/6-311++G(d,p)

Bortezomib in gas phase

Frequency (cm^{-1})	IR Intensity (KM/Mole)
19.1476	0.7080
28.8723	0.0723
43.5468	0.2482
56.3419	1.6212
65.9469	0.4772
73.2429	0.2279
80.0087	0.8198
82.7749	0.0109
95.8419	2.3648
97.8848	0.5506
110.9456	0.1837
118.3740	1.1409

Frequency (cm^{-1})	IR Intensity (KM/Mole)
121.9111	0.3447
122.4136	0.3113
157.3104	3.0920
175.7250	1.9169
192.3224	1.0400
203.9074	2.1493
228.2330	0.3883
232.7157	2.4481
256.6031	0.9174
264.2429	1.1537
301.3233	0.2717
311.7013	0.3177
320.5994	1.2583
343.6634	9.3784
364.7270	3.8128
374.7018	2.8315
378.6793	10.4859
385.1217	7.0462
395.4345	1.1853

Frequency (cm^{-1})	IR Intensity (KM/Mole)
416.0892	0.1854
427.5250	8.4858
436.8149	2.4362
443.1718	47.1256
460.7699	42.0368
492.7017	18.9593
512.2860	14.7280
540.5569	44.6800
550.9169	24.1061
568.0742	177.7256
581.3728	15.2784
619.4544	13.2675
630.5343	8.9575
631.7487	0.4346
655.3061	24.0182
662.6626	49.6733
709.5434	76.3969
718.0035	53.7799
726.6165	16.7871

Frequency (cm^{-1})	IR Intensity (KM/Mole)
757.0719	21.7536
768.0540	10.0596
773.8987	36.9953
788.1404	5.4648
800.5547	9.8864
811.7710	2.7999
834.7410	14.4411
846.1840	12.9431
873.6376	2.1853
878.7049	1.3368
894.9650	15.6365
906.3034	12.0138
931.7964	144.3866
937.7134	0.6487
951.2859	3.4518
962.4733	8.7092
976.2277	2.6998
989.5612	0.1856
991.1636	6.4352

Frequency (cm^{-1})	IR Intensity (KM/Mole)
1009.1875	0.3827
1015.8886	6.9111
1019.0744	0.4130
1021.9543	55.4815
1024.5632	5.7239
1029.3327	40.5636
1039.9391	25.5392
1048.7654	17.5790
1063.3590	3.6097
1093.3096	8.7504
1111.5785	8.1958
1130.4493	13.0140
1149.3751	26.2645
1164.2150	4.9011
1176.0973	0.6478
1176.7932	11.3569
1202.6928	39.6326
1207.0551	1.6358
1207.9398	1.0728

Frequency (cm^{-1})	IR Intensity (KM/Mole)
1209.8943	13.2066
1215.3168	2.5440
1234.8906	18.0624
1249.3704	6.4509
1251.8191	34.8974
1279.2080	13.5435
1301.5116	6.4523
1312.6425	18.2248
1319.5241	49.9977
1326.2439	5.5774
1340.2667	11.1377
1345.5117	1.8718
1356.5379	15.0880
1367.3162	2.1495
1372.5844	16.9353
1396.9751	58.4826
1400.0253	47.4167
1405.1703	187.8850
1409.0326	74.6517

Frequency (cm^{-1})	IR Intensity (KM/Mole)
1418.3034	131.0510
1424.2783	142.2257
1460.9834	79.4126
1471.6713	81.2033
1483.1342	1.9627
1492.5058	18.3814
1494.1125	6.3071
1495.7795	3.5898
1497.4651	34.4714
1504.5762	47.2309
1507.1874	11.4748
1515.8812	26.3348
1526.1321	9.8631
1530.4461	28.6291
1539.7039	7.8921
1631.6069	4.0745
1656.0068	1.4392
1661.1617	4.3920
1677.8143	5.0881

Frequency (cm^{-1})	IR Intensity (KM/Mole)
1777.6831	242.6475
1796.5041	650.4948
3039.3686	22.7796
3043.7690	9.8698
3046.3967	32.1006
3063.2242	14.7846
3074.5586	7.7732
3083.4557	14.4191
3097.6654	4.0669
3109.3316	6.4434
3111.4309	4.2622
3122.4351	44.0775
3125.5389	13.3450
3129.6823	19.5023
3132.6915	26.3481
3175.5392	2.3323
3184.4116	4.1659
3195.3590	0.2003
3200.6825	17.0452

Frequency (cm^{-1})	IR Intensity (KM/Mole)
3204.6241	1.5606
3214.7377	8.7888
3225.1317	8.8782
3225.3967	0.8401
3596.0934	47.8533
3605.5292	42.0811
3921.8457	19.9419
3926.4134	135.6930

Bortezomib in diethylether

Frequency (cm^{-1})	IR Intensity (KM/Mole)
17.9627	1.4214
20.3519	2.4483
26.9662	0.3741
35.0029	0.3558
39.7147	1.3330
48.9620	0.6314
61.8875	0.0493
65.9926	0.5590

Frequency (cm^{-1})	IR Intensity (KM/Mole)
70.7986	1.1926
82.3318	0.3444
94.5992	1.8044
113.0910	0.9888
124.2979	2.4348
131.5219	1.6221
175.1816	0.3254
189.6869	0.5462
198.4520	6.0549
203.9033	2.6932
230.7405	0.3425
241.0141	0.0997
257.4551	2.1082
263.8453	0.4093
288.8109	0.6292
295.0164	3.5548
333.5127	1.6121
344.7160	13.0484
355.6582	10.5552

Frequency (cm^{-1})	IR Intensity (KM/Mole)
366.2276	4.0720
382.4887	5.3729
396.8206	5.8046
415.4619	7.0079
415.9203	27.5129
439.1379	4.6911
448.1783	56.3966
459.5496	0.8789
461.7457	37.3475
485.7757	24.8723
526.4777	32.0076
547.8122	68.4379
569.2092	165.9786
588.4700	12.2339
595.6178	115.6087
604.4533	94.8852
617.6733	29.4944
629.3398	8.5920
631.3285	0.4220

Frequency (cm^{-1})	IR Intensity (KM/Mole)
642.5477	9.9003
673.8109	114.6261
710.0270	38.9865
718.5066	70.9090
755.1228	6.3030
778.2914	44.0821
780.4276	36.4491
793.8552	0.4767
800.4669	3.1839
812.3332	10.9819
829.2613	7.9293
844.4604	5.2083
875.4374	1.1411
876.7790	15.3490
894.1275	22.9589
904.3100	16.3276
933.0827	2.2671
948.2661	3.4630
950.7711	139.3265

Frequency (cm^{-1})	IR Intensity (KM/Mole)
952.3464	88.2345
968.8422	7.4942
977.2721	1.5164
983.3085	3.4767
1009.2012	0.7467
1012.0332	3.0419
1017.3484	0.6166
1017.7392	0.1859
1026.4501	0.5286
1040.9945	166.3116
1044.9633	31.5573
1060.6230	8.2109
1084.1477	5.5260
1095.0395	31.2448
1102.8729	12.4237
1117.5650	6.8285
1131.0560	49.8022
1152.2082	2.1010
1172.2844	3.9118

Frequency (cm^{-1})	IR Intensity (KM/Mole)
1175.2958	8.5523
1189.0565	42.6467
1199.9826	13.6044
1201.7349	2.6647
1202.4078	2.8316
1214.0417	9.9303
1224.2067	26.1927
1227.9915	21.7792
1238.2400	31.2095
1271.4304	23.6112
1290.3700	41.8447
1317.4761	30.0009
1320.3405	36.8743
1329.7599	5.9103
1334.1837	13.5788
1336.3341	12.2602
1357.1910	23.4930
1362.9455	23.1875
1380.1862	203.0160

Frequency (cm⁻¹)	IR Intensity (KM/Mole)
1391.7136	99.7601
1394.0837	14.6158
1401.6254	72.5840
1413.7313	252.0557
1418.3238	171.3946
1421.2767	156.5045
1447.6487	113.9970
1462.9667	100.4380
1478.3140	25.6304
1484.3222	4.0775
1490.8761	8.9071
1493.2050	10.6837
1498.9107	7.3417
1507.4342	36.5739
1509.4887	23.7984
1519.0564	4.9366
1520.5862	91.5234
1537.0883	130.4285
1538.1115	19.9879

Frequency (cm⁻¹)	IR Intensity (KM/Mole)
1628.7538	7.3907
1654.1501	1.3125
1661.8636	12.8231
1675.2514	10.3288
1757.3468	739.0071
1764.5193	824.4909
3031.0761	2.5829
3036.2172	19.5751
3043.0221	31.8562
3047.3329	37.3285
3058.9541	39.9884
3082.5515	21.6951
3091.1033	31.3044
3113.6862	7.4418
3117.9219	70.8526
3129.3650	27.8316
3133.0301	36.3821
3140.1113	3.1403
3161.3778	6.4350

Frequency (cm⁻¹)	IR Intensity (KM/Mole)
3176.0210	9.1001
3196.5892	9.8442
3200.0547	0.1386
3203.4965	0.1183
3208.7956	11.1773
3217.9079	10.9994
3218.6415	20.7233
3225.2400	8.9260
3600.6601	96.6991
3602.0606	58.3861
3904.7557	57.4350
3907.1970	204.5279

Bortezomib in methanol

Frequency (cm^{-1})	IR Intensity (KM/Mole)
10.9880	0.2266
16.5788	2.2427
18.7919	2.9969
26.3216	0.9378

Frequency (cm^{-1})	IR Intensity (KM/Mole)
36.9653	0.8015
46.9853	0.0764
57.6050	0.3086
59.6002	0.1191
70.0652	1.6082
79.3832	0.9618
93.0578	1.3287
106.8163	0.5586
120.4956	3.2252
129.8003	2.2832
175.6071	0.4490
186.6449	4.7159
192.6990	2.0696
204.6369	2.7710
230.2394	0.4128
239.9146	0.0878
256.4236	0.9653
260.9358	2.2851
286.7831	4.7621

Frequency (cm⁻¹)	IR Intensity (KM/Mole)
290.5057	0.2105
330.1181	2.5930
349.1540	15.1039
354.1916	13.5420
367.3408	1.9405
381.4359	9.3952
396.1066	7.4076
414.6795	23.5332
415.1404	12.9965
437.0868	3.5107
449.6932	82.3044
460.0785	12.3777
461.7705	18.1268
485.6706	40.7682
524.5931	45.8447
548.9796	93.4308
567.2779	158.9179
588.4243	24.5102
597.7271	89.8893

Frequency (cm^{-1})	IR Intensity (KM/Mole)
601.0533	194.3317
613.6666	9.7746
629.5396	3.0383
630.5955	0.7074
639.0990	16.4074
672.7201	133.4341
708.9254	42.6832
718.4569	77.5993
755.1266	4.8929
774.2762	24.4227
776.7635	61.6858
794.1887	1.0178
797.8236	9.6611
811.2197	11.3472
829.6554	7.8118
845.1414	7.4523
875.7844	2.6475
878.6268	8.6897
895.8272	32.0736

Frequency (cm^{-1})	IR Intensity (KM/Mole)
903.0001	17.7962
929.7415	2.3946
949.1678	3.0560
952.3826	8.8058
961.0321	259.0101
969.3786	11.5216
976.1026	2.2466
986.7820	4.0749
1010.5175	0.4237
1012.5608	3.2147
1017.2902	0.3085
1021.2044	0.5091
1027.4844	0.1784
1044.6734	44.5699
1050.1284	175.6752
1059.2814	10.1965
1083.8512	6.2582
1095.2376	41.5361
1103.4923	11.9221

Frequency (cm^{-1})	IR Intensity (KM/Mole)
1119.6829	13.2258
1135.3780	54.8531
1151.3229	4.2840
1170.4916	1.5357
1173.2621	12.7329
1190.2175	59.1759
1198.6967	8.1599
1200.3058	4.1656
1201.3885	7.5050
1213.5923	14.3539
1225.5558	18.8729
1228.3505	20.8619
1239.2833	35.8183
1271.0309	20.3557
1292.8572	54.0136
1315.7164	43.6351
1323.4012	26.6861
1329.8300	6.6468
1333.6050	7.6641

Frequency (cm^{-1})	IR Intensity (KM/Mole)
1335.3008	15.7554
1356.4499	23.8632
1360.0912	25.3838
1384.4777	299.7382
1391.1008	18.7523
1392.2967	81.9625
1399.8860	104.7197
1409.9134	345.6534
1416.8468	121.1214
1425.4381	166.0933
1447.3109	128.6240
1462.0716	118.6947
1476.8531	33.8518
1482.5065	4.6634
1487.7309	9.6126
1492.7011	11.3354
1495.4368	8.9677
1504.9014	31.7184
1506.6841	25.9297

Frequency (cm^{-1})	IR Intensity (KM/Mole)
1514.9178	15.6447
1519.0813	100.7460
1536.0002	35.3277
1542.0021	140.0070
1628.1437	9.8907
1653.4549	1.0286
1661.0507	20.5159
1673.5668	12.0050
1741.7091	1159.8386
1747.7652	740.8665
3025.9213	12.6696
3040.6086	22.9259
3043.6887	38.1116
3047.0806	40.7746
3059.6297	49.9571
3087.3399	24.6730
3093.4306	33.9760
3113.7494	10.0184
3117.4537	82.7587

Frequency (cm⁻¹)	IR Intensity (KM/Mole)
3128.6797	22.8993
3129.2810	57.7851
3145.9734	4.5981
3163.7185	8.0770
3179.1295	10.3821
3197.5926	11.4709
3201.0597	0.0395
3205.2973	0.0592
3209.1345	8.7300
3216.6103	19.0859
3219.8977	21.3269
3225.2557	12.6457
3596.6394	105.7842
3603.6711	86.6537
3893.9863	157.8368
3896.8365	140.4632

Bortezomib in water

Frequency (cm^{-1})	IR Intensity (KM/Mole)
9.7025	0.2749
20.2225	3.1913
20.6682	2.6088
28.1069	0.2537
37.1187	1.0505
45.8546	0.0525
58.1368	0.2812
60.1074	0.1936
69.3155	1.5407
80.6213	0.9854
92.5827	1.2298
105.8764	0.4730
119.8979	3.4138
130.0471	2.4063
175.3773	0.4315
185.7756	4.9264
192.8067	2.0025
204.6993	2.5897

Frequency (cm^{-1})	IR Intensity (KM/Mole)
229.8667	0.4700
239.1575	0.0498
255.3476	0.8911
261.5538	2.4078
286.5013	4.8423
290.5978	0.2392
330.0471	2.6254
349.4750	15.0385
354.2560	13.9275
367.9224	2.0288
381.6165	9.6198
396.1213	7.4493
414.5543	35.7923
415.6154	1.4333
437.3828	2.7649
450.6402	83.3726
460.3636	19.9759
463.4760	9.2776
486.2452	42.4637

Frequency (cm^{-1})	IR Intensity (KM/Mole)
525.2326	49.3089
549.0269	94.7763
567.0043	160.9153
588.2546	22.6015
597.2361	73.0045
600.5248	223.6708
613.6821	9.6787
629.4912	3.0170
630.6261	0.8543
638.7289	17.5329
672.1883	134.7105
708.9058	41.7945
719.2437	78.1445
755.1611	4.7659
773.7001	25.9054
776.5849	62.4023
794.0733	0.9133
797.8027	10.1984
811.1360	11.7497

Frequency (cm^{-1})	IR Intensity (KM/Mole)
829.7558	7.8153
845.0971	7.6884
877.0573	4.6961
878.8308	6.0951
896.0426	32.8791
903.0368	17.9831
929.5047	2.4056
949.3925	2.9582
953.3821	8.4684
962.5996	263.4297
969.6340	13.2578
976.2700	2.5007
987.0720	4.0573
1011.6235	0.4966
1012.6000	3.1098
1017.2380	0.3297
1021.5248	0.5063
1027.9634	0.1802
1044.5813	44.3270

Frequency (cm^{-1})	IR Intensity (KM/Mole)
1052.2602	180.6070
1059.0682	10.3658
1083.9191	6.3809
1095.3175	42.4858
1103.6045	12.6899
1119.7444	13.7619
1135.7052	55.4566
1151.1218	4.7273
1170.1981	1.3694
1173.1776	13.2232
1190.1964	60.9158
1198.4435	7.7970
1200.2286	4.8013
1201.4429	7.7145
1213.2495	14.9580
1225.6891	19.5634
1228.2243	19.1270
1239.2633	36.0190
1271.1528	19.6663

Frequency (cm⁻¹)	IR Intensity (KM/Mole)
1293.1347	54.3725
1315.4681	42.6559
1323.7472	28.0246
1329.6938	6.5853
1333.9460	10.1835
1335.1935	13.0322
1356.2892	23.2482
1360.0558	25.0769
1384.8011	316.1681
1390.8522	20.7146
1392.3207	81.1557
1399.7372	107.0665
1409.5296	347.3514
1416.7738	119.1199
1425.7216	165.7399
1446.9505	130.0722
1461.8708	120.3285
1476.9547	33.2988
1482.2803	4.5766

Frequency (cm^{-1})	IR Intensity (KM/Mole)
1487.2380	9.6849
1492.5037	10.8707
1494.9283	10.2727
1504.5731	31.9685
1506.2412	26.7453
1514.4406	17.6698
1518.8229	102.1910
1535.8170	35.2662
1542.0817	142.2185
1628.0618	10.0648
1653.2020	0.9866
1660.8681	21.1503
1673.3268	12.3085
1739.7373	1201.6816
1746.1152	741.1868
3026.6610	14.0043
3040.6502	23.3587
3043.6739	39.1596
3047.0349	40.9055

Frequency (cm^{-1})	IR Intensity (KM/Mole)
3059.6131	50.9381
3087.5624	24.9575
3093.6139	33.9994
3113.6772	10.7033
3117.3446	84.4639
3128.0466	31.7173
3129.0294	51.2439
3146.1670	4.8408
3163.9919	8.1433
3179.5707	10.5954
3197.9106	11.3446
3201.0837	0.0638
3205.7086	0.0502
3209.0864	8.4768
3216.4809	20.4741
3220.2002	21.4580
3225.2404	12.7619
3596.0690	106.9238
3602.7223	90.0247

Frequency (cm⁻¹)	IR Intensity (KM/Mole)
3893.1617	149.6547
3895.3483	152.0042

Bortezomib (de1) in gas phase

Frequency (cm⁻¹)	IR Intensity (KM/Mole)
20.9150	2.8577
26.2913	0.4567
39.9954	0.7601
58.4885	0.4671
65.5270	0.4178
76.7037	0.2148
81.9684	0.2743
88.9708	1.4143
95.9902	1.0449
101.0719	2.2232
118.6475	0.3507
126.7974	0.1317
133.0734	0.9343
143.3438	13.3019

Frequency (cm^{-1})	IR Intensity (KM/Mole)
158.2497	0.9157
191.0226	0.3395
194.6751	14.7196
206.8005	1.5019
234.5176	2.0658
243.1764	4.2044
254.8777	6.2496
271.5396	3.3961
306.2772	1.0366
307.7079	1.2532
322.7985	2.4425
336.8696	17.4333
362.1527	10.4982
381.5597	8.4879
384.4793	5.8949
395.5471	2.7511
415.0291	0.4505
427.8913	10.8032
438.8456	1.2435

Frequency (cm^{-1})	IR Intensity (KM/Mole)
447.5779	31.0223
472.1719	9.5607
501.6855	7.6817
517.3791	15.9665
539.0983	52.9552
558.0732	3.5253
583.8260	44.6370
594.8067	72.5359
614.3969	5.6765
631.9582	0.7620
632.6602	5.4052
652.5874	26.7098
694.9030	6.3742
714.6459	22.0104
716.4787	92.4326
755.1734	15.6980
760.1207	48.2412
763.3121	13.1407
774.9119	3.1825

Frequency (cm^{-1})	IR Intensity (KM/Mole)
799.9445	12.2623
803.6083	50.7564
812.9448	9.9049
846.3135	67.1883
849.2365	19.2233
872.5487	3.6121
878.8191	3.8505
887.5187	17.9694
907.5043	23.7627
935.9310	0.6420
946.6026	6.3531
955.5406	7.7101
975.6685	5.6708
977.0568	0.5420
984.3367	24.6901
1003.9754	0.5545
1009.9332	145.8133
1015.4928	1.6340
1016.0219	106.0653

Frequency (cm^{-1})	IR Intensity (KM/Mole)
1020.4507	4.4822
1023.2292	0.7341
1039.2369	17.2985
1051.0619	17.2544
1063.7057	4.8625
1095.3276	29.5487
1110.0424	15.1078
1113.9800	19.7568
1137.8284	10.7579
1143.0211	64.4661
1157.9109	1.5621
1173.7516	0.4392
1178.4897	28.6661
1202.8208	38.6905
1206.4976	10.2669
1208.2278	8.6409
1208.8749	2.7081
1211.9553	0.3267
1233.5145	31.9894

Frequency (cm^{-1})	IR Intensity (KM/Mole)
1243.3869	18.0490
1248.9569	6.4192
1283.2206	6.2533
1313.9087	3.5398
1318.4494	53.0947
1327.1419	9.5860
1342.5642	12.9929
1343.0627	0.4154
1344.6456	2.9387
1355.3900	45.1982
1364.7188	3.8636
1377.7405	2.6453
1396.1250	20.4644
1396.9891	7.8517
1413.8312	112.7569
1419.8483	16.8211
1450.9365	32.7474
1469.1643	73.6866
1476.4898	43.7478

Frequency (cm^{-1})	IR Intensity (KM/Mole)
1480.9798	74.3457
1490.3850	51.2156
1491.2932	3.2609
1493.6626	7.0387
1495.5760	13.4473
1504.4776	6.7564
1520.6957	57.2241
1521.6630	16.5356
1536.8906	35.5927
1539.3276	1.9275
1575.3994	369.0038
1627.8744	7.8735
1655.1420	2.0964
1661.7678	9.2340
1677.5866	8.1528
1745.7022	457.8311
1757.1409	589.7611
2995.5136	59.6361
3032.7844	33.2062

Frequency (cm^{-1})	IR Intensity (KM/Mole)
3040.4835	55.2057
3043.4647	8.5343
3052.1147	29.7954
3064.2399	15.1924
3086.0581	7.3663
3100.5553	9.6487
3110.7674	1.3417
3111.8752	49.5266
3113.3996	49.0925
3135.2135	14.7463
3136.5069	9.6038
3180.9893	5.5749
3190.7022	41.3879
3194.4327	1.2082
3201.7779	6.0045
3205.9212	16.6629
3211.0592	12.8899
3221.2323	17.5264
3221.8095	1.8602

Frequency (cm⁻¹)	IR Intensity (KM/Mole)
3471.7254	138.0927
3602.4890	24.4633
3903.1826	25.0076

Bortezomib (de1) in diethylether

Frequency (cm⁻¹)	IR Intensity (KM/Mole)
31.2771	1.6541
44.9936	0.3603
55.3625	5.6225
58.6056	0.0813
73.4025	1.2473
79.6244	0.6258
85.7265	1.0545
91.8735	0.9900
94.8154	2.2337
100.3670	0.2907
118.2501	1.9494
127.8447	4.2091
131.4934	0.5605

Frequency (cm^{-1})	IR Intensity (KM/Mole)
137.3961	10.5140
155.5836	5.4580
182.5485	8.3460
193.9001	3.4751
207.4321	5.1925
221.4420	9.4553
234.3271	7.1025
248.0404	18.0898
276.3029	0.9948
301.6975	0.1398
309.9070	1.6229
321.3125	3.0132
340.2455	21.6668
355.8991	15.6069
373.0628	11.0245
380.4987	6.7867
391.0493	3.6178
415.9458	0.3079
429.8583	23.6536

Frequency (cm^{-1})	IR Intensity (KM/Mole)
435.4706	3.2834
440.9667	35.6914
475.7990	35.5256
497.5877	12.6155
514.3855	14.8659
537.7840	90.6429
555.3045	33.5213
571.8840	110.0190
583.0915	25.1294
615.9829	15.1350
629.9639	10.5410
631.0613	0.2333
653.9442	25.3703
694.8045	2.6219
711.6473	95.3792
716.5640	82.9814
749.5121	68.0482
756.4520	36.1382
765.2830	4.0375

Frequency (cm^{-1})	IR Intensity (KM/Mole)
773.3875	22.4591
791.9372	54.6139
800.7070	18.7253
810.4915	5.3925
838.5987	40.7900
845.6805	34.8266
874.4733	3.9865
877.6168	2.5605
903.5171	40.0124
909.1390	13.0578
929.7755	0.4289
949.8989	8.6456
959.0044	10.4592
973.7507	2.9172
984.4877	25.2631
984.9494	54.8407
1001.4498	234.4835
1009.0091	0.2553
1017.4233	0.6931

Frequency (cm^{-1})	IR Intensity (KM/Mole)
1024.3928	1.4566
1026.3577	25.9806
1034.9240	3.1561
1038.6726	31.1114
1049.0883	22.4095
1061.6331	7.2252
1091.7321	19.6945
1109.0444	15.0279
1121.5801	39.3645
1131.3822	68.3868
1143.2927	60.3535
1163.1452	8.2606
1172.0482	0.6497
1176.1843	25.8480
1201.5922	4.6818
1203.9564	50.8546
1209.7897	16.9302
1211.1873	10.5583
1213.7005	0.6024

Frequency (cm⁻¹)	IR Intensity (KM/Mole)
1229.8454	44.5596
1246.5304	14.3275
1250.5390	8.9417
1290.0849	6.4105
1306.5968	7.8126
1318.3354	36.1363
1327.7762	24.4014
1328.4895	10.6369
1342.9781	22.5914
1347.6268	2.9061
1358.2374	33.0461
1364.9203	3.9451
1377.0841	3.9674
1396.0318	15.8521
1402.8618	10.6952
1413.3880	63.2288
1414.5892	172.2138
1457.2425	54.8860
1471.9924	60.4552

Frequency (cm^{-1})	IR Intensity (KM/Mole)
1477.9264	6.7269
1482.7032	124.2324
1487.2881	9.2056
1490.4845	7.8785
1492.0209	57.9812
1494.5425	28.7336
1498.3473	35.3789
1508.7781	103.1630
1511.3903	18.2139
1531.0196	76.7380
1537.8578	6.8506
1551.0262	480.7111
1627.6747	12.9553
1654.2493	2.4573
1662.9654	15.9736
1675.7519	8.9059
1719.1722	645.1888
1740.9699	961.5490
3014.4656	60.3230

Frequency (cm⁻¹)	IR Intensity (KM/Mole)
3036.4946	37.4163
3042.1195	48.6242
3059.9666	31.7869
3062.5910	25.6295
3069.9769	17.4349
3086.4245	11.0128
3102.7668	7.1319
3112.3216	7.2274
3112.4742	88.4913
3119.9787	42.5784
3134.6800	16.7970
3141.7896	20.7635
3186.0121	6.2935
3195.9996	0.1370
3202.5983	0.7587
3203.4596	3.7204
3213.1282	17.2379
3219.3642	10.8258
3222.9949	15.1357

Frequency (cm^{-1})	IR Intensity (KM/Mole)
3223.6007	4.5694
3494.9544	186.1176
3597.4474	56.9672
3885.1940	44.2864

Bortezomib (de1) in methanol

Frequency (cm^{-1})	IR Intensity (KM/Mole)
17.7821	1.5374
32.5939	1.8295
33.4856	0.1440
40.3567	2.3056
47.8509	0.0404
53.1148	1.1902
67.9455	1.1123
73.6328	1.7031
80.3807	2.2280
101.0103	1.0794
104.0036	7.3482
107.6613	5.6618

Frequency (cm^{-1})	IR Intensity (KM/Mole)
118.9224	0.6542
133.0963	1.2124
170.8165	8.6853
180.7991	1.3802
197.8865	1.4897
205.3636	3.6127
230.1417	11.5260
237.3097	13.7561
266.3295	8.3078
277.0516	3.3225
286.1470	10.0513
289.5029	0.4735
332.7515	5.2845
346.0781	33.5485
360.4545	1.8473
369.4203	5.3203
382.8745	7.0987
392.4311	13.9191
411.7931	13.9254

Frequency (cm^{-1})	IR Intensity (KM/Mole)
415.0403	0.2819
430.2256	27.7685
436.7586	16.2656
461.7801	59.5415
489.7825	57.6369
524.6034	46.5322
544.7961	89.3903
565.5611	30.4885
582.8069	159.8300
590.7785	8.0311
616.6580	58.9455
627.8143	14.4903
630.5882	0.2745
646.9281	35.1041
657.1170	12.0554
718.4090	74.2574
729.7599	61.6254
748.4771	31.6442
755.9308	48.0217

Frequency (cm^{-1})	IR Intensity (KM/Mole)
773.7837	37.5837
785.0865	10.7549
795.6831	101.5109
799.5749	24.8556
815.3112	7.7219
825.4503	24.8754
849.2515	17.6190
856.5273	32.3826
875.8215	1.3473
896.2656	25.5074
906.0536	21.9760
931.4092	1.1004
944.4693	3.7423
948.7576	11.4018
976.0648	1.4605
986.2908	3.1478
994.6103	97.9089
1003.8479	245.8452
1009.1968	1.5883

Frequency (cm⁻¹)	IR Intensity (KM/Mole)
1016.4587	0.2134
1020.2122	6.6173
1023.2476	0.6665
1025.9570	0.5063
1045.5922	43.6639
1058.6607	9.2874
1089.2659	17.7467
1096.3990	34.6240
1106.7106	13.1199
1118.9937	8.2988
1135.5135	141.6170
1138.5906	56.2832
1155.7074	5.7913
1169.1871	2.7514
1174.7740	79.2483
1189.0486	20.0110
1194.7422	33.2911
1195.5043	43.6225
1202.5425	14.3097

Frequency (cm^{-1})	IR Intensity (KM/Mole)
1211.1242	12.0800
1218.4006	29.5674
1231.9845	33.2551
1234.7321	3.8035
1276.3306	15.4053
1307.6545	21.5017
1314.1599	12.9488
1318.8805	52.8812
1328.4544	2.7349
1331.1166	9.6530
1336.9753	8.5010
1353.9443	3.0102
1369.9252	7.4559
1378.1976	33.1010
1392.0613	3.6291
1396.6023	15.8760
1403.2493	271.4722
1411.8756	23.6724
1434.7140	85.5353

Frequency (cm^{-1})	IR Intensity (KM/Mole)
1459.7334	76.2717
1475.1548	6.4209
1481.1525	3.7694
1484.5242	31.1023
1488.2173	158.1078
1491.4509	3.0305
1493.9024	67.0141
1502.9717	6.4269
1506.3437	51.4273
1515.8242	93.2389
1525.9848	465.9952
1531.7599	272.1721
1536.3212	64.3785
1623.2427	11.4853
1651.1926	2.6832
1658.7555	11.5169
1673.7439	16.4940
1709.3861	1116.6498
1735.3480	1096.8710

Frequency (cm^{-1})	IR Intensity (KM/Mole)
3012.9874	51.5780
3036.0598	109.8820
3037.1456	39.8659
3038.7587	8.3202
3046.8794	23.4070
3073.8329	38.4459
3091.0372	24.0347
3105.3036	15.5189
3109.3151	95.9440
3119.4491	48.8386
3131.4712	27.7848
3145.8383	6.4808
3184.0080	5.3356
3197.6531	0.3338
3204.8228	0.4307
3206.6608	6.8384
3210.5451	7.3870
3213.3659	18.8164
3219.9814	15.6591

Frequency (cm^{-1})	IR Intensity (KM/Mole)
3222.6310	15.9163
3233.1328	0.6738
3487.3128	278.0572
3592.4041	97.6191
3889.8958	53.6501

Bortezomib (de1) in water

Frequency (cm^{-1})	IR Intensity (KM/Mole)
31.1786	1.3274
31.8507	1.1445
35.7620	1.1318
38.1182	2.1095
48.6422	0.2600
53.0378	1.0839
69.0037	1.5490
76.1955	2.1684
80.2049	1.5925
100.1712	1.4322
103.0914	8.6392

Frequency (cm⁻¹)	IR Intensity (KM/Mole)
106.5584	3.6142
118.9516	1.0440
131.8707	1.2767
170.5129	8.8382
180.2064	1.3775
197.4048	1.6959
205.5145	3.2177
229.3825	11.0187
235.0273	14.9659
266.1326	8.2040
277.8007	3.3138
286.0317	9.2478
287.8879	1.6804
330.4088	5.4331
346.0016	33.9292
360.0654	2.0595
370.0029	5.6667
382.1010	6.6026
392.6814	15.6243

Frequency (cm^{-1})	IR Intensity (KM/Mole)
411.6824	14.3199
414.9124	0.3148
431.5848	34.0456
438.1799	9.3735
461.7725	61.6509
489.8858	57.8335
524.4302	50.5884
544.2580	89.5503
565.2914	30.8284
582.2836	165.8556
590.4323	8.1331
615.2997	62.2550
627.5771	13.2871
630.4886	0.2725
646.3636	33.9547
657.0455	11.7249
718.3164	73.8625
729.3196	67.2539
747.6547	38.0765

Frequency (cm^{-1})	IR Intensity (KM/Mole)
755.5337	46.8685
774.2393	36.3281
785.2189	13.5715
792.7244	102.3274
799.6802	23.5778
815.5135	8.0975
825.0789	23.2706
848.8868	17.2825
856.3683	32.6640
876.0221	1.2237
896.4354	26.0784
905.4801	21.9425
930.8350	1.0599
944.8510	3.6899
948.8574	10.9284
975.6362	1.4020
985.9642	3.2257
994.2198	109.2975
1003.3555	237.2881

Frequency (cm^{-1})	IR Intensity (KM/Mole)
1009.5274	1.2533
1016.4463	0.2376
1020.0058	6.9745
1023.6618	0.7389
1026.3797	0.5381
1045.6016	44.3085
1058.6132	9.5603
1089.2954	20.2455
1096.3658	35.2217
1106.8234	12.4513
1119.0273	9.1821
1135.4398	150.1084
1138.4130	56.2355
1155.5025	6.1471
1169.0543	2.6007
1174.9680	78.3522
1189.0791	19.5979
1194.8173	25.8568
1195.7347	54.2707

Frequency (cm⁻¹)	IR Intensity (KM/Mole)
1202.0266	14.3550
1211.0614	12.3282
1218.2055	30.4671
1231.8417	33.6821
1234.5951	3.8403
1276.3533	15.3793
1307.4228	20.8210
1314.1780	13.6740
1318.9382	55.0232
1328.5707	2.7144
1330.9382	10.3999
1336.6435	8.1401
1354.0623	2.9549
1369.3043	8.2424
1378.1477	34.0106
1391.6862	3.1658
1396.1792	16.0112
1403.1122	279.5939
1411.0166	23.9261

Frequency (cm^{-1})	IR Intensity (KM/Mole)
1434.5431	87.7331
1459.7423	78.7337
1474.7987	6.4541
1480.2442	4.6105
1483.9717	35.9999
1487.9922	140.2514
1491.2800	4.9192
1493.1064	85.8450
1501.8628	11.8903
1509.2874	48.8571
1515.5101	119.0328
1524.2946	492.2444
1531.5771	239.8799
1536.2493	56.5705
1623.1226	11.7626
1651.0348	2.6859
1658.9697	11.8847
1673.5822	16.8353
1708.0515	1138.7055

Frequency (cm^{-1})	IR Intensity (KM/Mole)
1733.8857	1110.6610
3013.6318	52.8825
3035.8778	51.5487
3037.4743	66.4908
3038.9299	31.4417
3046.9345	27.3636
3074.1236	36.9649
3091.0846	24.4724
3105.2502	16.6476
3109.4921	99.4309
3119.7861	50.0218
3130.9842	29.2487
3145.8534	6.7709
3184.2556	5.4868
3197.6897	0.3915
3205.5967	0.3609
3206.8498	6.8256
3210.7168	7.5759
3213.4798	19.6040

Frequency (cm^{-1})	IR Intensity (KM/Mole)
3220.5199	15.7666
3222.7142	15.8959
3232.5226	0.6376
3489.1296	276.8941
3591.8721	99.4619
3889.4780	54.4768

Bortezomib (de2) in gas phase

Frequency (cm^{-1})	IR Intensity (KM/Mole)
16.1089	1.3167
26.7472	1.1203
41.7646	2.5580
55.7120	0.9005
62.8419	0.9274
70.2786	3.3474
76.5529	0.2670
78.9319	4.2287
95.0995	0.2273
101.3172	2.2100

Frequency (cm⁻¹)	IR Intensity (KM/Mole)
116.1706	1.2998
120.1260	0.6110
122.9438	4.1686
142.2883	4.0213
158.6081	0.4219
179.2071	4.2273
194.3500	1.4450
204.6334	4.0235
215.9015	2.4437
230.2025	2.1094
254.4857	3.5057
264.5951	2.3129
293.6182	0.5328
310.0228	0.1551
318.3260	2.1515
343.0242	11.0624
366.0929	7.7773
376.6594	4.3718
380.2643	6.2997

Frequency (cm^{-1})	IR Intensity (KM/Mole)
394.2413	0.8633
414.5773	0.1708
430.7861	12.1122
438.2031	2.2042
445.0245	34.7634
473.2682	25.4779
484.0785	22.6724
514.6019	13.3968
532.6194	28.4558
544.8581	37.5969
576.8530	79.8506
584.6365	15.4455
625.5418	6.4472
631.7860	2.8388
632.5081	13.2561
658.9506	17.6943
686.0451	4.6595
708.8600	50.2122
714.7751	54.9979

Frequency (cm^{-1})	IR Intensity (KM/Mole)
746.2479	48.5996
759.2215	18.6169
764.1268	13.0107
772.3358	18.8627
788.8677	36.2390
801.5283	11.0769
811.0513	3.0880
834.3766	29.4801
839.7134	9.7092
871.3918	1.5347
875.5462	2.0731
899.4685	25.7563
909.7025	16.9610
933.6685	0.7188
945.9058	4.0098
948.8320	47.8574
975.7511	5.7817
977.9677	1.2663
986.9540	7.6440

Frequency (cm^{-1})	IR Intensity (KM/Mole)
994.0258	135.7341
1004.5773	1.3653
1014.8300	0.1697
1021.2109	11.2451
1021.8092	0.3204
1028.9915	5.5763
1044.1216	22.2031
1052.0622	12.6089
1063.8656	5.3986
1095.5209	17.1485
1109.8471	7.9936
1114.2357	53.8420
1134.5034	6.3371
1144.9801	9.5216
1160.5060	16.7757
1172.7924	0.6140
1177.3381	10.9050
1202.1631	45.1396
1205.5963	9.4344

Frequency (cm^{-1})	IR Intensity (KM/Mole)
1206.7002	2.3132
1207.2996	9.0153
1211.9028	2.1622
1228.2764	26.1624
1240.4154	12.8610
1250.4084	6.4285
1283.7887	3.6823
1300.6958	1.9315
1320.6623	17.1097
1327.3848	14.7128
1336.0540	0.8423
1342.7901	13.6436
1349.1363	4.4702
1355.7391	26.0577
1365.8191	2.9666
1376.6510	2.5966
1395.1108	20.7735
1402.7119	4.2100
1406.4862	150.6747

Frequency (cm⁻¹)	IR Intensity (KM/Mole)
1421.7429	11.7946
1463.8276	12.4000
1469.7723	67.5678
1476.1317	3.1559
1479.8825	97.9198
1491.4952	2.0123
1492.0547	18.9353
1494.1852	7.2032
1497.6552	53.8200
1501.5278	44.3341
1504.5465	16.7080
1519.0331	34.0099
1533.5425	31.9542
1539.4856	7.5596
1597.9460	402.1629
1626.8139	7.7666
1656.0490	1.5962
1663.0453	8.4917
1678.3231	6.7509

Frequency (cm^{-1})	IR Intensity (KM/Mole)
1740.3124	358.3264
1777.9411	637.0463
3004.8501	50.0339
3034.2937	28.6982
3039.6499	50.1531
3055.4132	10.8948
3055.6667	19.6529
3072.5200	16.0207
3098.9150	7.1241
3104.7453	4.8129
3106.8874	66.1773
3116.9217	37.4220
3118.1622	6.0166
3134.7991	9.0860
3144.3085	9.3319
3184.0358	4.4475
3190.0054	8.3702
3193.7407	0.8441
3201.3158	4.6308

Frequency (cm⁻¹)	IR Intensity (KM/Mole)
3210.2353	8.9802
3210.6481	14.0689
3218.8484	0.8672
3221.4491	16.7168
3560.7564	80.7109
3610.6811	29.0244
3908.9581	29.2301

Bortezomib (de2) in diethylether

Frequency (cm^{-1})	IR Intensity (KM/Mole)
28.0748	2.0723
30.1403	1.6588
35.7270	0.4904
37.7830	0.3283
45.6323	1.0654
46.6438	0.2008
66.9531	2.6931
70.8517	2.7168
78.9162	1.3773

Frequency (cm^{-1})	IR Intensity (KM/Mole)
83.1178	1.2497
105.3802	5.0436
117.3225	2.3960
126.0736	4.4349
142.7715	7.1556
182.0506	1.6421
192.0494	9.0133
194.1015	2.1512
201.8694	1.3859
231.9492	0.2710
258.6654	2.1156
261.3019	2.0307
280.9947	3.1089
290.6754	6.7233
299.4465	0.8738
332.3596	0.9140
347.4633	13.5916
354.7412	16.9634
372.9114	1.6567

Frequency (cm⁻¹)	IR Intensity (KM/Mole)
385.2787	2.7845
399.3452	7.3863
414.9467	33.4649
417.6872	0.4952
447.5541	69.9864
455.6305	2.9070
462.7608	7.7869
486.7870	21.7685
521.1792	34.9496
551.0470	54.9847
569.7174	81.0132
586.8059	16.8417
594.1275	163.3760
609.9393	1.8252
622.8474	10.4789
630.9861	0.4954
637.3345	2.3516
640.8884	6.2425
712.9985	109.0063

Frequency (cm^{-1})	IR Intensity (KM/Mole)
716.8217	15.3017
720.7525	106.4379
754.6700	18.9774
776.8721	24.6683
778.9510	39.7466
787.6241	7.7527
795.0019	3.8330
810.7022	3.8989
825.3011	16.7762
840.2544	4.8098
872.9746	1.4418
878.2297	16.2913
893.6197	24.8801
911.5093	13.8808
934.9949	8.0789
939.1465	18.6893
949.0920	7.2259
966.9696	3.0054
977.3241	1.3909

Frequency (cm^{-1})	IR Intensity (KM/Mole)
983.2483	16.9670
986.4689	274.1934
1006.5093	0.4792
1008.3924	17.9343
1017.0737	0.6586
1017.2327	0.0926
1023.5901	0.7858
1044.9348	34.5100
1060.8551	8.4711
1081.1318	6.3435
1091.9100	53.7450
1101.4103	3.3685
1110.8572	14.1661
1123.6597	97.7729
1133.7703	52.6015
1149.5819	2.1725
1170.5994	3.7431
1174.1845	17.5533
1188.7139	62.5149

Frequency (cm^{-1})	IR Intensity (KM/Mole)
1199.3222	9.0741
1200.5894	5.4933
1201.8328	1.1678
1214.1234	11.7282
1223.9243	31.6063
1228.4734	18.3572
1238.9209	23.9934
1271.9349	9.6383
1313.6268	24.2913
1317.8360	21.6210
1318.1812	23.4062
1328.7558	6.4416
1334.3003	25.4955
1340.8463	0.7796
1357.9765	20.0745
1367.2647	15.3940
1377.7165	146.3479
1390.4739	4.5433
1397.1858	14.7765

Frequency (cm^{-1})	IR Intensity (KM/Mole)
1418.6139	19.8053
1423.9188	113.6047
1448.9706	49.4564
1460.4801	47.7116
1478.9224	0.4171
1483.5390	8.6888
1489.5113	6.0539
1490.3868	110.8565
1493.3617	21.7162
1501.3381	32.4426
1508.0995	38.8957
1512.7238	155.8754
1521.7772	41.4043
1536.7270	38.6989
1539.8393	100.9852
1559.9407	654.4130
1629.5159	8.1777
1653.9186	1.7185
1662.2160	15.4811

Frequency (cm^{-1})	IR Intensity (KM/Mole)
1674.8645	12.2309
1731.6935	812.5675
1753.1622	888.3112
3018.7051	58.7685
3026.1808	15.4478
3040.1222	36.3894
3041.4306	66.0537
3046.7504	30.2413
3074.3676	25.9566
3081.8343	24.2897
3108.7586	11.1846
3113.0482	80.0802
3121.6476	39.6220
3135.9041	26.1300
3146.9429	4.1157
3170.1928	4.8870
3179.6569	8.7908
3193.8971	12.9410
3199.3685	0.2554

Frequency (cm⁻¹)	IR Intensity (KM/Mole)
3200.1428	0.2405
3208.7397	13.0053
3215.8326	23.4717
3217.3695	10.8690
3224.5074	12.0356
3583.8232	144.2911
3612.8022	62.9505
3902.7684	48.2129

Bortezomib (de2) in methanol

Frequency (cm^{-1})	IR Intensity (KM/Mole)
16.0214	0.7582
26.9317	3.3585
29.1385	0.2759
32.6632	1.1117
40.8821	0.4145
52.0950	0.2455
62.7535	1.3540
72.0972	2.7344

Frequency (cm⁻¹)	IR Intensity (KM/Mole)
76.3087	2.0272
79.9433	5.0654
86.0543	4.1491
105.5125	1.9279
121.9182	5.1431
131.6371	8.6793
175.7571	2.6515
182.1471	8.8347
196.0702	0.5257
208.3432	1.9123
218.8850	2.5508
231.7831	0.6620
262.7664	1.6484
269.0265	6.2984
284.9121	6.5954
285.9432	3.9573
338.0242	1.6778
355.1998	18.7418
359.0233	22.0687

Frequency (cm^{-1})	IR Intensity (KM/Mole)
369.3272	0.6949
381.2595	3.6915
396.9181	8.1267
414.7561	2.7101
416.1081	32.2359
438.2531	27.0142
447.1826	78.3551
467.8053	1.1230
477.6367	69.3916
511.4219	39.2314
544.0537	60.4213
565.4014	58.9430
588.4418	12.9068
597.6048	184.1764
610.2285	5.2992
624.6389	44.0836
629.6574	3.1701
630.7267	0.2799
642.1556	9.6814

Frequency (cm⁻¹)	IR Intensity (KM/Mole)
704.4923	143.6769
715.1610	30.0298
719.6412	81.1252
752.1928	14.5018
771.1185	34.9035
776.7723	42.9865
786.8587	9.9227
797.9664	12.6263
809.8508	4.3328
825.3531	20.6938
841.8311	2.6626
876.5211	1.7777
879.6993	9.4536
897.4277	29.9410
908.7584	17.8766
929.3385	6.8479
940.4829	22.5847
953.6790	4.4248
967.0071	6.5331

Frequency (cm^{-1})	IR Intensity (KM/Mole)
976.8341	3.6227
985.3337	324.2382
990.9351	2.5623
1004.7397	29.1518
1010.5463	0.5151
1016.8524	0.2979
1021.7474	0.5569
1027.8167	0.2976
1045.3234	40.0804
1058.8784	11.0467
1082.8964	3.8077
1094.5056	79.8004
1101.9681	2.7054
1106.2934	22.3570
1122.5660	80.7394
1140.1687	59.8846
1150.6137	7.7326
1170.2599	4.3406
1172.8094	42.9871

Frequency (cm^{-1})	IR Intensity (KM/Mole)
1190.9920	66.9224
1198.4434	9.5840
1200.5491	9.1199
1202.7853	7.5509
1212.6133	16.0696
1222.4751	27.8584
1229.1268	24.6973
1239.4511	31.2227
1267.9173	14.2800
1308.5848	55.2406
1317.5848	21.2456
1321.5793	8.7350
1329.0796	7.6523
1334.0305	24.4044
1341.3869	4.9688
1356.7953	32.5487
1366.3590	14.2133
1381.6447	153.4874
1390.6720	23.3453

Frequency (cm^{-1})	IR Intensity (KM/Mole)
1396.9201	15.4772
1412.4473	20.5501
1427.4198	101.1931
1445.8580	46.6769
1458.1201	59.5383
1475.1868	1.4432
1476.9794	14.5496
1484.2431	53.7278
1487.4206	161.0574
1492.6991	6.1746
1493.7637	85.7020
1503.1264	62.2451
1512.4776	78.4017
1513.8201	147.6858
1536.1820	35.7484
1539.4433	540.2130
1541.6467	241.9372
1629.0197	7.4326
1653.3689	1.0755

Frequency (cm^{-1})	IR Intensity (KM/Mole)
1659.7761	17.8338
1673.1528	13.2835
1721.3029	1024.5703
1743.3079	941.2487
3023.0827	17.0902
3029.7475	48.9661
3040.3590	32.7329
3042.0794	107.0335
3046.0119	34.8424
3080.3343	32.8220
3086.5673	24.9310
3109.4214	5.9525
3113.2938	105.6844
3120.0689	45.4131
3129.7270	33.1852
3148.3074	4.9804
3161.6855	7.8584
3177.1576	10.4149
3197.6212	10.4321

Frequency (cm^{-1})	IR Intensity (KM/Mole)
3199.3028	0.0518
3205.0985	0.1163
3208.3571	12.0605
3216.6110	19.0166
3219.3693	22.1259
3223.8153	11.0777
3584.0701	168.2704
3602.3102	83.4093
3898.9134	58.8358

Bortezomib (de2) in water

Frequency (cm^{-1})	IR Intensity (KM/Mole)
10.4192	0.6677
25.4796	3.5077
28.0483	0.3655
28.9715	1.1624
39.0804	0.4073
50.6268	0.1982
62.4163	2.1960

Frequency (cm⁻¹)	IR Intensity (KM/Mole)
71.4199	4.6893
73.9651	1.1534
85.1981	0.7871
91.6928	6.3255
108.2004	2.0656
123.4583	5.6845
133.7256	8.5621
175.3968	4.1939
185.9186	7.7944
196.8390	0.8248
208.4085	1.1985
230.1496	1.9689
232.1434	0.8634
259.1653	2.1714
269.3888	6.4376
283.3121	6.3115
284.1588	3.9618
335.6060	2.1112
356.0610	22.4666

Frequency (cm^{-1})	IR Intensity (KM/Mole)
359.3850	19.0414
368.9158	0.7463
380.0638	3.3644
396.5381	7.3338
412.6542	0.5779
416.8227	34.2097
433.8747	47.5298
444.3132	60.9661
469.0060	2.0345
476.9403	76.7598
510.5262	39.0721
543.6410	60.0126
564.3431	57.0921
585.8640	20.4372
595.0636	188.8694
609.1938	4.5813
625.4281	42.7159
629.2554	2.8173
630.4244	0.2310

Frequency (cm⁻¹)	IR Intensity (KM/Mole)
643.7933	8.5669
709.0979	127.6434
714.8211	42.2886
716.3273	91.3457
751.4123	14.9099
770.2877	34.2551
775.5418	44.1376
787.2870	9.2664
798.3697	12.7725
809.9682	4.6124
825.8536	22.4483
841.6967	2.7200
871.3496	1.1451
879.0160	10.0549
897.3169	29.3039
909.3206	17.8793
929.6224	8.3473
940.6539	23.5905
949.1392	4.3974

Frequency (cm⁻¹)	IR Intensity (KM/Mole)
966.8398	7.4206
977.0863	3.5053
981.5682	320.9170
991.8694	2.6914
1005.6651	25.3277
1007.4074	1.2184
1016.6346	0.2589
1021.4403	0.5522
1025.9333	0.1783
1045.1637	40.7339
1058.1391	11.4897
1083.1300	3.4728
1094.3602	84.5783
1101.3893	5.7786
1106.5076	17.5289
1121.6769	88.9241
1140.5247	61.1022
1150.9676	7.6732
1169.6730	1.8400

Frequency (cm^{-1})	IR Intensity (KM/Mole)
1173.0122	45.6700
1191.2509	62.6958
1197.1299	10.4627
1200.6073	14.7968
1203.1292	7.6058
1212.2046	16.5616
1222.4610	30.6098
1228.8328	26.0706
1239.0088	31.0601
1269.6971	14.9836
1308.5331	59.6517
1317.3399	19.6825
1322.3283	11.7046
1329.3106	7.6833
1333.7403	23.0470
1342.1309	5.9876
1355.6008	33.1194
1366.5957	15.5030
1382.3494	158.8048

Frequency (cm^{-1})	IR Intensity (KM/Mole)
1391.4205	22.8870
1397.0067	16.4182
1412.0310	19.1621
1426.3644	98.9251
1445.5665	51.1599
1458.2538	52.2171
1474.9166	1.5201
1478.4655	12.6479
1484.2231	58.7846
1487.2367	167.1396
1492.3355	5.1461
1493.6192	101.2162
1502.3742	65.8219
1511.2227	94.3550
1513.4901	138.1703
1535.5150	35.5110
1536.6573	654.2798
1540.7134	120.8155
1628.7573	7.2053

Frequency (cm^{-1})	IR Intensity (KM/Mole)
1652.9395	0.9824
1659.9110	18.1286
1673.0315	13.3979
1720.0169	1062.1543
1741.6135	933.6239
3026.2812	16.4394
3029.3121	45.4519
3040.4074	37.8966
3041.7557	109.6586
3046.8006	32.5305
3077.1376	36.7508
3086.8542	25.1287
3110.1652	8.4956
3114.1127	104.2882
3120.2807	46.9699
3129.5135	35.0610
3147.5870	4.9389
3160.7159	8.4795
3177.5911	10.6269

Frequency (cm^{-1})	IR Intensity (KM/Mole)
3199.7836	9.5112
3199.9964	0.7887
3206.2074	0.0668
3208.4200	9.6254
3215.9956	20.7452
3220.4168	21.7999
3224.1884	12.8433
3586.0299	169.9180
3600.1858	85.2462
3897.5331	59.2284

Threonine calculations at level of theory - M06-2X/6-311++G(d,p)

Threonine (Thr1-OH) in gas phase

Frequency (cm^{-1})	IR Intensity (KM/Mole)
69.7607	0.8004
95.7493	0.9238
178.1170	0.2669
232.3243	7.6722
256.2192	13.1838

Frequency (cm^{-1})	IR Intensity (KM/Mole)
294.6887	10.4902
305.1613	43.4032
332.9356	149.3995
384.5262	12.5979
407.9710	5.0739
489.2588	6.3584
535.1177	18.4011
614.8302	121.1891
678.1937	12.4347
778.9178	18.3746
831.4384	6.1470
867.8000	112.3445
936.4471	52.7952
985.0956	36.7438
1071.7446	13.6438
1105.1247	3.1392
1148.1572	4.6007
1163.9680	48.0973
1181.5678	257.7165

Frequency (cm⁻¹)	IR Intensity (KM/Mole)
1251.0827	59.5454
1292.2153	61.4666
1293.7527	6.9498
1338.8410	30.9140
1375.2123	22.5760
1396.2973	12.7816
1447.5295	29.3375
1452.3416	19.6033
1498.3876	2.0624
1505.8840	8.9398
1670.5938	43.5984
1872.8460	295.9451
3047.2748	29.9647
3075.6239	7.8790
3090.0689	14.2995
3154.6755	10.1293
3162.3259	8.9642
3537.0879	6.6065
3618.8209	12.5855

Frequency (cm^{-1})	IR Intensity (KM/Mole)
3819.4513	92.0462
3884.9191	37.3359

Threonine (Thr1-OH) in diethylether

Frequency (cm^{-1})	IR Intensity (KM/Mole)
51.5201	0.9204
95.6532	1.2788
179.4337	0.4928
232.3925	7.8142
254.0902	14.6099
293.6678	8.5592
314.0649	102.9451
331.4930	154.2956
383.9865	18.4555
406.6171	6.1954
489.4043	8.5514
535.4052	26.4075
610.2647	168.6344
678.9278	16.4777

Frequency (cm⁻¹)	IR Intensity (KM/Mole)
779.1106	25.1606
831.6480	8.4513
870.6199	133.0233
939.6143	77.2821
982.6733	55.5962
1069.3734	19.5671
1105.4686	5.5500
1142.7899	2.4525
1158.9934	111.5721
1180.0432	308.2078
1245.1092	81.7892
1289.3008	76.9875
1294.1868	8.7057
1341.1571	46.4990
1377.8836	24.5149
1394.9593	15.0332
1446.1456	37.9009
1452.5649	31.0221
1489.0531	3.3433

Frequency (cm⁻¹)	IR Intensity (KM/Mole)
1498.1766	11.9860
1662.2905	56.9023
1842.0045	448.4283
3055.8862	37.2927
3075.4862	11.8680
3096.0427	15.0220
3153.8151	16.3907
3160.4151	13.9385
3534.9970	9.9170
3614.0692	20.1019
3801.5934	129.7467
3874.0487	60.6780

Threonine (Thr1-OH) in methanol

Frequency (cm^{-1})	IR Intensity (KM/Mole)
48.2721	1.2212
91.3131	1.6775
176.9919	0.9877
233.5505	11.5116

Frequency (cm^{-1})	IR Intensity (KM/Mole)
251.8846	18.7852
293.9667	22.1779
297.1509	196.9748
318.3944	73.5770
384.1030	21.4593
407.6161	6.5204
489.1630	9.4472
535.6598	29.0620
606.4901	194.8190
679.3184	19.8700
778.4221	28.5112
832.4604	9.2961
871.0490	144.9424
939.3591	86.6591
982.9329	67.0434
1067.8510	25.2384
1107.4272	6.5408
1138.6139	2.7832
1156.2460	154.2539

Frequency (cm⁻¹)	IR Intensity (KM/Mole)
1179.6533	323.7461
1242.8334	94.0955
1285.1314	79.8555
1296.3582	7.3915
1341.1162	57.0160
1378.6993	28.1674
1395.0694	17.5589
1446.6008	38.9732
1453.4333	37.0308
1484.9591	3.8762
1494.6787	14.2831
1658.3274	65.3495
1827.0338	539.9586
3060.3927	41.7867
3074.2963	14.9862
3104.0202	15.9365
3151.7514	21.5688
3159.3367	17.4053
3534.3458	11.4216

Frequency (cm^{-1})	IR Intensity (KM/Mole)
3611.9144	24.1302
3794.9844	144.5046
3870.7942	77.8038

Threonine (Thr1-OH) in water

Frequency (cm^{-1})	IR Intensity (KM/Mole)
47.8562	1.2601
91.8712	1.5654
177.2036	0.7568
234.1412	12.0694
252.1445	18.3474
293.5115	31.1833
297.1864	185.8634
317.4377	79.1078
383.9557	22.0126
407.3769	6.2710
489.1152	9.6627
535.5867	29.3683
605.8396	197.9309

Frequency (cm⁻¹)	IR Intensity (KM/Mole)
679.3206	20.2343
778.2947	28.8142
832.4391	9.2926
871.0509	145.9445
939.3568	87.8792
982.9528	68.3040
1067.4060	25.7909
1107.4642	6.6930
1138.3314	3.0117
1155.9321	159.0179
1179.5370	325.3236
1242.6001	94.7497
1284.5354	80.8993
1296.2208	7.2049
1341.0346	58.1700
1378.4919	28.6854
1394.9785	17.2944
1446.1848	39.3060
1453.3737	38.0625

Frequency (cm⁻¹)	IR Intensity (KM/Mole)
1484.5796	3.9386
1494.4000	14.5147
1657.5816	66.4298
1825.4915	550.2713
3060.6612	42.1893
3074.1250	15.3952
3104.6503	16.0961
3151.4411	22.1610
3159.2707	17.8366
3534.2785	11.6044
3611.6836	24.6350
3794.3834	145.9285
3869.9487	79.8243

Deprotonated threonine (Thr1-O-) in gas phase

Frequency (cm^{-1})	IR Intensity (KM/Mole)
47.5413	6.5500
92.8766	2.0521
157.8472	0.6060

Frequency (cm^{-1})	IR Intensity (KM/Mole)
166.4759	34.8124
243.0880	5.6395
277.5747	3.0950
321.7486	0.8183
371.2821	14.0185
424.1812	6.5529
477.8683	28.5233
499.9025	51.6610
570.5033	71.0840
651.2798	42.0665
685.0577	53.3577
782.4407	65.8903
833.9120	49.7034
847.0122	98.8716
918.1914	43.4012
1034.8901	6.1064
1072.4130	40.4081
1123.9090	112.4887
1163.2762	147.0127

Frequency (cm^{-1})	IR Intensity (KM/Mole)
1173.5098	8.0699
1236.5292	156.0505
1281.3897	144.5829
1319.6714	17.5594
1334.9005	13.6425
1346.6276	24.4177
1367.5077	220.3629
1461.6253	70.8297
1472.1981	0.5841
1493.1067	6.9441
1621.6310	63.4098
1799.2408	417.0386
2621.5350	411.2857
3023.3066	39.1782
3051.1088	27.8838
3098.3092	48.9685
3112.5350	45.3113
3535.9965	4.3766
3635.0189	2.3135

Frequency (cm^{-1})	IR Intensity (KM/Mole)
3847.5672	38.9863

Deprotonated threonine (Thr1-O-) in diethylether

Frequency (cm^{-1})	IR Intensity (KM/Mole)
72.5109	15.8394
149.5145	11.4569
169.1056	65.3845
188.4200	6.4367
273.7888	10.8287
303.5534	4.7777
355.1235	1.3693
399.6604	7.8119
425.2458	2.1125
490.1240	30.5657
521.1639	42.3453
583.7681	129.9869
666.3163	60.6395
736.6439	11.0393
804.9304	51.2210

Frequency (cm^{-1})	IR Intensity (KM/Mole)
854.3346	89.2239
875.8095	82.6956
938.1032	118.6958
1057.3213	17.9410
1088.6451	15.0216
1128.9698	154.7310
1167.3100	145.7380
1196.3089	56.5597
1225.4167	479.1454
1267.4901	49.4151
1324.0789	21.6786
1340.4361	23.8291
1353.6018	69.4661
1366.1260	70.4662
1460.5413	70.9465
1485.4513	3.7178
1498.8513	4.6547
1634.6379	73.3452
1804.7968	548.9684

Frequency (cm⁻¹)	IR Intensity (KM/Mole)
2704.6417	408.8051
3040.9004	44.6600
3048.1418	43.2946
3116.0153	58.7729
3123.4670	43.8428
3531.1880	0.0673
3613.6969	5.9229
3824.9608	91.4624

Deprotonated threonine (Thr1-O-) in methanol

Frequency (cm^{-1})	IR Intensity (KM/Mole)
54.6546	10.2146
111.9668	17.0946
173.4903	42.8758
195.6880	44.2389
261.9536	9.0048
294.3973	5.6788
326.8183	8.0043
390.6736	9.1159

Frequency (cm^{-1})	IR Intensity (KM/Mole)
434.2768	1.4208
486.9197	23.7098
528.8749	46.5851
589.2771	155.6555
661.1754	60.0633
746.8862	3.3505
807.0083	76.8022
862.3629	70.7312
881.0708	81.4015
949.9899	154.8452
1051.2839	15.8906
1093.2800	22.1390
1131.0785	176.4241
1162.3427	121.1086
1192.6490	92.3365
1216.2255	583.7068
1269.6100	4.5161
1322.3419	32.3681
1343.7265	34.5754

Frequency (cm⁻¹)	IR Intensity (KM/Mole)
1356.2813	65.4940
1369.7377	57.2485
1458.6389	74.2682
1470.5533	8.7083
1488.0108	2.5123
1632.7631	82.4194
1799.2922	654.9180
2767.1962	374.7338
3035.5171	43.3362
3049.7669	52.0304
3108.1153	65.6314
3124.7525	52.1900
3532.3459	1.3635
3612.8784	10.8266
3810.1842	123.7373

Deprotonated threonine (Thr1-O-) in water

Frequency (cm^{-1})	IR Intensity (KM/Mole)
52.3620	11.7458

Frequency (cm^{-1})	IR Intensity (KM/Mole)
112.1363	16.4787
172.9970	41.8581
195.3470	46.6973
256.7929	8.9234
291.3144	6.0156
325.4775	8.6468
390.8142	9.3187
431.9762	1.5166
486.7660	24.1912
528.4686	46.6567
589.6293	158.6876
661.3618	61.0268
747.5106	2.9249
807.4315	74.6829
862.9141	69.8690
881.7587	81.8199
950.6947	159.9983
1050.5631	16.4591
1093.7527	22.8862

Frequency (cm⁻¹)	IR Intensity (KM/Mole)
1131.2932	180.0818
1161.6965	122.9542
1193.7285	92.9088
1214.8905	587.6585
1270.2270	4.3661
1322.3307	33.6862
1344.0547	32.1492
1354.9858	63.0587
1368.8633	59.9724
1458.2144	74.0148
1470.0506	9.4857
1485.6019	2.7772
1632.8620	82.6249
1799.0986	665.5585
2771.2541	373.4391
3036.2855	43.7655
3049.2878	51.8994
3108.8257	66.6615
3125.3607	52.5216

Frequency (cm^{-1})	IR Intensity (KM/Mole)
3532.1418	1.6104
3612.0520	11.2562
3808.6451	126.4783

Bortezomib calculations at level of theory - B3LYP/3-21G*

Bortezomib in gas phase

Frequency (cm^{-1})	IR Intensity (KM/Mole)
16.9519	0.5162
18.5312	0.9817
23.2171	0.1113
35.7331	0.2136
38.6271	0.5282
49.4384	0.1350
54.7058	0.3465
62.1458	0.4229
80.0917	1.1303
81.7012	0.3912
99.6077	0.2026
115.5984	0.9220

Frequency (cm⁻¹)	IR Intensity (KM/Mole)
129.8696	2.3029
151.4623	2.5861
168.2811	0.4658
177.6683	0.4584
183.6302	3.4302
206.7812	0.3653
234.8433	2.5085
244.9635	1.0859
247.0043	0.8596
274.8865	1.6978
298.2025	0.1327
319.9756	2.3062
328.3153	6.6093
354.3448	5.2757
368.0525	1.5482
388.4094	11.0186
401.6293	8.9967
406.7531	6.9920
432.2306	2.0109

Frequency (cm^{-1})	IR Intensity (KM/Mole)
432.3776	0.5401
437.3641	1.0371
447.0957	7.9420
458.7891	48.5604
473.1998	11.6509
480.3563	26.4020
496.8680	11.2437
530.1493	1.9607
565.2775	171.2124
573.5216	98.5662
594.9451	59.8168
612.1342	16.3114
639.1628	1.5535
654.8093	1.8053
661.7253	1.6158
675.3684	95.8986
693.7102	10.8428
731.0870	24.5651
743.7903	55.2633

Frequency (cm^{-1})	IR Intensity (KM/Mole)
765.0633	63.1074
779.0927	5.2720
786.8100	85.1164
793.0658	44.9075
803.6808	1.4119
816.1949	53.4233
844.3825	33.6159
845.6544	27.1457
879.9100	6.1513
887.7156	10.1195
893.4567	25.6275
917.1030	258.0035
932.0889	2.2175
944.6654	26.6500
952.8990	42.0837
971.1475	5.0993
973.5873	14.3898
981.4399	22.0301
992.0018	31.3170

Frequency (cm^{-1})	IR Intensity (KM/Mole)
1000.5234	13.2324
1019.3871	3.9753
1020.3037	10.4707
1030.9571	43.7679
1037.9634	2.4993
1049.8544	0.3189
1053.4313	2.3245
1054.3673	0.8724
1062.8053	8.6696
1068.1170	6.5921
1092.7082	25.3440
1113.2903	4.6557
1124.7161	11.8233
1127.1888	6.9675
1171.8840	45.7132
1183.8152	8.2150
1188.0133	23.9804
1205.0396	11.3596
1215.9859	12.1277

Frequency (cm⁻¹)	IR Intensity (KM/Mole)
1226.0589	0.6839
1230.2923	0.1663
1238.5501	3.6984
1243.6627	11.1161
1247.4668	6.7919
1303.5287	5.7759
1308.2115	8.7025
1311.5174	25.9973
1314.7485	4.4635
1322.1017	146.6586
1336.0201	104.5568
1359.6884	48.0789
1364.8073	82.1441
1372.6174	71.1227
1399.3340	2.5655
1409.5349	2.7396
1414.5361	36.9654
1421.2412	17.0648
1431.1012	68.1124

Frequency (cm^{-1})	IR Intensity (KM/Mole)
1448.2020	169.0783
1453.1412	25.4610
1456.7779	134.8830
1471.3741	12.6021
1486.4396	3.1774
1497.6419	6.8345
1507.2189	40.6488
1511.6613	8.5795
1527.0123	22.0583
1536.0135	2.6247
1544.6175	1.2693
1548.9078	40.7773
1561.1002	2.9993
1561.2847	6.2189
1564.4561	4.9601
1570.4934	2.6984
1592.0348	11.2294
1613.4900	6.2432
1634.4345	16.4794

Frequency (cm^{-1})	IR Intensity (KM/Mole)
1682.8668	305.9558
1694.4364	207.7079
3036.9093	13.1296
3040.3426	51.9233
3044.9024	12.5810
3061.0478	1.6807
3078.9646	11.2726
3082.7226	22.4849
3087.1099	8.2021
3090.7323	17.2407
3099.2290	5.6334
3106.7020	39.6040
3110.6468	57.5521
3132.2914	18.3527
3167.4885	0.9218
3174.9913	6.9366
3185.2439	2.2906
3195.9736	27.9016
3201.0273	6.3151

Frequency (cm^{-1})	IR Intensity (KM/Mole)
3211.1283	18.4404
3222.7271	17.7166
3227.4915	21.1288
3243.8047	0.6925
3504.7077	21.9873
3509.1754	59.7764
3568.2350	11.6231
3576.1692	18.0979

Bortezomib in diethylether

Frequency (cm^{-1})	IR Intensity (KM/Mole)
15.2994	1.4231
27.1811	0.4566
32.5732	0.9480
37.6081	0.7011
51.0557	2.3634
60.1416	0.6739
60.7049	1.8265
68.6104	1.4422

Frequency (cm^{-1})	IR Intensity (KM/Mole)
75.7722	1.0517
81.5300	0.2284
104.9093	1.2358
115.3071	1.0256
130.5575	4.8105
158.2217	2.3516
171.2841	4.9219
174.6619	0.6608
177.0268	0.3667
214.0955	1.0890
225.4199	0.4444
241.4937	0.4047
257.3578	2.3219
271.6715	5.7466
296.7190	0.1439
308.4393	3.2509
334.1830	3.2341
346.8244	11.1158
371.3447	11.0135

Frequency (cm^{-1})	IR Intensity (KM/Mole)
389.6306	8.3102
400.9431	5.3377
404.9000	17.8595
428.4520	0.7994
430.8899	0.7338
442.1214	10.1080
467.9692	36.7655
485.8596	13.5852
487.5719	23.2993
506.8974	11.7723
526.7961	15.7166
535.7161	64.9411
560.5454	14.9561
576.7543	383.2437
591.1272	38.6311
622.2053	58.2690
627.3857	2.0156
651.6876	5.9251
660.6167	1.8128

Frequency (cm^{-1})	IR Intensity (KM/Mole)
680.0987	56.6989
707.3417	24.7415
740.9432	52.5790
745.6964	45.5065
764.2841	97.9599
781.6858	10.2336
792.1650	100.7093
798.5675	45.6996
803.2991	26.5767
827.0319	102.7734
832.4163	44.8064
844.4011	14.3322
877.6729	5.6536
886.3605	4.4920
898.9249	25.6242
935.7315	28.3955
938.4058	341.0454
949.1626	12.5700
954.9935	31.0689

Frequency (cm^{-1})	IR Intensity (KM/Mole)
963.2400	4.9521
975.0299	2.9618
987.7464	4.7635
1002.2646	3.6237
1017.3077	27.2838
1021.4613	4.7228
1027.1156	121.9136
1035.6298	3.0413
1036.1956	39.7235
1041.5805	3.3373
1049.3872	6.9955
1056.1958	24.1517
1066.0737	7.3289
1072.1940	4.6030
1075.3028	15.2758
1106.3025	8.5190
1120.5885	14.4243
1130.2885	4.2984
1158.4979	99.0514

Frequency (cm^{-1})	IR Intensity (KM/Mole)
1182.0705	71.0074
1186.2058	16.5883
1201.0006	14.4886
1215.9377	13.7252
1224.8718	0.6160
1227.6934	0.2886
1235.1150	3.9792
1240.6017	4.6557
1269.5279	6.0179
1297.1399	148.3523
1301.1293	37.8129
1317.1258	34.5371
1324.8660	5.8052
1331.5347	21.5309
1349.0108	120.7722
1362.1898	91.3404
1367.3463	8.3630
1381.6532	90.6592
1398.2173	20.4644

Frequency (cm⁻¹)	IR Intensity (KM/Mole)
1404.4204	87.3928
1411.8362	41.6898
1417.3432	74.8636
1420.9260	112.1881
1438.1795	65.8801
1447.1740	63.1086
1452.4595	78.7825
1466.8668	19.4067
1488.0541	7.2466
1502.6014	31.1543
1508.4547	12.2145
1513.4041	13.9380
1521.8469	20.7139
1523.4621	20.8098
1535.6857	2.5583
1547.2880	43.5956
1554.8171	3.2762
1556.0235	6.0971
1564.8962	1.7455

Frequency (cm^{-1})	IR Intensity (KM/Mole)
1568.5198	5.6269
1587.5262	13.4058
1611.7182	7.0285
1631.0133	12.9350
1681.8297	292.9438
1687.6786	447.9935
3029.3496	33.0495
3035.5097	39.2755
3041.3610	30.1139
3053.4296	5.4539
3076.5389	33.1021
3078.2534	21.4397
3087.7473	9.9304
3094.2090	5.1557
3103.4637	77.1492
3104.6730	69.5747
3118.8051	10.0479
3136.8569	7.0155
3140.7349	14.1385

Frequency (cm⁻¹)	IR Intensity (KM/Mole)
3179.0492	8.9173
3189.0998	1.7411
3198.9631	32.8406
3209.9788	15.3454
3213.5098	23.4232
3230.4082	3.4199
3233.1300	9.8351
3247.4244	13.8473
3454.7799	21.4390
3496.5817	56.4635
3564.4669	51.7578
3566.8114	16.4153

Bortezomib in methanol

Frequency (cm⁻¹)	IR Intensity (KM/Mole)
16.5750	0.5142
22.2918	1.5329
32.8850	1.5860
36.8809	1.2026

Frequency (cm^{-1})	IR Intensity (KM/Mole)
43.4553	1.1593
51.4931	1.4196
60.4240	1.5521
67.1077	0.9544
72.8212	0.9810
78.3767	1.5883
90.0653	1.4167
115.0884	0.7852
132.2660	3.1921
156.2728	1.1427
176.1480	0.1099
181.4278	9.2363
193.2224	2.5757
207.7617	0.7569
210.7169	0.5861
238.7345	1.3736
248.4135	0.6356
253.9794	1.5186
287.8388	5.4296

Frequency (cm^{-1})	IR Intensity (KM/Mole)
300.5159	0.1460
324.4661	11.8538
336.9914	7.8510
344.3753	3.7062
355.1438	6.7338
395.6646	25.7527
404.5607	4.3245
425.3772	49.9031
427.9874	11.3104
431.1439	1.2322
463.3658	62.2768
467.4577	1.1112
472.6647	28.1117
482.8696	15.2487
520.1569	16.5752
539.4286	101.9602
561.9297	371.7962
581.9820	34.1253
612.7051	4.7599

Frequency (cm^{-1})	IR Intensity (KM/Mole)
619.4906	18.6671
632.1486	33.2717
647.1325	3.3892
659.9015	0.9728
675.0080	104.2318
691.4753	49.7336
734.9825	31.7498
742.6997	87.2938
769.6054	87.5471
774.4885	10.0239
781.1162	119.9330
792.1392	93.8269
794.8168	1.9550
813.0097	5.3643
823.8290	65.6929
843.1246	10.4076
877.7038	4.7033
888.9464	2.3615
895.1756	21.3150

Frequency (cm^{-1})	IR Intensity (KM/Mole)
900.3747	18.6422
933.2436	399.7644
947.1542	50.9786
953.3443	20.3575
968.6770	5.2416
975.6519	1.9308
987.7266	4.0297
999.4830	4.9927
1001.0909	1.2597
1023.4894	52.9012
1025.3405	10.7684
1027.7871	118.2306
1035.2358	2.9730
1038.4368	0.9853
1047.4063	0.4971
1051.4237	13.4224
1062.3018	35.1736
1065.4146	7.9135
1074.6941	23.2280

Frequency (cm⁻¹)	IR Intensity (KM/Mole)
1100.3047	3.2435
1119.3640	24.9898
1128.8723	6.6817
1148.2282	3.1098
1183.5320	10.7518
1188.2738	30.5265
1202.7948	96.7732
1216.0877	14.3275
1224.1471	0.9726
1227.3720	0.1068
1233.4169	5.6989
1239.8783	4.9525
1255.2839	7.7932
1300.2363	15.8446
1304.3577	119.6521
1318.7858	9.3066
1325.6251	24.6962
1330.1022	14.3176
1347.8079	120.8233

Frequency (cm^{-1})	IR Intensity (KM/Mole)
1365.9760	129.5562
1369.2094	5.4392
1381.5373	99.5082
1397.5435	12.6053
1408.4125	15.0412
1417.2606	99.3769
1419.3935	136.1711
1429.8799	102.4939
1431.6744	29.7998
1448.2183	89.2094
1451.9795	141.8640
1466.3452	29.8101
1494.3655	2.6280
1495.2943	49.5198
1502.3084	38.3030
1510.6206	23.2392
1512.4625	4.4206
1521.1301	35.7001
1534.6584	3.2018

Frequency (cm⁻¹)	IR Intensity (KM/Mole)
1546.5210	50.8411
1551.9799	4.2676
1553.6537	6.6521
1562.3117	2.0826
1565.9838	10.0902
1586.5798	12.8148
1610.1258	8.4595
1629.4180	19.0953
1669.6792	496.3405
1685.9915	428.5793
3032.1187	26.4831
3035.5605	53.6430
3041.3821	32.8723
3051.2480	12.7546
3077.0906	37.2177
3085.7406	23.7068
3093.7145	8.8903
3098.7735	14.9372
3101.7785	96.9135

Frequency (cm⁻¹)	IR Intensity (KM/Mole)
3103.9836	81.2678
3110.2286	5.5343
3140.4569	7.7892
3151.5102	7.5402
3182.1757	9.7149
3190.5492	2.2872
3200.1690	37.5378
3208.6039	16.2833
3214.5757	25.0077
3217.1067	16.6285
3233.7678	10.7666
3234.9398	24.9305
3487.7252	46.8483
3492.0941	91.4431
3562.8760	50.7149
3579.3323	48.5395

Bortezomib in water

Frequency (cm^{-1})	IR Intensity (KM/Mole)
13.5409	1.8200
28.9484	0.6495
31.9877	1.0003
37.9856	1.2085
51.0928	2.8216
60.0448	1.6082
60.8562	0.2774
69.8727	1.8760
75.1009	1.8543
81.7398	0.1101
103.9059	1.3452
113.4384	1.3822
128.6571	5.2204
157.2598	2.3906
171.6683	4.7452
174.2147	1.7819
176.7390	0.2859
212.7987	1.1372

Frequency (cm^{-1})	IR Intensity (KM/Mole)
224.1140	0.4337
241.7713	0.7255
256.5969	2.9392
270.4576	6.3383
296.4132	0.3431
308.6180	3.4595
332.9355	4.0882
344.7413	14.1584
370.2761	13.0805
387.2202	8.6937
399.1422	18.0502
401.7577	12.2545
427.8015	1.2851
430.9089	0.6947
441.4939	12.5144
466.7256	48.3636
483.6445	30.9296
486.7901	18.0839
506.0097	4.4341

Frequency (cm^{-1})	IR Intensity (KM/Mole)
515.7132	23.5814
534.4832	86.0451
560.2319	18.4544
570.3285	434.2890
590.8600	41.6885
622.2401	51.1044
628.1431	3.2607
651.5743	6.6970
659.8719	1.7996
679.1834	67.1794
705.4319	34.2150
741.4248	48.1292
745.0269	70.7212
763.2364	108.5750
781.5246	15.9538
791.7915	116.7434
795.0728	60.9740
801.9160	25.7720
824.4789	114.3853

Frequency (cm⁻¹)	IR Intensity (KM/Mole)
830.9480	36.2173
843.4861	14.4258
877.3046	5.5211
888.3441	3.7243
899.0168	26.1686
936.5368	21.9691
940.1505	408.3228
948.6697	32.3091
954.5225	30.4129
965.1168	4.0811
974.4127	2.5931
986.9260	5.5227
1001.7851	4.2883
1015.3119	27.2338
1023.6888	125.6804
1024.5501	6.2601
1034.8698	3.4187
1035.6976	43.8172
1041.9541	6.7000

Frequency (cm^{-1})	IR Intensity (KM/Mole)
1048.8789	8.3316
1056.1094	24.3622
1065.1782	8.7043
1073.8419	21.1559
1078.9545	5.1665
1105.2134	10.4497
1119.3111	19.1338
1129.1942	6.5281
1158.9162	91.0684
1181.7121	84.8827
1184.1458	22.7262
1200.4819	19.3740
1214.6909	14.8364
1223.4243	0.6514
1226.3206	0.5400
1234.0107	4.6015
1238.5646	3.6668
1264.8430	7.3870
1297.6791	139.1353

Frequency (cm⁻¹)	IR Intensity (KM/Mole)
1300.3370	73.7785
1317.3026	34.7368
1324.3319	7.5043
1330.6108	17.5911
1348.1933	164.1653
1360.9591	87.7907
1367.0293	7.4412
1381.1173	113.5645
1396.4137	15.4356
1405.0258	95.5963
1411.6275	56.4524
1417.2199	74.0238
1420.9973	139.1034
1438.2515	77.3969
1445.5736	81.3607
1450.8048	93.7939
1464.8029	19.9639
1488.0244	7.3817
1501.0749	40.3879

Frequency (cm^{-1})	IR Intensity (KM/Mole)
1507.2300	10.6994
1512.2897	15.7212
1519.4772	36.0050
1521.0470	14.5385
1533.1729	3.0706
1546.0397	47.0751
1551.0277	3.7384
1553.2708	6.7069
1560.8188	2.1729
1569.3971	7.1035
1583.3408	14.9002
1609.9779	7.8162
1628.9075	14.5762
1675.4229	344.7838
1680.8674	528.6466
3028.4248	43.1602
3034.5371	47.0183
3040.9460	32.4861
3050.7666	9.1060

Frequency (cm⁻¹)	IR Intensity (KM/Mole)
3075.2337	40.7124
3080.1446	27.1593
3088.4733	9.6895
3093.3598	7.1338
3101.1120	86.6026
3102.8135	96.1549
3122.2358	9.9265
3138.7343	11.5970
3142.1969	13.7088
3182.2275	9.3782
3190.4956	2.2395
3200.0727	37.4780
3213.4784	18.0733
3214.4601	24.9714
3228.5222	5.1853
3232.6094	9.4086
3251.8473	14.7310
3455.7703	30.7819
3490.9121	74.4111

Frequency (cm⁻¹)	IR Intensity (KM/Mole)
3563.8427	35.9625
3565.6664	63.3934

Bortezomib (de1) in gas phase

Frequency (cm⁻¹)	IR Intensity (KM/Mole)
17.7116	0.4003
30.4984	0.4066
35.3900	0.1940
51.9536	0.0883
56.4115	0.3808
62.6994	0.2681
69.4322	1.9334
86.6423	0.6953
90.9563	0.8299
99.5050	2.4323
106.9283	0.6175
127.2196	0.7456
133.4803	1.6645
155.0012	1.8630

Frequency (cm⁻¹)	IR Intensity (KM/Mole)
172.2420	23.8135
179.0058	3.5319
185.0144	10.7163
215.0567	3.6678
227.6137	0.9235
244.0339	1.4127
251.4848	6.8206
268.6555	8.2561
309.8383	1.2781
319.7258	5.1765
325.5026	14.2651
341.8893	12.4080
361.6165	13.5224
392.8264	14.4722
407.8516	3.3357
408.9305	8.5706
431.8099	1.5611
438.7120	0.7182
441.1636	2.9578

Frequency (cm^{-1})	IR Intensity (KM/Mole)
464.8673	28.0454
477.5669	3.2597
495.7400	9.2578
522.6470	23.7409
535.2166	29.5357
566.6964	5.3903
578.1096	89.0368
593.9437	37.3702
629.1265	5.1963
652.0737	3.4126
659.8307	5.9082
671.7122	25.5206
685.3060	22.9831
705.7991	57.0434
739.6928	52.6536
751.8554	33.3655
762.9992	6.0193
776.0306	10.5981
795.4694	53.6250

Frequency (cm⁻¹)	IR Intensity (KM/Mole)
798.7911	12.2298
823.9039	5.3509
839.3256	17.6923
847.8049	30.8622
863.1041	15.8974
883.5430	17.4760
895.7080	121.3611
903.1647	30.0883
950.9634	10.1352
956.2950	8.9087
959.9650	14.5598
966.8952	18.5640
978.1630	8.2028
993.4496	3.6454
1001.0549	10.2864
1011.1806	9.1408
1012.0974	177.2868
1018.2865	2.6275
1026.8924	19.9097

Frequency (cm^{-1})	IR Intensity (KM/Mole)
1036.8133	2.9194
1037.8290	30.9757
1048.6644	38.6389
1064.2900	1.8798
1069.6378	6.1044
1095.6611	41.7315
1109.6547	12.7174
1118.8132	8.6503
1120.5424	0.5189
1166.5408	4.9896
1181.8954	8.4139
1184.0058	24.0832
1196.9793	41.8221
1206.7834	26.3848
1209.5746	10.3103
1219.9600	25.1403
1226.9303	0.1148
1231.1267	1.5452
1236.5157	1.7240

Frequency (cm^{-1})	IR Intensity (KM/Mole)
1244.7700	5.4968
1261.9415	51.9669
1299.3858	14.1926
1302.4241	78.3462
1320.4005	13.8078
1327.3988	24.2485
1345.7429	3.7749
1349.9428	11.3081
1358.8698	98.4587
1368.9030	8.4581
1390.2246	102.8256
1401.7343	13.4356
1410.5446	60.9671
1417.9044	3.7955
1420.7636	1.2141
1443.4201	3.0116
1447.6269	113.1474
1470.9976	56.4308
1480.1594	12.5057

Frequency (cm^{-1})	IR Intensity (KM/Mole)
1499.1503	92.8380
1506.1766	18.4679
1514.8078	10.3326
1529.3141	21.1551
1531.5119	5.8200
1536.5181	1.4648
1548.9672	35.6081
1554.9429	54.8497
1560.6156	5.0382
1565.6527	34.3268
1571.2526	39.2302
1579.8586	79.1674
1596.2398	23.7197
1613.7434	5.5208
1634.3292	13.5739
1676.2497	387.6941
1680.1483	168.9403
2985.2891	57.4015
2992.6718	334.0938

Frequency (cm^{-1})	IR Intensity (KM/Mole)
3021.5499	19.5841
3026.8844	77.5582
3040.5712	41.1630
3046.4436	23.2057
3059.8434	11.4551
3073.1083	8.8490
3082.1694	7.5444
3094.9939	43.7078
3101.8769	59.0284
3118.0901	0.8563
3119.3709	15.4746
3147.7014	15.4098
3170.4083	4.9620
3181.4266	5.2950
3191.8040	26.2471
3191.8364	43.1257
3204.2257	9.6179
3212.5736	28.2729
3230.3957	6.0396

Frequency (cm⁻¹)	IR Intensity (KM/Mole)
3286.4051	207.6874
3473.8489	6.8002
3509.8486	4.5439

Bortezomib (de1) in diethylether

Frequency (cm^{-1})	IR Intensity (KM/Mole)
19.6112	0.8475
36.6694	0.8401
41.9837	0.4323
60.0735	0.5852
66.9932	1.2575
71.5412	1.8463
73.3198	0.5692
87.8582	0.9603
93.5585	1.1670
99.5370	5.2479
109.7771	0.6978
128.1109	0.3883
132.7044	4.1220

Frequency (cm^{-1})	IR Intensity (KM/Mole)
151.6897	8.9953
179.0500	9.1733
180.1619	17.7428
185.9013	23.8958
216.1858	1.5853
228.0167	0.2897
240.1726	1.4625
251.2764	8.4232
269.1529	13.7754
316.8039	5.5422
323.8596	23.2688
327.5405	6.3231
351.8582	13.4867
364.1338	17.3950
385.7606	15.8383
406.5662	11.7987
413.7857	2.1152
432.5532	1.4434
437.7699	3.2376

Frequency (cm^{-1})	IR Intensity (KM/Mole)
453.3878	1.7409
463.5057	41.4709
475.8479	15.4049
492.0839	13.8532
521.8275	40.6142
530.7103	42.1601
560.1913	105.1680
566.7529	29.5522
591.7830	30.8710
630.7734	3.6383
652.7515	5.8107
660.1267	4.2020
672.4061	36.9707
683.1813	32.5354
705.5945	77.6828
741.3437	66.6286
751.0629	43.5449
765.1749	5.6297
778.4101	22.7366

Frequency (cm⁻¹)	IR Intensity (KM/Mole)
790.2442	76.3280
797.0325	9.2604
821.5319	4.2511
837.0683	28.5732
845.3672	41.3694
861.3818	85.0726
876.0577	133.4696
888.3200	4.0495
904.6493	30.4827
951.7058	12.4974
957.5463	29.5666
961.0703	18.2285
968.4285	6.0421
980.2066	8.1320
990.3665	40.8174
996.0929	125.6935
1001.9821	66.6483
1015.9944	6.4780
1021.0706	2.8196

Frequency (cm^{-1})	IR Intensity (KM/Mole)
1025.6520	32.0551
1035.9421	2.5764
1038.3806	37.2659
1047.5643	35.6481
1054.3336	10.4594
1066.1677	6.4500
1094.1939	52.6756
1110.1318	19.9160
1117.7170	10.8167
1120.5242	0.7756
1161.6392	3.2644
1175.9502	6.1864
1179.9335	35.4544
1190.5174	61.1223
1203.0968	24.9746
1207.3220	45.3754
1219.3855	37.1531
1225.1930	0.3517
1229.9679	1.1435

Frequency (cm^{-1})	IR Intensity (KM/Mole)
1235.6033	2.9025
1240.8810	3.7918
1257.7560	76.8581
1300.9397	119.3347
1302.4882	19.2110
1318.8598	15.1307
1329.3647	43.3109
1344.7644	4.5580
1349.9498	36.8584
1358.3884	81.5655
1365.7348	10.2434
1393.2442	165.6780
1401.9765	26.2504
1411.9732	63.2962
1415.4435	7.1451
1420.5228	1.6863
1444.8292	3.5070
1448.9645	150.4819
1467.3232	48.3563

Frequency (cm^{-1})	IR Intensity (KM/Mole)
1475.0753	10.7356
1501.4404	111.5758
1506.8932	19.9813
1515.5483	16.9625
1525.9298	24.5559
1530.2176	7.7981
1542.0714	2.0834
1547.0277	41.1863
1553.7698	57.8036
1556.2394	24.1315
1559.2255	138.0380
1568.7090	19.6488
1576.6084	38.2090
1592.4616	22.0829
1611.8664	7.9868
1630.8380	13.9331
1669.0207	558.2668
1671.7710	212.1309
2998.0554	64.6494

Frequency (cm^{-1})	IR Intensity (KM/Mole)
3025.8657	22.3227
3031.1103	101.3146
3050.1826	161.2454
3053.6916	167.3707
3054.4806	40.3928
3058.3820	18.8303
3074.4998	16.6693
3083.6182	0.5649
3096.0022	78.7306
3102.6889	78.5353
3119.7072	1.4358
3123.5300	9.6583
3148.0993	24.0136
3178.5062	7.2406
3188.5825	1.9263
3197.4853	30.7083
3201.7383	33.7279
3210.3950	14.3055
3218.6207	19.0785

Frequency (cm⁻¹)	IR Intensity (KM/Mole)
3232.9878	5.6831
3324.9299	230.3039
3472.2992	19.3314
3506.8856	1.6750

Bortezomib (de1) in methanol

Frequency (cm⁻¹)	IR Intensity (KM/Mole)
17.1696	0.7023
21.6494	0.8480
26.6550	0.1953
34.9344	0.5602
39.1676	0.6459
47.4958	0.3847
60.7682	0.9146
65.7908	0.1188
79.5871	1.6464
94.6039	0.4743
107.1110	1.4188
127.0500	5.0637

Frequency (cm^{-1})	IR Intensity (KM/Mole)
131.7401	3.2765
146.2317	11.8206
169.3398	0.6003
174.6519	9.2924
189.8593	12.3072
214.3499	2.4717
230.4263	4.3726
238.0732	4.7167
250.5952	23.3960
263.2071	1.5319
312.7281	12.4891
322.3267	2.2112
338.0881	16.4432
344.6236	14.3452
363.7291	4.1960
387.9140	38.6109
399.8965	11.0955
404.3712	13.9477
410.7476	4.3285

Frequency (cm^{-1})	IR Intensity (KM/Mole)
430.9221	0.1004
435.6432	1.6169
470.0098	67.7834
473.5312	11.9397
489.5018	74.2034
522.2858	69.7896
542.2351	55.6007
556.3423	22.0805
571.2054	61.8638
596.8020	62.0499
635.4083	6.6420
644.1680	6.6419
657.3209	3.8425
659.8629	0.4522
688.9304	96.6966
718.9566	16.2339
739.6177	82.2168
751.4316	27.0254
767.0075	35.8635

Frequency (cm⁻¹)	IR Intensity (KM/Mole)
782.2044	78.3669
785.9155	9.9925
791.4813	11.8431
819.3468	13.6570
835.8099	17.0960
838.8211	47.3765
853.0162	39.4679
884.8027	3.7033
898.9274	42.0386
923.5689	4.4025
933.8286	94.1143
955.2346	95.5005
958.6760	13.4820
979.9518	1.4168
985.2478	28.7620
990.6211	0.6189
995.9869	154.3822
997.6371	58.9051
1019.6987	3.2841

Frequency (cm^{-1})	IR Intensity (KM/Mole)
1031.7835	70.3766
1033.1540	5.0514
1044.7824	2.2208
1046.3851	1.9117
1052.1337	5.1863
1052.9596	2.6091
1065.3905	6.0397
1070.0120	61.8204
1086.4967	24.6909
1110.0782	40.3137
1113.7262	26.1747
1118.0442	4.3918
1169.8859	16.4000
1173.5076	15.2011
1187.3216	55.4264
1190.2364	55.3052
1203.0119	4.8176
1214.1953	14.7131
1222.1964	18.6984

Frequency (cm⁻¹)	IR Intensity (KM/Mole)
1223.2935	0.3641
1228.7201	0.3201
1236.4878	4.0956
1251.8362	131.2516
1292.3245	19.2366
1297.1843	22.1055
1320.4808	1.7976
1322.1381	13.0082
1327.3614	52.6200
1343.1302	43.3887
1363.9965	5.9613
1379.5513	255.0515
1398.0495	152.5512
1399.7543	6.1043
1403.2148	15.3717
1413.3741	43.1487
1418.8582	21.0548
1432.1915	53.9150
1442.3096	143.2130

Frequency (cm⁻¹)	IR Intensity (KM/Mole)
1445.5275	4.6252
1469.9482	10.1824
1484.6414	18.3882
1496.0487	7.6475
1509.1209	10.4905
1516.0322	6.0501
1518.2851	46.1070
1544.9133	53.2932
1546.5508	1.6000
1550.2905	4.6834
1555.5960	17.5819
1563.9801	51.0684
1565.5474	22.0195
1584.0907	255.2093
1597.8676	27.1781
1609.2056	6.7263
1628.4837	22.5921
1656.4074	552.6594
1670.2766	510.6730

Frequency (cm^{-1})	IR Intensity (KM/Mole)
3013.4155	31.5528
3021.8601	79.7425
3027.2210	59.1283
3031.8009	70.9361
3053.7804	7.4692
3071.2904	20.9614
3080.7333	21.5421
3083.4124	30.6231
3094.1265	71.6839
3096.9035	123.5414
3120.2446	7.2168
3136.1836	10.7716
3183.6959	5.0928
3190.1134	4.2095
3199.2763	39.2077
3208.1854	4.0137
3212.4552	21.8896
3219.8543	501.1440
3223.2639	19.5986

Frequency (cm⁻¹)	IR Intensity (KM/Mole)
3226.7239	31.4251
3250.3641	1.9070
3264.1845	7.5968
3487.1556	54.5187
3518.2716	1.8206

Bortezomib (de1) in water

Frequency (cm⁻¹)	IR Intensity (KM/Mole)
17.1541	0.7496
21.4080	0.7604
27.6700	0.1813
33.7053	0.4615
34.9478	0.6928
46.9297	0.3886
58.7912	1.3147
65.7103	0.1209
77.7179	1.4128
93.5434	0.5141
106.9140	1.4471

Frequency (cm^{-1})	IR Intensity (KM/Mole)
127.8356	4.9491
131.9298	3.3565
145.3117	12.0532
169.2890	0.5533
174.6897	9.4861
188.9723	11.6782
214.7666	2.8820
229.7472	4.5474
240.1801	9.6896
251.7499	19.2531
263.2327	1.4267
312.5319	12.6929
321.8899	2.2563
337.6904	14.6519
344.6914	16.3434
361.7033	4.4505
388.0575	37.4772
399.8371	11.4317
403.3640	15.4482

Frequency (cm^{-1})	IR Intensity (KM/Mole)
410.7649	4.3262
430.7871	0.1099
435.4943	1.6895
470.1472	69.9258
473.4418	12.0511
489.7303	74.0350
522.2251	72.5939
542.0161	55.3519
556.4584	22.4263
570.9192	60.7904
596.5466	63.4353
634.8229	6.6913
644.3155	6.6342
657.1775	4.0815
659.7634	0.4105
687.4097	100.6204
718.9459	16.3230
739.9497	82.1472
751.4275	29.0064

Frequency (cm^{-1})	IR Intensity (KM/Mole)
766.7170	36.0027
781.6292	80.6446
785.8711	7.9962
791.5494	12.6402
819.1151	13.9426
835.5356	17.7698
838.8237	47.0286
852.9700	39.7759
885.3089	3.5072
898.7177	41.9801
923.6966	4.4974
931.5487	110.5140
953.8520	83.4815
959.0461	12.7760
979.5957	1.2887
985.1326	28.3950
989.8070	1.0536
995.7436	157.3348
997.5093	60.5872

Frequency (cm^{-1})	IR Intensity (KM/Mole)
1020.1628	3.3557
1031.7405	72.4732
1033.1572	3.9678
1044.9624	2.1132
1046.4814	1.8566
1052.2429	5.9969
1053.1376	1.6782
1065.3696	6.4089
1070.0875	62.0954
1086.2609	25.9017
1110.0051	39.7555
1113.6434	26.7042
1117.8362	5.1220
1169.7992	16.9335
1173.4398	15.6122
1187.2874	56.7936
1190.4820	55.9068
1202.8529	4.9558
1214.2578	14.8734

Frequency (cm^{-1})	IR Intensity (KM/Mole)
1221.8716	19.2409
1223.1863	0.3802
1228.6066	0.3353
1236.2504	4.1519
1251.7593	132.2559
1291.9382	17.6561
1296.8372	24.5002
1320.3583	2.2581
1321.9557	13.0548
1327.3764	54.2504
1343.2004	45.5712
1363.4029	5.8152
1379.1741	257.8830
1397.8012	155.8655
1398.8846	3.2419
1402.6660	19.7897
1412.8272	42.1407
1418.8117	21.1476
1432.1744	54.5489

Frequency (cm⁻¹)	IR Intensity (KM/Mole)
1442.1745	144.4772
1445.4325	5.1030
1469.1424	10.0996
1484.8114	18.6728
1496.0859	7.7215
1509.0940	10.6662
1515.9507	6.2747
1518.4113	46.8367
1544.8510	54.4417
1546.7784	1.8705
1550.5788	4.1928
1554.9536	17.9272
1562.8528	48.9280
1565.5466	22.3978
1583.2121	263.7644
1597.0953	28.3293
1609.0792	6.9757
1628.2781	22.9778
1655.8983	555.0645

Frequency (cm^{-1})	IR Intensity (KM/Mole)
1669.7971	516.8038
3014.2676	31.0464
3022.1400	76.6165
3027.5667	61.1021
3032.2513	72.6488
3053.2273	6.4686
3071.0777	21.7658
3080.9082	23.2084
3083.6675	30.7416
3093.7174	69.3777
3096.6953	130.9746
3120.3835	7.1181
3136.3979	11.0319
3184.3172	5.0394
3190.4108	4.3603
3199.6507	39.3464
3208.7294	3.8571
3212.7418	21.6487
3222.5668	275.2475

Frequency (cm⁻¹)	IR Intensity (KM/Mole)
3223.7439	244.2437
3227.1754	31.3156
3250.1909	1.9830
3262.4276	8.5595
3487.1914	54.8501
3518.9352	1.7022

Bortezomib (de2) in gas phase

Frequency (cm^{-1})	IR Intensity (KM/Mole)
16.2953	0.5853
18.5562	0.0700
29.0349	0.7413
36.4242	0.8557
41.1927	0.4852
47.9393	2.1852
54.7484	1.0575
63.2415	0.6828
84.3468	0.4225
91.0639	0.3041

Frequency (cm^{-1})	IR Intensity (KM/Mole)
102.6805	0.7676
124.9670	2.9948
150.2909	2.0333
159.7369	3.1241
167.4687	0.4132
183.8219	4.1901
191.4202	6.0720
217.0072	0.9824
235.0608	5.4768
249.8989	0.5450
252.7102	2.0164
272.2068	4.3779
318.2736	0.4154
329.9957	8.4023
338.5929	0.6708
360.9363	2.4110
368.3584	2.9200
393.1574	6.6877
400.0045	17.1264

Frequency (cm⁻¹)	IR Intensity (KM/Mole)
413.8497	9.3535
434.2736	0.6976
441.5205	5.1681
449.8866	1.1886
460.8821	71.8367
471.4344	6.6993
481.9936	22.2029
499.0728	8.4804
522.4849	8.9043
552.5712	102.5276
581.7878	11.1150
607.0478	27.3075
613.6527	7.8578
646.7680	15.1320
654.3021	4.2243
660.3576	11.9588
665.1946	81.3604
700.0419	14.5578
730.4018	8.9567

Frequency (cm^{-1})	IR Intensity (KM/Mole)
743.0778	43.2093
747.1147	23.1972
781.6353	47.9019
785.8653	18.5846
804.7259	6.5796
808.7128	3.3964
827.4908	0.6715
841.7399	37.7099
878.2649	0.9397
886.7861	5.3721
897.1017	20.9162
924.7416	97.4503
925.6006	22.1260
940.4156	65.1011
946.0288	78.0396
969.4670	32.5108
971.3766	63.5653
980.0014	3.9712
988.0670	5.2039

Frequency (cm^{-1})	IR Intensity (KM/Mole)
998.3186	2.6750
1011.8506	0.7354
1018.7984	23.7502
1029.7591	27.3077
1036.1910	6.8395
1041.7838	2.0722
1050.2869	1.2032
1054.4294	2.3650
1067.8093	5.4979
1082.8307	1.2698
1098.2208	44.8907
1109.8968	5.9608
1114.1260	15.5258
1122.9268	21.0832
1131.7164	25.9209
1174.5613	38.1783
1182.8106	13.1640
1189.5465	15.7503
1206.1452	9.8831

Frequency (cm^{-1})	IR Intensity (KM/Mole)
1219.3797	12.5629
1224.0544	0.6963
1227.0790	4.4367
1228.5018	1.7497
1240.7493	8.1444
1247.8705	10.4907
1299.4448	3.7941
1310.2015	3.8635
1311.4420	2.8165
1321.3055	38.4627
1329.0835	52.4985
1343.7585	23.7392
1356.6373	66.4396
1366.8871	2.6587
1380.3200	81.1427
1405.0474	5.1844
1409.1901	1.8566
1415.5550	30.1227
1419.5895	22.9926

Frequency (cm⁻¹)	IR Intensity (KM/Mole)
1445.1236	4.4499
1464.5654	104.2590
1481.6265	45.6277
1484.5198	7.3229
1486.0767	8.6505
1498.9227	18.9554
1509.3925	10.1845
1521.3790	39.7365
1535.8780	3.7068
1543.2343	9.4123
1545.6691	69.4039
1559.3915	2.4237
1563.1089	1.9048
1563.7749	8.2810
1574.0247	2.1689
1605.4235	15.1725
1610.4096	19.9376
1632.8039	48.8624
1655.3816	483.9797

Frequency (cm^{-1})	IR Intensity (KM/Mole)
1674.4275	35.1812
1683.8517	287.0606
2954.5561	95.0243
3021.4601	30.9953
3025.8367	76.0102
3040.5245	2.8013
3061.2852	31.6033
3067.4087	12.5215
3083.2340	23.7705
3084.4880	23.1884
3085.2393	33.0941
3096.6296	81.5764
3100.6366	12.0394
3130.4617	11.9938
3166.7698	1.2049
3167.8230	4.6433
3176.8318	10.8166
3187.3794	45.1986
3191.0727	16.5150

Frequency (cm^{-1})	IR Intensity (KM/Mole)
3200.6053	2.2848
3206.4876	75.4825
3218.7561	24.1560
3244.8241	1.2712
3372.1443	294.4133
3515.2364	27.8425
3546.5751	3.1498

Bortezomib (de2) in diethylether

Frequency (cm^{-1})	IR Intensity (KM/Mole)
17.5246	0.9602
22.4858	0.0667
30.5962	1.4402
34.4526	0.3444
39.8223	1.4080
49.9295	1.3663
57.8132	2.0143
66.7033	2.4180
81.9245	0.3141

Frequency (cm^{-1})	IR Intensity (KM/Mole)
82.6616	0.3184
108.5001	0.1401
129.4458	3.3310
147.4789	3.5781
158.5485	4.4129
166.0295	0.6981
179.6514	6.5014
192.0753	4.2235
212.2334	1.5725
235.8260	5.4164
248.2986	2.6423
249.6958	2.1796
270.0072	4.8306
321.0179	1.0962
334.0106	11.3202
344.3786	1.1676
363.3323	0.6638
370.0157	9.0520
390.3686	18.2854

Frequency (cm^{-1})	IR Intensity (KM/Mole)
399.7340	15.9045
411.7299	9.9387
432.7431	0.8278
438.6640	2.5701
448.9421	4.7138
460.1175	107.3390
472.0555	7.2139
485.7744	32.8688
495.9878	15.8245
524.5419	11.6075
544.8736	133.1934
579.2833	13.4423
607.6141	26.4104
613.0500	15.6464
648.6980	25.7492
655.4242	1.7728
659.8561	5.6272
665.2402	121.0141
701.1389	21.5508

Frequency (cm^{-1})	IR Intensity (KM/Mole)
733.2747	15.1139
746.8892	50.3247
751.8283	26.9749
782.7038	77.5246
786.5648	19.1914
802.1339	6.1810
807.3564	6.5261
829.2182	0.9483
840.4957	51.5692
877.9237	27.2883
890.2297	43.5074
894.5761	34.7971
900.1175	74.9259
927.2439	3.5404
934.3921	148.0623
946.4223	33.9143
973.4348	102.3117
976.2478	28.2254
980.4651	3.0335

Frequency (cm⁻¹)	IR Intensity (KM/Mole)
986.8053	11.0780
995.0186	6.6997
1019.9894	16.8717
1024.5981	1.0293
1033.1175	55.3651
1034.7823	4.1080
1046.1673	0.8664
1050.5516	1.5743
1055.3063	2.9881
1065.8051	9.4371
1079.0145	4.0052
1097.6945	48.6810
1109.3779	10.5865
1117.0257	26.4620
1121.0616	24.3717
1139.0587	42.6563
1174.1943	33.5960
1184.5377	22.5170
1189.1046	37.5210

Frequency (cm^{-1})	IR Intensity (KM/Mole)
1206.6819	16.1186
1221.6694	7.1320
1221.9794	16.8496
1227.3413	0.1999
1231.5518	4.9209
1235.7523	10.4922
1245.7741	9.4263
1300.6273	2.0812
1307.5552	3.1788
1322.4226	5.8793
1324.0394	48.1991
1330.3433	97.5871
1342.4974	42.7361
1354.8363	64.4749
1366.1406	0.7699
1384.5449	119.1803
1404.7652	5.6160
1409.9812	22.9487
1414.2820	33.6714

Frequency (cm^{-1})	IR Intensity (KM/Mole)
1420.9450	36.3488
1446.0901	5.9572
1465.1033	159.9281
1471.5476	66.7542
1474.9488	13.4840
1495.4465	3.0019
1501.3409	24.3955
1506.9146	22.4939
1517.3275	39.6370
1530.7693	6.7807
1543.3993	59.4949
1543.7575	29.9177
1553.8873	3.5400
1558.1869	5.2580
1566.5266	2.9244
1567.6246	12.3923
1604.7825	14.5490
1608.6905	33.4982
1629.7748	93.6077

Frequency (cm^{-1})	IR Intensity (KM/Mole)
1643.7510	662.5351
1658.3083	19.1555
1676.7718	369.5276
2988.2278	102.2280
3025.9156	27.3099
3030.2522	77.5167
3041.5352	2.2376
3064.5154	33.6865
3072.8093	46.0416
3084.6923	9.9075
3090.2149	12.1760
3090.7471	68.3627
3093.1954	25.9124
3096.3999	118.8671
3134.5962	10.8693
3168.4856	1.7735
3175.9162	8.5181
3184.2855	5.5925
3194.0798	42.9915

Frequency (cm^{-1})	IR Intensity (KM/Mole)
3204.3749	7.7469
3208.4658	10.1180
3215.7054	55.6100
3225.3654	29.9440
3245.9928	1.7626
3401.0658	306.9888
3510.0358	40.0712
3542.7644	1.6157

Bortezomib (de2) in methanol

Frequency (cm^{-1})	IR Intensity (KM/Mole)
22.0083	0.3732
25.2597	1.7799
31.0271	0.7316
36.5439	1.8753
41.3278	0.2457
54.8085	0.5400
60.4975	1.8215
63.9451	3.1580

Frequency (cm^{-1})	IR Intensity (KM/Mole)
73.5347	0.2853
92.6219	0.1599
95.8198	2.1781
121.8325	4.6784
132.7807	1.8242
155.8438	2.0722
180.9528	4.1745
188.4247	5.7059
200.1489	16.2919
211.9365	1.6202
219.0196	0.7070
246.4173	2.8300
248.3214	2.3821
256.4045	2.9426
290.5421	9.0603
317.2507	4.5759
329.2068	9.7657
345.5620	2.3909
361.8784	2.9230

Frequency (cm^{-1})	IR Intensity (KM/Mole)
364.8352	8.4185
393.5051	27.9263
409.4650	10.9810
430.2525	14.7769
433.0597	2.1163
433.5921	11.1768
446.4829	148.3178
479.6307	19.3536
481.7866	21.0091
512.9461	27.3935
528.0917	81.8733
554.9150	57.9204
585.3604	3.1526
596.8541	65.6572
619.8876	2.5585
636.5776	15.0358
649.4420	1.8817
659.9445	1.2735
666.3514	98.5845

Frequency (cm^{-1})	IR Intensity (KM/Mole)
698.3944	37.9342
735.7485	7.7244
743.6549	72.0902
752.9877	24.1273
773.5135	88.5158
786.4646	7.4741
796.3008	7.9532
813.1766	4.6857
822.5495	20.5308
829.5423	15.3469
864.6149	178.2148
880.6997	10.5903
890.8555	3.5306
896.1645	27.3913
912.7131	18.2325
928.7870	159.9104
953.1242	24.3949
966.7211	115.7343
970.9019	7.6735

Frequency (cm^{-1})	IR Intensity (KM/Mole)
981.3814	6.1769
987.2976	4.3095
996.2138	6.2501
1007.0249	0.5626
1022.9004	41.2407
1026.6611	4.3198
1034.8293	3.4565
1046.3057	0.1855
1049.3521	11.8569
1054.5118	1.1945
1065.4290	4.4135
1067.5484	44.0076
1080.4709	40.7368
1100.4441	1.3811
1117.7135	39.7027
1118.5728	30.3154
1134.5831	47.2754
1150.0309	4.7928
1184.3181	26.4083

Frequency (cm^{-1})	IR Intensity (KM/Mole)
1190.0278	25.1069
1199.9358	138.3498
1220.8367	17.4747
1223.6391	1.4840
1227.6019	1.1541
1231.9242	5.2925
1234.2021	6.5100
1239.1764	5.5964
1296.5883	0.6786
1299.9703	170.8512
1320.9995	13.2024
1330.2182	13.6990
1332.9032	6.5796
1349.4248	12.5010
1364.2166	112.2515
1366.6041	5.1931
1395.7658	53.3252
1402.1124	9.4918
1415.2332	10.2259

Frequency (cm^{-1})	IR Intensity (KM/Mole)
1417.6564	13.3499
1421.9828	178.3530
1430.4463	14.8243
1446.7456	8.2327
1457.6716	91.2290
1472.1686	8.2912
1493.8806	5.1780
1496.5630	10.9126
1509.8351	18.3766
1513.1914	11.7616
1518.5767	42.0417
1538.2228	7.1106
1545.6742	61.7389
1552.4613	5.2386
1554.9613	5.6372
1563.9301	6.7532
1566.5429	6.1417
1598.8009	18.4837
1609.3296	22.1794

Frequency (cm^{-1})	IR Intensity (KM/Mole)
1629.2358	48.4698
1643.7158	667.3518
1656.2862	186.0314
1682.0170	421.4075
3005.4126	76.4027
3026.8108	33.3059
3031.7717	71.4525
3043.4445	2.1262
3062.7212	47.5700
3063.0432	43.8902
3083.0361	25.3196
3086.7918	31.4722
3095.2885	74.6196
3096.2117	118.8423
3118.0821	5.9734
3136.1363	9.5326
3144.0610	12.0793
3181.6724	9.4130
3189.6373	2.7214

Frequency (cm⁻¹)	IR Intensity (KM/Mole)
3199.1975	38.9194
3199.6477	7.0969
3213.5303	22.6262
3218.3303	51.3589
3226.9187	17.5173
3240.9112	7.8187
3411.6457	285.1091
3491.1027	38.8115
3543.1949	0.3909

Bortezomib (de2) in water

Frequency (cm⁻¹)	IR Intensity (KM/Mole)
21.8470	0.5370
24.2735	1.9222
29.2361	1.1662
35.6777	1.3809
41.8740	0.2995
54.1719	0.4451
59.0221	2.2950

Frequency (cm^{-1})	IR Intensity (KM/Mole)
62.7085	2.9428
73.1355	0.3074
92.2392	1.0725
93.8370	1.0503
122.3717	4.7217
133.1927	1.8416
156.4436	1.9155
181.2817	3.9839
188.7387	5.6704
200.6538	16.7717
212.1979	1.2801
218.9484	0.8425
246.3653	2.8767
248.0322	2.4767
256.0650	2.9445
290.7873	9.2244
316.9718	4.3011
328.9361	9.8807
343.8964	2.5459

Frequency (cm⁻¹)	IR Intensity (KM/Mole)
362.3748	2.7784
364.5428	8.8988
392.9340	28.9269
409.3150	10.3769
430.3021	12.9988
432.9069	1.9042
433.1185	13.2012
446.2311	151.9465
479.5812	17.9780
481.7297	23.4798
512.8360	28.6338
527.4470	83.3898
554.4282	57.1072
585.3196	3.6840
596.7626	65.7349
619.6320	2.4845
636.5618	15.5000
649.2963	1.7775
659.8534	1.4471

Frequency (cm^{-1})	IR Intensity (KM/Mole)
666.4544	100.1449
698.1913	38.8823
735.3889	7.9826
743.6075	72.8925
753.1127	24.4657
773.2861	89.5930
786.4737	7.8383
796.2559	8.0252
813.0385	4.9691
822.6323	20.4393
829.4901	16.0833
863.5923	181.0346
880.6772	9.8935
890.9098	3.7954
895.9870	27.2223
912.8888	18.8469
929.0053	160.1094
953.0998	24.4667
966.4809	120.8498

Frequency (cm^{-1})	IR Intensity (KM/Mole)
971.0102	8.1292
981.4286	6.1442
986.8931	4.5765
995.9519	6.4410
1007.2411	0.5613
1022.8257	42.3363
1026.9627	4.3991
1034.7499	3.4275
1046.7800	0.1709
1049.3992	11.7951
1054.3398	1.3069
1065.3751	4.7237
1067.4575	45.1783
1080.3153	40.5569
1100.5635	1.4693
1117.8641	41.3109
1118.5497	30.8127
1134.7966	48.2770
1149.7958	4.7104

Frequency (cm^{-1})	IR Intensity (KM/Mole)
1184.4963	27.5744
1189.9984	24.9294
1200.2491	138.8662
1220.4846	17.6685
1223.5286	1.5250
1227.5610	1.1376
1231.8810	5.7331
1234.7825	6.5219
1239.0783	5.4822
1295.4030	0.7430
1300.3474	172.0336
1320.9688	13.7726
1330.5088	14.5436
1333.0644	6.4309
1349.8090	12.9663
1364.0632	111.4662
1365.9598	6.5035
1395.6943	51.3173
1402.0383	12.7143

Frequency (cm^{-1})	IR Intensity (KM/Mole)
1415.2208	9.9433
1417.7208	14.4232
1422.2917	179.6894
1430.6407	17.3816
1446.7995	7.9020
1458.5432	92.2584
1471.4832	7.9211
1493.8373	6.3518
1496.8062	10.0968
1509.7635	19.0394
1513.3243	12.0762
1518.2316	41.8530
1538.1948	6.7799
1545.6100	62.0869
1552.0321	5.3524
1554.5292	5.8690
1564.0643	6.9386
1565.4707	5.8575
1598.6482	18.9865

Frequency (cm^{-1})	IR Intensity (KM/Mole)
1609.1806	22.6732
1629.0135	51.5831
1642.4865	661.9511
1655.6395	204.1168
1681.0893	426.9166
3006.4539	76.6263
3026.7585	33.6769
3031.8425	70.2094
3043.1276	2.3984
3062.4291	51.5231
3062.7294	43.9780
3082.8978	27.7132
3087.2485	31.5845
3095.2341	84.5659
3096.2632	110.9635
3118.0327	6.0771
3136.7520	9.2391
3144.2106	12.6079
3182.1350	9.4605

Frequency (cm^{-1})	IR Intensity (KM/Mole)
3189.8739	2.7882
3199.4548	39.0528
3200.0111	7.0340
3213.7560	22.5389
3218.5720	51.1807
3226.9641	17.3062
3241.2824	7.8245
3412.2855	286.6150
3491.0941	40.5041
3543.2829	0.3714

Threonine calculations at level of theory - B3LYP/3-21G*

Threonine (Thr1-OH) in gas phase

Frequency (cm^{-1})	IR Intensity (KM/Mole)
65.6925	3.5802
130.2671	1.7789
168.8101	27.8023
233.0103	4.6019
259.1875	4.2292

Frequency (cm^{-1})	IR Intensity (KM/Mole)
288.9216	0.6464
309.8608	3.6043
350.9692	1.5457
430.5604	7.0798
474.8734	15.9425
527.5247	39.7560
563.5848	44.5780
592.3763	89.3207
628.6439	138.9156
673.7139	206.0909
716.6965	57.3618
816.2301	6.9445
863.5594	8.5060
949.5063	9.3979
1065.4035	28.2859
1076.8555	8.7321
1096.3564	261.1902
1138.3096	10.8360
1146.6538	71.9809

Frequency (cm^{-1})	IR Intensity (KM/Mole)
1229.6945	7.0374
1291.3072	0.8169
1302.9041	6.1660
1333.0885	10.4573
1392.3036	4.2794
1433.8929	31.9743
1442.8944	7.4167
1460.0204	83.7332
1563.5394	8.6033
1567.0476	0.7714
1690.6810	100.6236
1790.7296	127.6593
2986.6013	27.3523
3054.4735	13.8690
3091.7948	18.0740
3125.4024	38.1714
3149.7583	8.6990
3439.5024	48.8457
3448.5411	9.5549

Frequency (cm⁻¹)	IR Intensity (KM/Mole)
3475.1020	16.3677
3561.1758	12.1230

Threonine (Thr1-OH) in diethylether

Frequency (cm⁻¹)	IR Intensity (KM/Mole)
58.1174	4.7998
103.8587	52.7147
111.9281	3.7224
223.2178	2.7203
257.5527	2.7633
275.3426	10.5240
300.5325	8.3572
352.9176	9.4633
420.4721	18.9254
474.0099	11.5761
523.5771	47.7773
564.3732	35.0351
611.8279	67.6889
635.8330	247.7569

Frequency (cm^{-1})	IR Intensity (KM/Mole)
714.7693	7.0372
750.1542	333.0271
805.8805	9.4697
873.1064	19.8697
944.1496	13.7433
1048.5634	31.2815
1067.3379	187.9743
1091.3587	153.4455
1130.1036	52.7066
1157.2083	20.8988
1225.2827	16.2816
1279.0377	7.0834
1296.1311	9.8772
1344.7145	12.7064
1402.4587	4.4478
1427.8643	14.0033
1440.1871	19.5854
1467.8828	133.6597
1550.7308	1.4071

Frequency (cm⁻¹)	IR Intensity (KM/Mole)
1560.6071	11.1616
1727.6058	75.8182
1778.8708	191.2135
3051.1670	15.3709
3097.5680	5.7381
3103.3970	10.8848
3123.9717	60.6340
3136.5554	21.0963
3389.4793	111.3692
3412.7167	1.3184
3465.7293	29.3007
3501.4725	5.6258

Threonine (Thr1-OH) in methanol

Frequency (cm⁻¹)	IR Intensity (KM/Mole)
56.3474	6.4921
109.5344	8.4005
115.6727	57.1258
223.9911	3.1268

Frequency (cm^{-1})	IR Intensity (KM/Mole)
259.6136	2.8850
275.3333	13.7890
300.4440	8.3425
353.8286	10.9195
419.4809	20.6850
474.7350	14.7763
522.6164	60.9241
563.0657	33.0007
610.2101	134.1593
625.4884	213.4026
715.7071	9.8026
764.1238	353.6830
805.7407	10.1993
874.4286	25.4936
944.6463	18.1273
1046.6356	37.1745
1065.3776	240.7538
1090.1221	143.8735
1128.7602	55.7111

Frequency (cm^{-1})	IR Intensity (KM/Mole)
1158.0940	22.1016
1226.7040	17.9980
1278.8467	6.6668
1295.4564	11.2961
1345.6586	15.3441
1402.4550	5.0044
1427.0831	16.1837
1439.3195	20.3148
1470.0921	155.7880
1547.9164	1.2521
1557.6844	12.6227
1724.4458	79.8248
1775.3522	220.8402
3051.7527	16.2253
3095.9792	16.0638
3104.3524	9.9307
3122.4665	68.2303
3134.7795	25.2108
3374.4045	140.6348

Frequency (cm^{-1})	IR Intensity (KM/Mole)
3406.7460	1.4742
3467.6184	40.8586
3493.6449	5.8369

Threonine (Thr1-OH) in water

Frequency (cm^{-1})	IR Intensity (KM/Mole)
56.1611	6.4453
109.1299	9.8048
115.2483	56.9251
223.9933	3.1734
259.7887	2.9030
275.2780	14.0757
300.3635	8.3321
353.8551	11.0346
419.3427	20.8560
474.8427	15.0904
522.4556	62.4634
562.7120	33.7398
609.8819	146.0745

Frequency (cm^{-1})	IR Intensity (KM/Mole)
624.6325	203.8860
715.7772	9.9963
765.0529	356.0053
805.6600	10.3537
874.4397	26.0625
944.6958	18.6217
1046.4580	38.1920
1065.1485	246.3876
1089.8955	142.7115
1128.6128	56.1426
1158.0915	22.1787
1226.8217	18.1382
1278.7566	6.6791
1295.3787	11.3936
1345.6719	15.6529
1402.4236	5.0730
1426.9814	16.5192
1439.1194	20.3237
1470.2172	158.0449

Frequency (cm⁻¹)	IR Intensity (KM/Mole)
1547.6034	1.2283
1557.3449	12.7783
1724.0036	80.3644
1775.0227	224.0152
3051.8427	16.3275
3095.6285	17.4201
3104.4320	9.9004
3122.3554	68.9014
3134.6019	25.6701
3373.0021	143.7106
3406.5388	1.5149
3467.7226	42.0937
3493.3044	5.9086

Deprotonated threonine (Thr1-O-) in gas phase

Frequency (cm^{-1})	IR Intensity (KM/Mole)
35.5775	1.0498
43.0621	1.2286
81.9193	1.7902

Frequency (cm^{-1})	IR Intensity (KM/Mole)
121.1974	2.0237
176.2655	4.7548
195.9571	30.1585
214.1510	19.6765
276.3934	1.5155
332.2699	64.2371
409.1632	9.9962
433.6936	63.3566
477.4811	20.1408
518.2764	11.2899
601.9774	29.2731
670.7232	35.2276
728.1703	191.4329
757.2629	39.4813
878.5616	7.2526
914.6386	49.6264
941.9540	36.2699
1017.3842	253.6391
1153.4952	22.4781

Frequency (cm^{-1})	IR Intensity (KM/Mole)
1189.1222	8.9505
1200.3388	101.3605
1222.9950	0.4072
1260.3904	34.3739
1420.9467	28.0173
1447.3402	139.8630
1476.9749	24.6798
1549.5614	54.7587
1579.6687	116.7522
1682.8527	143.5520
1731.7020	95.6932
1776.9236	223.8592
2731.8618	784.9694
2942.3442	36.9165
3036.3834	15.7549
3106.7447	109.8357
3114.6328	33.5712
3289.5382	97.0005
3328.4598	34.2032

Frequency (cm^{-1})	IR Intensity (KM/Mole)
3450.2992	2.0968

Deprotonated threonine (Thr1-O-) in diethylether

Frequency (cm^{-1})	IR Intensity (KM/Mole)
69.3610	5.6674
104.6759	5.4063
164.8876	9.8551
235.0243	144.8064
253.1932	4.2487
276.1778	9.9003
296.2152	69.1236
324.7268	4.2992
392.9671	31.9013
427.2271	83.8518
478.0778	33.4892
550.1403	88.5905
584.8674	103.4675
630.7892	58.4617
757.2636	91.6509

Frequency (cm⁻¹)	IR Intensity (KM/Mole)
779.7702	139.0770
801.4263	105.2951
897.0164	16.3382
1023.5311	445.3758
1033.0983	37.3012
1061.6987	26.8470
1128.0766	46.2238
1149.5009	46.4828
1232.3993	109.5817
1313.8646	27.9737
1322.2394	91.5455
1343.8318	40.3102
1398.7165	29.6078
1438.4869	17.7124
1472.5469	160.4344
1547.8511	24.4725
1558.6224	7.5873
1692.0024	37.2497
1737.5194	342.5060

Frequency (cm⁻¹)	IR Intensity (KM/Mole)
2399.4825	668.9415
3022.8980	65.5200
3060.9527	72.2983
3090.7040	56.3266
3128.3569	44.1960
3341.6497	39.5152
3426.4284	2.2502
3459.3621	2.0447

Deprotonated threonine (Thr1-O-) in methanol

Frequency (cm^{-1})	IR Intensity (KM/Mole)
71.5574	5.3160
93.9284	4.6718
147.4020	12.3117
212.2493	6.8142
229.6523	108.3429
271.3016	5.4873
290.9913	18.2020
314.7824	70.1557

Frequency (cm⁻¹)	IR Intensity (KM/Mole)
395.0393	6.6699
428.3434	124.1113
481.6490	28.2585
571.3527	142.6188
605.6251	94.2547
630.5629	70.9028
762.6701	74.8234
785.2919	174.2221
805.0420	122.6218
895.5467	32.1001
1027.2397	432.9119
1050.6081	58.3137
1065.7803	46.0524
1135.5351	48.9474
1156.6368	48.5176
1236.0767	139.9109
1307.0252	110.2946
1324.6550	40.2106
1347.4117	43.9154

Frequency (cm^{-1})	IR Intensity (KM/Mole)
1396.1041	32.3239
1437.0822	27.0627
1464.0077	147.2319
1531.8526	3.2307
1547.0485	22.2811
1691.3338	47.6234
1734.0706	376.4163
2396.1013	758.4722
3023.5581	65.1474
3043.7511	87.9302
3089.2630	66.1897
3125.9796	54.7318
3354.1979	27.0810
3436.0932	0.6060
3460.3566	7.1206

Deprotonated threonine (Thr1-O-) in water

Frequency (cm^{-1})	IR Intensity (KM/Mole)
69.5709	5.1940

Frequency (cm⁻¹)	IR Intensity (KM/Mole)
103.3488	5.9087
149.8442	11.2255
208.0588	6.9007
229.9723	109.1325
268.9164	5.6552
291.9706	12.8808
316.3630	72.8420
395.3012	5.7811
429.6523	124.1124
481.6200	28.8373
571.7608	146.1093
607.9804	94.9395
631.2937	72.1282
763.1563	71.4839
786.0641	181.4089
804.8312	122.5865
895.4620	34.2477
1027.7680	441.5530
1052.3084	47.5576

Frequency (cm^{-1})	IR Intensity (KM/Mole)
1065.5339	51.6592
1135.0645	46.9401
1159.9150	49.4201
1236.3884	145.7145
1305.5794	113.8923
1324.6339	36.7439
1347.8080	45.5792
1395.5091	31.7886
1436.5378	27.6513
1463.0845	146.0955
1532.7838	3.7656
1545.6005	21.5600
1691.5632	48.6635
1733.8018	379.3320
2396.6742	767.2492
3023.6257	64.8629
3043.7043	89.6822
3089.0579	67.3133
3125.1408	56.3969

Frequency (cm^{-1})	IR Intensity (KM/Mole)
3355.1400	26.0430
3436.9012	0.5319
3460.4246	7.8107

Bortezomib calculations at level of theory - B3LYP/6-311+G(d,p)

Bortezomib in gas phase

Frequency (cm^{-1})	IR Intensity (KM/Mole)
8.7177	0.3371
17.0960	1.0505
20.8532	0.3983
23.1471	0.2273
29.8559	0.1191
37.6269	0.2903
49.8441	1.7292
53.2000	0.4794
58.8514	0.7589
64.8618	0.0669
76.4635	0.3697
96.9323	3.0654

Frequency (cm^{-1})	IR Intensity (KM/Mole)
105.1639	0.5526
121.6771	3.6560
156.4390	0.3705
166.7649	3.3430
174.6526	0.5961
198.3628	0.2489
210.7046	0.5856
225.9263	0.1218
242.2255	0.7125
254.2273	0.1337
273.3804	3.4304
295.1086	3.6788
325.4072	0.2718
343.1341	8.7307
353.7641	2.8240
365.6656	3.7007
378.4758	8.3940
389.0769	2.9187
413.2117	1.2292

Frequency (cm^{-1})	IR Intensity (KM/Mole)
414.8339	0.1936
422.1926	1.6409
423.7922	14.5934
451.6605	50.4138
463.2125	3.8983
475.0311	26.7590
509.6860	15.4821
535.0620	35.1243
559.2795	15.0376
568.6979	142.5780
579.9555	59.2756
602.5530	30.7738
616.4261	37.8309
634.1368	14.2114
635.9339	0.8298
650.9163	67.9334
678.8129	26.0911
695.6187	36.6211
712.7618	47.1291

Frequency (cm^{-1})	IR Intensity (KM/Mole)
739.8882	7.2148
765.4081	21.5603
767.8228	5.3391
773.5113	45.9883
793.2554	18.9491
799.4440	8.6422
814.5511	18.6930
837.2141	8.9510
860.1776	0.4899
865.6583	6.8828
885.3756	15.2451
888.8784	12.0159
929.4559	8.3349
934.4881	7.8434
938.9448	130.4068
944.5642	11.4224
949.9216	13.0352
964.5377	0.3769
965.4266	2.1651

Frequency (cm^{-1})	IR Intensity (KM/Mole)
989.1587	0.7892
993.6757	1.2457
996.3967	0.0832
1006.2953	0.2226
1012.3902	117.3143
1017.7542	0.0206
1037.1569	28.7272
1047.8332	10.8650
1050.7580	7.2485
1066.9602	23.5441
1084.9131	4.4547
1109.0302	23.7704
1114.0208	3.2626
1125.1472	3.4697
1164.6268	12.9654
1176.1653	114.2603
1180.9426	4.5943
1187.6552	9.6462
1198.8884	3.3252

Frequency (cm⁻¹)	IR Intensity (KM/Mole)
1203.6871	13.4369
1204.7051	5.1437
1220.1773	0.2568
1226.4898	4.7434
1233.3764	10.0173
1269.8119	23.5814
1295.8544	25.3353
1301.7294	121.9329
1310.8237	55.6231
1316.2877	6.1110
1339.1311	14.5696
1341.0428	8.7329
1352.5996	67.0332
1359.3389	94.5374
1364.8902	1.2185
1380.1650	139.3312
1383.6269	15.3163
1399.1476	93.1137
1403.9286	108.0019

Frequency (cm^{-1})	IR Intensity (KM/Mole)
1405.8760	83.5567
1408.1447	97.9302
1424.9298	20.3307
1442.5751	74.2763
1479.9358	5.0441
1481.3562	4.7032
1482.1968	3.7679
1487.8370	28.1191
1491.5775	8.7212
1494.8374	10.0150
1497.9055	4.0328
1501.3854	14.4166
1506.9453	5.9091
1516.8957	10.8994
1525.4451	15.3090
1578.5280	1.1367
1610.5858	4.4432
1621.2907	1.7991
1642.5852	7.6815

Frequency (cm⁻¹)	IR Intensity (KM/Mole)
1719.3195	456.8628
1726.8189	418.9098
3009.0464	19.1141
3010.9609	6.4482
3012.9144	40.3111
3020.2694	23.7753
3039.4035	12.8811
3045.6104	19.8417
3047.1893	32.1269
3071.6789	7.4802
3073.5194	8.6171
3079.2614	60.2616
3084.7114	34.9830
3087.6569	44.2935
3102.9678	7.8700
3144.7342	9.9843
3155.9017	1.3423
3160.8777	0.6051
3171.3833	21.4477

Frequency (cm⁻¹)	IR Intensity (KM/Mole)
3172.7027	36.4045
3179.1043	0.0236
3184.0608	20.5542
3196.3270	4.2642
3557.5317	20.0602
3584.1100	41.5430
3857.2836	13.5943
3861.6341	91.3216

Bortezomib in diethylether

Frequency (cm^{-1})	IR Intensity (KM/Mole)
16.1255	0.2548
18.1836	1.5739
20.8324	1.8231
22.8004	0.2005
27.7928	0.6387
40.2925	0.5466
44.2516	1.4991
48.7132	0.2340

Frequency (cm^{-1})	IR Intensity (KM/Mole)
54.5603	0.7147
71.6853	0.7779
76.2975	0.0232
95.3109	4.2817
105.3882	0.6207
113.8609	4.6976
155.7653	0.3113
159.9205	4.4946
186.8288	0.3145
193.8401	0.4562
202.4238	0.5818
226.1775	0.2135
241.8290	0.7823
254.8923	1.2697
259.6369	2.2855
289.2768	5.6707
326.4370	0.2700
338.7512	14.2256
346.9480	7.5505

Frequency (cm⁻¹)	IR Intensity (KM/Mole)
352.9936	1.5424
375.1321	15.3085
389.1743	2.9663
414.4165	29.5749
414.8413	0.0597
422.3527	1.4485
443.6755	56.8369
457.5537	7.6161
470.3334	13.3895
481.7950	46.1929
508.4208	5.9305
538.1290	43.0183
565.1526	218.4211
570.8384	63.2962
596.7140	57.5101
600.7069	33.5212
613.3350	50.8949
633.9939	21.0546
634.4907	1.1545

Frequency (cm^{-1})	IR Intensity (KM/Mole)
638.7008	40.3176
671.0810	107.0925
691.7681	24.7518
712.0954	67.0125
739.9822	7.0375
763.6981	12.0709
771.1116	77.5089
772.1894	2.3458
786.5333	2.8404
796.6713	5.2264
815.2264	13.9344
837.3429	12.1102
859.9180	0.3510
866.0797	10.7936
883.2483	24.6445
888.4519	15.1815
927.6614	9.0640
935.3016	5.4722
943.3751	7.2611

Frequency (cm⁻¹)	IR Intensity (KM/Mole)
952.7959	7.5371
955.4905	220.5924
964.3830	0.3304
966.7790	2.9362
991.6693	0.8022
994.9934	3.3121
999.4709	0.3517
1008.6978	0.4049
1009.9087	130.3613
1015.9011	0.0388
1035.2642	33.0646
1047.9071	9.4691
1049.6286	16.4727
1067.8973	32.7696
1085.2921	5.0249
1106.7448	45.9085
1111.6164	5.0363
1118.8287	4.8019
1161.6208	13.3016

Frequency (cm^{-1})	IR Intensity (KM/Mole)
1177.0090	1.2607
1185.3644	16.3633
1186.6076	93.5485
1197.7967	2.0048
1198.0923	1.6479
1205.8787	42.8906
1214.1805	7.9572
1220.0169	1.2284
1226.3950	14.3782
1268.1304	36.5009
1293.2920	60.6943
1305.3163	129.5154
1316.6327	31.6346
1321.0120	17.3609
1337.6604	18.7508
1338.7193	11.8417
1354.1602	32.2717
1357.5024	168.8373
1364.8475	76.7453

Frequency (cm⁻¹)	IR Intensity (KM/Mole)
1377.0262	278.1257
1380.2454	13.5659
1395.4410	68.1681
1399.9619	68.8912
1410.5945	184.0247
1415.7518	97.0489
1420.4022	19.3506
1440.7085	93.0558
1476.1778	12.4954
1478.7827	3.4760
1481.6968	4.9410
1485.3388	8.1949
1490.1597	10.6826
1492.5531	3.4663
1494.9976	36.4491
1500.0541	11.7944
1506.5183	59.3267
1509.2808	14.3570
1523.4441	18.7106

Frequency (cm^{-1})	IR Intensity (KM/Mole)
1575.7093	4.4927
1613.6647	6.0607
1619.3700	1.6834
1639.7344	11.5693
1690.8147	770.2644
1702.3515	636.3611
3008.6449	4.5284
3009.5692	16.0937
3012.5306	75.2051
3019.7525	34.6725
3043.0580	21.5047
3049.0845	44.5252
3050.9070	24.7303
3070.9116	18.9827
3078.1853	83.7375
3083.3449	56.9110
3087.5935	54.1685
3092.4918	4.3455
3111.7526	14.4105

Frequency (cm⁻¹)	IR Intensity (KM/Mole)
3148.7738	14.0680
3162.8930	0.5558
3164.2048	1.1070
3168.0164	19.8574
3172.5806	28.5793
3180.2649	30.8630
3184.2936	26.6814
3192.5775	7.9928
3564.7811	43.9817
3578.9583	61.2579
3835.6542	19.3340
3843.7135	162.9600

Bortezomib in methanol

Frequency (cm⁻¹)	IR Intensity (KM/Mole)
16.8859	0.8205
21.1712	1.9154
22.1672	0.3874
28.9162	0.5794

Frequency (cm⁻¹)	IR Intensity (KM/Mole)
33.0619	3.2312
40.2908	0.0623
44.8962	0.4978
48.1502	0.2876
55.2993	1.0192
74.6144	0.5620
76.2197	0.4653
94.0188	3.6018
106.6895	1.0315
112.8285	4.7171
156.3324	0.5792
160.0002	4.9725
188.8764	0.3428
194.9092	0.3323
201.8505	0.8159
226.9212	0.1660
242.8094	0.9569
253.3549	0.6505
261.1228	3.2111

Frequency (cm^{-1})	IR Intensity (KM/Mole)
288.2327	6.0437
326.5062	0.9073
340.8728	16.8300
346.1474	9.6099
355.5846	0.7276
372.9283	18.3868
388.7555	2.6820
412.5495	34.8585
414.6892	0.1285
422.2530	1.8015
450.0185	58.9436
459.6054	10.5873
480.2960	70.3147
495.5357	2.6718
516.9875	4.3046
539.5760	42.1467
564.0562	267.6576
572.0982	68.3598
598.2069	87.0834

Frequency (cm^{-1})	IR Intensity (KM/Mole)
604.0068	11.2033
610.5382	93.2057
630.3419	37.6461
633.8439	3.1588
634.4045	7.6545
669.3837	140.6187
692.3905	28.2792
711.5898	77.3614
739.7716	3.8312
762.6293	19.1907
768.1118	58.4013
772.0518	23.8719
785.4895	2.7936
796.2340	6.4848
815.8379	12.3311
837.7020	12.8504
859.5137	0.0543
867.5749	8.9842
883.6813	33.1966

Frequency (cm^{-1})	IR Intensity (KM/Mole)
888.0683	15.8933
926.8053	9.6143
936.5508	5.7049
943.0682	5.7390
953.1543	6.6604
961.9741	265.8512
964.0285	3.2436
967.7889	5.1403
992.5890	0.2991
995.7653	0.3718
1002.7777	0.3226
1009.7630	0.0597
1015.2064	0.1299
1030.6190	174.1575
1035.0314	34.7849
1047.5994	12.2680
1051.3779	18.3801
1068.0874	36.0794
1085.7151	6.4641

Frequency (cm⁻¹)	IR Intensity (KM/Mole)
1108.1785	59.2780
1110.9425	5.9668
1116.1915	7.9471
1162.4814	12.6574
1175.4925	0.3342
1185.0244	3.3018
1188.6402	95.7887
1196.9386	6.2226
1198.3714	5.7535
1207.4026	49.4950
1211.1004	12.9635
1220.5064	1.8917
1226.6296	10.3351
1268.4854	29.3890
1296.2434	73.0944
1309.3530	147.0034
1317.5127	20.4766
1322.3654	25.8904
1337.1930	21.4426

Frequency (cm^{-1})	IR Intensity (KM/Mole)
1338.4298	16.6221
1354.8056	26.7028
1357.3706	234.6648
1365.9557	197.2275
1374.6677	241.4123
1379.4880	22.2468
1394.9922	22.5313
1398.5787	89.9475
1412.4425	197.0607
1417.8297	101.0798
1419.0206	29.9860
1440.6497	117.5889
1474.9388	13.5734
1476.6849	6.2832
1481.5993	10.4998
1481.7575	4.5312
1487.8617	11.9719
1490.4616	4.7572
1494.1358	42.4896

Frequency (cm⁻¹)	IR Intensity (KM/Mole)
1497.7153	20.2694
1505.1416	16.9180
1509.3898	74.9655
1523.3941	19.5534
1574.8586	5.2134
1613.1978	11.8654
1618.5417	1.1772
1638.6981	15.7365
1674.5858	953.9365
1689.6285	758.7035
3005.5198	8.9405
3009.8573	20.4398
3013.1449	94.2943
3019.7175	39.6273
3046.9116	33.7022
3050.7142	43.4989
3053.9133	29.5827
3070.2934	30.8359
3077.2759	99.7487

Frequency (cm^{-1})	IR Intensity (KM/Mole)
3081.8467	70.3302
3086.9475	61.5841
3097.2006	4.2160
3117.1608	20.2576
3152.9702	13.2132
3163.5821	0.2472
3168.3129	1.1274
3171.1126	21.1013
3173.0161	29.3862
3183.1227	36.6272
3183.4991	30.5290
3190.1548	13.8612
3566.5114	63.6765
3576.3431	67.1860
3830.7021	83.5035
3836.0514	129.0758

Bortezomib in water

Frequency (cm^{-1})	IR Intensity (KM/Mole)
16.8247	0.7906
20.3181	1.5216
22.0171	0.6018
26.9622	0.8514
33.0189	3.3157
39.8020	0.0401
44.5511	0.4365
47.8950	0.3401
55.6161	0.9976
74.0102	0.5764
75.7300	0.5277
93.7685	3.6331
105.8748	1.0717
111.9353	4.7188
156.0144	0.6059
159.8335	5.0135
188.3095	0.3631
194.8141	0.3596

Frequency (cm^{-1})	IR Intensity (KM/Mole)
201.4493	0.8539
226.8318	0.1676
242.9098	1.0017
252.9806	0.6159
260.6902	3.3691
288.2578	6.1187
326.3836	0.9524
340.9468	16.6745
346.0457	10.0264
355.4437	0.7484
372.4971	19.0649
388.5791	2.5711
412.2877	35.3682
414.2500	0.1430
422.1150	1.9310
449.7842	60.8528
459.4789	10.7566
480.1619	73.5403
493.3156	2.2445

Frequency (cm^{-1})	IR Intensity (KM/Mole)
517.0269	4.3376
539.3701	41.9612
563.6412	266.9088
571.9726	71.2194
597.5798	90.8004
604.0393	10.2270
610.3731	98.0858
629.8883	37.9074
633.7404	3.6707
634.3641	7.8102
668.8089	142.9159
692.3700	28.3683
711.1749	79.0338
739.7108	3.5948
762.3391	19.7386
767.7686	57.6634
771.8719	25.2971
785.4716	3.0601
796.1282	6.5712

Frequency (cm^{-1})	IR Intensity (KM/Mole)
815.8089	12.4786
837.7138	13.0074
859.1464	0.0315
867.6131	8.7037
883.7034	34.2949
887.9925	16.0673
926.7188	9.7078
936.2395	5.5697
942.9902	5.7823
953.1087	6.8634
962.1431	267.9382
963.9328	5.8744
968.0717	5.0994
992.4674	0.2442
995.8228	0.3920
1002.9882	0.3336
1009.8889	0.0533
1015.1176	0.1290
1031.6507	177.4220

Frequency (cm^{-1})	IR Intensity (KM/Mole)
1035.0341	34.5537
1047.4678	12.2880
1051.5991	19.3585
1068.1107	36.7147
1085.8318	6.7453
1108.3196	61.1736
1110.8622	6.0548
1115.8292	8.2860
1162.4659	13.0530
1175.3582	0.3261
1184.9264	3.4621
1188.6397	95.4032
1196.8828	6.3747
1198.3084	6.3746
1207.5339	50.2491
1210.7841	13.6660
1220.4770	1.9508
1226.6759	10.0521
1268.3725	29.1771

Frequency (cm^{-1})	IR Intensity (KM/Mole)
1296.3008	74.0924
1309.5174	148.3184
1317.7068	20.1924
1322.9794	26.2596
1337.1006	21.3904
1338.3198	17.4358
1354.8524	31.2158
1357.2233	247.0386
1365.9196	225.6202
1374.7209	213.2605
1379.3585	24.8537
1394.9083	20.2458
1398.4329	92.8706
1412.5940	196.9862
1417.7929	80.6196
1419.2813	50.1933
1440.7849	120.1340
1474.4586	13.8877
1476.1718	7.0919

Frequency (cm^{-1})	IR Intensity (KM/Mole)
1481.0571	8.7545
1481.6273	6.2220
1487.5318	11.6602
1490.1441	4.6178
1494.0611	44.7016
1497.3845	21.1437
1504.6743	17.1273
1509.6617	75.9589
1523.3996	19.5155
1574.6534	5.3141
1613.1831	12.5545
1618.4243	1.1064
1638.5748	16.3404
1673.0268	975.9022
1688.0524	774.1776
3004.9992	10.4014
3009.9046	21.8705
3013.2589	95.2259
3019.7110	40.0180

Frequency (cm⁻¹)	IR Intensity (KM/Mole)
3047.6934	36.0419
3051.0277	42.4043
3054.0706	30.0582
3070.2426	32.3961
3077.1815	101.5118
3081.6590	71.9504
3086.6378	62.8924
3097.1261	4.2636
3117.3614	20.8853
3153.5468	13.0620
3163.6288	0.2063
3168.8162	1.0947
3171.5475	21.2459
3173.0503	29.2247
3182.8407	37.6614
3183.9253	31.1844
3189.9298	14.6002
3566.1930	65.9955
3576.2452	67.2127

Frequency (cm^{-1})	IR Intensity (KM/Mole)
3829.4838	83.2850
3835.1844	132.0016

Bortezomib (de1) in gas phase

Frequency (cm^{-1})	IR Intensity (KM/Mole)
18.5812	0.8585
24.5779	0.5927
33.4139	0.1594
37.9902	0.0925
49.5200	0.2009
50.1465	2.3510
60.1762	0.7894
61.1532	0.6811
72.1912	0.1339
86.5539	2.8639
99.6012	1.0642
106.5467	0.6770
116.1848	0.3143
132.6662	17.6831

Frequency (cm^{-1})	IR Intensity (KM/Mole)
145.7475	3.2109
171.6454	10.4972
180.9574	1.3716
201.5104	3.7869
209.4112	0.0902
228.2946	1.7465
239.7267	4.6199
251.5030	7.0383
265.3707	1.2410
300.3983	3.9273
319.2490	8.0129
332.8033	10.1784
350.5900	16.4961
372.3443	3.3162
380.0783	12.5009
389.6595	1.8648
415.7938	0.2444
422.3270	0.0602
428.1155	12.0606

Frequency (cm⁻¹)	IR Intensity (KM/Mole)
447.1177	28.6739
473.5496	7.0098
488.5158	4.8226
519.2018	12.0015
524.1951	45.5077
553.7016	2.4050
571.5552	86.0766
586.7174	25.4487
614.7208	3.5326
632.8680	2.8497
635.4076	0.8924
654.6105	32.3060
666.5270	11.5767
696.5282	50.9443
712.6283	52.2749
742.0766	10.5780
752.7742	4.5587
755.5240	8.7217
758.0044	60.3238

Frequency (cm^{-1})	IR Intensity (KM/Mole)
792.6403	13.2300
793.6212	23.3391
806.7038	32.9360
832.4929	49.3709
839.8479	43.4083
858.7684	1.4917
866.8075	3.8506
886.2341	15.8691
893.4043	21.0909
932.1323	3.8388
932.3259	6.5622
942.0064	6.8938
959.5264	19.9776
964.5340	3.7338
964.8530	0.0582
986.8535	1.1219
988.2330	187.3824
997.3364	64.0972
1004.4968	0.6342

Frequency (cm^{-1})	IR Intensity (KM/Mole)
1015.3844	18.9991
1017.4957	0.1871
1020.8874	6.5102
1039.7546	16.9195
1051.0564	6.6920
1071.7635	40.3515
1092.8328	29.0616
1100.4457	9.2288
1105.2583	3.6501
1120.6371	3.4511
1127.6289	46.7168
1165.6260	14.5305
1177.8162	0.0465
1187.2951	12.8226
1189.0675	78.7201
1200.3775	21.8730
1201.4878	0.8066
1204.2003	0.5788
1221.5550	21.4305

Frequency (cm^{-1})	IR Intensity (KM/Mole)
1226.4452	3.3594
1229.6969	4.8469
1273.7484	0.9992
1302.0092	88.9033
1315.3516	1.0828
1319.6499	12.3319
1334.8808	0.9840
1338.4392	2.7441
1344.2591	15.6522
1348.2971	73.6586
1365.8948	3.3001
1375.6760	2.1383
1385.7342	19.4502
1394.4531	7.1523
1400.6572	102.2362
1419.3175	87.9537
1420.7039	14.4504
1448.5865	137.8323
1455.3151	22.7620

Frequency (cm^{-1})	IR Intensity (KM/Mole)
1475.0148	0.3103
1479.8086	22.3788
1481.8667	4.2023
1491.8635	14.7147
1492.5523	3.8123
1501.8080	7.8573
1503.7271	3.6899
1506.9491	18.2517
1518.4672	13.8920
1525.2475	11.9864
1532.3033	300.3480
1574.5508	5.4453
1612.4640	9.2997
1620.5417	2.4750
1642.9325	11.8534
1687.2750	577.0079
1691.0242	377.1780
2962.2427	64.1485
2996.3526	27.2910

Frequency (cm^{-1})	IR Intensity (KM/Mole)
3002.3477	38.8540
3004.3969	55.5462
3016.7356	40.7946
3025.9149	22.6012
3032.0837	30.3730
3049.7230	4.0726
3059.9360	99.0599
3074.4737	50.3489
3076.0643	3.4987
3093.0190	14.1598
3097.4357	6.6851
3120.7551	76.9604
3144.1028	6.8930
3155.1272	3.4819
3155.2450	28.7219
3165.5675	30.4726
3177.0249	21.1435
3181.7990	5.8295
3185.0867	13.3493

Frequency (cm^{-1})	IR Intensity (KM/Mole)
3436.7042	152.1716
3554.2989	12.6207
3826.9314	9.7635

Bortezomib (de1) in diethylether

Frequency (cm^{-1})	IR Intensity (KM/Mole)
28.0170	0.5281
33.2367	1.6122
38.4277	1.1170
51.1568	2.4450
57.1564	0.4503
59.6099	1.4204
66.8184	2.0445
73.6409	1.0880
88.8321	0.8421
91.3470	3.6732
97.3146	4.1639
104.5136	2.5903
114.7442	0.7215

Frequency (cm^{-1})	IR Intensity (KM/Mole)
127.1394	15.9427
140.5600	1.3615
170.8143	21.1861
182.6920	0.5210
202.5683	2.9913
208.1469	0.1226
234.0126	11.7804
237.7405	0.9227
252.1281	8.3269
290.6101	1.8315
298.1751	5.1509
319.7250	16.1012
335.3671	14.2326
349.1876	18.5000
366.0269	7.5146
383.4990	16.0175
399.3570	1.1055
417.9061	0.2140
422.9807	6.9831

Frequency (cm^{-1})	IR Intensity (KM/Mole)
425.2363	13.9013
442.1843	37.3063
467.0340	42.7097
486.9617	0.9959
518.3044	20.8448
530.3100	65.7534
549.2312	103.5751
561.4233	15.0832
585.7162	38.7069
614.6777	1.7404
634.3488	3.9019
634.6722	1.5925
651.9959	48.5327
667.3527	4.7839
695.3528	96.3149
714.4870	67.8453
738.3615	44.4450
750.1520	20.0246
753.2182	55.5832

Frequency (cm⁻¹)	IR Intensity (KM/Mole)
758.5994	22.5555
777.0532	87.2439
790.2095	15.4437
797.6925	4.9269
828.4056	42.6061
838.7113	30.4453
861.7698	1.2469
865.8643	1.8029
877.3981	25.0747
892.0651	28.6713
934.0760	10.7050
936.4629	9.6442
943.9026	4.8000
958.7316	56.2382
965.4799	4.4075
966.0855	1.0817
978.3970	262.3029
993.5313	0.0671
997.7760	34.5736

Frequency (cm^{-1})	IR Intensity (KM/Mole)
999.4866	12.6155
1008.6606	1.2999
1015.5434	2.3671
1019.5806	13.2787
1038.0555	28.7734
1049.6909	9.5815
1071.4240	40.1367
1092.6040	49.5478
1100.0913	16.2497
1106.7168	7.0301
1117.6053	47.0347
1121.7939	36.0764
1164.6269	16.7821
1176.7875	0.0070
1187.1761	16.2825
1191.9204	96.2059
1200.8734	1.6207
1201.0279	0.2754
1204.9270	24.0385

Frequency (cm^{-1})	IR Intensity (KM/Mole)
1222.8293	23.5710
1225.7287	16.3727
1229.0501	6.9253
1274.5047	1.1629
1306.0210	144.1728
1318.1239	12.5757
1325.1899	2.5430
1326.0141	14.1178
1342.0941	2.7866
1344.2478	7.2979
1350.1581	65.6139
1367.2373	5.1122
1374.7811	3.7599
1390.4448	23.9571
1397.6706	5.2848
1407.2946	148.5533
1419.6522	18.4592
1421.3641	146.2903
1451.8086	145.5841

Frequency (cm^{-1})	IR Intensity (KM/Mole)
1456.2884	78.2941
1474.4729	1.0823
1481.6350	6.4628
1482.9563	33.4173
1489.6345	24.9051
1492.2007	6.6499
1498.9385	117.8033
1502.3527	163.4534
1504.8270	27.7759
1512.5591	191.2440
1520.7111	14.1986
1524.5924	13.5814
1573.9767	11.7559
1615.0329	17.3778
1619.5833	3.6458
1640.3823	18.8068
1665.3292	762.0754
1672.0051	691.9448
2982.4267	69.1664

Frequency (cm^{-1})	IR Intensity (KM/Mole)
3001.9384	28.6963
3004.1765	12.6250
3009.1273	78.0822
3029.3010	52.5798
3032.1700	36.4597
3032.4601	59.9433
3055.9476	0.6332
3063.8620	137.5274
3082.8954	4.4886
3084.5685	52.2145
3099.0666	24.4367
3099.7468	11.0512
3151.7102	9.3667
3161.1729	0.8388
3166.6244	18.1270
3169.8552	20.0521
3177.7587	26.7069
3182.0246	15.9785
3185.1096	9.5937

Frequency (cm^{-1})	IR Intensity (KM/Mole)
3186.8040	21.3117
3466.2852	186.4495
3552.2573	34.4789
3826.9564	21.3102

Bortezomib (de1) in methanol

Frequency (cm^{-1})	IR Intensity (KM/Mole)
18.7182	0.4582
21.8909	2.0850
23.8262	1.2886
28.4437	1.3396
33.9517	1.3658
37.5113	0.5745
42.3811	0.1223
54.1187	0.9296
55.4758	1.0360
79.1117	3.1212
85.9882	1.2100
97.5770	3.2673

Frequency (cm^{-1})	IR Intensity (KM/Mole)
105.2666	2.2646
113.8746	11.5351
157.0721	11.6264
167.3493	1.3842
183.3997	4.0514
184.6481	0.9551
210.5755	3.1329
229.8487	8.6867
247.6331	3.3458
251.4545	19.0232
257.6780	1.5346
289.2740	11.7204
327.7187	7.9736
339.2316	32.0621
348.3224	1.6847
368.4649	5.6645
370.7564	24.1391
393.4935	3.0830
408.9529	11.8460

Frequency (cm⁻¹)	IR Intensity (KM/Mole)
415.1412	0.0515
419.2754	0.4611
436.2039	26.6250
464.3176	55.6304
474.5187	37.5908
517.8483	1.7309
537.5539	112.7520
541.8258	98.2249
568.6737	51.9177
588.2773	24.3920
623.7787	8.8296
629.8649	8.6276
634.0568	0.5347
646.2585	100.1309
655.3159	28.8208
701.0721	85.8739
713.3234	67.1697
726.6103	77.3400
741.6236	49.7019

Frequency (cm^{-1})	IR Intensity (KM/Mole)
746.0851	15.0735
765.0183	40.1268
776.8730	57.1904
785.5201	5.8719
792.3666	28.7044
816.5524	13.0751
838.9479	41.7563
850.2908	17.3057
861.9204	0.3009
880.6748	19.6072
888.9880	35.5259
924.2768	10.5685
933.8098	7.0161
943.9882	10.5761
963.4728	2.4566
968.3871	4.4038
971.8389	198.8178
983.5508	132.2125
994.2569	0.9497

Frequency (cm^{-1})	IR Intensity (KM/Mole)
999.2986	16.6203
1002.8266	0.1870
1010.4537	0.0607
1014.9487	0.0935
1036.9781	42.4708
1046.8593	6.8976
1051.0078	34.4836
1070.5829	18.9505
1093.2543	11.5008
1101.7912	0.5450
1107.0990	52.4758
1112.8604	109.9199
1118.0351	30.5902
1161.8285	23.8950
1174.0702	0.1404
1185.4899	14.1500
1186.6327	112.1230
1191.8931	3.5791
1195.5463	0.5803

Frequency (cm⁻¹)	IR Intensity (KM/Mole)
1199.7196	3.8763
1221.5341	13.8076
1222.7935	29.0621
1223.2847	10.8879
1271.3128	3.0382
1290.9633	53.0425
1311.5635	27.0302
1316.1064	0.5501
1320.2358	8.0478
1337.6253	6.7615
1340.8241	6.5676
1356.8880	14.4479
1368.3834	1.4510
1370.7436	13.1459
1389.6118	99.2428
1390.2668	277.5850
1394.1761	10.2388
1414.3556	18.5195
1423.1464	171.2340

Frequency (cm^{-1})	IR Intensity (KM/Mole)
1442.6521	118.5725
1456.3652	89.6593
1472.8816	8.7653
1476.2064	49.8650
1479.8863	0.6797
1482.7366	79.6853
1484.4966	338.8101
1487.9102	2.1399
1490.5355	206.7659
1493.7303	11.2920
1501.0846	64.1400
1506.9991	20.5664
1522.7556	20.5424
1572.6867	9.4040
1610.8777	47.5941
1617.8813	3.1959
1638.5816	29.3843
1649.9404	1029.0766
1662.5940	931.1627

Frequency (cm⁻¹)	IR Intensity (KM/Mole)
2991.2094	60.1622
2998.9512	9.0621
3005.1943	42.2355
3011.7343	87.4382
3018.7664	71.0618
3035.7052	74.5122
3055.5124	29.2901
3060.6917	0.8329
3066.8220	159.4352
3083.6449	53.7272
3101.2675	25.9507
3101.4695	12.7368
3157.6620	5.2154
3163.1519	0.3565
3168.4673	0.5647
3168.9255	6.0279
3171.8565	21.0508
3178.6037	45.0340
3182.6771	43.1574

Frequency (cm⁻¹)	IR Intensity (KM/Mole)
3187.7939	21.1649
3191.7972	0.9536
3469.6438	225.8368
3555.8824	84.8018
3817.7819	26.4799

Bortezomib (de1) in water

Frequency (cm⁻¹)	IR Intensity (KM/Mole)
8.8947	1.6026
19.6516	1.3884
22.0460	1.4118
24.8601	2.2086
30.2041	0.6949
37.1789	0.0505
41.8775	0.1520
53.7753	1.2210
55.2388	0.8466
76.8438	3.1042
85.6993	1.3833

Frequency (cm⁻¹)	IR Intensity (KM/Mole)
97.3821	3.4948
103.9875	2.4731
111.6911	11.5517
156.5470	11.4298
167.2579	1.2817
183.2473	3.8971
184.2207	1.0292
210.2146	3.2085
228.8914	7.5284
247.2569	2.7591
250.9309	22.4125
252.6828	0.3178
288.9711	11.9974
327.2432	7.2620
339.1493	33.4182
348.2058	1.6803
368.3063	5.8602
370.3967	24.3428
391.5851	3.4141

Frequency (cm^{-1})	IR Intensity (KM/Mole)
408.8990	12.1546
414.7509	0.0497
419.0011	0.4605
436.1342	26.9300
463.5228	56.8117
474.8066	38.9709
517.7216	1.5778
537.1387	114.7460
541.0462	100.9725
568.1698	52.7243
588.2560	25.0522
623.0532	10.2783
629.7919	8.6450
634.0099	0.5939
645.4333	105.1587
655.3168	26.7758
700.5958	92.3207
713.0105	67.3759
725.3647	79.1425

Frequency (cm⁻¹)	IR Intensity (KM/Mole)
741.4791	49.5199
745.8074	14.7659
764.9115	43.2794
776.0821	56.9690
785.4155	5.2935
792.2111	28.0165
816.4014	12.9807
838.6045	41.8728
850.1047	17.7041
861.7013	0.1980
880.8948	19.9185
888.6634	35.8193
923.5988	10.6227
933.6689	7.0770
943.7398	10.4179
963.1214	2.6512
967.9664	4.1046
971.6995	201.9985
983.4882	134.6914

Frequency (cm⁻¹)	IR Intensity (KM/Mole)
994.2457	0.7895
999.2681	17.3157
1003.2844	0.1881
1010.7852	0.0626
1014.9106	0.1013
1036.8944	42.9608
1046.7890	7.1725
1051.0316	35.0011
1070.4942	19.1881
1093.2790	11.9462
1101.8958	0.6217
1107.0878	54.4411
1112.6085	112.9983
1117.7511	31.1454
1161.5841	24.3661
1173.9029	0.1218
1185.2432	13.6486
1186.6673	114.7406
1192.0767	3.8070

Frequency (cm^{-1})	IR Intensity (KM/Mole)
1195.4842	0.6451
1199.6154	4.1945
1221.5215	13.5966
1222.8499	31.2916
1223.1379	10.6012
1271.1679	3.1058
1291.3433	54.8320
1311.5798	27.3177
1316.0824	0.5798
1320.1932	8.4316
1337.4427	6.9417
1340.4913	6.4443
1356.9195	14.5088
1368.2770	1.1739
1371.0440	13.7077
1389.3331	96.6067
1390.0688	288.0905
1393.9231	11.2558
1413.8889	18.9529

Frequency (cm⁻¹)	IR Intensity (KM/Mole)
1423.3561	179.9563
1442.6716	121.4143
1456.2589	90.6368
1472.2634	14.0405
1475.6935	57.0561
1479.8083	0.6578
1481.6933	176.3991
1482.6395	292.9718
1487.4260	1.6990
1490.2655	163.0166
1493.1764	8.5415
1500.9957	66.6025
1505.0057	18.6878
1522.7343	20.7801
1572.6482	9.6009
1610.9046	50.6949
1617.7955	3.2673
1638.4348	30.8924
1648.3290	1043.0431

Frequency (cm^{-1})	IR Intensity (KM/Mole)
1661.3356	945.8635
2991.8151	60.8018
2998.3161	10.8324
3005.4166	43.1539
3011.8252	86.5073
3019.8637	72.7538
3035.5929	76.5344
3055.8258	29.5339
3061.0873	0.8937
3067.0621	162.8363
3083.3154	54.1222
3100.1214	28.5642
3101.8294	12.2517
3158.0438	5.3404
3163.3430	0.3109
3168.6777	5.9145
3169.2304	0.6966
3172.1375	21.0891
3178.8462	46.5536

Frequency (cm⁻¹)	IR Intensity (KM/Mole)
3183.2283	43.9632
3188.0212	21.0545
3191.4003	0.7572
3472.0727	223.2604
3555.3909	86.1514
3817.4627	26.9781

Bortezomib (de2) in gas phase

Frequency (cm⁻¹)	IR Intensity (KM/Mole)
7.9609	1.5691
15.3275	0.2105
22.7744	0.6578
25.4084	1.0686
29.0590	0.2261
36.4291	1.5361
45.5017	1.1914
50.2408	0.4575
54.9418	0.3231
66.8104	3.8801

Frequency (cm^{-1})	IR Intensity (KM/Mole)
72.7659	0.6757
101.1782	1.3430
111.6497	3.4312
122.4775	7.5923
162.4561	1.6296
167.8581	6.1217
179.6615	1.1768
197.6808	0.2480
206.8125	2.9056
222.2445	0.8099
238.4072	1.9745
256.1660	2.4886
262.4416	1.9274
287.5014	5.8284
321.3116	0.8078
337.4126	9.8895
349.2754	5.6914
355.5643	3.7020
381.5560	7.3134

Frequency (cm^{-1})	IR Intensity (KM/Mole)
391.7660	7.2821
415.6317	7.7505
416.0493	13.1437
424.6494	0.0887
442.6666	46.0796
459.8862	0.8353
473.4062	14.9380
505.7992	10.6713
542.2893	25.9376
568.0867	102.0332
573.7414	12.5605
583.7524	62.0763
610.4944	17.2567
615.1738	5.5530
633.3347	1.9165
635.6950	2.5733
640.1723	6.3077
691.2192	36.8485
712.1524	49.6240

Frequency (cm^{-1})	IR Intensity (KM/Mole)
729.8821	4.5440
748.2789	7.7857
750.6778	42.5281
765.9495	20.1792
771.0670	52.3830
787.5005	6.4620
800.6588	2.7665
813.9474	26.2515
825.3707	3.1467
859.3809	1.9205
862.7834	10.3774
882.3206	13.8209
892.2690	20.8235
918.6788	70.2987
931.7375	5.0754
933.8191	4.4298
950.9390	3.0505
958.3908	3.7030
963.1620	47.7788

Frequency (cm^{-1})	IR Intensity (KM/Mole)
967.5386	117.6852
986.3644	1.5335
987.1817	13.4756
990.5875	0.2386
1004.7636	2.0096
1017.6920	0.2142
1035.4886	22.6636
1047.7952	4.1971
1050.9195	10.3411
1066.4430	31.0686
1082.8583	4.0448
1097.9765	28.3381
1103.6238	20.5333
1106.5286	24.5402
1112.7903	1.1643
1161.1376	6.7768
1177.5896	0.4969
1184.6179	96.2718
1186.5106	4.0436

Frequency (cm^{-1})	IR Intensity (KM/Mole)
1198.1355	0.9554
1202.8840	9.3523
1205.2409	21.2977
1208.4523	1.4836
1221.1020	1.7146
1221.8167	1.1536
1270.4815	1.4911
1306.6343	15.9915
1311.5414	49.5020
1314.4259	19.8185
1316.6602	11.3157
1338.3215	3.0318
1342.0837	9.9130
1354.0919	96.6256
1364.7567	2.2821
1377.5367	2.9343
1384.2475	4.3051
1394.8278	6.0969
1408.6433	87.3256

Frequency (cm⁻¹)	IR Intensity (KM/Mole)
1418.8792	64.7476
1421.3623	2.7166
1442.5643	61.1552
1455.2400	29.5149
1476.7195	9.6067
1480.0249	13.4650
1480.8202	5.7861
1487.6560	36.3745
1489.8248	2.7125
1492.4133	5.0017
1501.3877	7.0704
1502.7927	28.8873
1514.0021	15.5099
1524.1370	18.9287
1574.4629	456.6886
1578.4494	4.9693
1612.1564	1.9044
1619.4470	2.4855
1641.8567	12.9890

Frequency (cm^{-1})	IR Intensity (KM/Mole)
1691.1005	540.4028
1706.1072	558.1032
2949.8069	85.1203
2995.1619	14.1040
2998.3453	32.2713
3005.4808	81.0480
3029.6493	24.4208
3039.1089	29.4229
3040.1020	14.5414
3055.5063	22.1446
3064.4259	59.7132
3067.3240	71.6921
3091.4061	2.0093
3107.9514	9.8687
3110.0659	7.3752
3143.4678	6.8483
3147.4078	8.1362
3154.1334	3.6647
3161.1431	24.9291

Frequency (cm⁻¹)	IR Intensity (KM/Mole)
3165.1715	33.9765
3168.3722	27.4570
3177.7115	26.5513
3190.0568	9.4429
3539.0453	110.2373
3575.9191	21.5596
3835.5487	13.2194

Bortezomib (de2) in diethylether

Frequency (cm^{-1})	IR Intensity (KM/Mole)
-19.3792	2.4966
18.8718	0.0791
22.4158	0.2640
27.2017	1.0858
34.9373	1.3575
43.6808	0.5723
49.2613	0.2859
51.2994	1.3401
55.1148	2.3502

Frequency (cm^{-1})	IR Intensity (KM/Mole)
75.0995	0.8467
80.2958	3.9859
104.1659	1.1381
111.4208	6.4640
119.7616	12.3391
160.8993	5.9990
166.7058	2.8459
188.7208	0.6208
194.7405	0.4523
208.3677	1.6943
229.1077	1.0447
241.5317	3.5051
256.7754	3.7656
262.6989	0.7991
287.7884	8.9258
328.6199	1.8596
340.8459	14.5467
349.4056	9.1276
356.6735	7.1761

Frequency (cm^{-1})	IR Intensity (KM/Mole)
378.3240	13.1193
393.9636	5.1004
413.8025	29.8351
415.6498	0.2302
425.8726	1.1740
447.2858	59.9980
461.2978	10.8288
469.5813	36.6304
505.7031	14.3439
539.5209	62.9109
560.5167	99.5443
574.3219	33.9735
596.4712	70.7851
610.5128	32.7000
615.1492	14.0207
631.7213	12.2551
634.4952	0.9399
638.2579	8.0972
688.4722	119.0875

Frequency (cm⁻¹)	IR Intensity (KM/Mole)
705.7756	28.8047
713.2761	74.0777
735.8450	14.0390
749.1550	0.8268
768.5506	56.0397
770.2911	26.7681
786.5313	3.3182
797.4760	3.6060
814.9118	20.1016
829.1563	7.5158
861.6830	0.7953
866.9092	10.9873
883.8273	26.0292
892.7181	24.7926
922.4863	87.3138
935.1493	20.4099
935.8647	5.7459
952.6018	7.9721
963.7395	35.6437

Frequency (cm^{-1})	IR Intensity (KM/Mole)
965.4459	3.6322
968.9988	173.8463
989.4220	18.7242
992.4759	1.2435
1000.7668	0.5207
1007.4870	0.7012
1016.0542	0.1145
1035.2405	31.3793
1048.4579	4.6682
1049.0949	17.6649
1067.8664	41.3466
1084.9705	5.0425
1100.5239	51.7719
1105.9619	44.1583
1110.1365	5.4674
1113.7893	30.4778
1165.1020	19.5352
1175.6911	0.4134
1186.5000	17.7360

Frequency (cm⁻¹)	IR Intensity (KM/Mole)
1187.1885	86.3301
1196.7545	1.8268
1199.8509	3.3436
1205.8057	49.3126
1215.1298	7.0564
1219.6246	3.1747
1220.9827	1.1973
1266.0839	4.4186
1309.2837	57.0178
1316.1828	78.3694
1317.3980	11.1299
1320.4578	5.1819
1339.2597	14.4439
1340.4573	3.7810
1356.0204	105.6584
1365.0586	11.2753
1376.6466	3.7765
1388.3434	7.1570
1395.4692	5.6377

Frequency (cm^{-1})	IR Intensity (KM/Mole)
1412.6338	133.5931
1418.3008	17.4034
1420.8425	93.5158
1442.5945	89.1355
1464.3487	66.7550
1478.9771	18.7665
1479.2812	19.2618
1481.3224	14.8072
1485.5377	14.2142
1491.2420	34.9686
1493.5836	7.1072
1498.4279	41.7839
1508.1586	39.6096
1508.5938	38.9225
1522.7888	529.9595
1523.6762	104.9203
1576.3210	5.2267
1614.6907	6.8375
1618.5947	2.7651

Frequency (cm⁻¹)	IR Intensity (KM/Mole)
1639.7424	15.7350
1677.2488	770.1742
1684.3047	768.8448
2980.6159	84.2840
3000.7771	8.0638
3003.8906	47.2857
3013.8713	66.9429
3036.4130	41.1315
3039.1966	48.8275
3049.0322	31.8437
3060.2724	8.8338
3068.2075	121.8295
3073.4139	69.9541
3100.2355	1.2234
3111.0905	18.7660
3114.3779	14.7724
3150.3182	10.8265
3160.9500	1.3935
3162.2976	5.8807

Frequency (cm⁻¹)	IR Intensity (KM/Mole)
3165.1833	19.1187
3170.8217	32.9220
3179.1899	35.0626
3182.6667	27.8139
3191.4178	9.1699
3559.9915	114.1228
3570.2118	42.6946
3825.2403	25.0710

Bortezomib (de2) in methanol

Frequency (cm^{-1})	IR Intensity (KM/Mole)
18.3902	1.9174
20.4002	1.2018
24.0956	1.7061
25.2193	1.3592
34.4978	0.7607
41.4455	0.2774
45.1101	2.0685
47.9972	1.7512

Frequency (cm^{-1})	IR Intensity (KM/Mole)
57.0879	0.3756
67.9208	8.7972
75.0494	0.3644
99.7931	2.1171
104.9577	7.7711
110.7540	11.7483
159.2427	4.9520
159.3521	2.7365
182.7609	2.9969
194.9310	0.5141
196.7949	2.3557
226.7456	0.9139
242.7305	2.4628
253.8371	1.5931
260.3837	3.7990
284.7420	8.8110
326.8294	2.0264
341.6027	13.0314
345.4699	26.6393

Frequency (cm^{-1})	IR Intensity (KM/Mole)
356.2001	1.1441
372.6404	13.4430
388.3267	4.1065
412.4763	33.3710
413.6681	0.2920
416.5999	1.0070
445.7943	80.0612
460.1718	4.0271
469.5082	51.3909
506.0104	16.0876
537.7838	86.1928
553.6107	79.4557
568.7809	56.9604
602.5227	108.8876
605.7789	16.3973
610.2966	39.0679
628.7444	11.7789
633.8982	0.7694
635.2206	8.5994

Frequency (cm⁻¹)	IR Intensity (KM/Mole)
682.3144	167.5239
697.5647	20.7729
711.1758	80.1760
736.1966	10.8358
747.0472	0.9259
767.0189	29.2022
768.6148	64.6974
786.0111	3.1798
795.5053	3.6564
813.3140	21.0217
827.6775	6.0641
859.1891	0.0855
867.5595	9.6123
883.6116	35.5461
887.1144	20.8687
919.3865	73.6813
933.6807	37.0298
935.7345	5.0336
951.9756	15.7276

Frequency (cm^{-1})	IR Intensity (KM/Mole)
962.8311	13.5730
968.1382	30.8189
970.0137	208.3669
988.9071	23.5555
992.0219	0.3931
1001.3785	0.3788
1009.2320	0.1535
1015.1872	0.0745
1035.6295	36.1174
1047.1589	13.3140
1049.9020	15.2381
1068.3632	44.0470
1085.3315	4.8481
1100.2450	42.6870
1105.2559	33.4874
1108.3121	45.7801
1112.5398	45.9078
1165.0292	36.4711
1174.9871	0.5648

Frequency (cm^{-1})	IR Intensity (KM/Mole)
1184.5737	12.4005
1188.5597	91.9166
1196.2937	2.7774
1197.7654	6.5190
1206.8577	57.3243
1211.0466	12.4967
1218.6051	4.1174
1220.5185	2.3914
1261.7165	8.5199
1311.0358	99.3806
1316.8548	47.4608
1318.7470	23.6086
1322.2813	8.4054
1337.6686	25.7164
1340.5133	1.4867
1356.2710	75.2122
1368.0608	63.3791
1370.6456	9.9444
1386.7082	6.0379

Frequency (cm⁻¹)	IR Intensity (KM/Mole)
1393.4231	8.8149
1412.8540	40.3012
1413.4617	108.5348
1420.4087	110.3796
1441.6621	107.7594
1460.4554	145.2347
1467.8881	68.2989
1476.2020	28.3467
1480.9710	19.4550
1481.7469	19.1147
1486.0801	19.8461
1491.1794	152.8080
1493.0053	53.8878
1499.0930	480.7319
1504.3718	46.3774
1510.7688	68.9714
1522.7876	19.4201
1574.8100	4.7981
1613.6931	13.9673

Frequency (cm^{-1})	IR Intensity (KM/Mole)
1618.1944	1.7406
1638.2896	18.5220
1666.9843	1056.1287
1672.4453	753.1351
2988.4623	83.6317
2998.5397	10.9053
3006.1133	57.0028
3014.4341	63.6422
3037.2561	62.5476
3045.2543	53.3688
3052.8222	33.6808
3062.9010	5.4569
3068.6076	154.6303
3075.7730	75.5247
3101.0926	2.1210
3103.5522	30.9359
3117.0784	22.0019
3153.2335	12.6667
3163.0499	0.4294

Frequency (cm^{-1})	IR Intensity (KM/Mole)
3167.4965	0.8114
3171.7079	24.5822
3172.5586	31.9528
3182.7872	32.3508
3183.1404	34.7667
3190.1863	12.9381
3565.3874	102.6392
3569.2255	60.4992
3819.9231	29.9917

Bortezomib (de2) in water

Frequency (cm^{-1})	IR Intensity (KM/Mole)
16.8208	1.2538
18.8830	1.9739
23.8693	1.0071
23.8760	2.0062
33.7625	1.7644
36.3265	0.8249
43.7079	0.5876

Frequency (cm^{-1})	IR Intensity (KM/Mole)
48.5730	1.5387
58.5032	0.3569
71.9649	9.2635
75.6601	0.2051
99.7314	2.7623
105.4779	6.2390
110.6195	12.2975
160.0781	5.9680
160.5654	2.3437
185.3913	2.0525
194.9835	0.4349
198.2613	3.0424
227.3826	0.7858
243.4909	2.4184
254.5597	1.6910
260.3855	3.6046
285.2421	8.7635
327.4023	3.0878
342.3315	14.6386

Frequency (cm^{-1})	IR Intensity (KM/Mole)
346.7592	26.2709
355.3608	0.7250
371.1792	13.1987
389.1375	3.9167
412.6119	34.2975
413.4614	0.1467
418.6097	1.0422
446.5356	82.6809
460.4360	4.1682
469.4909	54.9178
506.0300	16.0759
537.4826	88.3838
553.5641	77.9787
568.8249	57.7574
603.8696	94.3933
606.1031	32.2913
611.8363	34.9037
628.9821	14.1824
633.8431	1.0378

Frequency (cm⁻¹)	IR Intensity (KM/Mole)
635.3471	8.7907
683.8306	165.1810
698.7949	28.3954
710.9585	83.1180
736.6866	11.0905
746.9709	1.3475
766.7073	28.7374
768.3471	66.3364
786.1147	3.3237
795.4893	3.9452
813.4757	21.4463
827.9504	6.4275
858.4887	0.0591
867.6864	8.7868
883.7491	36.1362
887.8080	20.7473
918.9099	74.6387
933.6898	41.0968
935.2218	5.2618

Frequency (cm^{-1})	IR Intensity (KM/Mole)
951.8831	18.5522
962.6446	18.0153
968.4969	116.2519
969.6390	118.9053
989.9162	23.1371
991.6577	0.3414
1002.0036	0.3831
1009.3661	0.1273
1015.1007	0.0616
1035.5595	36.4914
1046.9589	12.7516
1050.8138	17.3930
1068.1631	44.1285
1085.3951	5.9361
1100.1061	40.1873
1105.2342	34.1104
1108.4097	48.3506
1112.8624	51.3007
1165.1881	37.6500

Frequency (cm^{-1})	IR Intensity (KM/Mole)
1174.9714	0.5975
1184.4457	12.0121
1188.6485	93.0635
1196.2419	3.0030
1197.5611	7.3957
1206.9347	57.4820
1210.7425	13.3578
1218.6832	4.2225
1220.4844	2.5042
1263.7658	8.6936
1311.2295	96.3195
1316.1917	60.4603
1318.7534	18.9178
1322.7485	6.1321
1337.4761	27.7020
1340.0174	2.2099
1356.0762	70.6841
1368.8332	63.8144
1370.8399	15.1824

Frequency (cm^{-1})	IR Intensity (KM/Mole)
1386.5521	5.6054
1393.1679	9.1239
1412.4793	9.1980
1413.4443	141.2281
1420.5801	111.6330
1441.8112	111.3209
1460.7774	163.1215
1469.8153	63.4932
1475.1802	31.6907
1480.4726	19.8155
1481.7195	19.6547
1485.8787	25.3758
1490.4210	201.0743
1493.2142	49.4570
1497.5230	448.9848
1503.5616	30.7792
1510.9743	70.0931
1522.8350	19.4499
1574.6080	4.8460

Frequency (cm⁻¹)	IR Intensity (KM/Mole)
1613.5893	14.5312
1618.1013	1.5637
1638.1946	19.0397
1665.6199	1092.3498
1671.2634	750.5791
2989.4761	82.9248
2998.3795	10.8109
3006.3194	56.5357
3014.0199	63.0156
3035.4942	70.6055
3046.1163	53.8687
3052.6043	33.9062
3062.9774	5.0272
3068.5659	158.3635
3076.1807	75.3853
3100.5822	2.2986
3102.8726	32.5220
3117.1124	22.8863
3153.5264	12.8731

Frequency (cm^{-1})	IR Intensity (KM/Mole)
3163.2302	0.3168
3168.1995	0.6934
3172.6258	26.3434
3172.7171	30.1112
3183.0880	38.2392
3183.4195	30.0022
3190.0980	13.3923
3565.8782	102.8905
3568.4249	62.2450
3818.8238	31.0903

Warhead model calculations at level of theory - B3LYP/6-311+G(d,p)

Warhead model in gas phase

Frequency (cm^{-1})	IR Intensity (KM/Mole)
31.0727	0.1464
39.7217	0.5871
53.7429	1.9442
62.9679	0.4861
77.4200	0.0159

Frequency (cm^{-1})	IR Intensity (KM/Mole)
100.4139	2.1450
132.9110	1.2081
189.2998	0.3503
197.7445	0.6121
229.4751	0.0646
255.5322	0.2553
272.1136	5.1380
327.2591	2.4303
354.5766	3.9565
385.5844	0.2494
415.8705	1.2695
423.3398	0.6364
456.2064	55.0454
475.5871	32.6395
536.2357	12.1323
539.6405	22.3409
559.9901	123.4821
586.2980	104.7726
649.8301	65.2241

Frequency (cm^{-1})	IR Intensity (KM/Mole)
703.5396	23.7593
753.9562	4.9854
814.0404	2.0902
844.0219	7.8073
894.6944	11.0186
929.2745	8.9236
936.2654	152.2444
942.4116	5.0654
964.4650	0.1332
993.2221	1.0453
1008.5929	125.3569
1028.4618	5.1767
1050.4287	3.9888
1110.9870	6.0670
1130.6215	10.9255
1165.0061	9.2384
1187.7040	6.3751
1222.0080	6.1122
1268.4961	19.6609

Frequency (cm^{-1})	IR Intensity (KM/Mole)
1290.9800	48.7442
1337.5806	15.9423
1348.9587	175.1977
1362.2516	30.6413
1383.9415	38.8437
1385.9018	224.3139
1396.8987	122.1881
1402.4486	79.3036
1407.4166	74.0388
1423.7403	13.5323
1471.8212	8.0868
1482.6504	2.8765
1484.7067	5.1286
1490.3427	10.3995
1494.5906	7.6880
1500.5028	9.1963
1506.6201	7.0282
1516.7635	11.4809
1729.3844	520.8619

Frequency (cm^{-1})	IR Intensity (KM/Mole)
2990.4069	19.9760
3011.1921	17.5442
3011.8337	9.6640
3016.0408	30.2163
3023.8676	33.9149
3042.1191	10.8344
3053.3289	20.6405
3071.3944	10.2371
3078.8046	58.4120
3083.1354	43.7512
3086.2794	44.0569
3102.4361	14.1730
3143.1183	10.6154
3589.7274	37.9665
3857.2806	15.1041
3861.7636	84.5576

Warhead model in diethyl ether

Frequency (cm^{-1})	IR Intensity (KM/Mole)
36.8937	0.2938
46.9903	0.3575
61.9414	4.5120
72.7924	0.2223
90.1572	0.2460
104.6732	2.1235
133.3055	1.4175
193.1962	0.3369
198.7839	0.7276
229.0837	0.0549
254.8103	0.0870
273.2874	7.7304
329.5093	2.9852
353.6050	4.8532
385.9149	0.4564
423.3332	1.3140
454.0220	66.3728
465.5048	27.1977

Frequency (cm^{-1})	IR Intensity (KM/Mole)
482.3648	30.3247
534.6328	13.1270
542.9703	42.1705
560.0485	159.1361
582.8222	142.6804
656.9833	90.2526
701.7049	26.0624
753.2787	5.3070
815.8133	2.7892
846.1140	9.5935
895.5532	16.6996
926.1362	7.3483
942.7853	6.2316
956.4336	224.3023
963.6905	0.0865
994.6659	2.7995
1008.0677	140.0675
1031.6554	9.5237
1052.5727	6.4996

Frequency (cm⁻¹)	IR Intensity (KM/Mole)
1110.6967	8.7944
1125.9343	15.4500
1161.9793	16.0780
1185.4335	7.9277
1221.2810	11.0286
1266.4157	34.0598
1290.5417	77.1891
1337.1358	28.4656
1352.2358	246.1427
1360.8516	18.8444
1379.8519	132.4953
1382.5874	270.2261
1394.0323	55.5759
1398.8813	80.8713
1410.3056	184.5876
1418.8623	12.8151
1467.9416	12.8222
1478.4219	6.0666
1481.0600	2.3035

Frequency (cm^{-1})	IR Intensity (KM/Mole)
1485.4170	10.7886
1489.8738	17.9875
1495.1666	9.0272
1499.2004	17.6724
1508.8350	13.7335
1678.9251	786.7424
3004.6059	20.7777
3008.4055	1.3419
3010.4378	40.2836
3016.3767	44.5761
3023.7709	56.2290
3046.2851	8.3516
3054.0664	30.7971
3070.0845	22.6251
3077.3333	81.9283
3081.2854	66.0641
3085.9935	54.9696
3109.6151	14.1893
3142.0299	15.7399

Frequency (cm^{-1})	IR Intensity (KM/Mole)
3583.0336	56.5893
3836.5318	16.5484
3843.1283	157.5269

Warhead model in methanol

Frequency (cm^{-1})	IR Intensity (KM/Mole)
35.9300	0.1661
39.2871	1.1474
60.4770	4.6874
72.8852	0.2144
83.9215	0.2310
101.5267	2.4791
133.8146	1.4905
195.4546	0.3011
198.3687	0.8747
230.1424	0.0613
255.4248	0.0553
274.5466	8.0201
331.5362	2.8105

Frequency (cm^{-1})	IR Intensity (KM/Mole)
357.1098	6.8997
386.3465	0.5218
422.8533	1.7048
459.3313	88.7768
479.2176	37.9549
488.7969	14.4653
539.3162	58.2005
547.0198	2.3740
559.6038	222.7157
583.1127	132.0695
654.1510	105.9886
702.0212	26.3541
755.3164	4.7364
817.1420	2.7812
847.2380	11.1069
896.0094	20.3964
925.2557	8.0030
942.6111	5.9683
963.2337	108.6516

Frequency (cm^{-1})	IR Intensity (KM/Mole)
963.7089	154.0071
995.8662	1.4618
1029.1850	89.9944
1042.1697	116.1489
1052.2157	9.3084
1110.7873	10.8186
1123.5439	18.1571
1162.9115	13.4784
1184.6880	8.6680
1222.8771	8.7799
1267.1245	24.3410
1293.8722	84.5558
1336.9594	26.2792
1351.3283	320.3612
1361.3782	22.4028
1376.9691	203.7257
1381.8815	214.0267
1393.9921	21.6545
1397.7309	109.8804

Frequency (cm^{-1})	IR Intensity (KM/Mole)
1412.0467	242.7484
1416.9781	10.7052
1464.2978	18.0258
1475.3188	8.7229
1478.5478	2.1286
1482.5989	10.4451
1487.8847	20.3923
1491.6728	7.4921
1495.7272	30.1809
1505.2690	14.5675
1656.7103	916.6090
3005.6134	11.0755
3009.4561	29.4270
3012.2800	40.8678
3016.8776	47.4057
3024.7543	72.3161
3047.6967	8.1809
3054.5342	37.2584
3069.2312	31.9080

Frequency (cm⁻¹)	IR Intensity (KM/Mole)
3076.1212	102.4873
3080.0035	77.0014
3085.8874	61.5859
3111.4588	14.5515
3142.6175	17.9131
3579.6911	65.4638
3834.2434	191.1569
3835.1076	17.6170

Warhead model in water

Frequency (cm^{-1})	IR Intensity (KM/Mole)
30.1573	0.5853
38.8673	0.8816
60.1788	4.5741
72.6951	0.2539
82.7209	0.2740
101.2996	2.4711
133.5061	1.5024
195.8292	0.2926

Frequency (cm⁻¹)	IR Intensity (KM/Mole)
198.2173	0.8921
230.3486	0.0634
255.5143	0.0574
274.7353	7.9653
331.6925	2.8118
357.5114	7.1170
386.4072	0.4978
422.8600	1.7146
459.9402	91.0199
481.2687	42.1753
490.3890	9.9186
539.0664	61.8229
548.1214	1.4884
559.8451	229.6836
583.3864	129.4421
652.9571	107.7863
702.0553	26.2408
755.6577	4.6777
817.3172	2.7739

Frequency (cm⁻¹)	IR Intensity (KM/Mole)
847.3476	11.3793
896.0757	20.8836
925.1845	8.0664
942.5798	5.9564
963.3201	24.9868
964.2455	241.8272
995.9349	1.6101
1030.0380	72.3643
1046.5627	142.5658
1052.0960	10.4514
1110.7366	11.1757
1123.2710	18.3424
1163.2923	12.9768
1184.6435	8.7722
1223.1140	8.3290
1267.2681	22.4170
1294.6299	83.8400
1337.0071	25.2965
1351.1909	329.1499

Frequency (cm⁻¹)	IR Intensity (KM/Mole)
1361.5886	21.4327
1376.5750	218.1818
1381.7904	202.8799
1393.9999	17.4179
1397.7021	114.2243
1412.3591	249.0458
1416.8043	10.0829
1463.7261	18.9534
1474.8825	9.2344
1478.2658	2.1086
1482.2570	10.3356
1487.6695	19.9631
1491.2861	7.1653
1495.3914	32.2830
1504.9697	14.6636
1654.7393	929.7643
3005.3704	11.5084
3009.4097	32.9692
3012.8482	41.2520

Frequency (cm^{-1})	IR Intensity (KM/Mole)
3017.0222	46.2353
3025.0103	73.7710
3047.6814	8.3546
3054.5955	37.9710
3069.1291	33.1994
3075.9721	104.9467
3079.8445	78.2260
3085.7479	62.5093
3111.2485	14.6076
3142.4960	18.0725
3579.4199	66.1073
3833.2530	188.3122
3834.6367	23.9513

Warhead model (de1) in gas phase

Frequency (cm^{-1})	IR Intensity (KM/Mole)
36.2686	0.4962
57.7034	1.2404
65.5820	2.5540

Frequency (cm^{-1})	IR Intensity (KM/Mole)
80.8309	2.3203
95.4153	0.0832
110.3289	4.8415
134.5854	3.6397
190.0788	1.3539
203.0152	0.3500
232.8480	0.9580
265.5911	0.4305
269.6280	29.2755
325.4107	7.0973
345.3803	4.8200
388.8311	0.4950
422.4647	0.1796
472.1740	33.3657
477.3435	12.6650
535.8641	21.5884
553.6547	62.8277
583.9788	12.8034
602.5130	49.5925

Frequency (cm^{-1})	IR Intensity (KM/Mole)
711.6954	14.7046
737.8114	6.5152
792.7160	31.0551
809.6103	34.9552
846.8997	38.4082
897.1328	33.4994
931.2870	6.7009
938.3707	4.6121
964.4736	1.2522
980.0624	184.5953
991.8286	63.8013
1026.9026	15.1548
1049.3692	2.8497
1097.9590	10.3573
1120.7635	56.5494
1128.6133	6.9196
1162.3961	11.7283
1186.5994	9.9862
1216.3657	24.2738

Frequency (cm^{-1})	IR Intensity (KM/Mole)
1267.2251	0.7368
1309.3601	6.5158
1339.0825	8.4372
1363.1601	13.2192
1374.1542	0.7594
1385.0797	21.9874
1392.0112	3.3067
1403.7677	96.3797
1419.2566	12.8918
1446.9478	102.3759
1471.4467	18.6221
1481.9365	5.5420
1485.9002	25.0377
1490.4092	1.9384
1496.2902	5.7397
1501.2779	4.1578
1519.6344	12.1374
1548.4413	342.9907
1684.0472	705.5818

Frequency (cm^{-1})	IR Intensity (KM/Mole)
2962.7066	34.9545
2978.5390	96.7331
2992.2985	36.6577
2998.2957	74.3301
3002.0707	14.4660
3026.2843	24.6603
3034.2064	18.3307
3045.2175	1.7343
3056.0600	119.3094
3058.3346	70.7192
3095.8359	2.7819
3096.1815	21.1081
3124.2694	26.1753
3442.2025	188.6301
3823.6978	6.6657

Warhead model (de1) in diethyl ether

Frequency (cm^{-1})	IR Intensity (KM/Mole)
49.4650	1.7705

Frequency (cm^{-1})	IR Intensity (KM/Mole)
53.9149	1.1176
74.1484	5.0006
86.9486	0.2994
112.4329	0.4002
121.8456	5.4379
142.1492	5.0405
190.9064	1.7395
208.3878	0.8158
231.7935	1.6562
261.7060	0.0491
274.5182	39.0373
331.4356	8.0968
347.9432	7.5636
392.3758	0.9259
420.4989	0.1566
471.5180	49.9207
480.6234	23.6590
539.9365	23.0326
546.0073	123.9299

Frequency (cm^{-1})	IR Intensity (KM/Mole)
577.2865	25.6583
593.0201	38.5643
709.8498	20.9793
738.9084	8.1279
768.0887	108.9673
805.0793	2.3776
849.1540	29.5332
896.9431	50.7431
926.1942	6.1034
941.1453	11.6744
963.8304	0.2532
978.2494	281.4642
993.3660	37.0283
1029.8561	22.2586
1053.0177	5.0348
1101.8488	6.6769
1116.2600	103.3042
1126.8634	13.5311
1162.3064	18.4311

Frequency (cm^{-1})	IR Intensity (KM/Mole)
1186.2817	12.6468
1221.1277	30.6299
1265.3433	2.1746
1311.3082	9.6190
1342.8475	9.5318
1368.4682	9.8084
1373.5532	22.4682
1388.1642	20.4534
1393.1135	5.8443
1407.8220	159.1949
1414.6014	13.4600
1449.6130	119.1204
1469.2719	41.1892
1480.5488	15.5224
1483.6484	71.5896
1486.1990	9.9588
1493.0245	3.5516
1497.1754	10.7296
1505.7656	278.4072

Frequency (cm^{-1})	IR Intensity (KM/Mole)
1509.4271	219.5869
1645.8473	972.5955
2979.7421	43.0417
2996.5439	75.0250
3001.5648	74.3043
3001.8017	0.9694
3009.7289	71.6256
3032.8962	48.8983
3042.2004	13.6493
3056.1447	4.8115
3063.7757	142.1656
3069.5459	75.9342
3106.2223	18.2859
3106.8143	15.0432
3134.4425	23.2294
3484.6322	193.8966
3823.7626	19.6852

Warhead model (de1) in methanol

Frequency (cm^{-1})	IR Intensity (KM/Mole)
28.9853	1.0526
54.9519	0.3978
69.8565	7.8487
84.0823	0.7700
101.6821	0.5111
111.1034	6.0824
132.9560	6.3410
188.2432	1.4725
205.1612	1.0168
233.8292	1.3907
254.8061	0.3351
272.3648	44.9182
330.3273	9.7417
347.0285	9.2273
389.8213	1.1805
421.9201	0.6106
462.9350	58.9750
476.9189	47.8324

Frequency (cm^{-1})	IR Intensity (KM/Mole)
536.0796	131.2759
540.0437	31.2357
573.5346	18.8854
587.5648	49.9892
707.6619	51.0325
729.8938	62.7121
745.5249	61.9087
805.6706	2.6815
848.7536	27.8098
895.8095	58.7313
924.5972	7.9121
941.1452	10.6892
963.0465	0.6154
975.7286	307.2115
993.1751	36.2413
1031.2056	24.9016
1053.0093	7.4282
1102.9877	8.1883
1113.4264	136.4811

Frequency (cm^{-1})	IR Intensity (KM/Mole)
1125.5901	16.4427
1162.0324	23.0176
1185.4682	14.2532
1223.7198	33.1110
1265.1651	3.0158
1312.4704	9.8822
1339.2794	10.8561
1368.2309	9.7230
1375.0878	32.1764
1388.4394	19.6153
1393.0760	7.9868
1407.1621	188.4775
1413.0723	18.4944
1447.9337	132.4258
1465.4995	111.2569
1476.4729	335.8037
1479.1173	62.6513
1482.6941	10.1885
1488.0147	159.3441

Frequency (cm^{-1})	IR Intensity (KM/Mole)
1490.3766	98.5591
1493.3792	7.9364
1503.4981	24.2415
1627.5647	1093.8255
2983.5914	50.5916
3000.5441	5.6588
3002.5179	49.4365
3004.8767	98.2276
3012.1096	83.5879
3035.4105	61.2521
3044.8883	11.6119
3060.4075	5.8021
3066.5638	161.2077
3073.5445	78.3684
3097.8683	29.6276
3109.1682	17.1189
3136.8802	22.8841
3493.5762	200.3397
3817.9351	25.4949

Warhead model (de1) in water

Frequency (cm^{-1})	IR Intensity (KM/Mole)
27.9828	0.8116
54.8019	0.2880
70.7563	9.1103
83.8930	0.8448
101.8949	0.3923
108.8121	5.4785
132.9517	6.4824
189.0704	1.4402
204.1456	1.0367
234.5576	1.2642
256.4046	0.5329
272.2985	45.2805
330.3388	9.6480
347.5155	9.6198
388.9654	1.2641
421.8680	0.6133
462.6901	59.6875
477.1898	49.6358

Frequency (cm⁻¹)	IR Intensity (KM/Mole)
535.6723	133.2307
540.5438	31.2906
573.0851	19.6338
587.0338	51.1319
707.5255	57.4199
728.0714	67.0441
744.9619	55.7513
805.6809	2.8531
848.4388	27.6240
895.5332	59.3599
924.2249	8.3201
940.9147	10.2718
962.7981	0.6317
976.0590	309.5957
992.9318	37.8957
1031.5185	25.2935
1052.9446	7.8012
1103.2637	8.2864
1113.4772	139.2499

Frequency (cm^{-1})	IR Intensity (KM/Mole)
1125.7652	16.7632
1162.0345	23.7937
1185.1872	14.5257
1224.0820	33.2735
1264.9731	3.1859
1312.2861	9.8994
1338.4390	10.9406
1368.1163	9.9222
1375.1560	33.5478
1388.2163	18.7601
1392.9035	8.8851
1407.0890	190.9953
1412.6357	18.6318
1447.7754	135.1229
1464.9320	133.4422
1475.2239	405.5729
1478.8934	36.6931
1482.5314	8.6172
1487.1867	152.5934

Frequency (cm⁻¹)	IR Intensity (KM/Mole)
1489.9052	59.4815
1492.7940	9.3892
1503.5553	23.1756
1625.7380	1105.4132
2983.5035	52.0233
3000.0840	5.8116
3002.9904	47.2713
3005.1724	101.5102
3012.3159	84.4713
3035.7788	62.0962
3045.0848	11.4665
3060.8709	5.7892
3066.8491	163.7453
3073.8917	78.7398
3095.9945	31.6716
3109.3276	17.0219
3136.9634	22.9340
3494.8368	201.2313
3817.4771	26.0257

Warhead model (de2) in gas phase

Frequency (cm^{-1})	IR Intensity (KM/Mole)
31.3837	1.1433
55.2012	0.3996
59.8261	3.2902
73.1262	4.8502
92.0661	0.4769
103.7753	4.2205
138.6305	3.2690
193.3997	2.2281
199.5302	0.2803
229.7419	0.1802
253.2053	0.1140
268.4242	14.8714
324.3206	4.1280
360.2895	1.9400
386.6849	0.9914
423.8512	0.4428
449.8636	51.0561
474.1685	17.9593

Frequency (cm^{-1})	IR Intensity (KM/Mole)
522.1774	4.1546
552.7457	59.6120
576.3779	9.0506
595.8924	61.2287
703.2843	22.9559
726.2192	6.8162
745.3843	53.1442
799.4032	0.4529
836.4463	16.2361
899.7083	10.2278
920.9473	77.8349
931.8848	5.2959
962.6230	49.3029
967.2470	115.9917
989.4283	25.3687
1027.0010	21.9616
1050.6479	3.0752
1097.8825	41.9585
1106.7204	15.1340

Frequency (cm^{-1})	IR Intensity (KM/Mole)
1120.1453	5.4354
1163.4571	13.5300
1185.9564	7.4344
1211.3352	4.8701
1269.4656	1.5720
1312.4053	28.5394
1337.2823	10.3334
1364.5493	14.3491
1375.9710	3.2351
1382.7457	10.4438
1392.5545	6.8969
1413.4046	147.9590
1419.1130	2.0704
1449.7740	51.0886
1471.0103	19.6087
1481.2478	5.4691
1485.9757	17.0566
1488.8227	2.4377
1493.9544	13.0579

Frequency (cm^{-1})	IR Intensity (KM/Mole)
1500.6803	7.3873
1512.9353	15.5311
1565.4970	438.6284
1685.4720	628.8815
2968.0714	52.3381
2991.3054	46.4429
2994.0279	34.3500
2995.3921	54.4652
3003.3279	65.8959
3036.4190	16.5868
3037.5243	14.0578
3052.4600	28.1967
3058.1753	63.9950
3064.4996	81.8258
3099.1023	19.1959
3107.3682	9.4769
3127.8493	23.1816
3558.1510	81.0446
3832.0185	8.9489

Warhead model (de2) in diethyl ether

Frequency (cm^{-1})	IR Intensity (KM/Mole)
53.9148	0.8007
63.4023	2.2910
71.7936	3.0073
85.2589	5.3482
101.8274	0.2403
128.9759	8.5634
149.5580	4.8083
215.5359	4.4760
229.9122	1.1330
244.8986	0.1555
277.7650	15.8227
327.1521	5.2972
347.8518	2.1906
368.1958	2.6635
399.5669	1.0399
439.6353	0.6841
463.8647	71.9627
480.1908	37.4498

Frequency (cm^{-1})	IR Intensity (KM/Mole)
527.0882	7.9624
551.3445	112.0891
577.3200	6.3666
591.8033	68.5863
708.1861	47.7477
723.6890	47.9031
741.9225	32.2727
808.4186	1.8949
844.5488	23.3009
905.5777	11.3529
928.0803	96.7245
937.0137	9.3233
965.7710	37.7585
971.4591	186.6976
1000.5072	25.1475
1030.3504	31.1458
1053.6148	5.3094
1104.9919	51.6537
1115.9428	31.3174

Frequency (cm^{-1})	IR Intensity (KM/Mole)
1120.3261	20.2470
1170.5984	28.0776
1192.7463	8.8286
1220.2313	9.1082
1284.3306	2.9417
1313.1554	52.2117
1341.4659	7.7326
1372.1200	26.0383
1378.0758	11.2462
1389.9361	11.4096
1396.4284	5.1452
1413.8532	185.1425
1422.8496	13.1431
1450.3370	113.8582
1470.7083	22.4931
1486.0108	3.0912
1486.3103	32.7158
1489.6799	27.3409
1498.2515	9.5387

Frequency (cm^{-1})	IR Intensity (KM/Mole)
1508.3956	229.9163
1517.8591	7.7470
1523.0913	439.7048
1649.5952	861.0306
2988.7157	50.5930
2996.6626	14.3901
3003.6262	56.7627
3010.1746	97.6724
3015.2933	37.3370
3040.2868	36.1029
3043.2533	12.9077
3061.3446	16.4374
3068.3552	86.5810
3069.5008	113.0023
3107.3865	17.4491
3111.2538	23.2290
3135.3105	21.2696
3573.0726	92.3844
3834.9417	22.3171

Warhead model (de2) in methanol

Frequency (cm^{-1})	IR Intensity (KM/Mole)
36.4568	1.1964
55.5129	0.7278
69.2089	5.5495
76.4782	6.8358
88.8814	0.3249
107.0070	8.9536
140.0918	6.7323
196.6174	3.2286
202.5155	0.6796
232.5590	0.2526
256.6734	0.1323
273.4854	22.4704
333.5340	5.4191
362.4276	2.8406
388.4244	1.0005
426.1887	1.0105
454.7892	87.8051
472.6189	59.5907

Frequency (cm^{-1})	IR Intensity (KM/Mole)
526.9220	8.1026
543.3654	138.8507
563.9340	5.1386
586.3923	71.0844
687.8676	121.4263
707.7056	8.5588
737.7686	17.7709
806.2043	2.1358
842.0725	16.9700
899.5300	16.4929
921.2658	74.6681
933.1893	40.1171
961.7426	43.2179
967.7332	213.9324
994.4147	25.6554
1030.0465	33.6832
1053.3920	7.9494
1101.8446	93.1936
1111.4426	24.5485

Frequency (cm^{-1})	IR Intensity (KM/Mole)
1117.9199	14.7376
1166.6058	43.9730
1185.4585	10.9827
1219.6914	11.8545
1270.8713	5.7978
1311.4002	76.3589
1337.0699	7.0267
1372.4106	25.3810
1373.5621	19.2427
1387.1883	7.1256
1393.1437	11.0347
1411.4478	150.7828
1413.6944	65.8794
1449.6363	197.5705
1466.6533	31.4212
1477.7366	3.0984
1481.8336	98.6417
1482.4565	86.2327
1486.9721	87.1485

Frequency (cm^{-1})	IR Intensity (KM/Mole)
1491.4827	64.2826
1499.0948	459.3974
1505.3557	43.4711
1633.3793	967.3668
2995.3276	33.0513
2998.4266	15.1226
3005.0309	49.7548
3009.0523	122.1193
3014.5511	52.3050
3038.4338	60.1494
3045.7677	10.3664
3062.1985	8.1697
3068.0119	158.3921
3073.5180	79.5481
3098.9645	35.0245
3110.1502	16.7114
3138.1184	22.1255
3568.5511	100.5511
3823.3197	28.0745

Warhead model (de2) in water

Frequency (cm^{-1})	IR Intensity (KM/Mole)
35.2590	1.1743
55.1889	0.8245
68.9999	5.3713
74.4317	6.9861
88.5879	0.3374
107.0488	9.2777
139.4950	7.1059
195.6084	3.4230
202.0161	0.4864
232.6322	0.2494
257.7980	0.1216
273.3448	22.7924
333.4967	5.3261
361.7568	2.8863
388.1662	1.0034
425.0071	1.0383
453.8727	90.1197
472.4122	61.6788

Frequency (cm^{-1})	IR Intensity (KM/Mole)
527.1234	8.5203
542.5297	142.4548
563.3271	4.8654
585.9213	70.5043
686.7682	123.1879
707.2616	8.8425
737.5567	17.6751
806.2141	2.1445
842.2011	16.9255
899.3767	17.1372
920.8821	74.3054
933.2122	43.0614
961.5981	47.1392
967.3975	211.8948
993.5972	25.6769
1030.1748	33.7998
1053.2756	8.2977
1101.4811	100.5573
1111.3040	20.9269

Frequency (cm⁻¹)	IR Intensity (KM/Mole)
1117.8355	14.6381
1166.4175	46.2221
1185.1876	11.1807
1219.9312	11.9043
1269.0637	6.5137
1311.6700	78.8161
1336.9910	7.2283
1371.9082	22.4304
1373.6296	23.1000
1387.1234	7.1248
1392.9555	11.4361
1411.2348	126.9167
1413.3351	94.4765
1449.9949	210.5004
1466.2984	32.8507
1477.8414	4.8955
1481.3267	165.3082
1481.9819	55.4498
1486.7747	96.1718

Frequency (cm^{-1})	IR Intensity (KM/Mole)
1491.0137	87.5881
1497.7289	408.7763
1504.2825	28.2227
1631.8229	978.0786
2995.8721	31.4893
2998.6913	14.8338
3005.1363	48.6560
3009.1221	122.6063
3014.5286	58.1134
3038.6258	62.3625
3046.0764	10.2486
3062.2564	7.7137
3067.9582	162.9333
3073.9649	79.4591
3098.2997	36.3421
3110.4794	16.7033
3138.4482	22.2614
3568.6548	101.8446
3821.9016	28.6586

Delanzomib calculations at level of theory - B3LYP/6-311+G(d,p)

Delanzomib in gas phase

Frequency (cm^{-1})	IR Intensity (KM/Mole)
10.4236	0.5151
11.4067	0.4179
23.0316	0.5175
36.0597	0.0389
37.9727	0.7994
44.9828	0.6829
47.3039	0.6317
52.7665	1.0012
61.6511	1.8860
66.5208	1.6941
94.5003	8.4529
100.5606	6.0362
106.7457	0.5541
123.2882	1.5404
128.4515	10.1819
152.6834	0.5977
167.5719	2.3574

Frequency (cm⁻¹)	IR Intensity (KM/Mole)
169.7148	7.4671
176.3391	3.7875
215.4854	4.0562
220.0368	9.2504
225.8557	1.5867
238.1552	4.3639
241.6126	26.3852
247.6120	11.1094
261.1921	6.1519
271.9365	77.4612
300.3194	2.0634
304.6231	10.2856
316.0252	24.1348
329.5915	10.3814
346.8249	2.1121
357.3353	10.2895
375.1918	10.6249
388.6830	6.1573
405.2418	9.8424

Frequency (cm^{-1})	IR Intensity (KM/Mole)
407.6315	2.2404
413.4092	0.4743
428.8286	16.1310
441.9150	1.7833
452.5760	3.8278
475.3920	27.0956
480.3356	5.8163
511.7884	245.6339
538.8023	3.8441
549.5670	18.5289
588.4307	15.3486
594.6039	47.7119
628.1395	34.0240
631.5744	1.9299
640.7782	8.7103
664.9177	41.4171
683.6719	8.4210
700.5062	49.9117
706.8092	38.8879

Frequency (cm⁻¹)	IR Intensity (KM/Mole)
730.1116	0.9869
740.8785	17.6359
751.5177	20.3736
769.5974	56.4978
771.8587	41.9972
796.1822	6.5311
823.0219	1.6868
828.4892	5.5848
853.2329	8.9644
857.0282	1.6490
862.1894	4.3256
901.8453	14.6373
914.6589	12.6933
929.9321	1.5657
936.0996	4.3534
939.5037	5.5031
947.3346	48.5053
947.8216	14.8576
956.5594	74.2566

Frequency (cm⁻¹)	IR Intensity (KM/Mole)
964.9870	0.6088
980.8672	19.7875
989.9436	0.9208
1005.6231	106.1186
1008.6027	0.1464
1012.6165	7.5147
1016.6176	1.5480
1020.4516	0.6050
1027.8802	24.8403
1046.6294	5.3859
1057.5381	12.1170
1065.5802	18.1477
1076.9796	4.0872
1095.6048	2.3481
1103.4964	15.7576
1106.3791	9.5469
1107.6939	7.5901
1116.5136	4.8633
1148.4528	39.5485

Frequency (cm⁻¹)	IR Intensity (KM/Mole)
1153.9676	1.5840
1182.2237	0.8149
1184.2661	0.1214
1190.4919	7.9296
1200.6774	26.7670
1205.5995	14.2938
1226.6352	37.0364
1252.3776	5.7402
1265.7505	60.2948
1271.0085	25.7915
1284.5150	50.2833
1301.0451	10.1342
1304.8583	19.7667
1309.1358	30.2895
1327.1563	0.2675
1332.3031	38.6788
1349.7594	78.5507
1354.5410	16.7442
1355.1868	16.3403

Frequency (cm^{-1})	IR Intensity (KM/Mole)
1361.2001	60.9626
1373.1555	101.4728
1389.1923	34.1523
1399.0482	51.8498
1401.5967	6.3917
1403.1925	15.0844
1405.9838	99.5891
1420.7154	3.0950
1430.7182	14.1534
1435.3688	0.2442
1476.5554	96.6352
1483.2114	6.9155
1483.9317	0.6175
1484.1612	4.2579
1492.1024	2.2006
1497.5855	2.8505
1500.2077	5.8684
1504.7247	2.3862
1513.3441	18.2109

Frequency (cm^{-1})	IR Intensity (KM/Mole)
1528.0385	5.5692
1562.2687	274.5605
1582.2824	242.5794
1603.8209	24.3976
1619.4292	69.8124
1625.8757	42.7738
1642.1661	0.4721
1691.5044	206.0377
1711.8624	330.2952
2982.0883	16.4242
3001.8075	16.2557
3010.1356	23.4640
3014.9694	21.9592
3018.4040	47.0389
3040.9208	9.4773
3044.2659	11.6548
3054.0041	25.0327
3068.3194	4.6723
3075.5720	78.0060

Frequency (cm^{-1})	IR Intensity (KM/Mole)
3082.0252	49.7671
3094.9846	32.1957
3103.8966	3.6041
3111.8506	20.0315
3121.1876	18.1144
3164.0052	1.8591
3171.5601	3.6838
3177.3673	14.1471
3181.1382	11.1282
3190.1482	17.1899
3200.0431	5.2207
3202.2577	5.7353
3218.0069	1.9699
3483.0939	227.3591
3564.5323	119.5447
3831.4895	26.6818
3862.5198	21.6390
3867.8160	60.6428

Delanzomib in diethyl ether

Frequency (cm^{-1})	IR Intensity (KM/Mole)
11.7433	0.0549
16.8312	0.2777
21.9650	0.8878
36.9215	0.3595
42.6184	0.1558
46.2196	0.1292
51.3796	1.5464
56.8708	0.5009
63.4245	1.9071
73.0531	2.8982
93.1783	6.7433
98.9026	1.9551
104.3405	4.5271
116.9602	1.3735
127.8858	8.7528
138.1071	1.6952
158.7704	5.7732
171.4069	8.7962

Frequency (cm^{-1})	IR Intensity (KM/Mole)
173.1068	1.3762
219.7912	5.0570
223.3028	19.0406
226.2840	2.3230
237.5945	4.9817
244.1353	39.0607
248.1238	1.3123
262.6059	3.5070
272.7578	97.1829
296.3938	3.9999
301.7844	2.9942
316.7352	25.7265
327.2992	25.4277
355.8513	6.0446
376.4856	12.2249
389.7882	6.9604
401.7912	8.0865
405.0062	10.1362
410.6617	1.1103

Frequency (cm^{-1})	IR Intensity (KM/Mole)
413.9317	0.7992
435.0019	15.1617
441.0808	1.9973
456.1528	9.9640
475.8093	27.5773
481.2281	23.7846
528.8411	294.5448
538.4741	14.8250
554.4146	18.2730
588.8995	8.2846
594.1756	59.2531
630.3402	2.0012
639.6790	10.8392
644.9705	40.3343
661.9620	82.6389
673.0524	48.9375
691.1174	37.0912
706.1587	56.3088
737.2729	9.2356

Frequency (cm^{-1})	IR Intensity (KM/Mole)
739.4056	15.8389
748.4898	20.7544
767.2857	45.9804
769.9778	74.3052
796.2650	7.3433
822.7394	2.8690
824.0135	4.6489
853.3992	12.4918
857.6924	2.1290
860.7384	3.7080
902.8461	15.9643
912.0918	25.0586
929.0248	2.0204
935.1002	10.8163
940.0851	3.1899
946.4954	10.8111
948.8049	2.0987
964.1670	113.5927
964.8068	74.7808

Frequency (cm^{-1})	IR Intensity (KM/Mole)
982.3528	7.0610
993.9105	1.0099
1011.3772	3.8086
1011.6854	4.1582
1015.3015	4.3282
1021.8572	118.9110
1023.7048	0.6263
1025.8425	37.9441
1044.7467	7.8324
1058.9664	34.8349
1066.6114	16.6239
1078.5614	6.5267
1094.6668	2.9612
1102.7754	19.8839
1104.5566	11.3834
1106.5165	18.0036
1114.8735	4.3623
1144.8241	49.4875
1156.1579	0.5948

Frequency (cm⁻¹)	IR Intensity (KM/Mole)
1179.2572	0.6138
1183.3592	0.8093
1189.2977	9.9784
1200.0697	16.7804
1205.1941	31.2295
1228.4236	46.4657
1254.2471	13.6465
1266.2464	75.6202
1274.4543	98.0766
1281.3226	43.2786
1300.4751	9.5154
1303.1040	30.7389
1312.5622	40.3567
1325.3056	4.0869
1332.5009	48.5674
1345.6937	109.4391
1353.9707	27.4775
1355.0057	10.8905
1361.2865	140.4280

Frequency (cm^{-1})	IR Intensity (KM/Mole)
1372.4019	90.3281
1382.9978	111.6301
1397.7262	5.4587
1399.1838	9.1341
1402.7661	9.0458
1405.1709	169.1171
1416.6947	3.5997
1426.7015	14.7531
1432.8726	1.3959
1476.1419	120.7565
1479.8623	7.8127
1480.8196	0.8561
1481.5839	11.3982
1486.1919	2.7605
1493.5889	4.4669
1494.2727	8.1627
1497.4255	3.1212
1505.6758	22.3564
1526.8365	10.3751

Frequency (cm^{-1})	IR Intensity (KM/Mole)
1558.3430	383.9843
1578.1785	360.7945
1601.2345	33.7784
1617.5158	93.9609
1623.7354	80.5288
1639.1968	0.6149
1675.3422	307.0503
1692.2934	563.8600
2986.8468	10.4667
2999.0772	45.0730
3010.5696	35.7809
3015.3808	30.5968
3018.7089	51.1194
3040.2973	17.3006
3047.5273	49.5190
3048.0680	12.3472
3069.5304	10.0532
3075.2528	100.5726
3081.4336	67.2558

Frequency (cm^{-1})	IR Intensity (KM/Mole)
3093.7034	47.2975
3104.3060	5.8244
3110.4336	32.2572
3119.0811	25.9730
3166.1833	1.5208
3173.8081	6.1542
3182.4985	34.5337
3184.4333	1.3615
3191.4894	22.3746
3199.5687	7.6164
3206.7142	7.2855
3220.2085	1.6949
3471.0950	316.3413
3554.7012	163.4143
3818.4097	47.4593
3838.7902	29.5984
3844.4675	112.3698

Delanzomib in methanol

Frequency (cm⁻¹)	IR Intensity (KM/Mole)
11.4648	0.0883
17.9344	0.2757
22.8611	1.0665
36.2807	0.2485
41.4287	0.5693
46.5344	0.0896
51.1486	2.6063
55.7147	0.4560
62.4648	1.5351
72.4297	4.0453
87.3984	12.1890
93.3017	2.0331
98.8658	1.0888
110.4879	3.9714
126.9354	8.7213
135.6438	2.0580
156.9109	7.5173
170.9456	6.9581

Frequency (cm^{-1})	IR Intensity (KM/Mole)
176.3329	3.7894
222.3588	5.3284
225.3115	10.1593
227.0097	6.4734
237.4010	5.9655
247.6275	30.2756
249.3301	6.8674
263.1738	6.7156
276.3803	127.1149
294.8534	2.4490
301.4705	3.6413
317.0812	27.7972
324.9006	37.2029
357.3900	5.9222
376.8799	12.6224
390.1647	8.9099
405.0935	11.7217
407.6116	5.4464
414.0898	0.8608

Frequency (cm^{-1})	IR Intensity (KM/Mole)
435.1159	21.8259
440.2921	3.2116
445.8147	9.3392
459.2821	7.3360
474.3054	47.4945
479.4630	23.2750
536.3998	249.4698
539.0195	106.3072
550.7539	22.7774
588.7183	6.7267
593.3770	71.1837
629.9833	2.5696
639.2929	13.6194
648.7549	32.0407
657.4767	151.4442
665.4794	61.5960
689.1407	29.0982
706.2067	66.5999
736.7054	22.4942

Frequency (cm^{-1})	IR Intensity (KM/Mole)
740.3352	6.2269
747.7760	20.7174
765.4811	46.5409
768.2744	91.6674
796.2310	8.1306
822.0991	3.0018
823.0017	5.0488
853.1918	15.4323
858.5617	1.9718
860.5060	3.3747
903.9168	18.2811
910.9813	30.9935
928.3547	2.1855
933.9008	13.1542
940.6864	2.4511
945.8125	6.3159
949.3604	2.3762
964.0929	1.5250
969.6875	228.9442

Frequency (cm⁻¹)	IR Intensity (KM/Mole)
982.7715	3.3875
996.4831	0.9965
1010.9296	9.4981
1013.4400	0.1380
1014.7294	5.8801
1021.8651	130.0575
1024.7923	4.5244
1025.1221	41.0187
1043.9517	9.5581
1057.9530	33.4299
1066.6359	22.0482
1078.2523	6.3749
1094.0552	3.5383
1102.1072	24.8520
1103.8856	9.2250
1105.9736	23.6108
1114.2453	6.5858
1142.8254	54.2473
1156.5785	0.9719

Frequency (cm^{-1})	IR Intensity (KM/Mole)
1177.4291	0.6535
1182.9272	1.6394
1188.9460	11.2214
1199.5595	11.9885
1204.5414	38.9889
1226.7731	47.5401
1255.8498	22.4981
1266.8456	83.9867
1274.3233	119.8239
1281.1278	54.1682
1300.9174	8.1863
1303.1165	36.9787
1314.4992	51.7993
1324.1608	6.9463
1332.7849	51.8070
1343.7620	163.5804
1353.9059	31.3175
1354.8648	15.7205
1360.1121	180.2812

Frequency (cm^{-1})	IR Intensity (KM/Mole)
1372.1875	83.1199
1378.9017	135.7688
1395.9456	4.9282
1397.3711	9.3249
1402.1423	27.3623
1404.2885	160.9939
1414.9272	5.9315
1424.3798	14.2408
1431.5388	3.6047
1475.7714	125.1241
1477.1612	12.2953
1478.8052	0.6082
1480.7234	17.7676
1483.8678	3.7218
1490.6357	9.7769
1491.8161	5.1338
1493.5030	3.0804
1502.3882	25.6391
1526.0840	12.9629

Frequency (cm⁻¹)	IR Intensity (KM/Mole)
1556.9544	460.4108
1577.0747	460.0793
1599.8801	37.4140
1616.3640	105.8437
1622.4762	104.9486
1637.6718	0.8433
1663.4788	464.6585
1680.8639	649.7091
2988.8766	14.5464
2999.9539	53.9981
3010.6423	44.6373
3015.9491	49.7573
3020.1529	37.4437
3038.4266	20.9554
3044.5141	74.6797
3049.6613	14.8926
3070.0049	14.6933
3074.9269	120.2578
3081.4262	73.1882

Frequency (cm⁻¹)	IR Intensity (KM/Mole)
3091.2555	58.0321
3103.4487	6.8135
3108.6316	44.9397
3117.6405	30.9474
3167.1907	1.3971
3174.8045	7.8577
3183.8377	37.5581
3187.1690	4.1796
3192.2699	24.3896
3199.5191	9.1966
3208.5728	7.9279
3220.7841	1.8530
3463.0619	383.9407
3551.5440	189.0145
3811.8108	60.4912
3831.3784	82.9192
3834.4461	90.2605

Delanzomib in water

Frequency (cm^{-1})	IR Intensity (KM/Mole)
11.2982	0.0941
17.4583	0.3495
22.6915	1.0841
36.3101	0.2719
41.1571	0.5896
46.3248	0.1271
50.8398	2.8271
55.3289	0.4526
62.1237	1.5649
72.1966	4.2766
86.2153	11.6403
93.4578	2.6071
98.8574	1.1226
110.1784	4.0879
126.9434	8.8911
135.4757	2.1714
156.7127	7.8537
170.8975	6.9188

Frequency (cm⁻¹)	IR Intensity (KM/Mole)
176.1761	3.9071
222.3744	5.6036
226.0350	6.8086
227.0904	8.2750
237.3299	6.0919
248.2428	18.1828
249.5744	15.1109
263.1909	7.0213
277.8200	134.0061
294.8672	2.4572
301.5301	4.1929
317.1713	28.1184
324.8156	40.2328
357.4599	5.9848
376.8918	12.7603
390.1326	9.1770
404.8561	11.9547
407.5691	5.3978
414.0250	0.8742

Frequency (cm^{-1})	IR Intensity (KM/Mole)
435.6351	21.6782
440.3149	2.9042
449.0141	12.9098
460.6496	6.3845
474.2270	47.2918
479.5882	24.9822
536.3744	257.2926
538.9931	105.2068
550.8798	23.2095
588.6683	7.1431
593.4529	72.1866
629.9467	2.6416
639.2498	13.8784
648.8413	29.9060
656.7104	160.7365
665.6268	63.2150
689.1150	30.0146
706.1471	68.4106
736.6816	23.5242

Frequency (cm^{-1})	IR Intensity (KM/Mole)
740.3578	5.7543
747.6926	20.8354
765.4461	48.9785
768.0823	92.1649
796.2396	8.2509
822.0290	3.0933
822.9109	5.2732
853.1661	15.8017
858.6141	1.9609
860.4473	3.3871
903.8280	18.6577
910.8425	31.5254
928.1579	2.2133
933.6786	13.3906
940.7027	2.4394
945.7763	6.2670
949.4293	2.4257
964.0566	1.3095
970.3113	232.5914

Frequency (cm^{-1})	IR Intensity (KM/Mole)
982.7337	3.1905
996.7110	1.0005
1010.8521	9.6290
1013.6269	0.1921
1014.6725	6.0451
1022.6151	131.7957
1024.8841	14.1849
1025.1030	31.9097
1043.8728	9.6619
1057.8561	34.9335
1066.7355	22.3879
1078.3260	6.7207
1093.9934	3.6214
1102.0089	25.6383
1103.7843	8.8102
1105.9078	24.1360
1114.0283	6.8989
1142.5034	55.0012
1156.5593	0.9165

Frequency (cm⁻¹)	IR Intensity (KM/Mole)
1177.2259	0.6611
1182.8645	1.7214
1188.8885	11.4199
1199.4689	11.8801
1204.4789	39.6406
1226.5557	47.6248
1255.9997	23.7306
1266.9356	83.9979
1274.4296	120.8965
1281.2172	56.8470
1300.9685	8.4293
1303.1542	37.5174
1314.5893	54.1613
1324.0281	7.2434
1332.7973	52.0326
1343.4626	173.0727
1353.8903	32.0021
1354.8616	16.1177
1359.9324	187.9611

Frequency (cm^{-1})	IR Intensity (KM/Mole)
1372.0784	81.7060
1378.4938	134.3417
1395.6975	4.5902
1397.0791	8.9225
1401.9373	28.2068
1404.1041	162.0435
1414.6483	6.1627
1424.1213	14.2150
1431.3951	4.0164
1475.7175	124.2563
1476.8544	14.1167
1478.5080	0.6349
1480.6348	18.5700
1483.5286	3.7564
1490.2341	9.7848
1491.3892	5.3040
1493.0281	3.1045
1501.9170	26.0372
1525.9930	13.4339

Frequency (cm^{-1})	IR Intensity (KM/Mole)
1556.7022	470.7719
1577.2437	473.2644
1599.7353	37.6515
1616.2426	107.0524
1622.3288	107.8017
1637.5120	0.8800
1661.9647	491.7282
1679.5685	652.9154
2989.0325	14.1462
2999.8339	57.0777
3010.6416	45.7834
3015.9601	50.7422
3020.2356	37.4001
3038.1235	21.4669
3044.3705	77.4479
3049.7285	15.3506
3070.0453	15.4209
3074.9028	122.7104
3081.4368	73.7986

Frequency (cm^{-1})	IR Intensity (KM/Mole)
3090.9870	59.4645
3103.2948	7.0215
3108.3338	46.7261
3117.5853	31.4842
3167.2957	1.3934
3174.9020	8.0188
3183.9350	37.7661
3187.4712	4.6244
3192.3427	24.5997
3199.4896	9.3786
3208.7595	7.9730
3220.7886	1.9085
3461.5421	394.0829
3551.0584	191.9802
3811.1262	61.9285
3830.2113	80.5299
3833.4230	96.2218

Delanzomib deprotonated (de1) in gas phase

Frequency (cm^{-1})	IR Intensity (KM/Mole)
16.8237	1.0249
20.6775	0.1736
25.0396	1.3276
42.4180	3.2795
48.9932	2.4182
52.7480	0.1640
54.2480	1.0762
62.1847	0.7131
68.6142	1.2533
69.5037	0.4451
82.8131	2.9746
92.8129	13.1209
100.5986	3.8741
111.0373	6.5670
127.4896	8.2699
144.3323	7.7270
164.6546	0.2012
176.6339	5.7482

Frequency (cm⁻¹)	IR Intensity (KM/Mole)
182.5374	1.8481
219.1714	4.8040
226.2150	1.1438
230.9437	1.2119
237.6260	3.5679
250.1775	3.2153
254.7487	6.1360
266.8036	4.0642
287.1403	1.1865
305.9131	0.0682
320.5708	14.6392
329.7019	5.8266
369.2025	5.6566
382.7754	3.5782
386.2426	11.4527
397.9884	3.6283
416.1589	0.7811
420.3873	7.2417
436.0288	2.5220

Frequency (cm⁻¹)	IR Intensity (KM/Mole)
441.3523	1.0191
459.0702	4.6126
477.8781	15.2640
480.3348	31.3052
525.3711	16.4707
536.5712	33.4849
545.3208	113.8290
587.6729	23.2934
599.2801	14.6662
628.0032	1.2654
640.0804	12.6564
648.9263	37.2318
671.4075	38.1287
677.8896	3.9400
679.0402	92.3588
694.8365	12.0257
708.4279	14.2274
721.7051	11.6043
742.7161	16.8845

Frequency (cm⁻¹)	IR Intensity (KM/Mole)
750.8516	23.9572
768.2693	79.0398
789.3994	15.8190
795.7722	7.5916
811.3082	5.4155
825.9904	0.8392
848.6568	8.1700
876.4514	3.3649
879.5668	6.7874
894.2363	86.7911
908.4705	24.2550
929.3916	1.4931
932.1735	0.9543
935.6471	20.0597
943.8239	20.5044
950.0482	83.8147
953.6582	1.3570
962.2152	0.4895
986.6190	2.2424

Frequency (cm^{-1})	IR Intensity (KM/Mole)
991.1525	34.6087
1008.9393	0.2570
1011.5231	6.9290
1015.3444	2.8928
1021.5571	9.2197
1037.5017	14.0197
1042.5192	13.7880
1068.5710	0.4381
1074.6406	6.4541
1087.4209	21.2350
1092.2929	1.3403
1093.5537	0.5101
1097.2695	22.7305
1098.1815	31.5864
1108.4010	5.6305
1118.0660	1.5177
1140.0518	29.6417
1149.0668	3.3886
1176.8585	1.4569

Frequency (cm^{-1})	IR Intensity (KM/Mole)
1186.1759	5.4357
1187.7606	9.7766
1202.4378	17.4902
1214.8437	16.8072
1215.9611	1.1953
1253.7517	17.3360
1266.7556	41.8262
1277.0260	12.2261
1299.8674	12.4577
1304.3843	18.8641
1308.1864	5.7139
1316.7566	11.1933
1327.1874	39.7521
1332.8648	29.1221
1347.0948	0.1088
1354.7028	0.7962
1359.1388	1.7022
1381.3287	8.7730
1383.3828	7.1956

Frequency (cm^{-1})	IR Intensity (KM/Mole)
1387.4186	5.2585
1393.1123	5.5641
1406.4475	16.2459
1409.4001	7.9678
1433.1289	4.2712
1467.1231	152.1648
1474.1287	75.5975
1480.7943	18.7766
1481.0259	2.7614
1490.3477	12.9856
1490.7374	5.2953
1496.2766	2.9423
1496.8163	5.1629
1500.6542	3.1773
1511.6323	5.2690
1521.2683	387.3998
1528.6678	41.0982
1562.7125	334.0606
1566.3729	307.0246

Frequency (cm⁻¹)	IR Intensity (KM/Mole)
1600.4649	24.2340
1617.0846	106.8649
1621.9887	71.5941
1636.6469	5.7040
1699.1742	286.2083
1719.6530	292.9671
2961.6153	35.1156
2981.2939	32.2544
2994.4511	51.9837
3001.5366	76.1563
3011.8197	13.4757
3027.8025	12.3028
3037.0057	25.2293
3047.4786	2.1399
3060.2969	110.3731
3061.6169	16.2137
3065.0727	53.8086
3066.2235	15.7799
3102.7462	22.0237

Frequency (cm⁻¹)	IR Intensity (KM/Mole)
3102.8628	27.0593
3114.9990	87.0466
3117.1411	21.1636
3147.9678	4.7999
3163.2149	36.6945
3165.4314	4.5369
3174.9526	39.6730
3196.8375	14.7504
3210.4847	5.2344
3214.0372	2.2510
3521.0062	122.5494
3556.0563	76.2098
3567.2556	527.7401
3833.8728	8.7995

Delanzomib deprotonated (de1) in diethyl ether

Frequency (cm⁻¹)	IR Intensity (KM/Mole)
19.5516	0.3033
31.7047	3.0458

Frequency (cm⁻¹)	IR Intensity (KM/Mole)
39.1511	7.5006
46.1379	0.9648
55.9365	0.7227
58.3787	2.3281
63.4236	2.1782
69.6613	0.7887
78.3957	1.0331
90.4052	2.7866
92.5175	1.9705
102.7283	4.2751
111.3425	20.7075
120.2945	2.6896
136.2629	1.7713
163.9520	3.8193
175.3616	6.9188
182.1969	8.0946
188.4597	2.2962
220.5303	7.5781
227.7408	5.8385

Frequency (cm^{-1})	IR Intensity (KM/Mole)
246.2755	1.0675
250.8918	8.8889
261.1920	4.3549
268.3713	6.5838
281.6017	5.7681
293.1199	0.2083
306.1414	0.2858
323.5226	20.3501
334.0697	9.0431
369.7569	11.9464
386.9414	19.3177
388.6731	3.5224
399.8344	8.5668
417.5064	0.8885
423.3024	10.3613
438.6528	1.5775
447.3382	4.7690
461.6874	9.1848
474.2698	59.1276

Frequency (cm^{-1})	IR Intensity (KM/Mole)
477.9807	15.8489
521.0064	155.8424
527.3392	45.4268
538.5802	8.3031
586.7165	33.4395
598.8381	29.1883
628.2368	51.1554
630.0181	28.1465
639.6895	14.6358
674.5164	17.5184
683.0398	33.4463
691.1133	66.8368
699.6708	60.9594
707.1444	32.3754
712.1685	34.1886
742.4015	28.0657
751.5909	22.4546
767.2290	114.2073
779.1615	11.8419

Frequency (cm^{-1})	IR Intensity (KM/Mole)
795.5889	12.4197
809.3548	10.4861
832.2806	1.3099
850.3323	11.4555
866.4741	1.8568
878.3361	10.9649
892.3168	137.6349
909.3937	31.9092
935.0627	32.3970
937.5158	1.1802
941.6982	12.3780
945.9481	6.2592
949.4385	120.6745
955.6020	1.1419
966.5252	0.3069
991.5724	40.4038
996.1741	1.0386
1011.4186	10.9981
1015.4447	3.3000

Frequency (cm^{-1})	IR Intensity (KM/Mole)
1019.2444	0.6091
1022.5116	7.1247
1031.9153	0.5622
1038.1548	5.5797
1042.4383	29.6608
1073.1178	8.2795
1085.3257	30.8956
1092.9203	0.9014
1093.5983	21.7038
1100.6498	33.0993
1101.0302	37.2297
1106.3936	13.9150
1116.9225	1.9977
1137.9970	44.4539
1153.7950	9.1079
1178.9797	2.0469
1185.0552	4.8363
1193.9319	10.0853
1202.3075	22.7471

Frequency (cm⁻¹)	IR Intensity (KM/Mole)
1207.6574	7.0597
1216.9287	6.9315
1256.2111	21.9007
1268.3437	75.9777
1279.0870	14.1934
1302.0946	20.7552
1311.1775	20.2325
1314.6518	3.5711
1319.7397	29.3308
1327.1187	11.6040
1328.5888	74.1700
1347.8833	0.3712
1354.9188	0.9491
1366.7990	7.7449
1379.8182	32.0471
1383.1187	3.8516
1392.3224	4.7017
1397.8904	7.3542
1407.8901	19.2679

Frequency (cm⁻¹)	IR Intensity (KM/Mole)
1420.4057	10.9696
1432.6977	5.4317
1469.6399	215.1665
1474.9128	114.5298
1481.4297	9.3574
1482.0587	5.2331
1486.6805	4.9548
1489.6092	37.5135
1492.2322	2.1274
1497.7999	6.6043
1503.7380	5.3159
1509.4531	30.8474
1518.3661	793.8769
1528.1076	55.7792
1542.1942	230.1710
1558.8014	529.5913
1600.1909	44.9133
1616.9040	139.2498
1623.2062	101.8554

Frequency (cm^{-1})	IR Intensity (KM/Mole)
1637.5663	2.6446
1681.0488	400.4300
1690.9824	472.1382
2971.9121	48.6503
2984.7590	48.0492
3001.7883	53.8548
3007.9703	54.7594
3015.7029	11.9971
3027.5951	33.5411
3037.6039	23.7061
3058.1606	6.8585
3064.0086	17.5821
3066.3266	70.4844
3067.3938	78.2943
3070.5825	88.2182
3099.6483	45.3144
3104.2534	32.0727
3116.9086	29.2888
3159.8438	9.7882

Frequency (cm^{-1})	IR Intensity (KM/Mole)
3166.8773	8.8895
3176.4489	21.3005
3180.3913	8.2177
3186.0795	32.6851
3204.3536	11.1860
3208.1966	0.4516
3217.4895	2.1553
3507.9776	901.5831
3515.7616	119.9047
3572.7184	104.5823
3829.2473	22.3303

Delanzomib deprotonated (de1) in methanol

Frequency (cm^{-1})	IR Intensity (KM/Mole)
11.4029	3.8616
17.9026	1.6794
32.3139	1.4139
41.2688	2.4291
44.6167	0.1968

Frequency (cm^{-1})	IR Intensity (KM/Mole)
47.0340	6.1728
53.7584	1.7549
57.8405	3.8256
64.2483	0.0203
66.0846	4.8921
78.4310	6.9932
91.3148	15.9023
97.9975	5.2449
112.6872	7.6650
125.1525	4.6810
152.4923	6.6870
166.7609	2.8316
176.2969	8.8922
184.4004	3.7541
216.6089	8.4967
227.0112	1.9949
238.7546	5.7222
245.7427	1.0297
251.1937	4.1483

Frequency (cm^{-1})	IR Intensity (KM/Mole)
257.5841	11.2835
271.4780	6.2139
291.4732	1.7304
306.9436	1.0187
322.4789	22.4141
330.4046	8.7370
368.3720	15.0380
384.1825	11.8629
387.9646	18.1472
396.4569	9.3954
414.4268	0.8732
420.0530	11.5485
436.4411	9.9392
439.7931	4.3851
461.8404	16.9271
476.0017	12.8906
483.6232	74.9159
507.8575	187.1724
527.7019	39.9006

Frequency (cm^{-1})	IR Intensity (KM/Mole)
539.1881	12.2776
583.7651	56.9552
597.5602	39.1400
611.6158	81.4915
630.0517	3.6339
639.3752	14.2215
672.2896	32.6455
673.0618	43.4455
676.3077	156.7225
688.6128	27.5331
697.8791	21.6456
706.8387	58.9219
742.5718	38.8206
751.5041	20.4177
766.6448	120.9721
770.6406	5.3733
794.0766	13.9265
807.7877	13.3583
826.7122	1.0413

Frequency (cm^{-1})	IR Intensity (KM/Mole)
852.2421	15.5471
861.5812	0.8883
876.2631	4.7683
892.5856	122.3020
911.1273	48.9946
929.3769	2.7892
930.4625	41.9736
940.5726	0.4868
950.1690	1.3172
953.4891	70.1297
954.6253	137.1005
961.0457	1.5676
993.1952	49.6213
998.6857	1.1675
1010.7468	12.9263
1014.3425	4.8295
1016.1470	0.5427
1020.4543	7.5812
1024.4535	0.4966

Frequency (cm^{-1})	IR Intensity (KM/Mole)
1039.4042	9.0828
1045.7228	32.8098
1074.2006	9.6956
1083.1850	34.6281
1090.0736	6.9880
1092.3917	2.2072
1102.9537	4.1914
1105.4466	39.6471
1107.0319	89.1804
1115.6640	1.7815
1132.9998	59.5273
1148.4699	12.4053
1179.4033	1.8056
1183.2102	2.1490
1185.2500	10.6548
1198.9673	23.9866
1205.1731	13.3453
1214.1294	15.4578
1257.2899	31.7540

Frequency (cm⁻¹)	IR Intensity (KM/Mole)
1266.7968	89.2642
1277.8430	24.3843
1301.0346	23.0213
1301.8861	15.5324
1310.6885	6.7817
1320.2146	35.3946
1324.9241	2.8713
1328.1537	84.5021
1349.3855	0.7630
1352.7201	12.2567
1356.1818	1.5076
1372.9283	7.2821
1378.9760	11.6362
1384.0375	30.7367
1393.8156	9.1987
1405.1902	28.2827
1410.3299	10.0983
1431.8922	6.8009
1467.5334	260.3905

Frequency (cm^{-1})	IR Intensity (KM/Mole)
1474.0323	124.3576
1478.7504	12.9067
1481.4066	5.3497
1482.2843	2.2118
1485.5389	130.9348
1488.1943	18.8301
1489.8913	7.5901
1493.3405	9.8350
1499.3119	600.7913
1501.6840	395.3170
1526.5296	69.8553
1531.2860	265.5989
1554.5422	555.8695
1599.9999	50.7835
1616.0820	166.0200
1622.1356	118.8704
1637.9220	1.2378
1670.1069	428.5027
1675.7280	589.4424

Frequency (cm^{-1})	IR Intensity (KM/Mole)
2980.2528	60.4003
2990.3754	56.0575
3004.1249	60.1387
3009.2349	53.1357
3017.1842	10.0930
3029.2772	43.5182
3038.6771	26.0857
3060.6875	15.3675
3061.6152	11.6513
3061.8465	50.1204
3069.0013	155.8818
3072.9519	85.6705
3088.5656	64.3364
3104.5508	38.1574
3120.2026	30.2530
3167.6950	1.0799
3176.6318	8.1931
3184.4367	34.6651
3187.0770	2.9613

Frequency (cm⁻¹)	IR Intensity (KM/Mole)
3191.6841	23.5353
3199.9725	7.4471
3208.8638	8.2880
3221.8093	1.6709
3483.8952	1063.5391
3523.0375	135.2055
3574.3419	134.0423
3822.5084	27.0430

Delanzomib deprotonated (de1) in water

Frequency (cm^{-1})	IR Intensity (KM/Mole)
10.9957	0.0983
18.6248	5.6563
28.3429	2.2076
38.8585	1.6802
40.6267	10.2685
47.5282	1.1895
54.2253	0.8332
56.7234	5.6905

Frequency (cm^{-1})	IR Intensity (KM/Mole)
59.8093	1.7927
68.2624	1.0237
78.3237	5.2062
93.7707	14.4470
97.8354	4.4032
111.4644	9.0444
124.6757	5.1036
153.8131	7.8691
165.4673	2.4831
175.8099	8.8945
183.3752	3.4745
215.9238	9.1434
226.1398	1.7845
235.5050	3.1407
243.3442	3.2701
249.0889	4.5668
257.2911	12.3486
270.7338	5.1243
292.0946	1.9544

Frequency (cm^{-1})	IR Intensity (KM/Mole)
306.9425	1.3207
321.7963	22.5744
330.5114	8.5063
368.0798	15.3587
383.7945	15.9830
387.2754	15.2194
397.1138	10.1972
414.2165	0.8561
419.3926	12.1804
436.9822	9.6972
439.5257	4.8444
460.7282	20.0380
476.8109	12.5101
485.1501	76.3250
504.4292	193.4470
526.6833	38.9795
538.3076	12.2351
583.9737	62.5152
596.1372	39.6167

Frequency (cm^{-1})	IR Intensity (KM/Mole)
609.0792	86.9080
629.7669	3.7019
639.2789	14.7826
666.5934	190.4407
670.7642	17.5431
672.6835	43.3741
684.5391	15.2597
696.6249	17.4345
706.7779	63.2158
742.4334	39.0805
751.4224	18.5591
766.2647	118.7190
769.1627	9.3385
793.8566	13.1585
807.4852	15.0049
826.6839	0.9173
852.5075	16.1203
860.9365	0.7007
876.2749	4.0484

Frequency (cm^{-1})	IR Intensity (KM/Mole)
893.5240	119.7625
910.8208	47.4652
928.5078	2.9564
929.9003	43.9868
940.9415	0.2963
950.4085	0.5787
953.5097	28.7517
955.6075	183.8067
961.0661	4.7292
993.7226	52.3817
998.6371	1.3813
1010.6497	12.7223
1014.0150	5.2006
1015.0099	0.6965
1020.8112	7.8058
1025.1866	0.5193
1039.9494	6.3949
1044.7792	37.0989
1073.9796	10.4733

Frequency (cm^{-1})	IR Intensity (KM/Mole)
1082.3625	35.2581
1090.0413	4.5795
1091.8753	2.7961
1102.4878	1.4711
1105.0653	37.4020
1108.4531	101.4645
1115.9795	1.7811
1132.0379	59.0965
1148.1496	14.0550
1178.0639	0.8916
1182.2052	2.7722
1186.2573	11.0156
1197.0803	23.5205
1203.3614	16.2099
1213.9011	15.5551
1257.1525	32.8523
1264.9032	84.0762
1277.6730	27.0085
1300.7595	23.8968

Frequency (cm⁻¹)	IR Intensity (KM/Mole)
1303.0354	19.0045
1310.6771	6.2361
1319.9695	29.6405
1324.4022	4.3618
1328.1816	82.3373
1349.8142	0.4479
1350.9948	11.5489
1355.3045	1.7454
1373.2291	7.9844
1378.6008	6.5270
1383.3572	34.3423
1393.4713	9.3374
1404.9108	29.6118
1409.9634	10.6444
1431.5492	7.7645
1466.0247	264.5521
1473.6926	126.9707
1478.5474	17.3645
1480.6260	4.9254

Frequency (cm^{-1})	IR Intensity (KM/Mole)
1481.8463	2.4967
1484.7829	124.0242
1487.7128	56.1046
1488.9331	6.4234
1492.6930	14.1670
1497.4355	744.2520
1500.6574	217.9124
1525.6853	88.6321
1530.5777	310.0543
1553.0125	533.6949
1599.8246	50.8438
1615.9333	174.4371
1621.8118	125.4592
1637.4956	1.2968
1668.0314	435.9895
1673.4086	597.8580
2980.5089	63.0273
2990.5126	58.0730
3004.5264	59.4391

Frequency (cm^{-1})	IR Intensity (KM/Mole)
3009.0349	50.7245
3017.0740	11.6678
3028.7244	49.4989
3038.7893	26.4864
3061.1800	11.9834
3062.3504	28.8268
3062.7943	37.2265
3069.1469	158.8997
3073.4447	85.9997
3086.9136	66.1042
3104.6211	39.1057
3120.0170	30.6392
3167.8322	0.8030
3176.3813	7.8275
3184.2027	34.3881
3187.5941	5.0641
3191.6010	21.7349
3198.3444	8.2876
3209.3459	8.2438

Frequency (cm⁻¹)	IR Intensity (KM/Mole)
3222.1259	1.6393
3485.9883	1065.7854
3524.6935	135.8011
3574.5228	133.3527
3822.6410	27.4464

Delanzomib deprotonated (de2) in gas phase

Frequency (cm^{-1})	IR Intensity (KM/Mole)
11.5817	0.1736
18.5108	1.6912
29.0645	0.1073
35.8932	2.3810
43.8022	0.9546
50.8923	1.0067
54.3407	0.5087
63.4100	1.4967
68.7535	0.1300
80.6300	2.7530
87.5584	0.1554

Frequency (cm^{-1})	IR Intensity (KM/Mole)
95.9279	4.3499
101.2890	7.5172
118.2812	10.7386
124.9540	4.6039
154.8485	3.4983
166.9470	0.4646
175.8268	8.3996
185.4756	1.3943
218.8596	7.9034
223.7326	0.1638
231.7479	2.8692
240.8367	1.1925
254.0575	7.0707
258.8658	1.6141
275.4575	10.8715
293.9895	1.6445
306.5741	2.6136
319.2964	9.3200
323.7800	4.7936

Frequency (cm⁻¹)	IR Intensity (KM/Mole)
369.4366	6.3835
383.5191	10.8543
385.3762	18.7492
392.6362	9.8992
416.1443	0.4104
417.9599	4.9321
427.6140	5.5599
441.7288	2.9379
458.5929	10.2289
480.8595	5.1179
496.2344	49.1923
528.2211	2.4918
542.2529	46.9078
551.8916	45.6670
588.4430	13.1898
602.8753	9.6635
628.1973	3.4278
640.9782	7.4932
653.5904	21.9824

Frequency (cm^{-1})	IR Intensity (KM/Mole)
668.0532	76.3766
680.0018	8.7994
689.2465	54.5206
705.5441	12.9234
711.3640	34.0716
716.7305	33.3975
742.5993	22.2420
750.7479	15.2585
766.2621	71.4706
793.0936	33.6618
794.6291	10.5589
818.7344	3.6507
827.3781	1.0287
850.1711	9.0331
866.9420	1.1955
878.9792	3.5125
900.8728	12.7343
925.4232	0.6579
928.1740	1.2756

Frequency (cm^{-1})	IR Intensity (KM/Mole)
934.5741	1.5848
934.7567	19.3935
950.4230	1.8540
952.9314	1.4719
960.7313	2.8495
969.5686	145.9336
989.8097	0.6036
1006.9035	61.5469
1009.3216	6.3636
1010.5463	45.5691
1012.7537	2.3015
1032.5100	0.3472
1040.1761	13.9560
1048.8753	11.3736
1055.8467	2.7089
1080.4407	1.7306
1084.3959	23.0129
1089.3304	0.1330
1100.0050	14.7032

Frequency (cm^{-1})	IR Intensity (KM/Mole)
1105.5288	2.5908
1110.9742	11.7580
1119.8256	1.5344
1136.1669	50.8836
1141.4367	19.5068
1149.7303	11.1560
1174.6597	0.8283
1179.3993	7.0897
1186.8117	12.0125
1195.5966	29.5283
1204.3560	7.6018
1215.9034	18.7481
1259.3878	15.9608
1263.6591	18.1558
1277.8293	22.6800
1295.7800	11.7037
1308.5210	2.5433
1316.7509	4.6641
1324.7785	0.5454

Frequency (cm^{-1})	IR Intensity (KM/Mole)
1328.8475	2.5931
1335.4593	100.1633
1349.4718	0.3093
1353.9446	12.7823
1358.3656	1.4377
1380.1068	14.8924
1381.7740	0.3730
1386.8071	6.7456
1393.7779	6.6715
1405.9264	15.9238
1410.8810	10.8633
1433.0466	3.1757
1465.3292	147.6762
1472.1641	92.4701
1476.6938	20.2641
1482.7075	3.8238
1488.8547	49.7881
1489.7297	66.6403
1494.9664	199.9434

Frequency (cm^{-1})	IR Intensity (KM/Mole)
1495.6133	46.5977
1499.0149	331.5080
1501.1489	20.7926
1509.6207	11.6696
1523.9776	10.5979
1527.4495	111.7499
1568.0342	470.8705
1603.3058	20.8416
1617.2711	115.2296
1621.2975	53.6632
1636.7654	2.2262
1691.3861	290.8993
1709.7973	251.6052
2968.0840	31.8454
2984.9117	26.4488
2995.4356	55.8435
3004.0837	69.6106
3010.3436	11.8977
3025.6597	36.5945

Frequency (cm^{-1})	IR Intensity (KM/Mole)
3037.8508	25.8847
3038.0122	27.9339
3048.7452	0.1375
3062.5957	78.5586
3069.5933	65.7793
3070.2610	11.7005
3093.4270	33.7236
3103.2647	25.4652
3119.7768	19.5280
3145.7842	69.8443
3149.0810	49.5106
3161.2425	37.1518
3164.0501	9.5524
3174.4250	26.5639
3192.5499	13.7524
3195.7476	15.8481
3215.1862	2.4552
3382.2005	230.4205
3516.3162	124.6969

Frequency (cm^{-1})	IR Intensity (KM/Mole)
3579.2221	526.5402
3823.0419	4.6959

Delanzomib deprotonated (de2) in diethyl ether

Frequency (cm^{-1})	IR Intensity (KM/Mole)
15.9117	2.3415
30.3400	1.1151
43.3257	0.8154
50.7172	3.7186
57.3213	2.5889
64.1666	2.2475
71.9631	0.1664
80.0659	1.2872
84.3336	2.2796
103.7007	3.2289
105.2024	1.5323
111.8247	6.3533
118.3123	18.9445
130.3263	10.9865

Frequency (cm⁻¹)	IR Intensity (KM/Mole)
145.4426	7.8390
163.6261	3.1338
170.3951	6.5876
180.3298	14.5651
198.3341	3.4519
220.3263	10.6132
229.7918	0.8126
241.3382	7.6716
253.2382	3.5985
257.4759	5.9920
263.2827	6.7706
279.2044	11.6323
291.7121	0.2025
305.1727	2.4535
319.5637	16.7573
322.7852	8.1077
368.7814	16.7210
385.6054	17.6076
389.0383	21.2276

Frequency (cm^{-1})	IR Intensity (KM/Mole)
399.3867	1.2565
420.5102	7.4715
431.1772	0.3471
438.1268	1.6382
441.3592	6.4346
460.7310	17.6045
483.0843	9.1691
499.5668	79.7629
532.8055	5.2829
547.9100	9.6653
590.8327	17.6564
599.9075	16.4351
626.7018	3.3090
638.7584	19.9393
652.1480	6.7931
669.7579	162.8369
673.6389	29.8367
680.5940	41.0531
698.2566	51.4015

Frequency (cm^{-1})	IR Intensity (KM/Mole)
710.8589	21.4389
716.7503	29.7031
726.0673	117.3901
745.9903	15.9618
755.3119	22.8665
767.0861	109.4832
793.7136	44.5505
798.1118	9.4834
813.9146	6.5981
830.8062	0.9693
851.7437	11.6061
879.2253	4.3219
885.4431	1.2037
901.3570	22.7442
925.3147	2.1447
931.4463	33.7242
934.2396	2.5838
938.1604	0.0718
954.1000	12.3852

Frequency (cm^{-1})	IR Intensity (KM/Mole)
961.7193	21.1301
962.1382	2.1908
968.6331	176.1894
1002.1305	4.1449
1009.4388	82.5668
1011.3690	78.4569
1013.2866	3.3610
1019.0715	0.2985
1033.0276	0.5227
1048.2480	9.8787
1052.9835	20.6115
1057.5984	17.1485
1083.0843	0.2643
1084.4269	39.1690
1095.1668	4.1658
1099.3762	14.4158
1101.9953	3.4717
1113.1248	8.8694
1119.2335	3.5021

Frequency (cm^{-1})	IR Intensity (KM/Mole)
1133.7470	58.8001
1140.6739	43.4844
1148.9798	6.1246
1179.5488	2.9372
1188.4160	12.9321
1190.4058	1.6880
1197.0514	32.0639
1215.1884	34.6053
1245.2802	5.0729
1257.0940	4.6441
1261.2430	42.9481
1275.3264	41.9741
1298.9364	20.3270
1309.2324	3.3934
1318.3677	4.9564
1327.0094	2.1977
1331.2286	9.9894
1334.3207	123.9642
1350.3211	3.0720

Frequency (cm⁻¹)	IR Intensity (KM/Mole)
1354.7596	9.2420
1365.9715	4.5443
1377.1910	31.1619
1378.7272	4.3628
1386.4962	9.9431
1394.6150	6.4303
1406.7816	22.3294
1414.1727	13.2846
1434.4051	3.7439
1463.2593	216.2077
1474.5967	122.5947
1481.5746	469.8701
1484.1724	119.6832
1485.8916	59.5097
1487.9854	26.5344
1489.4386	76.7621
1493.3688	13.0013
1495.0932	21.8983
1499.5645	24.6651

Frequency (cm^{-1})	IR Intensity (KM/Mole)
1505.8596	8.0503
1517.0238	388.4425
1537.4837	13.8803
1558.1899	571.3378
1601.8510	40.5611
1617.1430	174.6730
1622.8184	76.0411
1640.6382	0.8645
1674.7227	444.1108
1682.3964	377.8106
2978.5846	42.4897
2988.9232	42.9814
3001.7966	60.8639
3009.1995	60.1540
3016.7169	14.3406
3032.9911	22.5065
3038.9079	31.4798
3040.4005	23.3926
3057.7077	4.0074

Frequency (cm^{-1})	IR Intensity (KM/Mole)
3064.2492	18.2281
3068.3748	137.0037
3070.4385	73.8173
3092.5464	51.5021
3107.4178	30.1430
3119.6524	26.8143
3162.5485	2.3190
3174.2993	25.7079
3179.9207	10.7280
3184.9892	31.0747
3185.8666	12.1249
3203.9478	11.2568
3218.5714	2.0150
3226.2897	14.9338
3429.9039	282.6527
3526.3058	826.3709
3534.0936	141.9968
3833.1031	14.4486

Delanzomib deprotonated (de2) in methanol

Frequency (cm⁻¹)	IR Intensity (KM/Mole)
10.4823	1.9432
17.3710	3.1796
24.4600	2.9251
38.4701	0.0809
41.5873	3.3011
48.1210	3.9270
53.4062	1.2723
57.0685	3.3222
58.2706	2.0967
67.5975	6.2555
79.7522	3.2293
96.7234	9.8142
97.0826	3.2076
110.6855	15.0096
121.9499	10.9676
151.0279	2.7832
166.1391	2.9725
175.4181	13.4805

Frequency (cm^{-1})	IR Intensity (KM/Mole)
188.5585	5.4594
216.6441	10.5929
226.7629	0.3226
234.0800	6.7646
243.4775	1.5655
253.4573	12.9108
257.8736	7.1415
272.4002	14.1692
287.7715	0.5883
304.4785	2.8464
320.2681	15.5965
321.7846	10.6126
366.8023	33.2042
381.6404	19.6632
386.1840	20.9368
392.1515	0.2638
414.5291	0.7689
420.7663	9.5742
433.3707	2.8275

Frequency (cm⁻¹)	IR Intensity (KM/Mole)
439.1626	3.9114
459.0315	20.1535
476.1762	10.7919
496.3010	99.5068
522.5779	122.9378
531.8458	34.8375
550.4281	7.3327
585.0810	31.3704
598.4944	24.2968
629.5264	2.9107
639.0476	17.6623
641.6999	162.0015
659.2038	120.9548
665.8549	40.1585
679.4160	29.1526
698.1328	18.7454
707.2352	54.1546
710.1583	62.8903
741.9769	36.8563

Frequency (cm^{-1})	IR Intensity (KM/Mole)
754.7216	21.2927
766.2042	119.7585
787.0131	28.5978
793.8976	11.0981
809.6037	10.5355
827.6796	0.5689
852.1949	15.0530
861.8898	0.8128
877.4775	4.9155
900.4564	23.4468
924.1213	4.9031
926.4288	2.1060
930.2312	35.6533
941.2248	0.9500
950.4648	3.3013
953.5620	24.1036
960.3587	19.5392
963.1962	224.7623
999.3976	0.9060

Frequency (cm⁻¹)	IR Intensity (KM/Mole)
1001.5520	127.6132
1010.7226	14.3995
1014.0161	3.7192
1015.2834	0.7779
1024.3863	0.4472
1027.7010	0.4650
1041.9399	10.1184
1045.4018	30.9114
1079.9307	1.7522
1083.1155	37.4165
1090.6791	0.9556
1095.2393	1.7247
1102.4890	2.0621
1105.2295	29.0951
1116.5699	2.0649
1126.9118	145.1967
1133.8239	29.1217
1145.8309	3.2183
1177.6167	0.5731

Frequency (cm⁻¹)	IR Intensity (KM/Mole)
1182.6034	2.7193
1186.2263	12.9861
1197.7991	18.2518
1202.9872	21.0784
1213.9476	42.4804
1251.9262	4.2685
1260.6952	59.7801
1275.4380	51.5994
1300.7128	24.8315
1304.5990	1.9631
1318.2864	10.4144
1324.2597	11.7894
1325.3693	29.9513
1329.2548	131.1869
1349.8628	0.1741
1351.5202	9.2517
1354.6174	2.9110
1373.2569	7.0062
1379.9823	44.6356

Frequency (cm^{-1})	IR Intensity (KM/Mole)
1384.5261	8.7834
1392.5895	5.9827
1404.2125	26.4397
1408.7071	10.4783
1431.5008	6.9390
1464.5893	280.7554
1470.3535	696.6341
1474.2655	141.5640
1478.5763	19.0685
1480.4742	5.2844
1482.3697	23.5556
1485.0869	52.3117
1488.9654	6.8297
1489.7138	20.2277
1492.8009	12.9780
1499.4981	14.2478
1513.5755	548.3151
1526.1063	18.0385
1553.2968	631.2962

Frequency (cm^{-1})	IR Intensity (KM/Mole)
1599.9047	53.5722
1615.9561	177.0285
1621.9494	123.6888
1637.3368	1.4187
1667.6265	490.2196
1671.8824	511.8111
2982.4714	58.8108
2992.3588	51.7213
3004.1561	60.0894
3009.4668	53.6191
3017.6807	4.7172
3030.0386	20.6104
3037.3883	27.2239
3039.3453	78.0829
3061.3203	9.3693
3062.6103	26.2596
3069.4760	161.6561
3072.7850	79.4179
3085.4123	65.1452

Frequency (cm⁻¹)	IR Intensity (KM/Mole)
3103.2450	39.4859
3118.6851	31.3232
3166.8274	0.9410
3175.0083	9.6068
3183.4425	41.4600
3186.6453	2.9571
3191.8221	22.6499
3202.5461	3.3160
3208.3806	9.1019
3221.4958	1.7612
3471.0207	277.4710
3493.1711	1038.1416
3528.2380	136.7303
3809.4827	21.2824

Delanzomib deprotonated (de2) in water

Frequency (cm^{-1})	IR Intensity (KM/Mole)
10.5955	0.7733
18.8573	3.0282

Frequency (cm^{-1})	IR Intensity (KM/Mole)
24.4411	2.7741
35.5556	0.4257
39.6995	0.3244
46.8163	4.9549
53.4004	3.4450
56.3560	3.0962
59.1732	3.1450
70.2442	4.1878
79.0377	2.2809
96.4894	2.7791
100.1436	8.1888
111.0019	18.7135
121.8880	11.5407
150.5416	2.7596
166.5357	3.0497
174.8987	12.9989
188.7820	5.6172
215.4859	11.1996
226.2176	0.2599

Frequency (cm^{-1})	IR Intensity (KM/Mole)
235.0337	7.4095
245.2549	2.2768
254.1978	12.1297
257.7192	7.1340
272.3545	14.2473
287.5212	0.6290
304.4851	2.7438
320.0880	13.8792
322.0380	13.1076
366.7134	37.1111
381.1736	17.4921
386.5752	19.8632
392.2906	0.5833
413.9890	0.8337
421.7399	9.6037
433.3188	3.1855
438.9336	4.0372
458.0713	22.6990
475.5827	10.6878

Frequency (cm^{-1})	IR Intensity (KM/Mole)
496.0322	102.9576
518.8952	133.7068
530.8885	26.1737
549.9971	6.6680
584.4769	35.5431
596.6177	21.9980
629.3433	6.7302
637.4532	173.3609
639.1985	34.6416
653.6819	92.4426
666.1750	38.3363
678.7280	28.5002
697.8255	27.7189
706.1257	56.9184
708.6495	67.0891
741.8870	36.7771
754.9888	21.1641
765.6798	121.6540
788.0297	26.9902

Frequency (cm^{-1})	IR Intensity (KM/Mole)
793.6915	12.1257
809.1094	11.7675
827.9170	0.6196
852.2393	15.0613
859.6527	0.7818
877.6997	6.4505
900.2566	22.5231
924.0703	4.3168
926.7062	2.0907
930.2476	35.0027
940.5242	1.3007
949.5145	2.7557
953.3825	28.7258
960.0666	18.9426
963.1060	231.3544
997.8186	1.1635
1001.2144	121.7115
1010.6482	14.3558
1013.8698	1.1494

Frequency (cm⁻¹)	IR Intensity (KM/Mole)
1014.3590	3.5126
1024.7071	0.4545
1027.6234	0.4893
1042.1910	9.7475
1045.6555	33.0315
1079.4810	0.4245
1082.6518	38.0325
1090.6081	1.6164
1094.8218	1.8374
1102.5512	1.5694
1105.6437	29.5725
1117.1480	2.0734
1126.2042	150.0614
1133.9496	29.9379
1145.7170	2.9765
1177.4440	0.6080
1182.7466	2.2142
1185.8793	12.7583
1196.1327	23.1436

Frequency (cm^{-1})	IR Intensity (KM/Mole)
1202.8499	19.2797
1214.7155	42.3333
1252.7178	3.7901
1260.2810	63.4380
1276.3962	50.0601
1300.9542	25.1444
1304.3761	1.5302
1318.2673	8.2200
1324.2998	9.9166
1325.4816	33.5413
1329.2859	132.9211
1350.2517	2.3721
1350.8632	8.2334
1354.7367	2.4959
1372.8353	5.9616
1379.8522	45.0015
1384.2009	9.7106
1392.5721	5.9617
1404.1749	27.1342

Frequency (cm^{-1})	IR Intensity (KM/Mole)
1408.8894	10.7091
1431.4962	7.1653
1463.8481	284.2922
1468.7253	718.8591
1473.8962	144.7112
1478.3148	15.1980
1480.5661	7.2681
1481.8984	18.0876
1485.5272	50.8631
1488.4591	4.7170
1489.7016	20.5095
1492.3306	15.0368
1499.3375	15.0874
1513.7199	593.7878
1526.0206	19.1610
1550.2356	610.4309
1599.7449	53.8176
1615.8128	191.4181
1621.6943	123.5513

Frequency (cm^{-1})	IR Intensity (KM/Mole)
1637.3750	1.6137
1665.7128	467.9286
1670.0971	541.4615
2981.7509	62.8776
2992.9452	52.9787
3004.5025	60.9814
3009.7466	53.1201
3018.2463	4.4058
3030.7264	22.1228
3037.2604	27.2600
3040.4971	80.8934
3061.7994	10.3438
3063.6915	26.7351
3069.7626	162.9330
3073.1177	81.5139
3085.8841	66.2457
3102.8799	40.7611
3119.1508	31.3676
3167.1846	1.0806

Frequency (cm^{-1})	IR Intensity (KM/Mole)
3174.9992	9.0367
3183.7157	39.7985
3187.3608	3.9684
3192.2530	23.4747
3202.6950	4.9308
3208.9970	8.6595
3222.0086	1.6729
3478.6227	272.4895
3492.9647	1052.1273
3531.5789	138.0899
3808.6673	22.1748

Ixazomib calculations at level of theory - B3LYP/6-311+G(d,p)

Ixazomib in gas phase

Frequency (cm^{-1})	IR Intensity (KM/Mole)
8.6579	0.0046
9.8638	0.1450
27.1720	0.1900
28.7518	0.1885

Frequency (cm^{-1})	IR Intensity (KM/Mole)
35.9380	0.1462
39.6761	2.6926
68.0240	7.4729
71.1204	2.2422
91.0677	1.6890
100.9883	0.7356
103.6902	4.5258
138.2503	9.4447
147.0002	15.2655
156.9609	0.9002
180.0383	10.6312
194.6391	6.4836
209.2147	1.0074
223.2718	0.4130
236.9496	2.0300
252.3667	0.6503
271.1113	1.9054
293.3405	12.1807
295.9115	8.1357

Frequency (cm⁻¹)	IR Intensity (KM/Mole)
322.1748	3.7649
330.2886	0.9488
348.4451	4.0430
371.3868	10.5755
373.5178	1.4293
394.5602	8.2529
410.3901	2.8030
419.6467	0.6015
434.2678	39.1606
466.0022	25.9324
482.8256	47.5230
494.9436	83.9231
521.9088	30.5525
536.9527	70.4959
545.5995	178.6539
577.6537	12.2004
612.2056	26.7725
617.2846	2.8221
639.1348	10.5155

Frequency (cm^{-1})	IR Intensity (KM/Mole)
670.0415	15.1851
702.3550	6.5553
731.9105	2.8387
747.4435	28.3317
779.2806	2.7765
799.0861	7.7949
830.1153	30.4099
833.1067	2.4308
890.7762	3.4977
904.9729	12.1169
926.3622	5.1835
930.0098	9.8579
946.6976	142.4545
958.3829	12.0790
963.6447	0.3500
968.3391	0.4000
980.7869	55.5350
1000.7122	81.6498
1007.8764	0.7884

Frequency (cm^{-1})	IR Intensity (KM/Mole)
1031.7124	19.5446
1055.0169	1.1551
1061.4081	55.2468
1111.3600	4.2543
1113.5023	64.8818
1121.2970	8.8085
1149.2847	2.5728
1158.7684	7.2332
1188.6394	15.5298
1190.0947	1.3870
1217.7615	14.8264
1240.9618	77.5715
1248.3194	18.1057
1264.4547	37.8599
1271.9041	9.0868
1280.2251	9.8741
1305.6306	18.6024
1308.2812	1.7403
1349.2140	55.5586

Frequency (cm^{-1})	IR Intensity (KM/Mole)
1362.2989	37.9055
1369.5705	68.6382
1381.1663	181.0711
1389.8980	59.9907
1395.3963	173.5337
1401.4571	8.2941
1407.7663	19.7475
1421.3223	4.3418
1483.2554	2.0845
1486.9564	124.4825
1490.7310	1.8596
1497.5068	2.4980
1499.5757	8.3207
1505.2411	4.2276
1511.2446	26.4946
1520.0721	648.7889
1566.9900	141.5553
1595.0145	10.1503
1620.8012	4.2961

Frequency (cm⁻¹)	IR Intensity (KM/Mole)
1713.7353	359.6514
1734.9910	171.6665
2988.0479	28.2239
3010.1572	27.5668
3013.5836	26.9357
3024.3149	33.0250
3030.4532	9.9681
3031.4834	10.9046
3049.4093	6.5912
3060.6424	19.4761
3069.1430	7.1799
3078.5050	76.4281
3082.6298	39.5247
3085.8328	49.6004
3194.7294	0.5079
3197.0111	0.0044
3208.3529	0.6870
3572.1094	125.9067
3603.8464	27.9013

Frequency (cm⁻¹)	IR Intensity (KM/Mole)
3862.6141	14.4228
3867.4936	75.3985

Ixazomib in diethyl ether

Frequency (cm⁻¹)	IR Intensity (KM/Mole)
13.6777	0.0880
16.9574	0.1527
27.5404	0.3505
34.1565	0.5233
41.9230	0.4494
45.2929	2.6349
70.1036	4.3156
73.2705	12.3536
90.2371	3.7306
100.1839	4.5893
105.1196	0.8036
125.9669	8.7059
145.2452	21.7945
156.8571	1.8125

Frequency (cm^{-1})	IR Intensity (KM/Mole)
180.9748	10.9651
196.4798	10.1075
210.1990	1.3563
224.1601	0.5936
236.8495	2.6297
254.1268	0.8432
276.0427	3.0798
293.9665	8.3367
296.1684	11.9292
323.0251	4.5562
330.8357	1.6700
351.4728	4.0725
371.6985	14.7208
394.3369	10.4885
412.0985	3.6770
418.8960	1.9672
421.2829	7.2601
439.8081	41.8509
469.7627	32.8351

Frequency (cm^{-1})	IR Intensity (KM/Mole)
487.3415	66.9716
505.0178	91.0072
522.5096	62.7072
534.7523	88.0047
546.7615	262.4224
579.9781	26.5770
611.9201	30.3095
620.6162	2.9152
637.0662	10.8405
668.6550	16.5098
702.5223	7.4176
731.0077	3.9145
748.5676	34.7858
778.0729	3.0714
796.7246	9.1774
831.3529	41.1873
832.2903	3.7685
889.8695	3.7535
906.2404	18.1845

Frequency (cm^{-1})	IR Intensity (KM/Mole)
925.2386	10.2227
928.6186	14.3857
953.9216	66.7773
961.6863	154.8065
962.6933	6.2553
973.1897	1.3159
978.0848	55.8721
1005.5548	50.0004
1008.2215	44.5300
1035.2510	44.3321
1055.6769	77.0051
1056.8319	20.6866
1108.5676	14.0202
1111.3594	81.7618
1119.8864	15.9380
1148.4392	3.3594
1156.9264	9.6015
1186.3141	15.6139
1189.7935	3.7986

Frequency (cm^{-1})	IR Intensity (KM/Mole)
1217.6910	15.3319
1247.7144	18.3086
1250.9647	79.5246
1269.2919	64.6942
1273.5701	12.7580
1282.3733	13.6185
1304.6617	24.8794
1308.2351	7.1582
1344.2451	161.2131
1358.8257	138.7594
1366.1077	245.7028
1370.8105	41.1794
1384.6515	14.4733
1394.9347	144.2129
1397.4187	23.7783
1406.9898	28.7198
1415.5087	2.5576
1480.7977	0.5569
1485.2841	16.4823

Frequency (cm^{-1})	IR Intensity (KM/Mole)
1485.8728	132.2604
1492.8197	10.4538
1493.2789	26.9751
1497.5139	3.5698
1503.7292	25.0861
1524.5302	802.5111
1569.0482	151.4847
1592.4146	18.2760
1620.3976	8.1782
1687.1131	738.0567
1706.9236	151.7273
2996.1801	33.8003
3008.8692	41.9124
3012.7748	33.8104
3028.0764	21.7514
3032.7122	19.4108
3033.8985	24.4357
3059.5469	31.0913
3062.6268	7.1076

Frequency (cm⁻¹)	IR Intensity (KM/Mole)
3067.8221	11.5016
3076.0773	116.0816
3081.2665	42.7624
3082.3326	78.4974
3198.4589	0.4487
3199.4785	0.1139
3211.2942	0.4399
3566.5587	173.2899
3600.7357	49.8766
3844.2251	13.1473
3846.6618	133.5927

Ixazomib in methanol

Frequency (cm⁻¹)	IR Intensity (KM/Mole)
7.8343	0.1230
12.6269	0.2126
25.7545	0.5428
31.0415	0.7578
37.8393	0.4423

Frequency (cm^{-1})	IR Intensity (KM/Mole)
43.6985	4.3069
69.7909	2.5837
73.7375	18.1167
89.2259	4.8936
97.6969	3.2879
103.8146	0.5081
115.5302	12.1733
140.7282	22.3806
157.5909	1.8931
180.4209	11.6042
195.6319	13.0221
210.0131	1.4522
224.7907	0.4906
236.9341	2.8041
254.9103	1.1538
278.4249	4.1784
293.9179	6.6752
296.4733	11.9805
322.9300	4.3691

Frequency (cm^{-1})	IR Intensity (KM/Mole)
330.6267	1.7606
353.7206	3.7000
371.5902	16.2361
393.8108	13.6771
411.8103	2.7301
418.5592	3.3894
428.0795	12.2336
441.9700	47.6647
472.5365	46.7964
493.3051	53.1256
516.1718	84.8957
523.1817	118.5573
532.9859	105.8289
546.3983	310.2210
579.2709	42.0259
612.3710	27.7388
624.6430	2.8635
636.8804	10.0385
668.3607	16.9784

Frequency (cm^{-1})	IR Intensity (KM/Mole)
704.2380	8.1592
731.3074	4.4055
749.3590	38.7071
777.0342	3.0750
795.5726	8.7836
831.8542	49.7004
832.5687	4.0442
888.5826	3.5667
906.2411	23.3674
924.2472	17.7055
927.9555	14.8111
954.0190	19.9951
962.4786	1.5393
966.8722	249.2372
975.4512	4.8965
977.2035	47.0456
1006.6235	10.8225
1013.0255	71.6170
1040.9141	83.3281

Frequency (cm^{-1})	IR Intensity (KM/Mole)
1053.5260	107.6973
1060.0555	17.5318
1107.3005	29.0510
1109.8208	84.9539
1119.6073	19.1638
1148.4249	3.9458
1156.0103	11.4787
1185.5656	15.4554
1189.8861	4.2581
1219.0143	12.6046
1251.4054	1.0918
1254.0535	92.3553
1273.2126	78.4718
1276.9684	14.6546
1282.4529	15.4173
1305.1415	25.7388
1308.0174	11.0725
1342.1449	295.6591
1355.3678	211.3391

Frequency (cm^{-1})	IR Intensity (KM/Mole)
1363.4637	154.0691
1371.2044	38.3218
1382.2012	6.9730
1394.9685	59.4114
1396.5108	113.6505
1406.8913	33.3541
1413.4175	1.7599
1478.4288	0.6877
1481.9026	4.3395
1484.9781	150.1662
1489.1247	4.1703
1490.3822	54.7941
1493.7141	3.5394
1500.3710	27.4044
1526.9487	855.1158
1569.8798	164.0104
1591.1223	25.0756
1620.1895	10.6581
1669.6147	979.1583

Frequency (cm⁻¹)	IR Intensity (KM/Mole)
1693.4399	170.4186
3001.2766	33.8226
3008.2281	53.3596
3012.8469	37.3569
3025.8658	32.3079
3035.6638	14.5308
3037.7552	29.1642
3056.9092	45.5963
3067.2456	17.2311
3068.0294	9.0806
3074.7947	139.7232
3079.0609	70.0865
3081.6097	73.7431
3200.1345	0.4961
3201.0579	0.1763
3212.6341	0.3562
3563.0456	205.1311
3599.4190	67.3369
3835.1862	26.2901

Frequency (cm^{-1})	IR Intensity (KM/Mole)
3836.8644	149.5966

Ixazomib in water

Frequency (cm^{-1})	IR Intensity (KM/Mole)
6.3253	0.1885
11.2333	0.2057
24.9350	0.5250
29.9510	0.7519
35.9810	0.1893
42.2692	5.1498
69.6081	2.6109
73.3385	18.2073
89.1571	4.9341
97.3986	2.9976
103.5029	0.5033
114.2945	13.2780
140.4070	22.5922
157.4588	1.9120
180.2606	11.5529

Frequency (cm^{-1})	IR Intensity (KM/Mole)
195.4323	13.4871
209.5268	1.4140
224.3646	0.5279
236.8423	2.7973
254.6032	1.2032
278.2440	4.3283
293.8188	6.7858
296.4254	11.8268
322.8398	4.3352
330.5893	1.7852
353.7477	3.4840
371.5508	16.0044
393.6330	14.6607
411.6690	3.3204
418.3847	3.3244
424.9314	10.2624
439.9963	52.7719
472.2383	46.4918
492.6896	57.3625

Frequency (cm^{-1})	IR Intensity (KM/Mole)
515.6516	88.2383
523.2417	126.5880
532.4530	103.7718
545.7907	310.5756
579.2691	44.0916
612.2467	27.6882
624.8670	2.9709
636.6418	10.0896
668.2455	17.0631
703.9578	8.0551
731.4241	4.3965
749.3367	39.5316
776.9162	3.0276
795.4540	8.7480
831.9143	50.6773
832.5515	4.2641
888.6409	3.5121
906.1896	24.0907
924.1729	18.3453

Frequency (cm^{-1})	IR Intensity (KM/Mole)
927.8650	14.9993
953.9234	19.6502
962.4028	1.6547
966.9476	253.7598
975.6747	7.1650
977.0895	45.2774
1006.5565	12.3283
1012.6198	73.3709
1040.3352	82.4317
1053.2018	110.2314
1059.6725	15.5413
1107.0126	29.1179
1109.6746	85.9051
1119.4225	19.8887
1148.2937	4.1338
1155.9290	11.6521
1185.4291	15.4697
1189.9683	4.3025
1218.6862	13.0559

Frequency (cm⁻¹)	IR Intensity (KM/Mole)
1251.6890	0.9506
1254.4752	92.3103
1273.3421	80.6176
1276.7149	14.7448
1282.5344	15.7639
1304.8968	26.1214
1307.9891	11.9003
1341.4211	318.7336
1354.8935	212.2247
1362.9398	142.3610
1371.1501	40.3720
1381.8922	6.6357
1394.7744	57.6224
1396.2867	115.6043
1406.8972	33.5620
1413.1020	1.7237
1478.1486	0.5681
1481.6199	4.4749
1484.8947	149.8415

Frequency (cm⁻¹)	IR Intensity (KM/Mole)
1488.8776	5.2161
1490.1059	57.0371
1493.2766	3.5856
1499.7396	27.8990
1527.3510	862.5013
1569.8307	164.7759
1591.0030	25.9891
1620.1806	11.0006
1667.5629	1008.4568
1691.9575	174.0528
3001.5945	33.8676
3008.1384	54.8535
3012.8577	37.9466
3025.7055	33.5428
3036.2397	14.3983
3038.0989	29.1826
3056.8452	47.3565
3067.1798	18.4317
3068.5186	9.4738

Frequency (cm^{-1})	IR Intensity (KM/Mole)
3074.6417	141.9432
3078.7210	73.2435
3081.5624	73.8483
3200.2974	0.5101
3201.2167	0.1788
3212.7582	0.3488
3562.3864	209.1930
3599.1045	69.6871
3833.8257	14.7012
3835.5242	163.9295

Ixazomib deprotonated (de1) in gas phase

Frequency (cm^{-1})	IR Intensity (KM/Mole)
9.6739	0.0651
12.2194	1.3334
26.0716	0.2333
32.7553	0.4306
37.2181	0.8004
40.0430	7.0194

Frequency (cm^{-1})	IR Intensity (KM/Mole)
61.8400	3.3376
71.1121	0.5537
87.3174	7.3753
92.5706	0.0486
98.0374	6.7571
106.0122	0.7185
145.3441	7.7332
160.6367	4.2641
178.3624	19.6187
200.2873	4.1857
214.5518	2.4240
218.6188	0.5777
227.4783	2.1308
264.5825	0.6745
269.2722	3.8024
294.2944	2.2969
299.9489	6.5491
323.0431	6.9076
328.6808	1.3287

Frequency (cm^{-1})	IR Intensity (KM/Mole)
360.6235	6.1059
373.2277	29.4125
385.3633	1.3856
403.1984	0.2785
421.8565	4.3931
429.0340	11.7793
473.7154	34.9895
476.1255	43.0089
523.3726	79.0548
540.3678	40.8176
565.3659	17.9204
590.2416	16.3341
621.4778	8.0674
649.0867	22.1365
665.3697	8.6813
674.5664	53.1366
686.7746	3.3452
700.9774	6.4764
731.6165	2.0204

Frequency (cm⁻¹)	IR Intensity (KM/Mole)
734.0838	26.6780
780.0061	14.8877
795.6228	11.8805
821.1232	30.9122
826.2019	0.1560
897.0514	85.6341
905.0127	17.1241
912.4644	66.6257
929.4427	1.9314
944.2450	131.7285
952.3270	0.3141
958.3553	0.1245
962.0476	2.9027
977.2575	91.6519
1001.2682	3.2962
1006.0765	6.8501
1047.0388	5.0510
1060.1855	71.4253
1086.8851	12.1352

Frequency (cm^{-1})	IR Intensity (KM/Mole)
1101.9645	27.2991
1109.6454	14.4576
1110.6965	62.7318
1149.2004	5.1430
1154.6895	5.1534
1187.1594	10.4688
1187.8196	5.3151
1212.8516	12.7228
1233.2784	1.4913
1243.2267	7.2201
1270.6606	43.0019
1278.3321	10.1917
1301.7148	9.9835
1305.5540	8.4464
1310.7981	14.5690
1356.1258	10.4152
1363.6969	2.1901
1385.8628	1.6403
1388.4981	9.8225

Frequency (cm⁻¹)	IR Intensity (KM/Mole)
1399.3668	5.9568
1404.8029	19.7625
1408.4721	7.9055
1481.1985	0.5330
1485.1181	135.9228
1488.9043	2.0587
1496.1904	1.8953
1499.8045	7.0995
1500.6412	7.3939
1508.4061	6.1536
1518.2463	1103.9144
1541.8053	236.4223
1574.3334	232.2468
1593.7640	10.5252
1619.6830	1.7561
1706.9595	345.3032
1724.2335	297.9910
2977.7138	35.4830
2989.1375	64.2321

Frequency (cm^{-1})	IR Intensity (KM/Mole)
2997.0851	79.1556
3007.5347	20.1216
3028.2666	22.1034
3034.4737	23.0398
3041.5453	26.8542
3042.8508	2.6605
3058.7648	104.0484
3062.8343	51.2577
3064.6622	9.3039
3092.8597	30.7043
3190.7907	1.2736
3198.3840	0.0354
3205.3810	3.2249
3489.4797	250.3683
3539.0095	156.9488
3837.1224	12.7371

Ixazomib deprotonated (de1) in diethyl ether

Frequency (cm^{-1})	IR Intensity (KM/Mole)
14.1669	0.3349
26.5294	0.2242
33.6093	1.8749
39.5558	3.4702
54.8263	6.3037
68.8981	6.9455
71.3958	2.0133
86.8129	9.6195
92.3497	1.1415
106.4273	2.0711
108.7702	3.7718
112.7643	7.2538
151.2526	11.7283
173.4577	5.6505
184.8694	14.7055
199.6363	8.0953
213.2532	1.5248
227.7630	2.1353

Frequency (cm^{-1})	IR Intensity (KM/Mole)
241.5014	1.5606
257.4384	0.9012
277.6189	0.2568
294.8890	9.3055
301.2724	7.3647
322.8990	4.6890
329.5740	3.1156
358.8319	12.3215
373.9007	29.2335
394.3036	2.7008
408.2671	2.4262
424.6007	4.6308
444.9548	6.1599
476.0642	29.7972
492.0676	78.5238
522.7241	130.3920
536.3256	52.8676
558.0152	64.4778
579.3020	22.9668

Frequency (cm^{-1})	IR Intensity (KM/Mole)
617.0089	37.6068
624.3193	51.2149
657.1825	13.6322
663.1958	9.1092
675.8488	22.7958
689.1135	18.4510
730.8993	4.9311
736.3579	17.5300
776.1579	11.9596
792.7234	18.7811
828.5175	42.2383
830.6744	0.6784
890.8884	108.6779
904.6171	9.3252
913.7593	60.1042
941.8393	4.6856
951.9868	160.6931
956.0152	0.5937
965.5313	3.1166

Frequency (cm⁻¹)	IR Intensity (KM/Mole)
970.2018	0.1923
987.8859	138.0727
1005.6904	4.7547
1016.5546	7.2272
1040.5458	7.1673
1055.7546	113.2797
1090.9021	1.4181
1108.3044	69.2363
1109.9468	74.7539
1113.2544	23.5121
1152.3694	12.9738
1155.8422	6.3717
1187.9821	3.7242
1191.9609	9.9122
1216.2414	14.5898
1243.6223	4.5794
1247.8310	15.9757
1264.3175	53.0666
1280.7919	11.9151

Frequency (cm⁻¹)	IR Intensity (KM/Mole)
1302.1512	17.2257
1309.1449	16.1672
1312.1345	3.2639
1358.1729	0.4380
1370.4543	12.1963
1378.9298	9.8355
1388.1688	4.3435
1397.3863	7.5525
1406.4881	29.8930
1417.6473	10.6018
1481.1070	4.9405
1485.1904	185.8567
1487.1718	2.8517
1491.6761	1.5450
1494.9203	69.4421
1499.9540	9.7057
1509.9926	3.1628
1516.2985	1675.5465
1529.2447	143.1622

Frequency (cm^{-1})	IR Intensity (KM/Mole)
1546.6064	53.9634
1592.3676	17.6040
1620.6224	7.0392
1677.4234	690.0582
1697.8934	274.9702
2985.3451	49.2940
3001.6840	51.4061
3007.5393	62.8253
3018.4622	18.3065
3034.0693	16.8797
3035.3856	29.7621
3058.0045	6.2173
3066.0671	10.1044
3067.8827	150.5621
3070.2587	21.2997
3072.7383	73.0028
3096.0906	45.4588
3197.5508	0.7458
3200.5297	0.0565

Frequency (cm^{-1})	IR Intensity (KM/Mole)
3210.5105	0.9323
3531.9554	225.4403
3572.0938	141.2321
3832.7732	21.7793

Ixazomib deprotonated (de1) in methanol

Frequency (cm^{-1})	IR Intensity (KM/Mole)
10.6112	0.4613
14.2627	1.4945
28.1532	0.7785
33.3294	1.7973
43.3511	1.4890
45.1436	11.6207
64.2277	6.6492
77.9391	3.7273
83.5072	21.3274
90.7945	5.2895
93.6889	6.2814
104.1900	0.3931

Frequency (cm⁻¹)	IR Intensity (KM/Mole)
141.7561	10.9027
158.6067	3.5683
180.8083	16.3673
196.1855	11.9151
211.1340	1.6023
227.6695	1.5570
230.6487	1.4560
254.8496	1.2754
267.3152	0.2704
293.9665	10.4964
298.3239	7.2127
319.8328	6.4249
328.0829	2.7022
356.7558	13.9251
372.9916	28.0159
390.9113	5.3962
404.7124	2.2828
420.1847	6.2965
442.8707	11.5595

Frequency (cm^{-1})	IR Intensity (KM/Mole)
475.6731	35.3749
487.3277	94.4030
514.9700	158.3902
531.8703	69.8992
551.6347	102.3514
575.2290	27.1769
599.3685	94.7992
620.6373	17.9809
650.3799	6.2665
659.5604	20.4073
676.1021	22.9053
687.7411	21.2065
730.7054	3.0557
736.4813	18.8730
774.6515	11.8093
792.0436	20.2427
824.3049	0.8693
830.5853	50.3427
890.0635	108.0581

Frequency (cm^{-1})	IR Intensity (KM/Mole)
903.8966	9.8009
913.9276	71.5836
925.0855	2.3434
951.9732	185.0070
954.3787	16.1058
961.1978	9.3302
974.3526	0.2764
985.4032	126.3850
1004.9863	3.8323
1015.0295	5.9190
1037.0385	2.3074
1053.2032	134.3413
1091.4715	7.0116
1106.9345	123.4455
1108.8610	82.4312
1112.9689	21.2919
1147.2618	17.1311
1155.2440	9.2648
1185.2518	8.7286

Frequency (cm^{-1})	IR Intensity (KM/Mole)
1188.6050	4.6493
1214.3877	18.1175
1246.8878	1.1847
1250.3051	30.6291
1265.2040	58.0874
1281.7029	12.8014
1301.6083	20.5361
1305.3040	14.2522
1309.5653	8.8217
1352.3033	0.6051
1370.2112	14.5372
1371.5180	12.9632
1384.7080	4.7870
1392.5168	9.1046
1406.1486	35.4252
1408.2555	10.0089
1477.4226	11.6651
1482.1243	3.4847
1484.5745	188.5247

Frequency (cm^{-1})	IR Intensity (KM/Mole)
1487.7958	53.4831
1489.9020	153.4107
1492.0534	7.1397
1500.1570	382.4899
1503.2236	817.1737
1523.9547	681.7786
1546.1536	53.7384
1590.9720	24.5473
1620.3129	10.5647
1661.5917	964.3810
1685.1292	246.9271
2988.9855	54.8598
3004.4540	56.6996
3008.5822	43.2647
3016.2887	19.2828
3027.7071	56.1697
3037.2831	31.6286
3061.8588	7.4333
3067.3570	33.0380

Frequency (cm⁻¹)	IR Intensity (KM/Mole)
3068.4122	11.6944
3068.8462	157.6394
3073.5845	92.4712
3087.5170	64.9345
3200.1674	0.8229
3201.2491	0.0354
3212.6648	0.5006
3543.9132	234.7464
3577.7700	145.5203
3825.0682	26.0817

Ixazomib deprotonated (de1) in water

Frequency (cm⁻¹)	IR Intensity (KM/Mole)
6.6177	1.4947
12.3245	1.3277
25.7765	1.4246
31.7975	0.4613
39.8849	14.2366
49.8316	0.0744

Frequency (cm⁻¹)	IR Intensity (KM/Mole)
60.9546	6.2709
76.7159	1.2340
81.8280	27.1037
89.5965	6.4353
93.0420	2.6757
102.9160	0.3674
142.0939	11.0136
158.7554	3.2566
179.7827	16.3470
195.4964	12.1815
210.9844	1.8081
225.5133	0.8388
227.8471	2.2481
254.2695	1.6464
264.9177	0.2841
293.6806	10.6074
298.1645	7.4409
320.0324	5.8527
328.1425	2.6279

Frequency (cm^{-1})	IR Intensity (KM/Mole)
356.6083	13.4172
372.8015	27.5263
390.5065	6.0686
404.5009	2.3101
420.1248	6.7771
442.8219	13.0068
475.6735	35.9769
486.4685	99.2067
512.0421	159.0380
530.9401	66.9947
550.6022	112.2973
574.1263	28.9587
596.5525	97.8496
620.3495	16.6028
649.6798	6.2273
659.2264	21.1657
676.3511	22.4031
687.3769	22.1687
730.4857	3.3590

Frequency (cm⁻¹)	IR Intensity (KM/Mole)
736.1516	18.2258
774.3954	11.9804
791.9840	20.4480
824.4305	0.9475
830.6723	51.3919
889.9310	108.2291
903.5787	9.9047
913.8027	73.5358
924.1850	2.2580
951.8210	190.8315
954.2581	15.3774
960.6594	8.0655
974.6270	0.2741
984.7967	120.9682
1004.5698	4.0575
1014.9137	6.0276
1036.4776	1.7736
1052.8630	136.4341
1090.9814	9.6478

Frequency (cm⁻¹)	IR Intensity (KM/Mole)
1107.4506	131.0868
1108.8903	82.2911
1113.0301	23.3914
1147.1038	18.7979
1155.3901	9.6477
1185.1495	8.8965
1188.6994	4.5923
1214.5472	17.7617
1247.0878	1.5448
1250.4337	31.3033
1265.1886	57.3678
1281.8085	12.9701
1300.6248	21.9250
1304.9531	14.7944
1309.4721	8.1754
1350.8343	1.2306
1370.0364	14.6879
1372.3828	14.1737
1384.1368	4.7902

Frequency (cm⁻¹)	IR Intensity (KM/Mole)
1392.3716	9.2207
1406.1780	36.1827
1408.0166	9.9076
1476.7259	14.5750
1480.9632	2.2942
1484.3834	183.5850
1486.9158	69.1315
1489.0813	167.5784
1491.7529	7.5897
1498.7259	375.0823
1501.1161	769.6753
1524.1596	728.5966
1546.5782	56.8768
1590.8342	25.4692
1620.2502	11.1226
1659.8143	998.6734
1683.8096	244.8773
2988.4790	56.1927
3004.8354	56.5667

Frequency (cm⁻¹)	IR Intensity (KM/Mole)
3008.6086	39.9828
3015.7801	22.9616
3026.9603	59.9710
3037.5100	31.7775
3062.2562	5.0754
3065.3504	42.5434
3068.7032	12.4421
3069.1569	158.7122
3073.8688	89.5145
3087.5738	66.1713
3200.5324	0.8440
3201.5406	0.0431
3213.0072	0.4898
3545.1123	236.6183
3578.3911	145.5559
3823.5097	27.1663

Ixazomib deprotonated (de2) in gas phase

Frequency (cm⁻¹)	IR Intensity (KM/Mole)
9.6372	0.1007
12.7742	0.7473
25.5380	0.2247
32.0748	0.1548
37.4916	1.4380
51.0493	0.6191
68.2359	4.6596
70.9716	2.6739
92.5590	0.4836
96.0723	6.5255
99.5972	2.5764
109.6449	2.6056
145.5392	15.3304
160.5457	1.2147
178.5928	27.6277
200.8112	4.1604
214.7778	6.4293
222.5903	0.5900

Frequency (cm^{-1})	IR Intensity (KM/Mole)
228.3545	1.7886
259.5972	3.3480
270.1291	16.5533
294.6280	1.0627
296.4184	9.0132
321.8174	6.7784
326.9837	1.2491
354.8495	16.5989
371.9937	28.5046
388.3113	0.9253
401.5940	1.8402
421.6546	3.5164
433.3475	4.5892
474.0696	33.7267
502.3454	58.1472
541.8306	35.3625
548.4423	69.4686
574.5578	9.7156
599.7523	12.7303

Frequency (cm^{-1})	IR Intensity (KM/Mole)
623.8282	8.6212
651.6848	24.3489
667.3505	13.8327
683.1117	49.8232
690.9052	3.5773
732.3683	4.4232
736.0255	23.8214
779.8192	10.3209
795.3876	10.9419
820.1081	31.3032
826.0877	2.0949
836.6962	45.1643
899.2872	10.8018
907.9636	17.4089
925.9840	17.0206
928.4870	5.0591
952.5877	15.3932
957.2073	1.6700
961.0823	6.9810

Frequency (cm⁻¹)	IR Intensity (KM/Mole)
962.5997	223.9943
992.6363	92.6942
1003.2404	4.3764
1022.1230	31.0623
1052.9066	20.1381
1062.4696	57.7873
1091.6587	4.0780
1110.2125	62.5887
1111.0442	5.5715
1140.5948	53.0721
1147.4640	6.8456
1154.1351	4.9489
1186.9653	6.1259
1187.2098	10.7354
1207.4985	57.9410
1230.5248	1.0691
1242.8191	4.4112
1257.9852	29.9485
1277.7779	8.2176

Frequency (cm^{-1})	IR Intensity (KM/Mole)
1302.7762	4.6233
1307.8501	9.3245
1324.7094	28.8543
1354.1249	3.0705
1359.7940	0.9237
1381.3427	1.2402
1387.3352	11.7657
1393.4796	5.3357
1404.4272	19.2892
1409.6242	10.4137
1482.7477	2.3596
1484.7987	128.5760
1489.2220	3.0520
1496.6848	115.3846
1498.3060	63.7683
1500.6828	11.1184
1507.7168	766.8005
1509.7806	13.1869
1520.2428	520.5413

Frequency (cm^{-1})	IR Intensity (KM/Mole)
1546.8250	167.7559
1593.4825	10.0391
1619.3351	1.5268
1700.4336	328.4564
1723.1405	365.1222
2983.7477	26.1279
2994.4959	51.6215
3002.2929	65.1476
3009.0880	10.4471
3025.3057	41.2079
3036.0028	29.3211
3039.4201	21.4869
3046.9104	0.2994
3062.0266	85.0113
3068.0312	70.6136
3070.3202	8.4453
3094.3807	30.7918
3190.2902	1.3815
3198.5165	0.0331

Frequency (cm⁻¹)	IR Intensity (KM/Mole)
3204.9975	3.6305
3297.3592	473.7899
3481.3081	251.4478
3819.8622	7.9858

Ixazomib deprotonated (de2) in diethyl ether

Frequency (cm⁻¹)	IR Intensity (KM/Mole)
14.7324	0.0407
21.5039	1.0460
30.8656	0.0666
35.1266	2.5193
54.5299	2.1168
62.2593	4.4853
69.5427	5.2365
88.6832	3.1704
93.0259	1.6816
100.1564	0.9215
104.1506	4.0834
107.5943	11.1968

Frequency (cm^{-1})	IR Intensity (KM/Mole)
145.4795	21.8913
163.5064	1.6574
184.1802	25.8936
196.4792	7.9650
213.8765	6.4290
229.6087	2.4478
239.5535	1.1219
259.6970	11.1941
275.4082	6.1872
294.6522	9.2570
295.9867	5.6566
316.7138	5.9003
326.8146	1.8049
355.4313	20.2901
372.3327	32.7014
389.9492	0.2297
402.8415	3.7555
422.4417	3.6604
438.7001	4.4193

Frequency (cm^{-1})	IR Intensity (KM/Mole)
474.4270	43.0681
503.3198	72.5089
538.5334	140.6607
538.9973	36.8615
571.5890	17.9091
598.2683	10.7633
621.7430	11.1198
634.8354	79.4576
654.0552	18.0692
667.5471	23.7863
689.5384	9.1883
731.8966	4.8700
739.2925	25.1557
765.9495	73.1284
778.7187	12.9563
794.7554	7.8395
827.8027	29.3382
827.9762	12.9738
899.0717	9.2656

Frequency (cm^{-1})	IR Intensity (KM/Mole)
907.8523	23.5947
926.1086	18.1348
931.7500	9.3317
954.4400	19.6245
962.3624	0.6108
966.1051	298.3348
969.5770	1.4934
988.5294	80.0876
1005.9523	0.9046
1023.9206	17.8061
1050.3967	45.8913
1059.6132	70.6961
1092.7018	0.9861
1109.4439	89.6744
1114.4727	2.4985
1131.7751	106.8823
1148.2931	3.5162
1155.3106	7.8239
1188.5682	4.8896

Frequency (cm⁻¹)	IR Intensity (KM/Mole)
1189.8873	12.0606
1211.8610	51.3593
1246.7469	1.1977
1246.8374	6.1697
1259.8200	35.0112
1280.5289	11.0765
1302.1999	13.8575
1308.2713	10.8876
1329.3641	52.4809
1352.5544	2.0554
1369.5380	8.4594
1379.7612	8.4274
1389.9213	14.5595
1394.8008	4.8798
1405.8741	26.2159
1411.5809	13.9504
1482.0314	131.6209
1484.9927	231.3862
1485.1660	196.0222

Frequency (cm^{-1})	IR Intensity (KM/Mole)
1486.4258	352.6976
1493.9061	47.2563
1495.6368	19.3694
1497.6614	19.5185
1507.4398	7.8348
1518.6656	1156.6391
1537.0118	18.6676
1592.0810	17.6658
1620.3918	7.3185
1674.2945	621.7005
1696.6810	352.0764
2987.3718	41.8701
3001.5750	56.1762
3007.9854	60.2084
3015.5249	9.9440
3032.4535	44.3824
3039.3170	18.9405
3039.6259	37.2433
3057.8021	4.7348

Frequency (cm⁻¹)	IR Intensity (KM/Mole)
3069.0668	140.6406
3070.2177	10.0741
3071.0483	71.3696
3089.3160	51.2379
3197.2269	0.7496
3199.4346	0.0856
3210.0374	1.0642
3427.9137	355.0420
3531.3121	226.5069
3819.3820	18.5507

Ixazomib deprotonated (de2) in methanol

Frequency (cm⁻¹)	IR Intensity (KM/Mole)
12.0656	0.0760
13.9987	1.4392
26.3307	0.0198
31.7674	2.6008
40.4598	0.6337
48.2521	2.6218

Frequency (cm⁻¹)	IR Intensity (KM/Mole)
64.4792	10.1456
76.2754	4.6337
91.9866	2.0256
96.0056	5.8608
99.0016	13.2217
102.7412	7.1801
144.8580	22.3609
160.1877	0.9055
180.8277	28.0134
195.0345	11.9414
212.8315	6.8165
227.2132	1.1110
228.2641	3.0666
258.5873	10.6658
268.4233	4.3505
294.5073	5.5904
295.1600	9.8627
314.7897	5.2108
326.6171	1.8510

Frequency (cm^{-1})	IR Intensity (KM/Mole)
354.6866	19.5003
372.9043	31.8154
389.0520	0.5240
400.8200	3.1265
419.8461	5.5562
438.6915	9.1033
474.7779	49.5682
501.5382	80.4183
517.7374	127.5665
535.6585	100.5040
566.4019	34.6824
590.4084	6.0465
608.5059	105.7516
624.3552	32.5172
654.5893	19.3461
665.9813	20.6351
687.7638	9.1828
715.3603	81.7886
732.4731	0.3050

Frequency (cm^{-1})	IR Intensity (KM/Mole)
742.8658	47.2306
774.7736	4.8497
792.5394	10.2070
826.7570	0.0254
830.6797	50.5323
897.7337	8.5585
905.8672	25.1352
924.8213	19.6362
927.5926	8.4606
953.0605	25.2653
960.7500	0.8488
965.0039	340.4640
974.2264	0.2798
986.3641	69.9275
1005.0282	0.9610
1022.5743	15.5242
1047.1207	46.1970
1056.0306	96.6205
1088.8452	0.9518

Frequency (cm^{-1})	IR Intensity (KM/Mole)
1108.1573	103.0688
1114.8108	2.7816
1127.1345	118.6554
1146.2795	3.6381
1155.4777	9.5358
1185.4955	11.9973
1188.5752	5.4815
1212.3042	48.0680
1244.5240	2.1696
1246.4019	6.2998
1260.7891	43.1017
1280.8516	11.7320
1301.2779	11.4799
1306.8347	13.8387
1321.4257	78.9949
1348.6973	0.2731
1367.6413	10.1069
1373.8244	3.8532
1382.9444	35.7168

Frequency (cm^{-1})	IR Intensity (KM/Mole)
1392.7079	6.4527
1406.1930	29.3487
1409.2753	12.5642
1470.1847	811.6743
1479.0816	15.2187
1482.1856	19.1983
1484.3429	172.6794
1489.2239	80.4058
1489.6648	12.8396
1492.8772	15.5064
1500.1081	16.5142
1518.5314	1235.7749
1536.3169	6.4870
1590.9908	23.4313
1620.2610	10.9137
1659.9670	893.7044
1683.5331	307.7362
2989.5801	55.2941
3003.9732	59.8631

Frequency (cm^{-1})	IR Intensity (KM/Mole)
3009.4938	61.1118
3016.2820	11.1746
3032.6523	23.3728
3037.5187	22.1163
3038.5779	74.4002
3061.1729	9.9663
3069.6451	12.5755
3070.2713	163.1020
3072.7475	77.9427
3086.4245	64.7636
3199.3487	0.4544
3200.3954	0.3055
3212.2311	0.4365
3478.5722	308.2374
3542.2027	237.8233
3810.3773	21.7291

Ixazomib deprotonated (de2) in water

Frequency (cm^{-1})	IR Intensity (KM/Mole)
9.2292	1.5189
11.0481	0.1052
25.4917	0.0895
29.9782	2.7565
38.8857	0.1103
45.6805	2.7386
63.6408	9.8033
75.3499	4.5273
91.4232	4.5941
94.9130	10.8794
97.9564	10.2243
102.2005	4.5721
144.6645	22.6476
160.0113	0.9367
180.9790	27.5982
194.6456	12.3028
212.8452	7.0008
226.7517	1.0180

Frequency (cm^{-1})	IR Intensity (KM/Mole)
228.0675	3.2137
258.8407	10.3399
268.1319	4.9119
294.4034	4.9039
295.1330	10.4742
314.4738	5.3414
326.6768	1.8287
354.7652	19.1037
372.8228	32.3983
389.0644	0.4844
400.5592	3.1835
419.5168	6.0464
438.3459	9.0903
474.6251	49.7496
502.2140	83.8772
517.6979	131.3859
535.0079	105.1070
566.3161	36.6556
588.4745	2.3846

Frequency (cm^{-1})	IR Intensity (KM/Mole)
603.9751	120.4673
623.8927	26.7366
654.7662	19.1548
665.9394	19.4732
687.8080	9.9356
710.0052	84.7298
732.4048	0.5114
742.8191	45.2650
774.3819	4.7222
792.3479	10.2677
826.4018	0.0420
830.8447	51.5834
897.6561	8.7900
905.4390	25.7863
924.8739	17.9316
927.0768	10.6593
953.0554	21.9078
960.4191	0.9038
965.3848	346.3212

Frequency (cm^{-1})	IR Intensity (KM/Mole)
974.6319	0.2790
986.3510	69.6758
1005.0347	0.9939
1022.2663	16.2050
1047.0122	48.4001
1055.8072	97.2524
1089.3490	0.9061
1108.0213	104.8466
1114.9655	2.6475
1127.1726	121.0395
1146.3033	4.1252
1155.3830	9.6114
1185.2906	12.2803
1188.3804	5.3153
1212.5375	47.0632
1244.6772	3.6140
1246.2611	4.8962
1260.8054	44.1176
1280.8727	11.6574

Frequency (cm⁻¹)	IR Intensity (KM/Mole)
1301.0532	10.0037
1306.6363	15.1942
1321.0314	82.1801
1348.5915	0.2018
1367.8083	10.3516
1373.3124	3.5555
1382.8386	38.0393
1392.6994	6.2854
1406.2115	29.9550
1408.9984	12.1922
1468.4745	831.7169
1478.7412	10.4302
1482.0942	17.6634
1484.2948	172.1460
1488.8754	88.9110
1489.3800	11.9917
1492.2129	15.1931
1499.7667	17.1162
1518.5118	1240.3302

Frequency (cm^{-1})	IR Intensity (KM/Mole)
1536.2772	6.5706
1590.8731	24.1768
1620.2705	11.3164
1658.2766	928.7485
1682.0865	301.0980
2990.0092	56.5458
3004.2747	60.8281
3009.6418	60.4041
3016.6180	11.1338
3032.4650	21.6416
3037.8569	19.7055
3038.7204	83.1211
3061.6470	10.9847
3069.7164	13.0604
3070.3517	163.7453
3073.0098	80.3727
3085.2968	66.6463
3199.5940	0.4574
3200.7135	0.3217

Frequency (cm⁻¹)	IR Intensity (KM/Mole)
3212.5290	0.4016
3481.4270	307.5027
3543.0487	240.3238
3810.0904	22.1290

Threonine calculations at level of theory - B3LYP/6-311+G(d,p)

Threonine (Thr1-OH) in gas phase

Frequency (cm^{-1})	IR Intensity (KM/Mole)
59.5963	0.8323
80.5530	0.4989
162.7794	0.1303
226.9869	8.9231
238.1402	9.7849
269.9216	14.4009
293.5098	42.0271
313.2089	133.5374
374.9294	10.7096
397.9458	5.0860
483.5219	5.9413

Frequency (cm^{-1})	IR Intensity (KM/Mole)
521.4483	16.7860
617.7745	115.5733
669.5823	13.5439
757.7069	16.2277
813.7473	6.0713
841.8394	120.2825
912.1436	41.2047
956.1439	51.5718
1048.0926	15.1536
1095.2487	8.4531
1115.6628	11.1482
1125.7821	74.2424
1141.4969	238.5173
1227.7090	52.9596
1275.3949	37.0688
1288.9903	19.3265
1321.9635	28.8270
1364.9734	12.5102
1399.3658	9.3522

Frequency (cm^{-1})	IR Intensity (KM/Mole)
1429.7797	11.5732
1440.2168	17.3374
1493.7111	2.6384
1500.3178	7.0069
1670.8520	40.6568
1805.2494	276.9892
3005.2504	34.4770
3045.1765	12.6827
3055.2351	18.8278
3113.7503	16.3485
3119.9836	15.8814
3504.6804	2.7285
3580.7154	7.7508
3742.9336	59.5317
3824.1972	25.3555

Threonine (Thr1-OH) in diethylether

Frequency (cm^{-1})	IR Intensity (KM/Mole)
50.8115	1.5189

Frequency (cm^{-1})	IR Intensity (KM/Mole)
85.8019	0.7885
164.8042	0.0326
229.6108	10.3191
239.4373	10.8113
261.9070	24.6291
294.3047	56.9123
315.1211	172.4171
375.7273	13.2707
399.6915	8.4536
483.2192	8.4949
521.3331	23.5120
612.7025	160.6282
670.4534	17.4089
757.7278	22.6882
814.5632	8.1478
845.8604	148.4178
913.2901	57.5912
953.8778	83.1607
1049.5269	22.6640

Frequency (cm^{-1})	IR Intensity (KM/Mole)
1095.3580	12.1585
1107.5992	13.7230
1122.4449	162.8366
1141.8287	268.8240
1223.1874	69.6805
1274.5603	53.4757
1290.2590	12.4705
1321.7619	43.4773
1368.5594	16.3381
1399.8921	13.8652
1430.7190	16.3688
1441.5244	20.0585
1485.6388	3.9717
1492.9748	8.8362
1661.7040	54.2682
1776.1009	423.9894
3016.3633	43.1848
3043.3870	17.1556
3066.1732	21.2909

Frequency (cm^{-1})	IR Intensity (KM/Mole)
3110.8154	26.3009
3117.2954	24.7721
3502.5685	3.8613
3575.9737	11.8017
3730.2088	88.1260
3816.7053	45.8384

Threonine (Thr1-OH) in methanol

Frequency (cm^{-1})	IR Intensity (KM/Mole)
45.9138	1.8936
87.7053	0.9544
165.6930	0.0348
228.7915	13.1817
239.4662	12.1745
258.7093	33.8781
293.9527	52.8312
315.8802	197.3420
375.6195	17.0971
400.3670	8.2702

Frequency (cm^{-1})	IR Intensity (KM/Mole)
482.5583	10.3200
521.7563	26.6557
608.1988	187.1358
669.5586	19.9040
756.4334	26.0672
814.9856	9.2058
847.1709	158.2919
913.3300	68.5612
952.4493	102.1860
1049.3768	27.0382
1094.7246	11.9952
1104.6113	20.2479
1120.5402	217.3323
1142.3671	270.2039
1221.5946	76.6249
1272.9623	61.6363
1288.9018	9.8126
1321.5999	53.5053
1368.9585	17.2910

Frequency (cm^{-1})	IR Intensity (KM/Mole)
1398.0205	15.7170
1431.2034	20.8172
1439.4753	21.2505
1480.7543	4.7274
1489.2436	10.1706
1657.5414	63.1712
1762.5211	513.4625
3021.7123	47.7871
3041.6438	20.4805
3073.2689	23.0969
3108.4223	33.9194
3115.3821	30.5182
3501.0622	4.9764
3573.6596	14.6732
3724.8667	100.0885
3812.7796	60.4112

Threonine (Thr1-OH) in water

Frequency (cm^{-1})	IR Intensity (KM/Mole)
45.4051	1.9479
87.7401	0.9776
165.4675	0.0386
228.6833	13.3346
239.0268	12.6368
258.0032	35.3567
293.8815	51.8054
316.3675	200.4802
375.6513	17.7262
400.3068	7.9003
482.4379	10.5543
521.8863	26.9032
607.8204	189.9521
669.3596	20.2600
756.1928	26.3544
815.0310	9.3285
847.2934	159.0071
913.4371	69.7765

Frequency (cm⁻¹)	IR Intensity (KM/Mole)
952.2790	104.5383
1049.2184	27.6401
1094.6790	11.8404
1104.4661	21.2750
1120.3634	224.9312
1142.4317	268.3717
1221.4244	77.4354
1272.9925	62.8032
1288.7671	9.6745
1321.5264	54.5809
1368.9983	17.2923
1397.7721	15.7261
1431.1426	21.5767
1439.0940	21.3827
1480.2056	4.8018
1488.8065	10.2916
1657.1268	64.1566
1760.7814	523.9157
3022.2674	48.2693

Frequency (cm^{-1})	IR Intensity (KM/Mole)
3041.5203	20.8918
3074.4068	23.3239
3108.1935	34.9723
3115.3524	31.1120
3500.8171	5.0839
3573.2635	14.9642
3724.2228	101.2840
3811.8176	62.2826

Deprotonated threonine (Thr1-O-) in gas phase

Frequency (cm^{-1})	IR Intensity (KM/Mole)
22.8815	6.0813
89.7071	2.2591
144.2889	15.7939
166.3304	30.8740
228.6556	2.0662
249.7142	4.4211
300.6754	9.9306
332.6265	54.8174

Frequency (cm^{-1})	IR Intensity (KM/Mole)
400.7272	19.5869
468.3808	62.3446
479.6866	18.6548
568.1200	67.6166
620.4981	74.5440
643.0792	31.2377
770.3977	33.9124
811.5266	99.5304
820.9322	77.0569
908.5083	43.1328
1026.0403	19.9643
1057.3780	15.1991
1073.0868	251.1512
1131.4343	118.7849
1160.5562	23.4026
1208.2798	119.8814
1280.3706	128.1313
1302.7178	25.6836
1320.4770	24.1954

Frequency (cm⁻¹)	IR Intensity (KM/Mole)
1351.5651	32.5486
1363.6785	198.1574
1429.2364	52.3430
1470.2162	0.6761
1483.2503	5.8839
1624.3134	59.5770
1740.2439	420.6363
2493.5019	528.1086
2991.4226	51.6504
3019.6915	37.5979
3059.0152	56.7094
3064.3297	55.2372
3475.6483	21.5560
3556.5860	0.1750
3764.8887	18.7395

Deprotonated threonine (Thr1-O-) in diethylether

Frequency (cm^{-1})	IR Intensity (KM/Mole)
56.2152	11.4484

Frequency (cm^{-1})	IR Intensity (KM/Mole)
88.4756	9.3800
158.9233	13.8128
189.9112	10.4150
201.7568	53.3485
257.0304	10.9155
308.3443	2.0322
386.6055	13.1281
407.6347	6.2468
488.6745	19.8318
501.4214	72.6872
589.2708	123.7635
648.2728	53.9690
688.2030	38.7514
785.4135	30.0458
826.2925	134.5578
843.6838	88.6483
924.8624	120.8647
1034.5773	37.5073
1066.5391	33.5130

Frequency (cm^{-1})	IR Intensity (KM/Mole)
1092.9536	218.7248
1127.6415	151.3049
1188.5039	42.5946
1198.2433	483.3464
1254.4570	51.8102
1305.4213	21.3381
1334.7500	32.3817
1342.3574	91.2626
1365.0769	26.4901
1432.9603	40.2935
1466.8050	3.1756
1476.1061	3.2765
1633.6006	76.5221
1735.6779	567.8825
2608.6521	519.3292
2999.0867	58.2235
3009.1057	58.0016
3063.7712	75.6386
3069.3263	74.1183

Frequency (cm⁻¹)	IR Intensity (KM/Mole)
3487.6739	3.0775
3563.6431	1.6530
3748.6034	59.1076

Deprotonated threonine (Thr1-O-) in methanol

Frequency (cm^{-1})	IR Intensity (KM/Mole)
58.0127	17.0073
76.8178	10.4956
160.2998	29.1281
189.3119	63.4057
222.6425	5.2572
257.8623	9.8813
311.2233	5.8121
385.8810	9.0343
413.2172	4.2016
485.7266	24.8781
506.4134	52.2230
591.7268	162.0019
651.6177	62.3123

Frequency (cm^{-1})	IR Intensity (KM/Mole)
710.4046	16.2735
791.1805	30.0755
832.1689	130.2519
854.2617	85.8419
930.8177	199.6934
1038.6729	41.2745
1065.5240	31.6337
1094.9576	214.1491
1120.6852	184.7910
1180.2345	512.8500
1196.9418	109.7056
1258.0216	17.9677
1304.2281	26.4529
1337.5001	30.5410
1346.1962	61.3730
1362.4880	37.3377
1432.7645	42.4933
1466.8745	5.5477
1473.7105	2.2775

Frequency (cm⁻¹)	IR Intensity (KM/Mole)
1635.0915	77.8354
1734.1495	648.4673
2672.5078	486.6111
3000.6290	62.2220
3009.1961	67.8573
3063.2918	86.8935
3072.7667	80.0896
3489.6627	0.3653
3562.4048	3.7545
3737.7253	82.6381

Deprotonated threonine (Thr1-O-) in water

Frequency (cm^{-1})	IR Intensity (KM/Mole)
52.2287	15.6219
81.3133	13.5251
157.6147	28.7506
187.4420	65.5859
225.5428	5.5773
258.8258	8.7968

Frequency (cm^{-1})	IR Intensity (KM/Mole)
312.0071	6.4434
385.0528	8.8968
413.6326	4.3036
486.0425	24.5729
507.3369	51.7710
592.5649	165.4892
651.7027	63.0907
711.7584	15.3977
791.4701	30.5610
832.6173	130.7218
855.1145	85.8056
931.6728	206.6456
1038.8768	41.2130
1065.5350	33.3413
1095.0697	217.0436
1120.1374	187.1681
1179.2176	526.0348
1196.7928	103.1796
1258.4620	17.3987

Frequency (cm^{-1})	IR Intensity (KM/Mole)
1303.9194	26.8646
1337.7383	29.8062
1347.5026	59.7153
1362.9850	38.2785
1432.6127	43.1977
1467.5246	5.8387
1473.7218	2.2603
1635.0269	78.1101
1733.8880	658.4863
2678.6708	483.4353
3001.4504	63.5653
3009.0288	68.2146
3064.1559	87.6438
3073.2938	81.1395
3490.0299	0.2440
3562.4267	4.0373
3736.2518	85.3621
